# Hibernation-Induced microRNA Expression Promotes Signaling Pathways and Cell Cycle Dysregulation in *Ictidomys tridecemlineatus* Cardiac Tissue

**DOI:** 10.3390/metabo13101096

**Published:** 2023-10-19

**Authors:** W. Aline Ingelson-Filpula, Kenneth B. Storey

**Affiliations:** Department of Biology, Carleton University, 1125 Colonel By Drive, Ottawa, ON K1S 5B6, Canada; alineingelsonfilpula@cmail.carleton.ca

**Keywords:** metabolic rate depression, hypometabolism, ground squirrel, hibernation, microRNA, signaling, epigenetics, post-translational modification

## Abstract

The thirteen-lined ground squirrel *Ictidomys tridecemlineatus* is a rodent that lives throughout the United States and Canada and uses metabolic rate depression to facilitate circannual hibernation which helps it survive the winter. Metabolic rate depression is the reorganization of cellular physiology and molecular biology to facilitate a global downregulation of nonessential genes and processes, which conserves endogenous fuel resources and prevents the buildup of waste byproducts. Facilitating metabolic rate depression requires a complex interplay of regulatory approaches, including post-transcriptional modes such as microRNA. MicroRNA are short, single-stranded RNA species that bind to mRNA transcripts and target them for degradation or translational suppression. Using next-generation sequencing, we analyzed euthermic vs. hibernating cardiac tissue in *I. tridecemlineatus* to predict seven miRNAs (let-7e-5p, miR-122-5p, miR-2355-3p, miR-6715b-3p, miR-378i, miR-9851-3p, and miR-454-3p) that may be differentially regulated during hibernation. Gene ontology and KEGG pathway analysis suggested that these miRNAs cause a strong activation of ErbB2 signaling which causes downstream effects, including the activation of MAPK and PI3K/Akt signaling and concurrent decreases in p53 signaling and cell cycle-related processes. Taken together, these results predict critical miRNAs that may change during hibernation in the hearts of *I. tridecemlineatus* and identify key signaling pathways that warrant further study in this species.

## 1. Introduction

The thirteen-lined ground squirrel is a rodent that lives throughout the central United States and Canada. It is an obligate hibernator that hibernates circannually at a set time every year regardless of specific environmental conditions [1]. By contrast, facultative hibernation is displayed by some species that undergo short torpor bouts in direct response to environmental conditions [2]. With regard to *I. tridecemlineatus*, the animals prepare in late summer/early autumn by consuming large amounts of food (hyperphagia) and building up lipid stores to the point of obesity [3]. Hibernation bouts in *I. tridecemlineatus* consist of multiple periods of deep torpor, ranging from days to weeks in duration, during which core body temperature (T_b_) drops to near-ambient (but not below 0 °C), and metabolic energy expenditure can be reduced to as low as 1–5% of euthermic rates [1]. Squirrels cycle through prolonged periods of torpor interspersed with brief interbout arousals when T_b_ rises again to euthermic values for approximately one day before another bout of deep torpor is induced [4]. Despite these periods of arousal, having a low T_b_ during torpor allows squirrels to save up to 90% of the energy that would otherwise be needed to remain euthermic during the winter [5]. In order to decrease their metabolic rate so strongly, squirrels undergo multiple physiological, biological, and molecular alterations, including downregulation of all nonessential processes. However, this can leave squirrels susceptible to detrimental environmental conditions, such as hypoxia or ischemia, due to a prolonged reduction in heart rate and breathing (sometimes including long periods of breath-hold or apnea) in organ systems [6].

Metabolic rate depression (MRD) is broadly classified as the global downregulation of nonessential genes and processes to conserve endogenous fuel resources and prevent the buildup of toxic waste byproducts. MRD is employed by many animal species, both invertebrate and vertebrate, to contend with extreme environmental stresses including: reduced oxygen (hypoxia); lack of oxygen (anoxia); subzero temperatures during winter (freeze tolerance and/or freeze avoidance); and lack of water (dehydration/estivation) [7,8]. During MRD, the metabolic rate can be lowered to just 5–40% of the resting rate under normal environmental conditions, with the ability to reverse when favorable conditions return with very little damage to cells and tissues [9]. Due to these aforementioned characteristics, MRD is indisputably a cornerstone of hibernation—both in ‘cold hibernators’ such as *I. tridecemlineatus* as well as ‘warm hibernators’ such as the brown bear *Ursus arctos* [1,10]. The heart and respiration rate are slowed, animals typically become immobile, and the kidney filtration rate is reduced [11]. To facilitate this, ATP turnover is completely restructured at the protein synthesis/degradation, urea synthesis/degradation, gluconeogenic, and ATPase levels [12,13]. Energy-expensive pathways such as the cell cycle are suppressed, a feature observed in multiple species undergoing MRD [14,15,16]. Fuel usage is often switched from carbohydrates to fatty acids, while metabolism adjusts accordingly between aerobic and anaerobic pathways [17,18]. The cessation of food intake, the global reorganization of virtually every aspect of cellular metabolism, and the ability for this state to be rapidly reversed without catastrophic damage to cells and tissues necessitates a well-coordinated interplay of regulatory mechanisms at the molecular level to ensure its success.

MicroRNAs (miRNAs) are one such regulatory mechanism. These short, single-stranded, noncoding RNA species bind to mRNA transcripts through an 8 nt ‘seed sequence’ and target them for degradation or translational suppression [19]. One mRNA can be targeted by multiple miRNAs, and likewise, one miRNA can bind multiple mRNAs [19]. This adaptability allows for near-limitless regulatory potential—and indeed, we observe differential regulation of miRNA in many animals that use MRD to survive extreme environmental stress [20,21,22,23]. This includes hibernation, as many studies have identified miRNAs as key players in maintaining skeletal muscle integrity, altering fuel usage, facilitating MRD as a whole, and preventing other molecular forms of damage that occur during hibernation [24,25,26,27,28,29,30,31]. In *I. tridecemlineatus* specifically, miRNAs fluctuate on a tissue-dependent basis, playing roles in cytoskeletal and DNA binding, protein signaling through GTPase and transferase activity, lipid oxidation, and antioxidant defenses [26,32,33]. Therefore, it stands to reason that identifying miRNA in the currently understudied tissues of *I. tridecemlineatus* would reveal further functional roles of these RNA subtypes during hibernation.

This study aimed to examine miRNA’s influence on cardiac tissue in control vs. hibernating *I. tridecemlineatus*. Cardiac tissue is critical for life and is therefore an explicable target for research and further understanding. During hibernation, ground squirrel cardiac muscle becomes hypertrophic to better pump cold, viscous blood, while skeletal muscle resists atrophy under extended periods of disuse [34,35]. In the hypometabolic state, myocytes strategically alter key pathways and reprioritize cellular demands depending on the particular needs of the organism. We hypothesized changes to these effects: shifts in energy-expensive processes, including the cell cycle and signaling pathways dependent on pertinent survival functions; altered pathways related to energy metabolism, given that MRD is hallmarked by transitions between carbohydrate and lipid fuel usage; and cardiac-specific remodeling to facilitate tissue integrity. Therefore, isolating how miRNA post-transcriptional regulation facilitates changing molecular processes in cardiac tissue during hibernation will shed more light on understanding MRD and hibernation in this species. Next-generation sequencing (NGS) was used to create a small RNA-seq dataset, from which mature miRNAs were screened out and run through bioinformatic suites to identify individual miRNAs and the molecular processes they affect.

## 2. Materials and Methods

### 2.1. Animal Collection

Female thirteen-lined ground squirrels (*Ictidomys tridecemlineatus*) were wild-captured in August in proximity to the University of Manitoba Carman Research Station (49°30′ N, 98°01′ W). The squirrels were trapped with nets and water buckets, treated with ivermectin and Droncit for endoparasites (0.4 mg/kg, subcutaneous), and given a flea spray for ectoparasites immediately after capture. The animals were captured with the approval of the Manitoba Conservation and Water Stewardship under wildlife scientific permit WB15027 and transported to the University of British Columbia, Vancouver, BC. Dr. Ryan Sprenger’s laboratory conducted all further hibernation experiments. 

Ground squirrels (weighing 150–300 g and aged 1–3 years old) were anesthetized with 5% isofluorane and subcutaneously injected with one of two sensor chips: either implantable RFID temperature chips (ITPP-300 extended calibration) read with a DAS-8017 reader, which came precalibrated by the company (resolution: 0.1 °C; accuracy: 0.2 °C), or wireless real-time telemeters (CTA-F40 and TA-F40; Data Sciences International, St. Paul, MN, USA). The telemeters were read continuously with an MX2 and PhysioTel connect system. Both telemeters were calibrated using two-point calibration. To ensure comparable measurement, six animals were co-implanted with both systems, and the two read within 0.2 °C of each other. (IPTT-200; Bio Medic Data Systems, Seaford, DE, USA). Animals were housed individually in shoebox cages in a holding room with an ambient temperature of 21 °C under a 12/12 h light/dark cycle and fed IAMS small chunk dog chow (>25% protein per care standards for this species; [36]) supplemented with apples and peanuts ad libitum. during the euthermic period (April to October). No food or water was provided during the hibernation season (October to April). All animals were kept in a temperature-controlled chamber (20 °C ± 2 °C) on a photoperiod that matched the daily photoperiod in Vancouver during the euthermic period. Experiments were conducted only during the middle of the hibernation season (November to March).

Euthermic animals (control group) were sampled in August prior to transfer to the hibernaculum. At the end of October, the remaining ground squirrels were transferred to the hibernaculum in cages containing wood shavings and held at constant darkness at 5 ± 2 °C and 60% humidity. Body temperature (T_b_), time, and respiration rate were monitored via subcutaneous chips and used to determine the stage of hibernation. Experiments were performed from December to March, and all animals (except summer animals) had been through a series of torpor–arousal bouts prior to sampling. Hibernating animals were sampled at late torpor, designated by remaining in the deep torpor phase of the hibernation bout for 5 days and without beginning an interbout arousal (T_b_ = 5–8 °C). Squirrels were removed from the hibernation chamber, anesthetized with 5% isofluorane, and sacrificed by decapitation within 2 min. Tissue samples were rapidly dissected, flash-frozen in liquid nitrogen, delivered to Carleton University on dry ice, and stored at −80 °C until use. All procedures were conducted under a protocol approved by the University of British Columbia Animal Care Committee (UBC A17-0018 and A19-0250) and were in compliance with the policies of the Canadian Council on Animal Care.

### 2.2. RNA Extraction

Total RNA was extracted from cardiac tissue of euthermic and hibernating *I. tridecemlineatus* (*n* = 4 for both conditions). Aliquots of tissue weighing ~50 mg were crushed under liquid nitrogen, homogenized in 1 mL of TRIzol reagent (Invitrogen, Carlsbad, CA, USA; Cat. # 15596-018) before adding 200 µL of chloroform, and centrifuged at 4 °C at 10,000× *g* for 15 min. The upper aqueous phase—containing total RNA—was pipetted to a sterile microcentrifuge tube at room temperature containing 500 µL of 2-propanol and incubated for 10 min to form an RNA precipitate. Samples were centrifuged at 10,000× *g* for 15 min at 4 °C to form an RNA pellet, washed 2× with 70% ethanol, and air-dried for 10–15 min. RNA pellets were resuspended with 50 µL of RNAse-free water. The concentration/purity of total RNA was determined using a BioTek Take3 microspot plate and a PowerWave HT microplate spectrophotometer, and RNA integrity was further validated with an OD 260/280 ratio of ~2.0 and 1% agarose gel electrophoresis.

### 2.3. Small RNA Sequencing

Total RNA from the cardiac tissue of euthermic vs. hibernating *I. tridecemlineatus* was sequenced by the BC Cancer Agency (Vancouver, BC, Canada). RNA quality was corroborated using an Agilent Bioanalyzer 2100 (Agilent Technologies, Santa Clara, CA, USA), miRNA libraries were constructed, and small RNA cDNA libraries were assembled according to protocols in [37]. All cDNA libraries were validated with the Bioanalyzer before sequencing on an Illumina HiSeq 2500 platform.

### 2.4. Read Processing

The subsequent processing steps are outlined in [38,39]. Small-RNA raw data files in .fastq format were received from BC Cancer Agency and uploaded to the SRA database (available at SRA Accession: PRJNA1010398). Cutadapt was used to filter out low-quality reads and excise the 6-nucleotide adapters [40]. To validate both successful adapter removal and proper distribution of small RNA lengths to ~25 nt, fastqc was run for each sample [41]. In order to differentiate mature miRNA sequences from other non-miRNA small RNAs, reads were first aligned to a negative reference file (compiled from Rfam [42] and piRNABank databases [43]) and eliminated using bowtie [44]. The remaining reads not screened out in this process were aligned using bowtie to a positive reference file composed of mature miRNA sequences from miRBase, the miRNA database [45]. The parameters were perfect seed sequence matches, and an overallsequence length of 20 nt to ensure accurate mature miRNA matches. The reads that aligned to mature miRNA sequences were sorted, and samtools was used to generate read counts for each miRNA [46]. MiRNAs with fewer than four reads were discarded, and read counts were normalized using the voom method [47] as detailed in [39].

### 2.5. Differential Expression Analysis and Clustering

The differential expression of miRNAs between euthermic and hibernating ground squirrels was quantified using the R package limma [48]. Statistically significant differentially expressed miRNAs were defined by (1) a false discovery rate (FDR)-corrected *p*-value < 0.05 and (2) an absolute log_2_ fold change of ≥1.5. Significantly differentially expressed miRNAs were hierarchically clustered via the Ward method by both miRNA and sample [49].

### 2.6. Gene Set Analysis

The RBiomirGS R package [50] was used for gene set analysis to delineate significantly enriched gene ontology (GO) terms [51] and Kyoto Encyclopedia of Genes and Genomes (KEGG) pathways [52]. RBiomirGS quantifies miRNA:mRNA interactions by calculating both a miRNA score (*S_microRNA_*) and a mRNA score (*S_mRNA_*) to enumerate the number of potential interactions between different miRNAs and individual mRNA targets, all compiled from multiple databases [53]. 

The *S_mRNA_* of the mRNAs was used to distinguish significantly enriched gene sets (FDR-adjusted *p*-value ≥ 0.05) and corresponding model coefficients for each GO term and KEGG pathway. Estimated model coefficients and standard error were calculated via a logistic regression-based method and were then applied to determine statistical significance [50]. A negative model coefficient signified that the GO term/KEGG pathway was downregulated due to increased negative regulation by miRNA, and a positive model coefficient indicated that the regulation of the process/pathway by miRNA was decreased and could result in upregulation. 

### 2.7. Statistical Analysis and Visualization

Matplotlib and seaborn Python packages were used to generate heatmaps and biplots for gene set analysis [54,55]. Microsoft Excel was used to generate differential expression and GO/KEGG volcano plots. Hierarchical clustering of significantly differentially regulated miRNAs was produced by the gplots R package (version 3.1.3) [56].

## 3. Results

### 3.1. Small RNA Sequencing Summary

The mean raw reads from an *n* = 4 of euthermic vs. hibernating *I. tridecemlineatus* hearts, respectively, were 16,196,407 ± 40% and 12,574,355 ± 54%. Trimming and quality filtering via cutadapt yielded 14,364,366 ± 43% control reads and 10,300,495 ± 57% torpid reads. Subsequent to negative filtering, 4,732,846 ± 30% reads in the control aligned with 78 mature miRNA sequences, whereas 4,378,780 ± 47% reads aligned in the hibernating group.

### 3.2. Differential Expression of miRNA in Response to Hibernation

**Figure 1:**
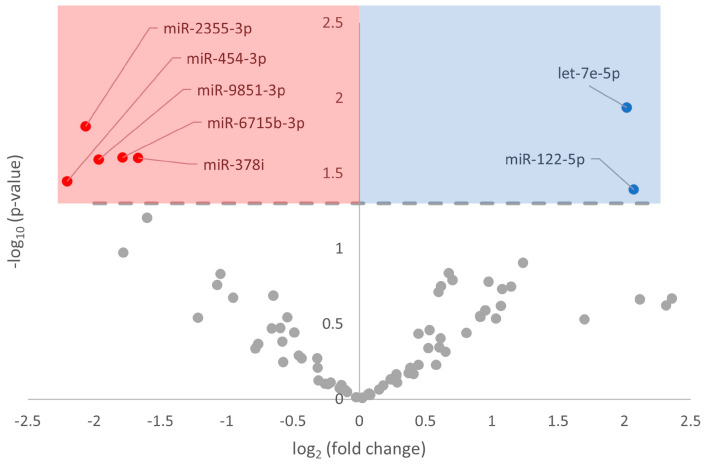
Volcano plot of differentially expressed miRNA in euthermic vs. hibernating *I. tridecemlineatus* hearts. Fold-change thresholds were set to ±log_2_ 1.5 and a false discovery rate (FDR)-adjusted *p*-value < 0.05. Red markers indicate significantly downregulated miRNA and blue markers indicate significantly upregulated miRNA. Grey circles are miRNAs that did not pass the fold-change and *p*-value thresholds.

Of the 78 mature miRNAs that matched with the quality-filtered reads, 7 demonstrated a statistically significant (FDR-enriched *p*-value < 0.05) change during hibernation compared with euthermia (Figure 1). More specifically, five miRNAs (miR-2355-3p, miR-6715b-3p, miR-378i, miR-9851-3p, and miR-454-3p) were downregulated and two miRNAs (let-7e-5p, miR-122-5p) were upregulated (Figure 1). These results were visualized using a volcano plot, with red markers corresponding to significantly downregulated miRNAs and blue markers corresponding to significantly upregulated miRNAs (Figure 1, Supplementary Table S1).

### 3.3. Gene Ontology Terms Enriched for Differentially Expressed miRNA

**Figure 2:**
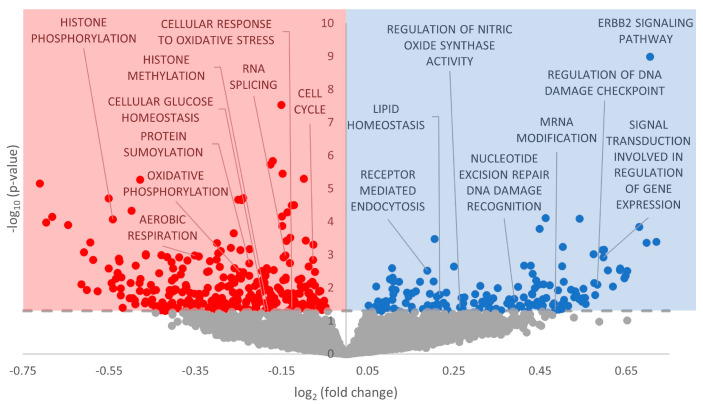
Volcano plot of GO Biological Processes in euthermic vs. hibernating *I. tridecemlineatus* hearts. Fold-change thresholds are set to ±log_2_ 1.5 and a false discovery rate (FDR)-adjusted *p*-value < 0.05. Red markers indicate significantly downregulated terms and blue markers indicate significantly upregulated terms. Grey circles are terms that did not pass the fold-change and *p*-value thresholds.

**Figure 3:**
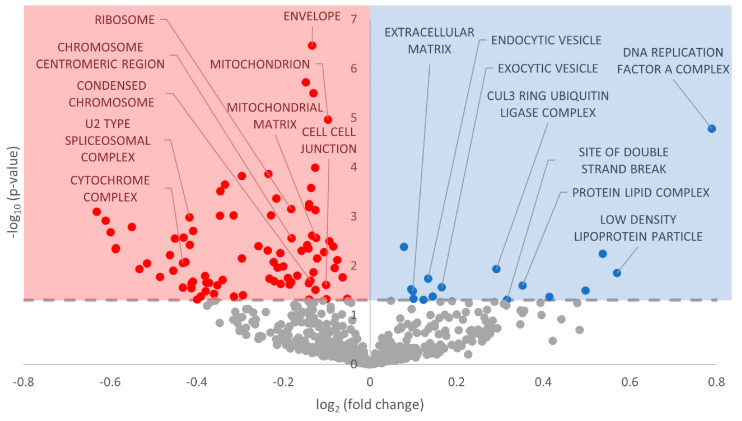
Volcano plot of GO Cellular Compartment in euthermic vs. hibernating *I. tridecemlineatus* hearts. Fold-change thresholds are set to ±log_2_ 1.5 and a false discovery rate (FDR)-adjusted *p*-value < 0.05. Red markers indicate significantly downregulated terms and blue markers indicate significantly upregulated terms. Grey circles are terms that did not pass the fold-change and *p*-value thresholds.

**Figure 4:**
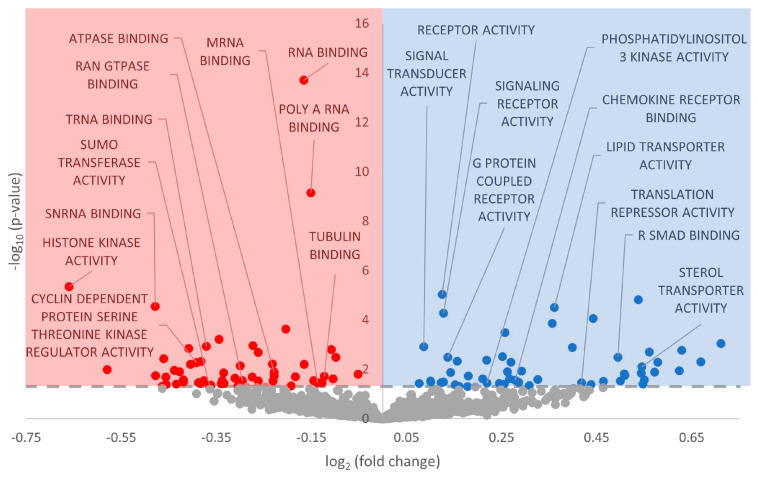
Volcano plot of GO Molecular Function in euthermic vs. hibernating *I. tridecemlineatus* hearts. Fold-change thresholds are set to ±log_2_ 1.5 and a false discovery rate (FDR)-adjusted *p*-value < 0.05. Red markers indicate significantly downregulated terms and blue markers indicate significantly upregulated terms. Grey circles are terms that did not pass the fold-change and *p*-value thresholds.

Gene set enrichment analysis was performed on the GO Biological Processes, GO Cellular Compartment, and GO Molecular Function databases. With regard to GO Biological Processes, 451 terms were statistically significantly enriched (FDR-adjusted *p*-value < 0.05), with more downregulated than upregulated (Figure 2, Supplementary Table S2). For GO Cellular Compartment, 102 terms were significantly enriched, again with a heavy skew toward downregulation (Figure 3, Supplementary Table S3). Finally, 110 terms were enriched in GO Molecular Function, with an almost perfect split between downregulated and upregulated terms (Figure 4, Supplementary Table S4).

### 3.4. KEGG Pathways with Reduced miRNA Regulation

**Figure 5:**
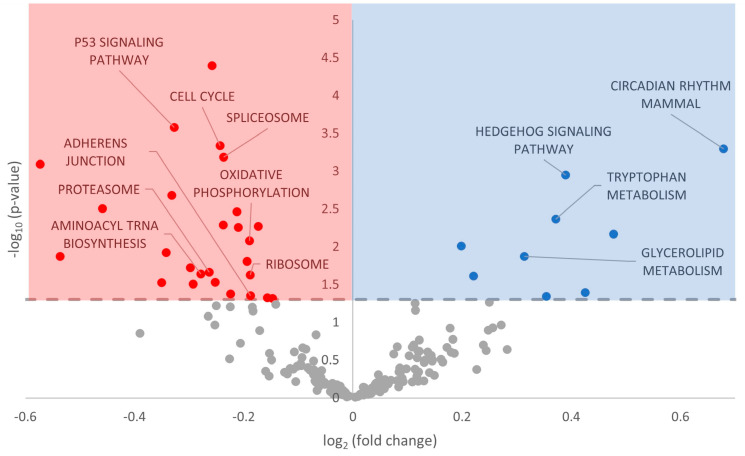
Volcano plot of KEGG Pathway Analysis in euthermic vs. hibernating *I. tridecemlineatus* hearts. Fold-change thresholds are set to ±log_2_ 1.5 and a false discovery rate (FDR)-adjusted *p*-value < 0.05. Red markers indicate significantly downregulated pathwaysand blue markers indicate significantly upregulated pathways. Grey circles are pathways that did not pass the fold change and *p*-value thresholds.

The Kyoto Encyclopedia of Genes and Gene Processes (KEGG) database was also screened to determine a potential miRNA influence on particular cellular pathways. A total of 35 KEGG pathways were differentially regulated during hibernation, with 9 demonstrating upregulation and 26 showing downregulation (Figure 5, Supplementary Table S5).

## 4. Discussion

### 4.1. Upregulation of ErbB2 Signaling and Implications in Downstream Signaling

In GO Biological Processes, the term GO ErbB2 Signaling was the most drastically changed (upregulated) during hibernation, with a fold change of 9 and a *p*-value of 1.02 × 10^−9^ (Figure 2, Supplementary Table S2). ErbB2 is a member of a family of protein tyrosine kinase receptors (which include ErbB1, ErbB2, ErbB3, and ErbB4) that are bound the by ligand neuregulin-1 (NRG-1) [57]. Broadly, coupling with NRG-1 forms the NRG-1/ErbB signaling axis, which is indispensable for both cardiac and neuronal development and was proven to be embryonically lethal in knockout mouse models [58]. 

ErbB2 (also referred to as HER2) is most widely known for its roles in breast cancer, in which the ErbB2 receptor is overexpressed in 25% of tumors [59]. While ErbB2 cannot bind NRG-1 directly, it forms heterodimers with ErbB3 and ErbB4 and is essential for ErbB signaling in the mature heart [57]. During adulthood, the NRG-1/ErbB signaling axis mainly regulates cell survival/growth during cellular stress (largely governed by NRG-1) and sympathovagal cardiac control systems [60,61,62,63]. Mouse cardiomyocytes implicate NRG-1 and ErbB2/4 in survival regulation, hypertrophy, proliferation, cell–cell contact between cardiomyocytes, and anti-apoptosis in response to oxidative stress [61,63,64]. We observed that GO terms corroborated these downstream effects, including many involving cellular adhesion, such as GO Cell Junction Organization, GO Cell–Cell Junction, GO Chemokine Receptor Binding, and GO Adherens Junction (Figure 2, Figure 3, Figure 4 and Figure 5, Supplementary Tables S2–S5). Multiple in vitro studies have supported this reasoning by showing that interference with ErbB2 signaling promotes a proapoptotic cascade in cardiomyocytes and inhibits pro-survival pathways.

Given the aforementioned functional roles of the NRG-1/ErbB signaling axis, it follows that this signaling pathway is differentially regulated during hibernation. As T_b_ and heart rate decrease during torpor, cardiac remodeling helps to pump cold and viscous blood to critical organs and shunt it away from peripheral, splanchnic organs [1]. It is widely known that ischemia–reperfusion-induced oxidative stress is a common side effect of hibernation in many tissues [65,66,67,68]. Broadly speaking, oxidative stress occurs from the transformation of O_2_ gas into negatively charged reactive oxygen species (ROS), including superoxide, hydrogen peroxide, hydroxyl radical, singlet oxygen, ozone, lipid peroxides, nitric oxide, and peroxynitrite [69]. These ROS are incredibly disruptive to cellular function (especially DNA) due to their negative charge, and therefore, many enzymes exist for antioxidant defense (catalase, superoxide dismutases, glutathione, peroxidases, and redoxins, to name a few) [69]. In hibernating *Spermophilus dauricus*, levels of hydrogen peroxide and superoxide dismutase 2 are elevated in the heart [67]. The authors hypothesized that the increase in these reactive oxygen species (ROS) activated the Nrf2/Keap antioxidant pathway to protect major organs from damage throughout hibernation [67]. In the *I. tridecemlineatus* intestine, elevated oxidative stress from hibernation triggers the NFκB pathway to mitigate potential damage [68]. However, in the heart, oxidative damage is markedly reduced during hibernation compared with interbout arousal when mitochondrial metabolism resumes and ROS are generated once more [70]. The upregulation of ErbB2 signaling via miRNA could thus be enacted for an anti-apoptotic role in preparation for oxidative stress defense during interbout arousal; however, many GO terms in GO Biological Processes showed a marked decrease in oxidative stress defense. For example, GO Cellular Reaction to Oxidative Stress and GO Cellular Response to Reactive Oxygen Species both had negative model coefficients in GO Biological Processes, suggesting that the increase in ErbB signaling via miRNA is not translated to oxidative stress defenses (Figure 2, Supplementary Table S2). Therefore, we can look elsewhere for alternative downstream functions resulting from ErbB activation.

ErbB signaling is linked with other major signaling pathways, including MAPK and PI3K/Akt, both of which change during MRD and extreme environmental stress survival. Akt in particular also leads to an anti-apoptotic response, while ERK1/3 activation induces a hypertrophic response. Preliminary research has already been conducted into MAPK signaling during hibernation in the *I. tridecemlineatus* heart [71]. The authors uncovered fluctuations in phospho-kinases and anti-apoptotic factors across the torpor–arousal cycle, broadly directing cellular function toward a decrease in pro-apoptotic signals during hibernation and an increase in hypertrophy during interbout arousals [71]. Other research has reinforced an increase in MAPK factors ERK, p38, and JNK in cardiomyocytes, specifically in apoptosis induced by ischemia–reperfusion injury [72]. The upregulation of let-7e-5p is already implicated as a blood biomarker for ischemic stroke [73], which mirrors our results and highlights the importance of ischemia defenses during hibernation, further supporting ischemia-targeted processes (Figure 1). Therefore, there is evidence to support ErbB2 signaling activation via downstream increases in MAPK signaling to fulfill anti-apoptotic roles.

With regard to Akt signaling, we observed a statistically significant GO term, GO Phosphatidylinositol 3 Kinase Activity, providing a further connection to the P13K/Akt pathway (Figure 2). Akt is differentially regulated during hibernation in various tissues—a study on *Spermophilus richardsonii* showed a decrease in both Akt enzyme activity and total protein levels of phospho-active Akt in the liver and skeletal muscle [74]. In hibernating marsupial *Dromiciops gliroides*, downregulated miRNAs, as measured by RT-qPCR, may promote PI3K-Akt function in the liver [75]. These results were corroborated in the hibernating grey mouse lemur *Microcebus murinus*, where the activation of insulin-dependent signaling (possibly through Akt) was upregulated in the liver [76]. Finally, in the intestine of *I. tridecemlineatus*, Akt increased 20-fold during hibernation [77]. While specific studies on cardiac tissue of *I. tridecemlineatus* have not been performed, there is precedent to suggest that Akt may be activated during hibernation—lending credence to upregulated ErbB2 signaling being specifically targeted toward Akt regulatory roles. It is important to note that PI3K/Akt signaling may also lead to cell cycle progression, which will be addressed in the following section.

### 4.2. Cell Cycle Processes Appear Heavily Downregulated by miRNA

Numerous statistically significant GO terms from GO Biological Processes, GO Cellular Compartment, and GO Molecular Function that involve the cell cycle had a negative model coefficient (GO Mitotic Cell Cycle and GO Cell Cycle Process), along with overall KEGG Cell Cycle (Figure 2, Figure 3, Figure 4 and Figure 5; *p* < 0.05). This points to a strong downregulation of cell cycle processes enacted via miRNA—a not-unexpected result given the overarching themes of MRD, particularly the downregulation of energy-expensive genes and processes. Many animals that use MRD as a survival strategy display concurrent decreases in cell cycle processes, including freeze-tolerant *Rana sylvatica*, anoxia-tolerant *Orconectes virilis*, and anoxia-tolerant *Trachemys scripta elegans* [16,78,79]. MiRNA involvement in the post-transcriptional suppression of cell cycle proteins was likewise documented in *T. s. elegans*, given that miR-16-1 and miR-15a were implicated in suppressing levels of cyclin D1 during anoxia [78]. In *Apostichopus japonicus*, an aestivating sea cucumber, miR-200-3p was verified to bind to cyclin A and inhibit protein production in the intestine [80].

With regard to hibernation specifically, decreases in cell cycle processes have been observed in other tissues of *I. tridecemlineatus*. Wu and Storey found decreases in total protein levels of cyclins D1 and E, with varying levels of Cdk proteins across the different phases of hibernation [15]. Overall, the interplay of cell cycle factors pointed toward cell cycle arrest in the G_1_ phase and at the G_1_/S checkpoints before resuming euthermic function upon arousal [15]. It is important to note that arrest before the S phase avoids the energy-expensive process of DNA synthesis, keeping in line with MRD principles. Indeed, this is supported by our observation of many GO terms with negative model coefficients implicated in DNA synthesis, including GO Membrane Disassembly, GO Attachment of Spindle Microtubules to Kinetochore, GO Cell Cycle Process, GO Chromosome Separation, GO Sister Chromatid Segregation, and GO Regulation of Cyclin-Dependent Protein Kinases, to name a few (Figure 2; Supplementary Table S2).

While further research needs to be conducted on specific cyclin and Cdk levels in the hibernating squirrel heart, our results point toward a similar pattern of cellular quiescence during hibernation in *I. tridecemlineatus*. This further suggests that altered PI3K/Akt signaling does not serve to activate cell cycle progression and instead plays other functional roles (including anti-apoptosis, as mentioned earlier).

### 4.3. Downregulation of p53 Signaling

We observed that the KEGG P53 Signaling Pathway was significantly downregulated, with a negative model coefficient and a *p*-value < 0.0003 (Figure 5; Supplementary Table S5). Tumor-suppressing transcription factor p53 is widely implicated in DNA repair, apoptosis, autophagy, and cellular survival. Its classification as an oncogene clearly links the roles of cell growth, senescence, and apoptosis, given that these are hallmark cancer characteristics. However, numerous studies have demonstrated p53 responses to hypoxia, DNA damage, oxidative stress, and glycolytic pathways, all of which are differentially regulated in response to extreme environmental stress and MRD. There have been many studies identifying that p53 plays a functional role in mitigating these stresses in *Rana sylvatica* and *Trachemys scripta elegans*, which experience anoxia in their natural environments [81].

While studies on p53 during hibernation in *I. tridecemlineatus* are limited, preliminary research has shown the upregulation of p53 in skeletal muscle [82]. While levels of total p53 were upregulated during hibernation, there were no observable changes in the measured post-translationally modified p53, which would suggest downstream pathways [82]. The transcript levels, nuclear localization, and DNA binding capacity of p53 did increase during hibernation, making the apparent “lack” of downstream effects perplexing and warranting future study [82]. However, given the concurrent lack of the DNA damage sensors ataxia telangiectasia mutated (ATM) protein and ataxia telangiectasia and Rad3-related (ATR) protein, the authors suggested that p53 was not being activated by increased DNA damage during hibernation, as might be postulated [82]. 

Our predicted decrease in p53 signaling, therefore, is not out of place in the existing literature. For example, levels of nuclear p53 have been measured at fourfold lower levels during hibernation in the intestinal mucosa of *I. tridecemlineatus* [77]. Regulators of p53 likewise vacillate throughout the torpor–arousal cycle (e.g., the Mdm family, RPL26, p53BP2) [83]. It is possible that p53 acting as an oncogene, i.e., linked with cellular proliferation and growth, is the deciding factor. MRD is strongly linked to transcriptional and translational suppression to conserve cellular energy, and therefore, having signaling pathways promoting cellular growth would run counter to the principle of survival. Also, the protective functions of p53 (hypoxia, DNA damage, oxidative stress, and glycolysis) share considerable overlap with ErbB and MAPK signaling, which are hypothesized to be differentially regulated during hibernation (Figure 2 and Figure 4; Supplementary Tables S2 and S4). It is possible that p53 is downregulated due to its roles in cellular proliferation and growth, while other pathways are activated to serve pro-survival functions in the absence of p53.

### 4.4. Global MRD and Other Modes of Regulation

Aside from the unique signaling changes discussed above, many differentially regulated GO terms referred to changes in fuel usage, a hallmark characteristic of MRD. Such terms included GO Lipid Homeostasis, GO Lipid Localization, and GO Lipid Translocation in GO Biological Processes; GO Carbohydrate Binding, and GO Lipid Transporter Activity in GO Molecular Function, as well as KEGG Oxidative Phosphorylation and KEGG Glycerolipid Metabolism in KEGG Pathway (Figure 2, Figure 3, Figure 4 and Figure 5; Supplementary Tables S2–S5). During hibernation, fuel usage switches from carbohydrate metabolism to lipid oxidation, drawing on the increased stores of lipids from hyperphagia during the fall [1]. A reduction in oxidative phosphorylation, the main ATP-generating step of aerobic respiration, is perfectly in line with other examples of MRD and is demonstrated in Figure 2 [4,7,11]. We also observed many mitochondrial-related GO terms with negative model coefficients to corroborate this assertion, including GO Mitochondrion, GO Mitochondrial Matrix, and GO ATPase Binding (Figure 3 and Figure 4).

It is important to note that this study and all the hypotheses herein were predicated on miRNA influence alone. In reality, this does not take into account the in vivo conditions of the entire cellular environment, which consists of many regulatory mechanisms other than miRNA. Epigenetic modifications, including DNA and histone modifications, and post-translational modifications, including methylation, acetylation, ubiquitination, and SUMOylation, all work in tandem to elicit functional changes [84,85,86]. 

Another example of post-transcriptional regulation is alternative splicing, in which various exons in mRNA transcripts are integrated or discarded into the final transcript which is directed for translation [87]. Alternative splicing patterns can fluctuate in response to physiological conditions, allowing an organism to react to environmental changes by altering specific portions of its genome, even extending to changes in individual genes [87]. Many GO terms related to alternative splicing were predicted to be differentially regulated in response to miRNA, including GO RNA Splicing, GO mRNA Modification, GO U2-Type Spliceosomal Complex, GO mRNA Binding, and GO Spliceosome (Figure 2, Figure 3, Figure 4 and Figure 5; Supplementary Tables S2–S5). The majority of these terms had negative model coefficients, indicating that miRNA influence may downregulate the frequency of alternative splice variants in the cell. However, GO mRNA Modification had a positive model coefficient, suggesting that other mRNA modifications may serve a prioritized role for regulatory support (Figure 2; Supplementary Table S2). There are over 100 types of RNA modifications that alter the fate of mRNA transcripts, including m^7^G, ψ, 3′ poly(A) tail, and m^6^A modifications [88]. There is an indication that poly(A) modifications may be downregulated, given the corresponding GO term GO Poly A RNA Binding in GO Molecular Function (Figure 4). However, further research can be conducted to elucidate the crosstalk that may occur between miRNA and alternative splicing, including other mRNA modifications.

With regard to post-translational modifications, we saw terms including GO Histone Phosphorylation, GO Histone Methylation, GO Protein SUMOylation, and GO SUMO Transferase Activity in GO Biological Processes and GO Molecular Function with negative model coefficients and statistically significant *p*-values (Figure 2 and Figure 4; Supplementary Tables S2 and S4). SUMOylation, or small ubiquitin-like modifier, is a post-translational modification that affixes a 100-residue peptide onto the epsilon amino group of specific lysine residues. SUMOylation has existing ties to hibernation and especially ischemia tolerance—in the brains of thirteen-lined ground squirrels, there is a massive increase in SUMOylation to mitigate damaging effects on this sensitive organ [89,90]. It is interesting that predicted miRNA would downregulate SUMO in cardiac tissue instead of upregulating it like in brain tissue. However, perhaps other epigenetic, post-transcriptional, or post-translational mechanisms counteract this effect and SUMOylation is at euthermic or activated levels. Conversely, SUMOylation may be truly downregulated, and its protective effects may be rescued by other cellular defenses. This is a possibility considering that there were also GO terms with positive model coefficients related to DNA damage, including GO Nucleotide Excision Repair DNA Damage Recognition and GO Site of Double Strand Break (Figure 2 and Figure 3; Supplementary Tables S2 and S3). There has been introductory research into post-translational modifications during hibernation in *I. tridecemlineatus* [84,91,92,93], although no studies have yet been conducted on cardiac tissue. Further study will allow us to contextualize miRNA regulation in the broader whole of epigenetic, post-transcriptional, and post-translational modifications during hibernation.

## 5. Conclusions

**Figure 6:**
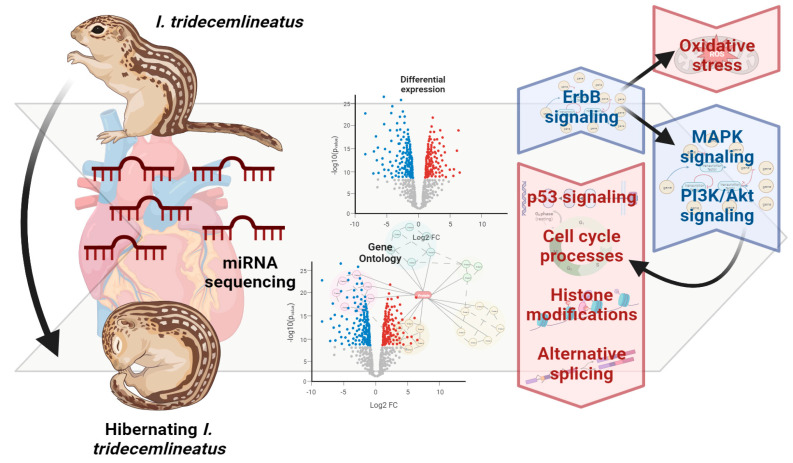
A schematic of the miRNA-induced changes in downstream pathways and their interrelations in the euthermic vs. hibernating *I. tridecemlineatus* heart. Figure made using BioRender.com.

Overall, we predict that miRNA regulation results in a strong activation of ErbB2 signaling, and concurrent decreases in p53 signaling and cell cycle-related processes (Figure 6). Signaling through ErbB receptors may further trigger PI3K/Akt and MAPK signaling (through ERK) according to the statistically upregulated GO term related to these downstream pathways. ErbB2 signaling also serves tissue-specific functions, including cardiac remodeling, hypertrophy, and signaling in cardiomyocytes. Via PI3K/Akt, cell cycle progression is negatively regulated due to the overarching themes of MRD and suppression of energy-expensive processes that are not critical for pro-survival means. Therefore, PI3K/Akt activation may lead to anti-apoptotic roles, antioxidant defense coupled with other functions of ErbB2 signaling, and/or fuel usage through glycolysis/gluconeogenesis (again, highly possible due to changes in energy metabolism in other MRD-utilizing species and changes in GO terms involving lipid metabolism). MAPK signaling through ERK may lead to additional functions of DNA damage repair and cellular adhesion, both of which also have GO terms to reinforce their potential involvement (Figure 2, Figure 3, Figure 4 and Figure 5). Furthermore, crosstalk with other forms of regulation including alternative splicing, epigenetic modifications, and post-translational modifications warrants further study given the plethora of GO and KEGG terms implicating miRNA involvement in these functions (Figure 2, Figure 3, Figure 4 and Figure 5).

This study provides novel insight into miRNA regulation during hibernation in *I. tridecemlineatus* cardiac tissue, and it followed the expected themes set forth in our hypothesis. Furthermore, we isolated a potentially critical subset of miRNAs that may be differentially regulated during hibernation as well as four signaling pathways (ErbB, PI3K/Akt, MAPK, and p53) ideal for deeper study. Further investigation will help uncover the critical roles of miRNA during hibernation across all tissues in this animal, providing a comprehensive and valuable model for hibernation research.

## Data Availability

The genome files for *I. tridecemlineatus* have been uploaded to the SRA database (SRA Accession: PRJNA1010398). All Supplementary Data have been uploaded with the manuscript.

## Supplementary Materials

The following supporting information can be downloaded at: https://www.mdpi.com/article/10.3390/metabo13101096/s1, Table S1: miRNAs differential expression; Table S2: miRNA GO biological processes; Table S3: miRNA GO cellular compartment; Table S4: miRNA GO molecular function; Table S5: miRNA KEGG.
