# Supplementary material for: Hibernation-Induced microRNA Expression Promotes Signaling Pathways and Cell Cycle Dysregulation in Ictidomys tridecemlineatus Cardiac Tissue
Source: Metabolites. 2023 Oct 19;13(10):1096. doi: 10.3390/metabo13101096 (PMC10608741; doi:10.3390/metabo13101096)
Supplement: Supplementary file 1 [file metabolites-13-01096-s001.zip › metabolites-2612037-supplementary.pdf]

Table S1. miRNAs differential expression.

MicroRNA	Log FC	Average Expression	p-value
let-7b-5p	-0.257767565	9.590098599	0.786188028
let-7d-3p	1.143899591	8.835562853	0.178495926
let-7e-5p	2.01728148	8.918097648	0.011511287
miR-1-3p	0.236863137	8.771163125	0.733937209
miR-10527-5p	0.650831278	11.00779943	0.482423314
miR-12135	0.284835294	9.79232893	0.772316247
miR-122-5p	2.068984293	9.030464054	0.040497256
miR-1247-5p	0.44794322	8.365218213	0.367052683
miR-1260b	0.975807489	12.15544915	0.165156848
miR-1301-3p	-1.04637832	13.61008464	0.147164728
miR-1323	2.314613299	12.47942818	0.237468876
miR-1343-3p	-0.761378133	16.15781987	0.427930901
miR-1343-5p	1.235509718	8.69103246	0.124301392
miR-181d-3p	-0.318192262	8.89642668	0.533309385
miR-181d-5p	-0.214402767	11.9283733	0.772966579
miR-18a-3p	-1.779454691	9.942831982	0.105986128
miR-1973	0.447601849	9.82355904	0.589481077
miR-208a-5p	0.065272778	8.612857599	0.927275068
miR-2114-5p	2.355745141	12.31749217	0.213979363
miR-221-3p	0.673837234	8.227059822	0.145217306
miR-2355-3p	-2.063005204	9.488152162	0.015353623
miR-23b-3p	0.615898489	8.228192794	0.177043907
miR-23c	0.702380433	8.508031262	0.161539061
miR-24-3p	0.808660928	8.64860479	0.361660836
miR-26a-5p	0.279824751	10.11404847	0.700227575
miR-27a-3p	-0.491017148	8.415111781	0.359041941
miR-27b-3p	-0.435545339	8.98424124	0.533977786
miR-3064-5p	0.910641912	8.732741792	0.280916133
miR-3074-5p	1.077443098	8.82675008	0.184744521
miR-30a-3p	-0.660609542	16.70819256	0.337206309
miR-30a-5p	-0.648330312	8.254272551	0.204230889
miR-30b-3p	-0.59670685	13.56310586	0.336341377
miR-30c-1-3p	-0.135022501	13.51796779	0.828600474
miR-30c-2-3p	-0.315064839	16.34869336	0.617709676
miR-30d-3p	1.0674587	11.09961909	0.239852043
miR-30e-3p	-1.072803706	15.90652981	0.173564996
miR-32-3p	0.949969462	8.623658006	0.256566636
miR-3613-3p	0.150152735	11.39523213	0.858551927
miR-3613-5p	-0.23520085	13.22417841	0.792874549
miR-3671	-0.542323231	8.742694655	0.28516143
miR-376c-3p	0.278637965	8.766562943	0.681213857
miR-378a-3p	-0.102902201	11.85950382	0.872992618
miR-378c	-0.147774263	11.55598111	0.843773533
miR-378i	-1.667304842	8.789656105	0.024855717
miR-421	0.604726575	13.9671473	0.451063385
miR-425-3p	0.372432097	9.764943723	0.668167593
miR-4454	0.578370887	11.70914105	0.590196076
miR-4484	-1.217522006	9.316686715	0.286714833
miR-4485-3p	0.023955197	10.52703801	0.978893422

miR-4488	2.117544273	10.04127104	0.216044322
miR-454-3p	-2.202353153	9.277720574	0.03554327
miR-4695-3p	0.597284192	8.427523947	0.193507196
miR-483-5p	1.032159453	9.992445746	0.291445245
miR-486-5p	-0.023628752	8.441727157	0.967237738
miR-493-3p	-1.601720573	12.13938222	0.062398439
miR-493-5p	-0.579272167	14.75937321	0.41320258
miR-503-5p	0.615898489	8.228192794	0.177043907
miR-504-5p	0.075194964	9.014646294	0.91734928
miR-5100	0.408305956	10.86649269	0.680097898
miR-512-3p	-0.13612152	8.397206268	0.804463915
miR-515-3p	1.697248663	10.81967378	0.295461665
miR-515-5p	-0.308439573	9.114614648	0.750864788
miR-517a-3p	0.08148892	9.685051367	0.933622221
miR-517b-3p	0.08148892	9.685051367	0.933622221
miR-518c-3p	-0.574959703	9.620373091	0.565468736
miR-524-5p	0.384825112	9.133264293	0.616878294
miR-6529-5p	0.01850486	16.14942946	0.976297297
miR-663b	0.530179655	8.610435908	0.346120637
miR-6715b-3p	-1.785596654	8.92608118	0.024697274
miR-769-3p	0.17909617	12.95901879	0.807924222
miR-769-5p	-0.092104569	15.93849867	0.89259804
miR-7704	0.612339045	12.72541658	0.394217322
miR-92b-5p	0.912742064	8.60008553	0.283031217
miR-93-3p	-0.950567827	11.44550153	0.210670596
miR-93-5p	-0.456602	12.11237832	0.512869265
miR-935	-0.782858727	10.73810529	0.459692819
miR-95-3p	0.520872354	16.64424777	0.455730214
miR-9851-3p	-1.965001558	10.34341884	0.025475125

Table S2. miRNA GO biological processes.

GO Term	Genes Tested	Model Coefficient	p-value
POSITIVE REGULATION OF VIRAL TRANSCRIPTION	39	-0.078311167	0.542300703
CARDIAC CHAMBER DEVELOPMENT	130	0.057699495	0.42844073
DNA DEPENDENT DNA REPLICATION MAINTENANCE OF FIDELITY	23	-0.235053966	0.132979454
CIRCADIAN RHYTHM	118	0.013507728	0.858591625
PHOSPHATIDYLSERINE ACYL CHAIN REMODELING	12	0.179057287	0.45624813
SPINAL CORD DEVELOPMENT	84	0.071855289	0.42809703
PLATELET DERIVED GROWTH FACTOR RECEPTOR SIGNALING PATHWAY	32	-0.057945153	0.685071577
CELLULAR RESPONSE TO LIPOPROTEIN PARTICLE STIMULUS	12	-0.255788847	0.234913356
REGULATION OF NLRP3 INFLAMMASOME COMPLEX ASSEMBLY	6	0.262823783	0.433000517
POSITIVE REGULATION OF EPITHELIAL CELL DIFFERENTIATION	46	0.108382648	0.377759208
POSITIVE REGULATION OF KINASE ACTIVITY	433	-0.044396152	0.261101665
NEGATIVE REGULATION OF TRANSCRIPTION FACTOR IMPORT INTO NUCLEUS	37	0.12893907	0.348226826
POTASSIUM ION TRANSPORT	105	0.13447175	0.099276899
REGULATION OF T CELL RECEPTOR SIGNALING PATHWAY	26	-0.068532963	0.664306907
CARDIAC MUSCLE ADAPTATION	8	0.297783911	0.299694675
NEGATIVE REGULATION OF EPITHELIAL CELL PROLIFERATION	98	-0.037815854	0.645424414
MOVEMENT IN ENVIRONMENT OF OTHER ORGANISM INVOLVED IN SYMBIOTIC INTERACTION	82	0.026532124	0.770793028
REGULATION OF PROTEIN TARGETING TO MITOCHONDRION	80	-0.11218016	0.207483997
APICAL PROTEIN LOCALIZATION	12	0.254363537	0.296966815
REGULATION OF ESTABLISHMENT OF PLANAR POLARITY	103	-0.085615085	0.279517133
FOREBRAIN NEURON DEVELOPMENT	30	0.104773337	0.491039555
POSITIVE REGULATION OF PROTEIN MATURATION	15	0.050870229	0.81155953
NEUROMUSCULAR JUNCTION DEVELOPMENT	30	-0.153835591	0.282168217
MITOTIC CYTOKINESIS	28	-0.212044227	0.142381845

NEGATIVE REGULATION OF RESPONSE TO ENDOPLASMIC RETICULUM STRESS	39	-0.234186489	0.054504564
SMAD PROTEIN SIGNAL TRANSDUCTION	43	0.106755473	0.400867307
CYTOPLASMIC TRANSLATION	40	-0.36843619	0.001488424
MEIOTIC CHROMOSOME SEGREGATION	52	0.005427439	0.961950314
POSITIVE REGULATION OF CALCIUM ION TRANSPORT	89	-0.200260704	0.014627842
REGULATION OF DOUBLE STRAND BREAK REPAIR	35	0.082524992	0.556831078
RNA DEPENDENT DNA BIOSYNTHETIC PROCESS	20	-0.185864081	0.286705202
REGULATION OF B CELL RECEPTOR SIGNALING PATHWAY	15	0.16088039	0.460323245
REGULATION OF G PROTEIN COUPLED RECEPTOR PROTEIN SIGNALING PATHWAY	101	0.002387189	0.976693403
DENDRITE DEVELOPMENT	65	-0.108377042	0.273369452
REGULATION OF RESPIRATORY BURST	10	-0.173534853	0.481341451
G PROTEIN COUPLED RECEPTOR INTERNALIZATION	10	0.192660528	0.461702672
MEMORY	81	-0.08898947	0.317741739
NEURON DEVELOPMENT	595	0.03707889	0.281925637
REGULATION OF GOLGI ORGANIZATION	12	0.451220435	0.040308472
ENDOTHELIAL CELL DEVELOPMENT	42	-0.099273389	0.421567212
POSITIVE REGULATION OF MYOTUBE DIFFERENTIATION	25	-0.0628827	0.69634427
REGULATION OF CELL ACTIVATION	396	-0.060450196	0.141261981
MULTICELLULAR ORGANISM AGING	28	-0.266639248	0.063193699
ATP DEPENDENT CHROMATIN REMODELING	71	-0.212435948	0.020068117
ANATOMICAL STRUCTURE FORMATION INVOLVED IN MORPHOGENESIS	840	0.010938871	0.707032184
ION TRANSPORT	992	0.028452675	0.292167114
LIPID MODIFICATION	186	0.144811647	0.018599829
NEGATIVE REGULATION OF KIDNEY DEVELOPMENT	16	0.028773594	0.888809684
REGULATION OF HEPATOCYTE PROLIFERATION	11	-0.278686397	0.218909841
ACYLGLYCEROL HOMEOSTASIS	20	0.312629115	0.086764417
NEGATIVE REGULATION OF PHOSPHOPROTEIN PHOSPHATASE ACTIVITY	15	0.09937388	0.643424827
NEGATIVE REGULATION OF PROTEIN COMPLEX ASSEMBLY	94	0.095542051	0.266397227
HEMOGLOBIN METABOLIC PROCESS	6	0.00555061	0.986757588
SINGLE FERTILIZATION	73	-0.048077741	0.612355053
LEUKOCYTE ACTIVATION	349	-0.074206543	0.08839381
CHROMOSOME ORGANIZATION	909	-0.033315988	0.230883026
REGULATION OF MITOCHONDRIAL DEPOLARIZATION	14	-0.026226155	0.903966695
NEGATIVE REGULATION OF MAP KINASE ACTIVITY	68	-0.149129106	0.119001259
SYNAPTIC VESICLE LOCALIZATION	88	0.002102372	0.980830293
PHENOL CONTAINING COMPOUND METABOLIC PROCESS	60	0.098406665	0.35958199
REGULATION OF ANTIGEN RECEPTOR MEDIATED SIGNALING PATHWAY	39	-0.001843673	0.98878022
ESTABLISHMENT OF SPINDLE ORIENTATION	25	-0.105128757	0.509697617
METANEPHRIC NEPHRON MORPHOGENESIS	17	-0.02505975	0.898966762
GLYCERALDEHYDE 3 PHOSPHATE METABOLIC PROCESS	18	-0.006414004	0.973441006
RESPONSE TO UV C	10	0.376366962	0.140582762
CARBOHYDRATE CATABOLIC PROCESS	94	-0.009160695	0.913636453
DENTATE GYRUS DEVELOPMENT	11	0.39240615	0.111871932
ESTABLISHMENT OR MAINTENANCE OF TRANSMEMBRANE ELECTROCHEMICAL GRADIENT	10	0.241481548	0.362138269
LABYRINTHINE LAYER DEVELOPMENT	42	0.448447933	0.000164397
N TERMINAL PROTEIN AMINO ACID ACETYLATION	14	-0.227969182	0.259749971
NEUROLOGICAL SYSTEM PROCESS	655	0.073898681	0.025561538
NEURONAL STEM CELL DIVISION	13	0.228937698	0.3199391
CERAMIDE CATABOLIC PROCESS	8	0.188602106	0.519615371
EXCRETION	29	0.163670212	0.291791846
DIGESTION	89	0.189263187	0.033132707
POSITIVE REGULATION OF SYNAPTIC TRANSMISSION GLUTAMATERGIC	15	-0.233768867	0.227969806
POSITIVE REGULATION OF MITOTIC NUCLEAR DIVISION	46	-0.1267384	0.277991235
HINDBRAIN DEVELOPMENT	120	-0.028949187	0.697533308
OLIGOSACCHARIDE METABOLIC PROCESS	54	-0.087282723	0.423394462
NEGATIVE REGULATION OF DENDRITIC SPINE DEVELOPMENT	10	-0.540609144	0.010450219
MODULATION OF GROWTH OF SYMBIONT INVOLVED IN INTERACTION WITH HOST	11	0.333397189	0.17198357
CHROMOSOME SEGREGATION	246	-0.140447199	0.005645596
NEGATIVE REGULATION OF CYCLIN DEPENDENT PROTEIN KINASE ACTIVITY	28	-0.312639318	0.025083924
ETHER METABOLIC PROCESS	9	0.093970273	0.734332468
HEMATOPOIETIC PROGENITOR CELL DIFFERENTIATION	91	-0.07110088	0.400364692
CYTOKINE METABOLIC PROCESS	14	0.421573272	0.050119478
ANTEROGRADE AXONAL TRANSPORT	25	-0.024825343	0.878823299

NEGATIVE REGULATION OF ERK1 AND ERK2 CASCADE	48	-0.048407842	0.678924551
STEROID BIOSYNTHETIC PROCESS	88	-0.013393896	0.877921536
ALDITOL PHOSPHATE METABOLIC PROCESS	28	0.097297528	0.535743578
SODIUM INDEPENDENT ORGANIC ANION TRANSPORT	14	-0.148057853	0.478422478
REGULATION OF PROTEIN TYROSINE KINASE ACTIVITY	55	0.07381894	0.510029488
REGULATION OF INTERFERON BETA PRODUCTION	39	-0.220728753	0.072500171
DNA TEMPLATED TRANSCRIPTION ELONGATION	92	-0.05618866	0.505561448
N GLYCAN PROCESSING	17	0.072605712	0.718146743
INORGANIC ION IMPORT INTO CELL	14	0.289022572	0.194008893
ENDOPLASMIC RETICULUM TO CYTOSOL TRANSPORT	23	-0.319614345	0.045597275
NEGATIVE REGULATION OF REPRODUCTIVE PROCESS	41	0.045833666	0.722454033
G PROTEIN COUPLED PURINERGIC RECEPTOR SIGNALING PATHWAY	15	-0.005388937	0.979630961
MEMBRANE DEPOLARIZATION	51	0.023317476	0.83971486
OLFACTORY BULB INTERNEURON DIFFERENTIATION	13	0.008677934	0.969558943
POSITIVE REGULATION OF G PROTEIN COUPLED RECEPTOR PROTEIN SIGNALING PATHWAY	19	0.114749424	0.547965083
ATTACHMENT OF SPINDLE MICROTUBULES TO KINETOCHORE	13	-0.682106393	7.03E-05
REGULATION OF LYASE ACTIVITY	59	-0.051867129	0.622499068
RESPONSE TO NITROGEN COMPOUND	732	-0.049499912	0.106632214
NEGATIVE REGULATION OF TRANSMEMBRANE TRANSPORT	74	-0.042031779	0.656130919
REGULATION OF DNA RECOMBINATION	53	0.067870893	0.551614299
REGULATION OF LEUKOCYTE PROLIFERATION	173	-0.091219721	0.135815539
RESPONSE TO ETHANOL	113	0.012989618	0.866809389
SULFUR COMPOUND METABOLIC PROCESS	302	-0.05640418	0.2294525
POSITIVE REGULATION OF TYROSINE PHOSPHORYLATION OF STAT1 PROTEIN	10	0.412506892	0.114889226
REGULATION OF CELL CYCLE CHECKPOINT	28	0.290552808	0.067855148
NEGATIVE REGULATION OF TRANSCRIPTION REGULATORY REGION DNA BINDING	16	0.129228721	0.533796852
POSITIVE REGULATION OF DNA REPLICATION	76	0.032521277	0.731288918
NEGATIVE REGULATION OF NEURON APOPTOTIC PROCESS	125	-0.016668574	0.819868032
POSITIVE REGULATION OF REPRODUCTIVE PROCESS	42	0.146699994	0.25562387
POSITIVE REGULATION OF METANEPHROS DEVELOPMENT	12	0.041692403	0.861050538
POSITIVE REGULATION OF LYMPHOCYTE APOPTOTIC PROCESS	16	-0.191336582	0.319547789
NUCLEOTIDE PHOSPHORYLATION	52	0.06292405	0.584049023
POSITIVE REGULATION OF GENE EXPRESSION EPIGENETIC	75	0.004040381	0.965995458
GLUTAMATE SECRETION	26	-0.142095672	0.357738937
REGULATION OF MRNA METABOLIC PROCESS	109	-0.032759618	0.674723653
POSITIVE REGULATION OF PROTEIN BINDING	71	-0.037889046	0.694497368
REGULATION OF NFAT PROTEIN IMPORT INTO NUCLEUS	16	-0.033203898	0.87008196
REGULATION OF VASCULOGENESIS	13	-0.157880943	0.464149704
LENS FIBER CELL DIFFERENTIATION	19	0.067985272	0.720473423
SENSORY PERCEPTION	402	0.106950704	0.010937202
HEPATICOBILIARY SYSTEM DEVELOPMENT	114	0.004576885	0.952589917
FATTY ACID CATABOLIC PROCESS	64	0.110390385	0.288687569
REGULATION OF VESICLE FUSION	52	0.07506254	0.514331289
MITOCHONDRIAL RESPIRATORY CHAIN COMPLEX I BIOGENESIS	55	-0.095215326	0.376584694
REGULATION OF LIPID STORAGE	36	0.086579628	0.532287598
CARBOHYDRATE TRANSPORT	71	-0.077800337	0.414690172
REGULATION OF CELLULAR RESPONSE TO GROWTH FACTOR STIMULUS	207	0.078916841	0.174169395
EMBRYONIC HEMOPOIESIS	16	-0.224074386	0.240143108
EMBRYO DEVELOPMENT ENDING IN BIRTH OR EGG HATCHING	507	0.010823261	0.770103184
ANTIGEN PROCESSING AND PRESENTATION OF PEPTIDE OR POLYSACCHARIDE ANTIGEN VIA MHC CLASS II	85	-0.024401929	0.782733073
RESPONSE TO IONIZING RADIATION	135	-0.084655441	0.221519831
PROTEIN EXPORT FROM NUCLEUS	29	0.299146503	0.060403964
EPITHELIUM DEVELOPMENT	797	0.036165251	0.227621469
POSITIVE REGULATION OF SEQUESTERING OF CALCIUM ION	16	-0.118407793	0.548745233
SECONDARY METABOLITE BIOSYNTHETIC PROCESS	15	0.135030134	0.528449107
CELL MIGRATION INVOLVED IN HEART DEVELOPMENT	13	-0.175277919	0.41400484
PROTEIN LOCALIZATION TO CYTOSKELETON	28	-0.00221083	0.988594981
NUCLEAR ENVELOPE REASSEMBLY	17	-0.35148138	0.051666006
INTRINSIC APOPTOTIC SIGNALING PATHWAY IN RESPONSE TO DNA DAMAGE BY P53 CLASS MEDIATOR	29	-0.265382949	0.053403264
REGULATION OF AUTOPHAGY	230	-0.038658712	0.473139912
PHOSPHOLIPID METABOLIC PROCESS	313	0.010615669	0.820652669
AMMONIUM ION METABOLIC PROCESS	133	0.022935976	0.748629165

REGULATION OF INTERLEUKIN 4 PRODUCTION	21	0.31172669	0.090436044
NCRNA CATABOLIC PROCESS	20	-0.052701638	0.7705869
REGULATION OF FAT CELL DIFFERENTIATION	96	0.004211787	0.959930274
GLUTAMINE FAMILY AMINO ACID BIOSYNTHETIC PROCESS	18	-0.084464372	0.654921921
REGULATION OF PROTEIN ACTIVATION CASCADE	26	0.241882571	0.135553694
REGULATION OF TELOMERASE RNA LOCALIZATION TO CAJAL BODY	15	-0.109867544	0.592939234
REGULATION OF RNA STABILITY	132	-0.064829775	0.357141508
BUNDLE OF HIS CELL TO PURKINJE MYOCYTE COMMUNICATION	10	0.060962919	0.815875106
REGULATION OF CELL MATURATION	15	-0.188048476	0.347211357
NEGATIVE REGULATION OF CELL DEVELOPMENT	262	-0.011548176	0.820364091
MAMMARY GLAND MORPHOGENESIS	33	-0.046229653	0.74294239
CELLULAR PROTEIN COMPLEX DISASSEMBLY	114	-0.161086499	0.0281719
REGULATION OF CYTOKINE PRODUCTION INVOLVED IN INFLAMMATORY RESPONSE	14	0.337445667	0.112215765
URETER DEVELOPMENT	10	-0.238456867	0.317509872
BODY MORPHOGENESIS	38	0.016743466	0.900069737
SOMATIC STEM CELL POPULATION MAINTENANCE	62	0.11336615	0.285179035
CYTOPLASMIC MICROTUBULE ORGANIZATION	35	-0.062213515	0.648249952
POSITIVE REGULATION OF DNA DAMAGE RESPONSE SIGNAL TRANSDUCTION BY P53 CLASS MEDIATOR	13	-0.075639464	0.733999785
PROTEIN ACTIVATION CASCADE	49	0.274298897	0.019105628
POSITIVE REGULATION OF PHOSPHOLIPASE ACTIVITY	41	-0.116552927	0.348243753
NEGATIVE REGULATION OF PEPTIDYL TYROSINE PHOSPHORYLATION	36	-0.013675377	0.91995289
REGULATION OF COLLATERAL SPROUTING	17	0.288492997	0.14961819
ESTABLISHMENT OR MAINTENANCE OF MONOPOLAR CELL POLARITY	15	-0.156214794	0.443112461
NEURON RECOGNITION	27	0.077171774	0.6286033
NUCLEOSIDE DIPHOSPHATE METABOLIC PROCESS	74	0.038259281	0.690351163
MICROTUBULE POLYMERIZATION OR DEPOLYMERIZATION	34	0.107959367	0.450154584
RESPONSE TO AMMONIUM ION	44	-0.148230507	0.211476703
REGULATION OF INTERLEUKIN 5 PRODUCTION	11	0.426505006	0.064148985
REGULATION OF GLUCOSE IMPORT	52	-0.035467025	0.753031523
NEGATIVE REGULATION OF PROTEOLYSIS	265	-0.013642331	0.787249267
PINOCYTOSIS	10	0.259076983	0.324604114
REGULATION OF EPITHELIAL CELL APOPTOTIC PROCESS	51	-0.175247062	0.108036769
PHOSPHATIDYLETHANOLAMINE METABOLIC PROCESS	16	-0.280272767	0.132751017
PHOSPHATIDYLCHOLINE METABOLIC PROCESS	46	0.05587587	0.647106941
LUNG EPITHELIUM DEVELOPMENT	31	0.192717486	0.199178671
METENCEPHALON DEVELOPMENT	85	-0.054350218	0.536107675
G PROTEIN COUPLED PURINERGIC NUCLEOTIDE RECEPTOR SIGNALING PATHWAY	12	0.041869325	0.860422894
ACTIN FILAMENT BUNDLE ORGANIZATION	43	-0.068861997	0.575074363
CELLULAR RESPONSE TO VIRUS	21	-0.059943626	0.733408044
CYTOPLASMIC MRNA PROCESSING BODY ASSEMBLY	12	-0.305908231	0.15360164
RESPIRATORY GASEOUS EXCHANGE	40	-0.006107853	0.962335453
NEGATIVE REGULATION OF PROTEIN MODIFICATION BY SMALL PROTEIN CONJUGATION OR REMOVAL	129	-0.114969281	0.101608667
AMINO ACID ACTIVATION	49	-0.252674929	0.021242994
DENDRITIC CELL DIFFERENTIATION	29	-0.090920847	0.541251735
NATURAL KILLER CELL DIFFERENTIATION	10	-0.41998809	0.049978619
BONE TRABECULA MORPHOGENESIS	11	0.005177874	0.98327572
REGULATION OF INFLAMMATORY RESPONSE TO ANTIGENIC STIMULUS	16	-0.056658848	0.778807104
REGULATION OF PROTEASOMAL UBIQUITIN DEPENDENT PROTEIN CATABOLIC PROCESS	143	-0.044787763	0.510375007
NEGATIVE REGULATION OF NEURON DIFFERENTIATION	166	-0.015470854	0.807888927
REGULATION OF SKELETAL MUSCLE TISSUE DEVELOPMENT	44	0.221234076	0.079352488
MUSCLE TISSUE DEVELOPMENT	238	-0.019023945	0.720783778
NEGATIVE REGULATION OF PROTEIN AUTOPHOSPHORYLATION	9	0.074807778	0.78646277
ACID SECRETION	54	-0.047779772	0.664696295
POSITIVE REGULATION OF ADAPTIVE IMMUNE RESPONSE	63	-0.098009089	0.330593364
REGULATION OF OSTEOCLAST DIFFERENTIATION	55	-0.075798131	0.484184096
REGULATION OF PROTEIN BINDING	158	0.020608023	0.753771789
CARDIAC SEPTUM DEVELOPMENT	79	-0.071100215	0.43322173
REGULATION OF ICOSANOID SECRETION	15	0.029169951	0.890821349
L ALPHA AMINO ACID TRANSMEMBRANE TRANSPORT	30	0.035087998	0.815687599
REGULATION OF HISTONE METHYLATION	51	-0.12760266	0.251257851
POSITIVE REGULATION OF MITOCHONDRIAL FISSION	13	-0.116557718	0.595645596

ANTIMICROBIAL HUMORAL RESPONSE	32	-0.109945068	0.433528945
EPIDERMIS MORPHOGENESIS	26	0.13323672	0.415076145
INTERMEDIATE FILAMENT BASED PROCESS	36	0.061230901	0.65749014
COGNITION	214	-0.027574313	0.622278975
RESPONSE TO ZINC ION	46	0.04405059	0.717552413
PURINE DEOXYRIBONUCLEOTIDE METABOLIC PROCESS	14	0.131461438	0.554466813
DISRUPTION OF CELLS OF OTHER ORGANISM	19	0.280792575	0.138931132
AXIS SPECIFICATION	76	-0.03655458	0.695149699
PHOSPHATIDYLINOSITOL METABOLIC PROCESS	168	0.066355296	0.30154346
CELL PART MORPHOGENESIS	550	0.071704764	0.046345322
PIGMENT GRANULE LOCALIZATION	23	0.073024746	0.672362455
VASCULAR ENDOTHELIAL GROWTH FACTOR SIGNALING PATHWAY	14	0.313378829	0.147800497
RESPONSE TO ORGANOPHOSPHORUS	117	-0.070133768	0.347215049
AXO DENDRITIC TRANSPORT	34	-0.01650717	0.906097989
SINGLE ORGANISM MEMBRANE FUSION	104	0.016382175	0.8392652
REGULATION OF PROTEIN UBIQUITINATION INVOLVED IN UBIQUITIN DEPENDENT PROTEIN CATABOLIC PROCESS	95	-0.152582975	0.058562661
SKELETAL MUSCLE TISSUE REGENERATION	22	0.084543549	0.633338891
POLYSACCHARIDE BIOSYNTHETIC PROCESS	40	0.051552858	0.693434863
REGULATION OF TYROSINE PHOSPHORYLATION OF STAT PROTEIN	58	-0.094277765	0.369971223
ORGAN REGENERATION	76	-0.122514331	0.179111377
GLAND MORPHOGENESIS	82	-0.056085867	0.530371872
NEGATIVE REGULATION OF ADAPTIVE IMMUNE RESPONSE	28	0.155206144	0.324614918
REGULATION OF SYSTEMIC ARTERIAL BLOOD PRESSURE BY CIRCULATORY RENIN ANGIOTENSIN	10	0.099698962	0.704122541
RIBOSOME BIOGENESIS	298	-0.092980647	0.047146256
B CELL HOMEOSTASIS	20	-0.274115395	0.112728877
IRE1 MEDIATED UNFOLDED PROTEIN RESPONSE	53	-0.103812193	0.343363637
SINGLE ORGANISM BEHAVIOR	315	0.019065148	0.683460124
POSITIVE REGULATION OF CALCIUM ION DEPENDENT EXOCYTOSIS	20	-0.241107886	0.154150032
POSITIVE REGULATION OF ENDOCYTOSIS	96	0.096080457	0.258926932
MRNA SPLICE SITE SELECTION	25	-0.372546746	0.011038711
REGULATION OF TRANSLATION IN RESPONSE TO STRESS	19	-0.182123432	0.305187118
NEGATIVE REGULATION OF GENE EXPRESSION	1339	-0.004313157	0.853755165
PHENOL CONTAINING COMPOUND BIOSYNTHETIC PROCESS	25	0.100298424	0.545697276
POSITIVE REGULATION OF ERYTHROCYTE DIFFERENTIATION	22	-0.056043593	0.744719193
DENDRITIC CELL MIGRATION	18	0.196675783	0.313915347
SENSORY PERCEPTION OF TEMPERATURE STIMULUS	15	0.351189727	0.091723541
LYSOSOMAL TRANSPORT	65	0.090032581	0.384249256
CELLULAR CATABOLIC PROCESS	1156	-0.0338313	0.173898
POSITIVE REGULATION OF DNA BIOSYNTHETIC PROCESS	56	0.023224874	0.832848562
POSITIVE REGULATION OF MACROPHAGE ACTIVATION	8	-0.080264438	0.776818332
REGULATION OF CALCIUM MEDIATED SIGNALING	62	-0.1635793	0.098640136
REGULATION OF ALPHA BETA T CELL ACTIVATION	59	0.103669558	0.33987761
MUSCLE ORGAN DEVELOPMENT	235	0.004452648	0.934079717
POSITIVE REGULATION OF HEMOPOIESIS	145	-0.018839849	0.781662858
OUTFLOW TRACT SEPTUM MORPHOGENESIS	12	0.245980097	0.302320476
REGULATION OF MAMMARY GLAND EPITHELIAL CELL PROLIFERATION	16	0.078154855	0.707072158
REGULATION OF PHOSPHATIDYLINOSITOL 3 KINASE ACTIVITY	38	-0.214019788	0.084690047
CELLULAR RESPONSE TO EPIDERMAL GROWTH FACTOR STIMULUS	24	-0.315152121	0.037041955
LEUKOCYTE DEGRANULATION	25	-0.21068778	0.169121382
MYELOID CELL HOMEOSTASIS	78	-0.225433695	0.010133108
REGULATION OF BLOOD PRESSURE	135	0.109932077	0.126062412
REGULATION OF MORPHOGENESIS OF A BRANCHING STRUCTURE	48	-0.171127167	0.126938008
RESPONSE TO ELECTRICAL STIMULUS	34	-0.135864574	0.313773473
NEGATIVE REGULATION OF TELOMERE MAINTENANCE VIA TELOMERASE	12	-0.534192977	0.005239507
REGULATION OF T CELL CHEMOTAXIS	10	-0.576301	0.012588569
REGULATION OF ESTABLISHMENT OF PLANAR POLARITY INVOLVED IN NEURAL TUBE CLOSURE	13	-0.112249357	0.609446974
REGULATION OF CYTOKINE PRODUCTION	473	-0.019395371	0.61034399
REGULATION OF ERBB SIGNALING PATHWAY	77	-0.001409425	0.987964522
REGULATION OF CHOLESTEROL HOMEOSTASIS	11	-0.214252188	0.366461462
VENTRICULAR SEPTUM MORPHOGENESIS	25	0.124230438	0.455861631
POSITIVE REGULATION OF ENDOTHELIAL CELL CHEMOTAXIS	11	0.332182611	0.178194584
POSITIVE REGULATION OF ENDOTHELIAL CELL DIFFERENTIATION	12	0.035886723	0.880102872
NEGATIVE REGULATION OF SMOOTH MUSCLE CELL PROLIFERATION	34	-0.004607811	0.973801605

TOLERANCE INDUCTION	11	-0.368890656	0.099784819
CHEMICAL HOMEOSTASIS	707	0.046777039	0.141086785
SYNAPSE ORGANIZATION	126	-0.091043104	0.202742818
METANEPHROS DEVELOPMENT	71	0.152576823	0.123964461
REGULATION OF MICROTUBULE BASED PROCESS	218	0.021738537	0.698160293
NEGATIVE REGULATION OF TRANSPORTER ACTIVITY	57	-0.091206274	0.389956589
ENDOSOME ORGANIZATION	59	0.090095511	0.406299863
REGULATION OF WOUND HEALING	111	0.04924759	0.531216354
REGULATION OF MAPK CASCADE	563	-0.00378197	0.914219701
DEVELOPMENTAL PROGRAMMED CELL DEATH	24	0.289378517	0.086331639
POSITIVE REGULATION OF SKELETAL MUSCLE TISSUE DEVELOPMENT	24	0.245947798	0.147970084
MYELOID LEUKOCYTE MIGRATION	79	-0.018275763	0.842361225
COLLAGEN FIBRIL ORGANIZATION	34	0.315874773	0.026235676
POSITIVE REGULATION OF NEURON DEATH	59	0.057171324	0.596215435
RESPONSE TO ESTRADIOL	133	-0.021351543	0.763332814
CORONARY VASCULATURE DEVELOPMENT	33	-0.068296655	0.626317966
CARTILAGE DEVELOPMENT	123	0.114962403	0.127406867
REGULATION OF TUMOR NECROSIS FACTOR SUPERFAMILY CYTOKINE PRODUCTION	86	-0.025898543	0.768384931
NEURON PROJECTION EXTENSION	47	0.003481194	0.976778771
REGULATION OF CARDIAC MUSCLE TISSUE DEVELOPMENT	45	0.092863888	0.453766734
NEGATIVE REGULATION OF CYCLIC NUCLEOTIDE METABOLIC PROCESS	32	-0.047724692	0.738685769
PYRIMIDINE NUCLEOTIDE BIOSYNTHETIC PROCESS	28	-0.223873072	0.121616865
RESPONSE TO MUSCLE STRETCH	15	-0.1593907	0.428835949
NEGATIVE REGULATION OF OLIGODENDROCYTE DIFFERENTIATION	12	-0.313280324	0.134407355
REGULATION OF PHOSPHATIDYLINOSITOL 3 KINASE SIGNALING	125	0.131995811	0.07786106
RESPONSE TO COLD	37	-0.028054617	0.833878895
NEGATIVE REGULATION OF SODIUM ION TRANSPORT	11	0.202057337	0.42679625
CELLULAR RESPONSE TO INTERLEUKIN 1	72	-0.01568783	0.870587078
CIRCULATORY SYSTEM PROCESS	298	0.029875621	0.534864775
REGULATION OF VASODILATION	38	0.083682151	0.534313744
REGULATION OF TRIGLYCERIDE METABOLIC PROCESS	29	0.093108534	0.546412783
NEGATIVE REGULATION OF GLYCOPROTEIN BIOSYNTHETIC PROCESS	11	0.22828217	0.365980953
RESPONSE TO COCAINE	39	0.047651687	0.718805592
CELL CHEMOTAXIS	138	0.046408087	0.51068099
NEGATIVE REGULATION OF INTRINSIC APOPTOTIC SIGNALING PATHWAY BY P53 CLASS MEDIATOR	18	0.064005836	0.742844399
RIBONUCLEOTIDE CATABOLIC PROCESS	27	0.12255438	0.444035376
POSITIVE REGULATION OF MITOTIC SISTER CHROMATID SEPARATION	13	-0.198682663	0.349111363
DNA TEMPLATED TRANSCRIPTION TERMINATION	99	-0.137710153	0.082521409
NEGATIVE REGULATION OF INTERLEUKIN 1 PRODUCTION	10	-0.20880465	0.392523823
POSITIVE REGULATION OF CATION CHANNEL ACTIVITY	29	-0.497554192	4.64E-05
REGULATION OF PROSTAGLANDIN SECRETION	11	-0.05959702	0.806152017
REGULATION OF PODOSOME ASSEMBLY	11	-0.403116969	0.064941081
REGULATION OF CELLULAR SENESCENCE	26	0.064432333	0.691832975
CARDIAC LEFT VENTRICLE MORPHOGENESIS	10	0.352603303	0.162050587
NEGATIVE REGULATION OF STRIATED MUSCLE CELL DIFFERENTIATION	22	0.241785529	0.171229868
RESPONSE TO AMINE	40	-0.14664673	0.237122497
REGULATION OF ORGANIC ACID TRANSPORT	44	0.189357697	0.131011459
REGULATION OF DNA DEPENDENT DNA REPLICATION	39	-0.064332527	0.618307039
NEGATIVE REGULATION OF POTASSIUM ION TRANSMEMBRANE TRANSPORTER ACTIVITY	13	-0.12814282	0.561507968
PROTEIN HOMOTETRAMERIZATION	53	-0.04933247	0.657397847
IMMUNE SYSTEM PROCESS	1595	-0.022258833	0.302250156
HEART TRABECULA MORPHOGENESIS	24	0.113950952	0.504919499
RESPIRATORY CHAIN COMPLEX IV ASSEMBLY	17	-0.118291727	0.53792613
VESICLE LOCALIZATION	199	-0.034912826	0.546631369
NITRIC OXIDE METABOLIC PROCESS	14	0.210649951	0.33955216
POSITIVE REGULATION OF LEUKOCYTE APOPTOTIC PROCESS	23	-0.269991376	0.08300374
POSITIVE REGULATION OF CELL FATE COMMITMENT	9	0.42780214	0.092507039
FOREBRAIN MORPHOGENESIS	11	0.057063586	0.818901973
TELOMERE CAPPING	28	0.080149202	0.609548678
PROSTATE GLAND MORPHOGENESIS	21	0.012597626	0.943926992
NEGATIVE REGULATION OF AXON EXTENSION	37	0.153684039	0.26352701
POSITIVE REGULATION OF TYPE I INTERFERON PRODUCTION	66	-0.041739032	0.67622495
REGULATION OF STRESS ACTIVATED PROTEIN KINASE SIGNALING CASCADE	176	-0.01896102	0.75885356

MYELOID CELL DEVELOPMENT	40	-0.251979333	0.035622615
NEGATIVE REGULATION OF KINASE ACTIVITY	225	-0.146090584	0.005799825
REGULATION OF MITOCHONDRIAL MEMBRANE PERMEABILITY INVOLVED IN APOPTOTIC PROCESS	22	0.016729834	0.92389823
INTRACELLULAR PROTEIN TRANSPORT	714	-0.069332115	0.024966552
REGULATION OF HISTONE DEACETYLATION	22	-0.217241006	0.180180152
REGULATION OF PHOSPHATASE ACTIVITY	114	0.067328712	0.387346659
NEUROTRANSMITTER UPTAKE	13	-0.390143292	0.041573273
MICROTUBULE ANCHORING	16	-0.005483894	0.978592908
CELL DIVISION	432	-0.052890052	0.180288165
REGULATION OF MRNA CATABOLIC PROCESS	24	-0.055118971	0.738160357
INTERMEDIATE FILAMENT ORGANIZATION	17	-0.036896538	0.851245877
REGULATION OF INTERLEUKIN 10 PRODUCTION	37	0.205311092	0.135647552
SODIUM ION EXPORT	10	0.130691927	0.620305623
NEGATIVE REGULATION OF CELL DIVISION	54	-0.232323557	0.026437488
CELLULAR RESPONSE TO UV	63	-0.224961396	0.018668774
REGULATION OF NUCLEAR DIVISION	149	-0.05467995	0.410803032
RESPONSE TO ACID CHEMICAL	278	-0.008940851	0.856450807
RESPONSE TO PROSTAGLANDIN E	23	0.440578188	0.00603263
NEGATIVE REGULATION OF RESPONSE TO REACTIVE OXYGEN SPECIES	18	-0.053599873	0.778431456
POSITIVE REGULATION OF EPITHELIAL TO MESENCHYMAL TRANSITION	31	0.10813811	0.470589994
RESPONSE TO STEROID HORMONE	423	0.041476062	0.307784968
POSITIVE REGULATION OF RNA SPLICING	24	-0.154872165	0.332851022
RESPONSE TO IMMOBILIZATION STRESS	18	-0.042820517	0.822512013
RESPONSE TO INTERLEUKIN 4	29	0.129519012	0.402235275
POSITIVE REGULATION OF KERATINOCYTE DIFFERENTIATION	12	-0.044130214	0.850261422
CARDIAC CHAMBER MORPHOGENESIS	91	0.142617374	0.104113537
REGULATION OF CARTILAGE DEVELOPMENT	54	-0.110253236	0.307773434
POSITIVE REGULATION OF INTERLEUKIN 8 PRODUCTION	35	-0.002705146	0.984400154
SEROTONIN METABOLIC PROCESS	10	-0.07177102	0.777596975
REGULATION OF DEFENSE RESPONSE TO VIRUS	167	-0.114853616	0.063020353
RESPONSE TO PEPTIDE	350	0.022510334	0.612477593
REGULATION OF ENDOTHELIAL CELL APOPTOTIC PROCESS	36	-0.106356091	0.422784591
DNA DEPENDENT DNA REPLICATION	96	-0.094123897	0.249466177
REGULATION OF SKELETAL MUSCLE CELL DIFFERENTIATION	13	0.326194038	0.161390734
POSITIVE REGULATION OF CELLULAR PROTEIN CATABOLIC PROCESS	180	-0.101299533	0.090590298
STEROL HOMEOSTASIS	45	0.271539319	0.02935268
EMBRYONIC AXIS SPECIFICATION	27	-0.101815099	0.507060381
REGULATION OF LEUKOCYTE APOPTOTIC PROCESS	68	-0.143907239	0.131911452
POSITIVE REGULATION OF INTERLEUKIN 1 BETA PRODUCTION	23	0.087652387	0.612219382
CELLULAR RESPONSE TO LIPID	403	0.082388903	0.049330745
SECRETION BY CELL	399	-0.030946688	0.452858425
LIPOPROTEIN METABOLIC PROCESS	105	-0.031275761	0.6941518
DISRUPTION OF CELLS OF OTHER ORGANISM INVOLVED IN SYMBIOTIC INTERACTION	10	0.20401084	0.441954992
IRON COORDINATION ENTITY TRANSPORT	11	0.235775506	0.346950871
PHOSPHATIDYLCHOLINE BIOSYNTHETIC PROCESS	21	-0.194330772	0.247566316
KERATAN SULFATE METABOLIC PROCESS	29	-0.428391582	0.001035527
REGULATION OF COAGULATION	77	-0.016085634	0.862862029
MEMBRANE BIOGENESIS	28	-0.259437654	0.065075525
REGULATION OF EPIDERMIS DEVELOPMENT	55	0.15768443	0.161763097
FORMATION OF PRIMARY GERM LAYER	100	-0.171141287	0.029183413
NEGATIVE REGULATION OF MUSCLE CELL DIFFERENTIATION	45	0.074722444	0.546128512
ENERGY RESERVE METABOLIC PROCESS	64	0.053395393	0.606152248
GLYCOLIPID BIOSYNTHETIC PROCESS	53	-0.134667074	0.213411166
REGULATION OF ACTIN NUCLEATION	26	0.092200154	0.572063723
REGULATION OF DENDRITIC CELL DIFFERENTIATION	9	-0.370952732	0.102906214
EPITHELIAL CELL DEVELOPMENT	165	-0.027914473	0.660941914
AMIDE TRANSPORT	68	0.014731723	0.882564347
REGULATION OF INTERFERON GAMMA PRODUCTION	76	0.201274985	0.034927171
NEGATIVE REGULATION OF LIPID METABOLIC PROCESS	65	0.22600597	0.028283287
PROTEIN LOCALIZATION TO KINETOCHORE	11	-0.320255665	0.145342597
NEGATIVE REGULATION OF FAT CELL DIFFERENTIATION	38	0.026652939	0.841865411
PHOTOPERIODISM	21	-0.099342667	0.568819907
ALPHA AMINO ACID BIOSYNTHETIC PROCESS	68	-0.096317421	0.32056351
MESENCHYMAL TO EPITHELIAL TRANSITION	14	0.056989506	0.796552815

ESTABLISHMENT OF ENDOTHELIAL INTESTINAL BARRIER	11	-0.051563852	0.832462213
SALIVARY GLAND DEVELOPMENT	29	0.20294909	0.191305765
CELL CYCLE DNA REPLICATION	10	0.261673703	0.315119418
NEGATIVE REGULATION OF TRANSCRIPTION ELONGATION FROM RNA POLYMERASE II PROMOTER	10	-0.19199999	0.429806742
CHROMOSOME CONDENSATION	27	-0.230621128	0.114754416
POSITIVE REGULATION OF RESPONSE TO STIMULUS	1621	-0.015676214	0.464830608
REGULATION OF MONOOXYGENASE ACTIVITY	52	0.267614373	0.020106261
SENSORY PERCEPTION OF MECHANICAL STIMULUS	120	-0.016016343	0.83024624
ESTABLISHMENT OF EPITHELIAL CELL POLARITY	20	-0.362840873	0.024913385
TOLL LIKE RECEPTOR 9 SIGNALING PATHWAY	12	0.101470934	0.672220901
EMBRYONIC DIGIT MORPHOGENESIS	55	-0.133072765	0.212178856
REGULATION OF CANONICAL WNT SIGNALING PATHWAY	213	0.088388438	0.122927788
NITROGEN CYCLE METABOLIC PROCESS	13	-0.033967189	0.880084894
T CELL ACTIVATION INVOLVED IN IMMUNE RESPONSE	45	0.023403337	0.848753388
NEGATIVE REGULATION OF LEUKOCYTE PROLIFERATION	57	-0.120194705	0.253889611
NECROPTOTIC PROCESS	21	0.004982251	0.977772776
NEGATIVE REGULATION OF GLIOGENESIS	35	-0.062415519	0.64697187
DENDRITIC CELL CHEMOTAXIS	15	0.08694636	0.684569696
REGULATION OF ANTIGEN PROCESSING AND PRESENTATION OF PEPTIDE ANTIGEN	10	-0.069832028	0.783307692
REGULATION OF NEURONAL SYNAPTIC PLASTICITY	41	0.111534528	0.391411726
REGULATION OF PHOSPHOLIPASE C ACTIVITY	30	-0.063754441	0.665068292
REGULATION OF DEVELOPMENTAL GROWTH	263	0.047613298	0.353811004
MEMBRANE DISASSEMBLY	47	-0.478187441	5.34E-06
REGULATION OF SYNAPTIC TRANSMISSION GLUTAMATERGIC	41	-0.08149794	0.515167257
ACTIVATION OF PHOSPHOLIPASE C ACTIVITY	18	0.023039484	0.905391338
NEGATIVE REGULATION OF BIOMINERAL TISSUE DEVELOPMENT	16	-0.123787664	0.53108898
NEGATIVE REGULATION OF BONE RESORPTION	9	-0.301856554	0.22889737
CELLULAR RESPONSE TO EXTERNAL STIMULUS	243	-0.04125102	0.431269665
NADP METABOLIC PROCESS	26	-0.153814678	0.316431036
POSITIVE REGULATION OF MRNA PROCESSING	31	-0.069119062	0.632659511
EPITHELIAL TUBE BRANCHING INVOLVED IN LUNG MORPHOGENESIS	24	0.34769071	0.04280732
DETECTION OF MECHANICAL STIMULUS INVOLVED IN SENSORY PERCEPTION	21	0.150105831	0.410305415
REGULATION OF CELL CELL ADHESION MEDIATED BY CADHERIN	11	0.217386191	0.382798294
METANEPHRIC EPITHELIUM DEVELOPMENT	16	0.119395163	0.565485029
REGULATION OF SMOOTH MUSCLE CELL DIFFERENTIATION	18	-0.081473751	0.666834931
FATTY ACID BETA OXIDATION	46	0.128944435	0.293585621
ACTIN FILAMENT BASED PROCESS	388	0.020530059	0.626991334
RESPONSE TO FLUID SHEAR STRESS	31	0.067041059	0.652961141
NEGATIVE REGULATION OF MUSCLE CONTRACTION	19	0.023944597	0.899024516
TRANSITION METAL ION TRANSPORT	93	0.050924548	0.553311655
POSITIVE REGULATION OF MUSCLE CELL DIFFERENTIATION	75	-0.042586831	0.649625268
REGULATION OF ADAPTIVE IMMUNE RESPONSE	102	-0.026593549	0.742015436
PHOSPHATIDYLINOSITOL BIOSYNTHETIC PROCESS	110	-0.042543692	0.583027963
POSITIVE REGULATION OF CELL COMMUNICATION	1331	-0.028782389	0.21789096
POSITIVE REGULATION OF LIPID KINASE ACTIVITY	30	-0.300820071	0.025313838
RESPONSE TO CORTICOSTEROID	152	0.012067648	0.856756376
POSITIVE REGULATION OF LEUKOCYTE DEGRANULATION	15	-0.026352622	0.900140118
SODIUM ION TRANSMEMBRANE TRANSPORT	65	0.071026118	0.490131934
PROTEIN UBIQUITINATION INVOLVED IN UBIQUITIN DEPENDENT PROTEIN CATABOLIC PROCESS	128	0.074980529	0.308858742
NEGATIVE REGULATION OF BLOOD VESSEL ENDOTHELIAL CELL MIGRATION	22	-0.068461666	0.689655526
CELL PROJECTION ASSEMBLY	235	-0.013522329	0.80091439
REGULATION OF PROTEIN IMPORT INTO NUCLEUS TRANSLOCATION	20	-0.233905799	0.168601364
GLUCOCORTICOID METABOLIC PROCESS	7	0.054058652	0.862524916
PROTEIN MATURATION	229	-0.02516007	0.642317208
SKELETAL MUSCLE ADAPTATION	8	-0.011098351	0.969321373
PROTEIN N LINKED GLYCOSYLATION	70	-0.167921711	0.073190816
ANAPHASE PROMOTING COMPLEX DEPENDENT CATABOLIC PROCESS	73	-0.249957361	0.004879699
HINDLIMB MORPHOGENESIS	31	0.174500465	0.244032939
ESTABLISHMENT OR MAINTENANCE OF EPITHELIAL CELL APICAL BASAL POLARITY	28	-0.080309177	0.596886639
LOOP OF HENLE DEVELOPMENT	8	0.374483504	0.191404027
VESICLE ORGANIZATION	249	0.013882561	0.791147165
KERATINOCYTE PROLIFERATION	10	0.636107569	0.002566829

MACROPHAGE ACTIVATION INVOLVED IN IMMUNE RESPONSE	10	0.215136659	0.408912749
INDOLALKYLAMINE METABOLIC PROCESS	15	0.277558698	0.186686951
POSITIVE REGULATION OF BINDING	121	0.050953106	0.499212384
SMOOTH MUSCLE CELL DIFFERENTIATION	28	0.038669531	0.80415497
MALE GENITALIA DEVELOPMENT	17	0.005485844	0.977979376
REGULATION OF HYDROLASE ACTIVITY	1123	0.001975755	0.937953021
ENDOCARDIAL CUSHION DEVELOPMENT	31	0.160144479	0.284473497
NEGATIVE REGULATION OF ERBB SIGNALING PATHWAY	42	-0.029551079	0.813969585
RESPONSE TO DIETARY EXCESS	17	0.169720265	0.399269086
REGULATION OF MEIOTIC NUCLEAR DIVISION	23	0.20596454	0.234785347
BRANCH ELONGATION OF AN EPITHELIUM	17	-0.318176448	0.070933242
POSITIVE REGULATION OF CELLULAR AMIDE METABOLIC PROCESS	102	0.045938072	0.575431785
POSITIVE REGULATION OF VACUOLE ORGANIZATION	12	-0.423637094	0.032348091
NEGATIVE REGULATION OF PLATELET ACTIVATION	15	-0.029366734	0.888720399
GLUTAMINE FAMILY AMINO ACID CATABOLIC PROCESS	22	-0.136282537	0.415564763
EMBRYONIC SKELETAL SYSTEM DEVELOPMENT	114	0.08166032	0.295096788
NEURON MATURATION	23	-0.265279496	0.08930718
THYMIC T CELL SELECTION	17	0.056303762	0.779143135
CELLULAR RESPONSE TO BIOTIC STIMULUS	145	0.056473707	0.413010559
COENZYME A METABOLIC PROCESS	16	0.036562135	0.859169208
PROTEIN LOCALIZATION TO CILIUM	22	0.139318963	0.433003514
RNA STABILIZATION	28	0.106603061	0.499078355
ACTIN MYOSIN FILAMENT SLIDING	28	-0.029869667	0.845854871
REGULATION OF CELL MORPHOGENESIS INVOLVED IN DIFFERENTIATION	306	0.068344283	0.153152941
REGULATION OF PROTEIN HOMODIMERIZATION ACTIVITY	20	0.006006334	0.973855855
REGULATION OF HETEROTYPIC CELL CELL ADHESION	16	0.097963367	0.637109939
POSITIVE REGULATION OF REACTIVE OXYGEN SPECIES BIOSYNTHETIC PROCESS	43	0.124687035	0.326655438
ALPHA AMINO ACID METABOLIC PROCESS	199	-0.024108729	0.67797611
MITOCHONDRIAL DNA METABOLIC PROCESS	13	-0.076372041	0.731786998
CGMP METABOLIC PROCESS	16	0.120880763	0.561170836
ADULT BEHAVIOR	112	0.059983017	0.444340499
RETINAL GANGLION CELL AXON GUIDANCE	15	0.061019115	0.775284755
POSITIVE REGULATION OF MEMBRANE INVAGINATION	10	0.408869866	0.091341241
SPINDLE LOCALIZATION	37	-0.020827135	0.876508278
HYDROGEN PEROXIDE CATABOLIC PROCESS	12	-0.113319354	0.620644089
CELLULAR RESPONSE TO ORGANIC CYCLIC COMPOUND	423	0.052110426	0.200847643
POSITIVE REGULATION OF CYTOKINE PRODUCTION INVOLVED IN IMMUNE RESPONSE	28	0.142257765	0.365297174
REGULATION OF PROTEIN SUMOYLATION	17	-0.189896806	0.30845852
RESPONSE TO ENDOPLASMIC RETICULUM STRESS	217	-0.109587607	0.044166889
CELLULAR RESPONSE TO LIGHT STIMULUS	78	-0.180923046	0.039111238
POSITIVE REGULATION BY HOST OF VIRAL TRANSCRIPTION	12	-0.091516506	0.691293897
NEGATIVE REGULATION OF STEM CELL DIFFERENTIATION	36	0.079472924	0.565506156
NEGATIVE REGULATION OF PRODUCTION OF MOLECULAR MEDIATOR OF IMMUNE RESPONSE	23	0.123613225	0.476742572
SPHINGOLIPID MEDIATED SIGNALING PATHWAY	9	0.037585596	0.891185274
SYNAPTIC VESICLE CYTOSKELETAL TRANSPORT	15	-0.059714115	0.774453728
LUNG CELL DIFFERENTIATION	23	0.214051662	0.21950511
PLATELET ACTIVATION	130	-0.073263587	0.300634716
CYTOSKELETON ORGANIZATION	733	0.009745976	0.753491365
NUCLEOTIDE BINDING DOMAIN LEUCINE RICH REPEAT CONTAINING RECEPTOR SIGNALING PATHWAY	26	-0.093067078	0.553658182
TRANSCRIPTION COUPLED NUCLEOTIDE EXCISION REPAIR	69	0.088395491	0.377533114
MEMBRANE DEPOLARIZATION DURING ACTION POTENTIAL	32	-0.148201292	0.28492792
MATURATION OF SSU RRNA	41	-0.090894548	0.466871063
BETA AMYLOID METABOLIC PROCESS	11	-0.262392214	0.250838703
RESPONSE TO WOUNDING	487	-0.049137486	0.187496754
NON CANONICAL WNT SIGNALING PATHWAY	129	-0.126304227	0.071057453
RENAL SYSTEM PROCESS INVOLVED IN REGULATION OF BLOOD VOLUME	14	-0.165766341	0.424354998
NEGATIVE REGULATION OF LEUKOCYTE MEDIATED IMMUNITY	40	-0.132128544	0.289872738
POSITIVE REGULATION OF CREB TRANSCRIPTION FACTOR ACTIVITY	12	-0.021106407	0.928461362
NEGATIVE REGULATION OF CELL GROWTH	155	0.099568112	0.138215446
MODIFIED AMINO ACID TRANSPORT	19	-0.12783972	0.482276562
CGMP BIOSYNTHETIC PROCESS	9	0.341104635	0.195449332
PROTEIN SECRETION	96	-0.041837478	0.613965495
ALTERNATIVE MRNA SPLICING VIA SPLICEOSOME	11	-0.522603844	0.016987466

ASSOCIATIVE LEARNING	62	-0.085238437	0.402810179
SNORNA METABOLIC PROCESS	9	-0.301971015	0.210888279
NEGATIVE REGULATION OF CELLULAR CATABOLIC PROCESS	146	-0.000989645	0.988391025
REGULATION OF RESPONSE TO EXTRACELLULAR STIMULUS	157	-0.011669353	0.858516897
COFACTOR TRANSPORT	23	0.050724533	0.768449121
POSITIVE REGULATION OF FAT CELL DIFFERENTIATION	47	0.00479218	0.968046692
NEGATIVE REGULATION OF PROTEIN POLYMERIZATION	48	0.111294546	0.35397948
EXOGENOUS DRUG CATABOLIC PROCESS	6	0.010636771	0.974652915
NEGATIVE REGULATION OF REACTIVE OXYGEN SPECIES METABOLIC PROCESS	38	0.179283268	0.184017713
REGULATION OF PROTEIN DEACETYLATION	31	-0.225151628	0.097365655
OTIC VESICLE DEVELOPMENT	11	-0.093905475	0.696172895
POSITIVE REGULATION OF DNA TEMPLATED TRANSCRIPTION INITIATION	21	-0.214453236	0.200097754
NEURON PROJECTION EXTENSION INVOLVED IN NEURON PROJECTION GUIDANCE	10	0.079390607	0.762144143
PHOSPHOLIPID DEPHOSPHORYLATION	31	-0.033044512	0.820877202
NEGATIVE REGULATION OF DNA REPAIR	14	0.241704096	0.284422521
METAL ION TRANSPORT	453	0.087549802	0.027071267
INSULIN SECRETION	31	0.103002356	0.490315954
POSITIVE REGULATION OF FIBROBLAST PROLIFERATION	48	0.227921764	0.061152593
PREASSEMBLY OF GPI ANCHOR IN ER MEMBRANE	14	-0.067400787	0.754001822
NECROTIC CELL DEATH	28	0.098114435	0.532251432
REGULATION OF MAST CELL ACTIVATION INVOLVED IN IMMUNE RESPONSE	27	-0.253047505	0.082195796
POSITIVE REGULATION OF CELLULAR AMINE METABOLIC PROCESS	10	0.079933776	0.76031951
POSITIVE REGULATION OF INTRACELLULAR PROTEIN TRANSPORT	222	-0.032148686	0.558265782
REGULATION BY VIRUS OF VIRAL PROTEIN LEVELS IN HOST CELL	11	-0.118362912	0.619791317
CHROMOSOME LOCALIZATION	57	0.09725916	0.378313617
REGULATION OF RESPIRATORY SYSTEM PROCESS	14	-0.263367099	0.183301536
POSITIVE REGULATION OF ADENYLATE CYCLASE ACTIVITY	34	-0.038460511	0.78228633
POSITIVE REGULATION OF NEURON DIFFERENTIATION	271	-0.005539173	0.911937348
EMBRYONIC SKELETAL SYSTEM MORPHOGENESIS	87	0.089492857	0.316118465
ACTIVATION OF GTPASE ACTIVITY	65	0.132140472	0.202183949
INOSITOL PHOSPHATE METABOLIC PROCESS	51	0.002901337	0.979839425
T CELL HOMEOSTASIS	28	-0.186096296	0.204201512
REGULATION OF RECEPTOR ACTIVITY	98	-0.047092451	0.565799567
HISTONE METHYLATION	72	-0.181202419	0.048954797
CELLULAR RESPONSE TO FLUID SHEAR STRESS	18	-0.140831212	0.447861448
POSITIVE REGULATION OF TRANSPORTER ACTIVITY	63	-0.171634736	0.081869474
POSITIVE REGULATION OF TRANSCRIPTION FACTOR IMPORT INTO NUCLEUS	46	0.172528377	0.160442822
ALPHA BETA T CELL DIFFERENTIATION	38	0.076194795	0.571818064
ESTABLISHMENT OF PROTEIN LOCALIZATION TO CHROMOSOME	12	-0.099540486	0.664233053
PEPTIDE CROSS LINKING	29	0.18711701	0.224136715
PEPTIDYL SERINE MODIFICATION	140	0.100433497	0.155395067
CELLULAR RESPONSE TO EXTRACELLULAR STIMULUS	177	-0.03248848	0.596717242
ROUNDABOUT SIGNALING PATHWAY	10	0.347813003	0.170984743
ESTABLISHMENT OF PROTEIN LOCALIZATION	1281	-0.052341755	0.027008957
NEGATIVE REGULATION OF RESPONSE TO DNA DAMAGE STIMULUS	49	0.03646714	0.757093861
GLUTAMATE METABOLIC PROCESS	26	-0.064430813	0.683276689
SPHINGOMYELIN METABOLIC PROCESS	12	0.395998942	0.081865106
TRNA METABOLIC PROCESS	162	-0.182764018	0.002912557
NEGATIVE REGULATION OF ATPASE ACTIVITY	12	0.057162895	0.810862707
POSITIVE REGULATION OF LOCOMOTION	381	0.005166323	0.903230285
REGULATION OF DENDRITE MORPHOGENESIS	65	-0.076550068	0.443047837
CELLULAR RESPONSE TO LITHIUM ION	14	-0.099839572	0.638952109
MAMMARY GLAND EPITHELIUM DEVELOPMENT	48	0.034099796	0.774577038
CELLULAR RESPONSE TO PROSTAGLANDIN STIMULUS	22	0.300142771	0.085013551
REGULATION OF MYELOID CELL APOPTOTIC PROCESS	22	-0.12452985	0.460274242
NEGATIVE REGULATION OF VIRAL PROCESS	79	0.074123191	0.427847169
ERYTHROCYTE HOMEOSTASIS	65	-0.300841839	0.001409376
RESPONSE TO HEAT	72	-0.146951894	0.113975072
NOTCH RECEPTOR PROCESSING	15	-0.550732574	0.002617049
CELLULAR RESPONSE TO CADMIUM ION	14	-0.229515937	0.252801559
SUCKLING BEHAVIOR	11	-0.418489469	0.044267869
POSITIVE REGULATION OF KIDNEY DEVELOPMENT	37	-0.06577008	0.61958863
REGULATION OF MITOTIC SPINDLE CHECKPOINT	11	0.093813402	0.708192385
I KAPPAB KINASE NF KAPPAB SIGNALING	65	-0.021747094	0.829825525
REGULATION OF NON CANONICAL WNT SIGNALING PATHWAY	17	0.443581184	0.026061462

REGULATION OF KINASE ACTIVITY	694	-0.083631606	0.007418369
CHROMOSOME LOCALIZATION TO NUCLEAR ENVELOPE INVOLVED IN HOMOLOGOUS CHROMOSOME SEGREGATION	9	0.120244434	0.664597638
NEGATIVE REGULATION OF DNA RECOMBINATION	15	-0.201549296	0.31510406
POSITIVE REGULATION OF LYMPHOCYTE MIGRATION	24	-0.274783154	0.073431671
NEGATIVE REGULATION OF CELLULAR RESPONSE TO INSULIN STIMULUS	28	-0.067099088	0.659195203
POSITIVE REGULATION OF CELLULAR EXTRAVASATION	11	0.348831458	0.147063457
MYELOID LEUKOCYTE DIFFERENTIATION	87	0.041229448	0.641987001
PEROXISOME ORGANIZATION	28	0.030164551	0.846354593
DNA STRAND ELONGATION INVOLVED IN DNA REPLICATION	25	0.046078772	0.780308443
CELLULAR MODIFIED AMINO ACID CATABOLIC PROCESS	15	-0.165722433	0.407866044
SINGLE ORGANISM CELL ADHESION	390	-0.024503572	0.557240102
POLYOL METABOLIC PROCESS	84	-0.010038598	0.910500951
POSITIVE REGULATION OF TRANSCRIPTION INITIATION FROM RNA POLYMERASE II PROMOTER	14	-0.332739394	0.087265098
APICAL JUNCTION ASSEMBLY	35	-0.203481888	0.118836265
PYRIMIDINE RIBONUCLEOSIDE TRIPHOSPHATE METABOLIC PROCESS	14	-0.53030794	0.003550498
LYMPHOCYTE HOMEOSTASIS	42	-0.154190827	0.204417605
SERTOLI CELL DEVELOPMENT	13	-0.011491108	0.959519536
NEGATIVE REGULATION OF RNA SPLICING	24	-0.022300098	0.89331625
CELL CYCLE PHASE TRANSITION	251	-0.123570743	0.014473155
TISSUE MORPHOGENESIS	466	0.006164613	0.872920916
ENDOSOME TO LYSOSOME TRANSPORT	38	0.251776268	0.068195244
MRNA PROCESSING	405	-0.110830916	0.005758316
RESPONSE TO ANGIOTENSIN	14	0.225763728	0.305955574
DENDRITIC SPINE DEVELOPMENT	15	-0.043670333	0.834616763
REGULATION OF PROTEIN OLIGOMERIZATION	31	0.181828323	0.223609274
HEME METABOLIC PROCESS	25	-0.398023134	0.004977619
SERINE FAMILY AMINO ACID BIOSYNTHETIC PROCESS	15	-0.107843212	0.599709184
CEREBELLAR PURKINJE CELL LAYER FORMATION	9	-0.20630215	0.423522539
CELLULAR RESPONSE TO CARBOHYDRATE STIMULUS	65	-0.15512096	0.111174091
INOSITOL PHOSPHATE CATABOLIC PROCESS	11	-0.252298424	0.260375958
C21 STEROID HORMONE METABOLIC PROCESS	18	0.013826195	0.943030611
NEGATIVE REGULATION OF GLIAL CELL DIFFERENTIATION	24	-0.022445743	0.892609972
POSITIVE REGULATION OF SYNAPSE ASSEMBLY	48	0.097976767	0.414355878
TRANSMISSION OF NERVE IMPULSE	39	0.247779712	0.061550809
POSITIVE REGULATION OF VASCULATURE DEVELOPMENT	122	0.014731936	0.843426194
POSITIVE REGULATION OF EPIDERMAL CELL DIFFERENTIATION	17	0.047593822	0.812238833
MEMBRANE FUSION	133	-0.016631092	0.814762242
POSITIVE REGULATION OF TOR SIGNALING	29	-0.074240077	0.618767478
POSITIVE REGULATION OF GLIAL CELL DIFFERENTIATION	27	0.196483996	0.225642672
MALE SEX DETERMINATION	7	0.418604842	0.221675628
TYPE B PANCREATIC CELL DEVELOPMENT	12	0.051109948	0.830388677
REGULATION OF SUPEROXIDE ANION GENERATION	10	0.133334827	0.611884158
ANTIGEN RECEPTOR MEDIATED SIGNALING PATHWAY	148	-0.11851316	0.070503672
POSITIVE REGULATION OF MITOTIC CELL CYCLE	115	-0.079540367	0.289598139
LYMPHOCYTE COSTIMULATION	60	-0.1760186	0.081298498
REGULATION OF SERINE TYPE PEPTIDASE ACTIVITY	8	0.088211323	0.763362066
NEGATIVE REGULATION OF STEROID METABOLIC PROCESS	19	0.431250987	0.015220128
COFACTOR METABOLIC PROCESS	292	-0.04268028	0.372690952
BARBED END ACTIN FILAMENT CAPPING	12	0.199625943	0.404516425
CELL MIGRATION INVOLVED IN SPROUTING ANGIOGENESIS	14	-0.122037234	0.566099591
NEGATIVE REGULATION OF INTRACELLULAR ESTROGEN RECEPTOR SIGNALING PATHWAY	9	0.283357909	0.299499474
REGULATION OF TYPE 2 IMMUNE RESPONSE	23	0.132438379	0.444588365
3 UTR MEDIATED MRNA STABILIZATION	11	-0.271876197	0.229375741
DRUG CATABOLIC PROCESS	8	-0.188175805	0.488383254
POSITIVE REGULATION OF RHO PROTEIN SIGNAL TRANSDUCTION	12	-0.246906353	0.269495922
PRIMITIVE STREAK FORMATION	11	-0.321557485	0.150844967
RESPONSE TO PHENYLPROPANOID	7	0.343061124	0.250609141
REGULATION OF EPITHELIAL CELL MIGRATION	151	0.043310334	0.520889232
POSITIVE REGULATION OF NOTCH SIGNALING PATHWAY	29	0.109155538	0.480095019
CELL DEATH	893	-0.012533521	0.656179555
CELLULAR IRON ION HOMEOSTASIS	37	0.021280852	0.874968391
CONTRACTILE ACTIN FILAMENT BUNDLE ASSEMBLY	11	-0.402620584	0.05548418
MACROMOLECULE DEACYLATION	63	-0.003899179	0.969858129

TRANSLATIONAL INITIATION	143	-0.18926351	0.003770268
ACTIVATION OF NF KAPPAB INDUCING KINASE ACTIVITY	16	-0.018214459	0.928772729
SYNAPTIC TRANSMISSION CHOLINERGIC	16	0.08790732	0.671532216
NEGATIVE REGULATION OF CYTOKINE PRODUCTION INVOLVED IN IMMUNE RESPONSE	18	0.075331892	0.69982719
REGULATION OF TOLL LIKE RECEPTOR 4 SIGNALING PATHWAY	16	-0.151044579	0.443740008
POSITIVE REGULATION OF CELLULAR PROTEIN LOCALIZATION	318	-0.049221066	0.283073836
PLASMA MEMBRANE ORGANIZATION	181	0.10359135	0.096219806
REGULATION OF NEUROTRANSMITTER SECRETION	41	-0.040537761	0.748988696
POSITIVE REGULATION OF PROTEIN CATABOLIC PROCESS	244	-0.046924554	0.369121698
ACTIVATION OF ADENYLATE CYCLASE ACTIVITY	27	-0.013532891	0.931351244
WHITE FAT CELL DIFFERENTIATION	11	0.019014733	0.938808156
OOGENESIS	53	0.120504042	0.292498184
REGULATION OF SHORT TERM NEURONAL SYNAPTIC PLASTICITY	12	-0.061424724	0.791821707
ALDEHYDE CATABOLIC PROCESS	12	0.014127246	0.952453263
RESPONSE TO TYPE I INTERFERON	57	-0.196775918	0.058152194
MITOCHONDRIAL RESPIRATORY CHAIN COMPLEX ASSEMBLY	72	-0.121348496	0.193445887
HYALURONAN CATABOLIC PROCESS	14	0.109190283	0.622660174
NEUROTRANSMITTER BIOSYNTHETIC PROCESS	7	0.45269038	0.140138594
PENTOSE PHOSPHATE SHUNT	13	-0.060964046	0.785078318
CYTOKINE MEDIATED SIGNALING PATHWAY	369	-0.051605312	0.225963911
ATP GENERATION FROM ADP	35	0.070720034	0.613891519
NEGATIVE REGULATION OF LOCOMOTION	238	0.027796304	0.605010512
IMP METABOLIC PROCESS	14	-0.102515491	0.630721253
NCRNA 3 END PROCESSING	20	-0.198095965	0.247186551
LYMPH NODE DEVELOPMENT	15	-0.236015514	0.224293533
REGULATION OF CELLULAR COMPONENT SIZE	301	0.0205245	0.66780227
MITOCHONDRIAL TRANSPORT	167	-0.139699169	0.022534255
SULFUR COMPOUND BIOSYNTHETIC PROCESS	171	-0.109099629	0.074496674
T CELL PROLIFERATION	31	-0.26553745	0.049040987
REGULATION OF RESPIRATORY GASEOUS EXCHANGE BY NEUROLOGICAL SYSTEM PROCESS	11	-0.363257758	0.085130164
PROTEIN TARGETING	379	-0.088532947	0.033801233
POSITIVE REGULATION OF CHOLESTEROL EFFLUX	13	0.224011216	0.338115256
SINGLE ORGANISM BIOSYNTHETIC PROCESS	1155	-0.072626954	0.003277707
POSITIVE REGULATION OF VIRAL GENOME REPLICATION	30	-0.019006705	0.898410952
NEGATIVE REGULATION OF INNATE IMMUNE RESPONSE	32	-0.378955939	0.002599834
REGULATION OF ALPHA AMINO 3 HYDROXY 5 METHYL 4 ISOXAZOLE PROPIONATE SELECTIVE GLUTAMATE RECEPTOR ACTIVITY	16	0.075746427	0.715084913
REGULATION OF ENDOTHELIAL CELL DIFFERENTIATION	23	0.124275381	0.474203331
REGULATION OF NOREPINEPHRINE SECRETION	13	0.239834705	0.295800022
GLIAL CELL DEVELOPMENT	68	0.01629463	0.870256675
MITOCHONDRIAL TRANSLATION	102	-0.143876761	0.064899545
REGULATION OF RETINOIC ACID RECEPTOR SIGNALING PATHWAY	21	-0.126488762	0.46252941
STRIATED MUSCLE CELL DIFFERENTIATION	149	-0.021176628	0.752077697
REGULATION OF SYSTEMIC ARTERIAL BLOOD PRESSURE	66	0.107741026	0.293209076
REGULATION OF SMOOTH MUSCLE CONTRACTION	51	0.11387838	0.329781755
POLYOL CATABOLIC PROCESS	16	-0.281305807	0.123815584
SECOND MESSENGER MEDIATED SIGNALING	137	-0.124395019	0.066630321
CELL CELL SIGNALING	580	0.062210866	0.07569024
NEGATIVE REGULATION OF B CELL ACTIVATION	25	-0.241070381	0.118078112
POSITIVE REGULATION OF COAGULATION	24	-0.024279716	0.883840637
NEGATIVE REGULATION OF PROTEIN TYROSINE KINASE ACTIVITY	19	-0.036929729	0.842743495
LUNG MORPHOGENESIS	43	0.224016554	0.082007168
VENTRICULAR CARDIAC MUSCLE CELL DIFFERENTIATION	17	0.242955761	0.228279142
OXIDATIVE DEMETHYLATION	9	0.108903394	0.694056286
REGULATION OF MACROPHAGE ACTIVATION	19	0.031908244	0.865905087
VASCULAR ENDOTHELIAL GROWTH FACTOR RECEPTOR SIGNALING PATHWAY	72	0.097283566	0.322375792
POSITIVE REGULATION OF MAPK CASCADE	397	-0.040230031	0.32926661
MULTICELLULAR ORGANISMAL MACROMOLECULE METABOLIC PROCESS	63	-0.149419115	0.131732419
CARBOHYDRATE DERIVATIVE CATABOLIC PROCESS	147	0.005773276	0.932223638
L AMINO ACID TRANSPORT	49	-0.058883974	0.609787405
POSITIVE REGULATION OF ACTION POTENTIAL	8	0.121456964	0.680490808
REGULATION OF MUSCLE CONTRACTION	125	-0.023139557	0.751556694
REGULATION OF CHOLESTEROL EFFLUX	16	-0.098501984	0.622508508
REGULATION OF EXOCYTOSIS	153	-0.009413296	0.887155979

NEGATIVE REGULATION OF TOLL LIKE RECEPTOR SIGNALING PATHWAY	25	0.080516433	0.628675987
ANION TRANSMEMBRANE TRANSPORT	198	-0.006961079	0.905171685
POSITIVE REGULATION OF PROTEIN LOCALIZATION TO CELL SURFACE	10	-0.249373911	0.298443819
PROTEIN MODIFICATION BY SMALL PROTEIN CONJUGATION OR REMOVAL	780	-0.062249296	0.036109278
RENAL VESICLE DEVELOPMENT	12	-0.099787432	0.664662416
REGULATION OF BODY FLUID LEVELS	431	-0.040391753	0.308057581
REGULATION OF HORMONE LEVELS	380	0.012392937	0.771159425
BONE CELL DEVELOPMENT	20	-0.394890124	0.011389296
REGULATION OF DEFENSE RESPONSE	640	-0.047077667	0.150144506
NEGATIVE REGULATION OF NEUROLOGICAL SYSTEM PROCESS	12	0.160433203	0.503419117
PYRIMIDINE NUCLEOSIDE MONOPHOSPHATE METABOLIC PROCESS	14	-0.026900632	0.901514886
POSITIVE REGULATION OF HEART GROWTH	24	0.115059835	0.497944867
NEGATIVE REGULATION OF LIPID BIOSYNTHETIC PROCESS	39	0.26965034	0.04132378
NEGATIVE REGULATION OF PEPTIDE SECRETION	39	0.033425315	0.800134632
VESICLE TARGETING	73	-0.205361477	0.023156413
REGULATION OF PROTEIN EXPORT FROM NUCLEUS	33	-0.276009771	0.031708683
LAYER FORMATION IN CEREBRAL CORTEX	14	-0.07108515	0.74096825
POSITIVE REGULATION OF ACTIN CYTOSKELETON REORGANIZATION	14	-0.02992471	0.890394987
SEX DETERMINATION	13	0.549065642	0.020673896
OLIGOSACCHARIDE CATABOLIC PROCESS	9	0.26347534	0.330775343
B CELL PROLIFERATION	24	0.164969965	0.334096788
POSITIVE REGULATION OF DNA METABOLIC PROCESS	167	-0.020294426	0.748782246
PROTEASOME ASSEMBLY	13	-0.329755874	0.094230521
ESTABLISHMENT OF PROTEIN LOCALIZATION TO ENDOPLASMIC RETICULUM	104	-0.133308068	0.086024534
PYRIMIDINE NUCLEOSIDE TRIPHOSPHATE BIOSYNTHETIC PROCESS	17	-0.41117209	0.016814933
NEGATIVE REGULATION OF ENDOCYTOSIS	33	-0.222896055	0.092953594
RESPONSE TO POTASSIUM ION	10	0.171036383	0.515337529
REGULATION OF LYMPHOCYTE APOPTOTIC PROCESS	47	-0.131799224	0.252841658
SMALL MOLECULE METABOLIC PROCESS	1486	-0.033322306	0.133681276
THYMUS DEVELOPMENT	42	-0.105724311	0.390680416
NEGATIVE REGULATION OF ADHERENS JUNCTION ORGANIZATION	15	-0.265723843	0.164375929
HOMEOSTATIC PROCESS	1100	0.004123642	0.872201025
CEREBELLAR CORTEX MORPHOGENESIS	24	-0.142909271	0.373070986
POSITIVE REGULATION OF CYTOPLASMIC TRANSPORT	239	-0.053510362	0.309856327
MORPHOGENESIS OF AN EPITHELIUM	357	-0.026929911	0.536465573
OSTEOBLAST DIFFERENTIATION	113	-0.021533012	0.779390001
REGULATION OF STRIATED MUSCLE CELL DIFFERENTIATION	75	0.048002753	0.615422902
DEFENSE RESPONSE TO VIRUS	133	-0.080763488	0.247441447
SENSORY PERCEPTION OF CHEMICAL STIMULUS	101	0.250058753	0.002287168
REGULATION OF RUFFLE ASSEMBLY	19	0.152600068	0.422946876
CELLULAR PIGMENTATION	42	0.124518745	0.332128451
NEGATIVE REGULATION OF CALCIUM ION DEPENDENT EXOCYTOSIS	10	0.229655293	0.384715344
VACUOLAR TRANSPORT	235	0.097779712	0.07399573
NEGATIVE REGULATION OF CELL CELL ADHESION	123	0.006447147	0.930724752
POSITIVE REGULATION OF VIRAL RELEASE FROM HOST CELL	15	0.369458361	0.103771339
LEUKOCYTE MEDIATED CYTOTOXICITY	27	0.018267867	0.908047914
POSITIVE REGULATION OF VACUOLAR TRANSPORT	14	-0.246967653	0.234551504
POSITIVE REGULATION OF LYASE ACTIVITY	41	-0.044613786	0.724453865
ENTRAINMENT OF CIRCADIAN CLOCK BY PHOTOPERIOD	18	-0.144166152	0.43924766
SUPEROXIDE METABOLIC PROCESS	22	0.07211875	0.683493567
ORGANIC HYDROXY COMPOUND METABOLIC PROCESS	395	0.051248211	0.223525763
TELOMERE MAINTENANCE VIA TELOMERE LENGTHENING	24	-0.107905351	0.507011202
PHOTOTRANSDUCTION	28	0.043968401	0.778130368
REGULATION OF LIGASE ACTIVITY	120	-0.095648906	0.191151533
REGULATION OF OXIDOREDUCTASE ACTIVITY	77	0.146823164	0.12402866
SULFATE TRANSPORT	9	-0.490388287	0.023352405
RESPONSE TO FOOD	16	-0.052981369	0.793075577
REGULATION OF PROTEIN INSERTION INTO MITOCHONDRIAL MEMBRANE INVOLVED IN APOPTOTIC SIGNALING PATHWAY	28	-0.151871306	0.304956813
TELOMERE ORGANIZATION	100	-0.077020782	0.338783984
IMMUNE SYSTEM DEVELOPMENT	508	-0.039682432	0.278413653
CAMP BIOSYNTHETIC PROCESS	11	0.228195703	0.357611744
ALPHA AMINO ACID CATABOLIC PROCESS	79	0.059886027	0.520692635
CELLULAR COMPONENT MAINTENANCE	14	-0.121794348	0.565242495
REGULATION OF CARDIAC MUSCLE CELL CONTRACTION	26	-0.195689483	0.195847314
FC GAMMA RECEPTOR SIGNALING PATHWAY	65	0.023714598	0.816498295

RRNA METABOLIC PROCESS	246	-0.105264401	0.039972774
RESPIRATORY SYSTEM PROCESS	14	-0.113216681	0.594286499
NEUROTRANSMITTER TRANSPORT	119	0.020458376	0.786686853
REGULATION OF T HELPER CELL DIFFERENTIATION	22	0.11065618	0.534214147
REGULATION OF VOLTAGE GATED CALCIUM CHANNEL ACTIVITY	17	-0.291214038	0.104659173
PH REDUCTION	34	0.262962121	0.065610453
POSITIVE REGULATION OF TRIGLYCERIDE LIPASE ACTIVITY	6	-0.135788612	0.673356379
MONOSACCHARIDE CATABOLIC PROCESS	49	0.009847198	0.933093382
POSITIVE REGULATION OF MRNA METABOLIC PROCESS	42	-0.074121188	0.550271201
RESPONSE TO FUNGICIDE	8	-0.072842231	0.79744968
NEGATIVE REGULATION BY HOST OF VIRAL TRANSCRIPTION	12	0.266679457	0.263513323
REGULATION OF EPIDERMAL GROWTH FACTOR ACTIVATED RECEPTOR ACTIVITY	21	-0.061360288	0.72760326
PROTEIN NEDDYLATION	13	0.056958334	0.803759409
POTASSIUM ION HOMEOSTASIS	16	-0.021016396	0.917802557
HORMONE TRANSPORT	56	-0.090814175	0.395946271
PROTEIN CATABOLIC PROCESS	507	-0.034989794	0.340210832
POSITIVE REGULATION OF INTRACELLULAR STEROID HORMONE RECEPTOR SIGNALING PATHWAY	12	0.230644214	0.34972647
NEGATIVE REGULATION OF MYOTUBE DIFFERENTIATION	15	0.264484186	0.21008897
INORGANIC ANION TRANSPORT	95	0.02491769	0.768395681
CARBOHYDRATE PHOSPHORYLATION	20	-0.00944639	0.958754167
PROTEIN IMPORT	139	-0.007220873	0.917366093
NUCLEOSIDE BISPHOSPHATE METABOLIC PROCESS	33	0.070942782	0.622971241
REGULATION OF INTRACELLULAR STEROID HORMONE RECEPTOR SIGNALING PATHWAY	51	-0.025342878	0.824197925
MAMMARY GLAND EPITHELIAL CELL DIFFERENTIATION	16	0.430215754	0.032492437
CELLULAR RESPONSE TO GAMMA RADIATION	19	-0.135307552	0.453697966
REGULATION OF HEAT GENERATION	15	0.060425406	0.777155777
REGULATION OF FIBROBLAST MIGRATION	26	0.006637388	0.96707375
INDUCTION OF POSITIVE CHEMOTAXIS	12	-0.054231106	0.815912768
REGENERATION	144	-0.038778072	0.567929517
EXTRINSIC APOPTOTIC SIGNALING PATHWAY	87	-0.10808492	0.20689568
REGULATION OF TRANSLATIONAL ELONGATION	23	-0.091305798	0.58438598
CELL MATRIX ADHESION	105	-0.027354495	0.731165932
PITUITARY GLAND DEVELOPMENT	33	-0.076498632	0.584099478
MATING	28	0.28078866	0.071344151
DEVELOPMENTAL INDUCTION	23	-0.270029983	0.082694319
REGULATION OF TRANSFERASE ACTIVITY	844	-0.086943531	0.002260501
NEGATIVE REGULATION OF ALCOHOL BIOSYNTHETIC PROCESS	16	0.598092066	0.000698261
CHROMOSOME SEPARATION	16	-0.488406809	0.002146639
GANGLIOSIDE METABOLIC PROCESS	22	-0.061138746	0.722032149
TISSUE HOMEOSTASIS	133	0.092480388	0.2014723
ANTIGEN PROCESSING AND PRESENTATION VIA MHC CLASS IB	12	-0.080366968	0.728411212
GAMMA AMINOBUTYRIC ACID SIGNALING PATHWAY	18	0.04801747	0.805139758
POSITIVE REGULATION OF STEROL TRANSPORT	15	0.200943628	0.353935778
ADAPTIVE IMMUNE RESPONSE BASED ON SOMATIC RECOMBINATION OF IMMUNE RECEPTORS BUILT FROM IMMUNOGLOBULIN SUPERFAMILY DOMAINS	102	0.073716373	0.370945358
CELL CYCLE ARREST	144	-0.080380714	0.231645809
THYROID HORMONE METABOLIC PROCESS	13	0.118360464	0.607869881
APOPTOTIC CELL CLEARANCE	21	0.10132216	0.577584194
MODIFICATION OF MORPHOLOGY OR PHYSIOLOGY OF OTHER ORGANISM	90	0.124691986	0.156915908
RIBOSOME ASSEMBLY	51	-0.274721178	0.00913108
NEGATIVE REGULATION OF ACTIN FILAMENT DEPOLYMERIZATION	29	0.046563088	0.761403435
DNA LIGATION INVOLVED IN DNA REPAIR	12	0.479616508	0.0327038
REGULATION OF CELL JUNCTION ASSEMBLY	65	-0.00576248	0.954750565
HYPEROSMOTIC RESPONSE	17	-0.200720505	0.28236743
REGULATION OF SMAD PROTEIN IMPORT INTO NUCLEUS	12	-0.002065938	0.993019355
NEGATIVE REGULATION OF NATURAL KILLER CELL MEDIATED IMMUNITY	10	-0.467115402	0.029082118
ARTERY DEVELOPMENT	70	-0.014260947	0.883939804
PROTEIN DEALKYLATION	26	-0.019824589	0.901319156
NEGATIVE REGULATION OF DENDRITE MORPHOGENESIS	12	-0.172503088	0.439505273
NEGATIVE REGULATION OF TYPE I INTERFERON PRODUCTION	37	-0.105282739	0.422224547
EXTRACELLULAR MATRIX ASSEMBLY	14	-0.071265226	0.739946424
POSTREPLICATION REPAIR	51	-0.094867643	0.39806359
SKELETAL SYSTEM DEVELOPMENT	395	0.005798728	0.889556427

ORGANOPHOSPHATE METABOLIC PROCESS	776	-0.041369435	0.166431607
CARDIAC MYOFIBRIL ASSEMBLY	12	-0.044139187	0.850279005
REGULATION OF HEART CONTRACTION	179	0.085783273	0.169389828
CELLULAR AMINO ACID BIOSYNTHETIC PROCESS	78	-0.050817577	0.579633063
CRANIAL NERVE MORPHOGENESIS	20	-0.062095459	0.730499217
ACTIVATION OF JUN KINASE ACTIVITY	30	-0.055213061	0.7081883
REGULATION OF BROWN FAT CELL DIFFERENTIATION	10	0.025314945	0.922456552
FIBRIL ORGANIZATION	18	0.205483587	0.294576486
LONG CHAIN FATTY ACID METABOLIC PROCESS	64	0.152235844	0.143904782
MONOVALENT INORGANIC CATION TRANSPORT	322	-0.011287644	0.806037426
RELAXATION OF CARDIAC MUSCLE	13	-0.246291923	0.237371882
POSITIVE REGULATION OF BEHAVIOR	15	0.180405184	0.40145762
CALCIUM MEDIATED SIGNALING USING INTRACELLULAR CALCIUM SOURCE	15	-0.131319652	0.517237874
NEURAL TUBE DEVELOPMENT	138	-0.116560775	0.086231838
POLYSACCHARIDE METABOLIC PROCESS	74	0.0161058	0.866281882
REGULATION OF INTRACELLULAR SIGNAL TRANSDUCTION	1450	-0.035536767	0.113505118
CYCLIC NUCLEOTIDE METABOLIC PROCESS	44	0.085014142	0.497238299
POSITIVE REGULATION OF T CELL PROLIFERATION	82	-0.113827394	0.195423257
MULTICELLULAR ORGANISMAL MOVEMENT	33	0.011793723	0.934228986
CLATHRIN COAT ASSEMBLY	12	0.159279047	0.507705941
NEGATIVE REGULATION OF MEIOTIC NUCLEAR DIVISION	9	-0.004344284	0.987280298
VASCULAR SMOOTH MUSCLE CELL DIFFERENTIATION	12	-0.020015365	0.932176359
POSITIVE REGULATION OF BIOSYNTHETIC PROCESS	1573	0.027777543	0.204603224
NEGATIVE REGULATION OF NITROGEN COMPOUND METABOLIC PROCESS	1345	0.011182263	0.632984665
DNA INTEGRATION	8	-0.287425728	0.276906652
CELL ACTIVATION	484	-0.049987454	0.181178754
REGULATION OF MYELINATION	30	0.20841159	0.170846629
NEGATIVE REGULATION OF JUN KINASE ACTIVITY	13	-0.077157327	0.728599631
REGULATION OF AXON GUIDANCE	37	0.078651479	0.564490992
REGULATION OF DOPAMINE SECRETION	17	0.230284105	0.250209761
RESPONSE TO COPPER ION	25	-0.028212526	0.862312429
REGULATION OF CIRCADIAN SLEEP WAKE CYCLE	14	0.213612965	0.334616572
NEGATIVE REGULATION OF CELLULAR PROTEIN CATABOLIC PROCESS	62	-0.066170093	0.518164181
REGULATION OF DOUBLE STRAND BREAK REPAIR VIA HOMOLOGOUS RECOMBINATION	17	0.246584228	0.221712276
RESPONSE TO AXON INJURY	45	-0.161234276	0.167757464
REGULATION OF VACUOLE ORGANIZATION	38	0.049525831	0.712022516
CIRCADIAN REGULATION OF GENE EXPRESSION	54	0.07939377	0.483120616
RETINOL METABOLIC PROCESS	23	0.08820697	0.610033848
OXALOACETATE METABOLIC PROCESS	11	-0.080821352	0.737863179
NEGATIVE REGULATION OF TUMOR NECROSIS FACTOR MEDIATED SIGNALING PATHWAY	12	-0.048953718	0.833823366
NEGATIVE REGULATION OF EXOCYTOSIS	26	0.085235255	0.6008353
INTRACELLULAR STEROID HORMONE RECEPTOR SIGNALING PATHWAY	64	0.074642354	0.472776273
REGULATION OF WNT SIGNALING PATHWAY	278	0.060163436	0.229484622
NEGATIVE REGULATION OF CELL JUNCTION ASSEMBLY	19	-0.062345713	0.735889215
ANTIGEN PROCESSING AND PRESENTATION OF PEPTIDE ANTIGEN	157	-0.127715375	0.044081446
HIPPO SIGNALING	26	-0.361857858	0.014044809
NEGATIVE REGULATION OF EPITHELIAL TO MESENCHYMAL TRANSITION	20	0.079610022	0.667706373
REGULATION OF MEMBRANE DEPOLARIZATION	34	0.060642775	0.66923085
AMINO SUGAR BIOSYNTHETIC PROCESS	10	0.167623648	0.526010763
REGULATION OF NERVOUS SYSTEM DEVELOPMENT	645	0.019648537	0.552125843
REGULATION OF ESTABLISHMENT OF PROTEIN LOCALIZATION TO CHROMOSOME	10	0.113376336	0.666192453
REGULATION OF CARDIAC MUSCLE CELL DIFFERENTIATION	18	0.191668302	0.33115892
POSITIVE REGULATION OF DIGESTIVE SYSTEM PROCESS	8	0.159110104	0.593935276
INTRINSIC APOPTOTIC SIGNALING PATHWAY IN RESPONSE TO DNA DAMAGE	65	0.008231952	0.935588708
TETRAPYRROLE BIOSYNTHETIC PROCESS	26	-0.285438323	0.049930893
NUCLEOSIDE PHOSPHATE BIOSYNTHETIC PROCESS	160	-0.126086963	0.044926776
PROTEIN LIPID COMPLEX ASSEMBLY	18	0.146322899	0.456026911
POSITIVE REGULATION OF SODIUM ION TRANSPORT	32	0.126707181	0.391534546
DETECTION OF OTHER ORGANISM	10	-0.196785428	0.416723453
ESTABLISHMENT OF LOCALIZATION IN CELL	1529	-0.075894135	0.000496437
G PROTEIN COUPLED GLUTAMATE RECEPTOR SIGNALING PATHWAY	10	-0.003618238	0.988835096
NEURAL RETINA DEVELOPMENT	41	0.005958238	0.962899232
CLEAVAGE INVOLVED IN RRNA PROCESSING	19	0.020033291	0.915389288

REGULATION OF EXIT FROM MITOSIS	15	-0.297136179	0.117538048
FORELIMB MORPHOGENESIS	36	-0.034150815	0.800890003
ASPARTATE FAMILY AMINO ACID BIOSYNTHETIC PROCESS	22	-0.014940684	0.931552289
MANGANESE ION TRANSPORT	11	-0.26833055	0.232646759
POSITIVE REGULATION OF HISTONE H3 K4 METHYLATION	16	-0.140711376	0.47369245
NEURON PROJECTION DEVELOPMENT	472	0.04669436	0.226375909
MALE GAMETE GENERATION	349	0.122700447	0.00660592
POSITIVE REGULATION OF FIBROBLAST MIGRATION	9	-0.078963447	0.768006079
NEGATIVE REGULATION OF RESPONSE TO OXIDATIVE STRESS	33	0.006472279	0.963837237
CHAPERONE MEDIATED PROTEIN COMPLEX ASSEMBLY	12	0.080178955	0.73768386
ORGANELLE TRANSPORT ALONG MICROTUBULE	58	-0.068879264	0.515447358
CHONDROCYTE DEVELOPMENT	19	-0.038782496	0.834908263
MEMBRANE RAFT ORGANIZATION	15	0.01788017	0.932813997
MITOTIC SISTER CHROMATID SEGREGATION	85	-0.179345469	0.03388762
MICROTUBULE NUCLEATION	16	0.189086984	0.367875437
ESTABLISHMENT OF PROTEIN LOCALIZATION TO ORGANELLE	337	-0.05666659	0.202856262
NEGATIVE REGULATION OF ACUTE INFLAMMATORY RESPONSE	10	-0.071325694	0.778859678
REGULATION OF SEQUESTERING OF CALCIUM ION	91	-0.211265401	0.008789987
REGULATION OF MEMBRANE LIPID METABOLIC PROCESS	11	0.171174959	0.496734492
XENOPHAGY	94	-0.155208571	0.055211065
DRUG TRANSMEMBRANE TRANSPORT	16	0.076000142	0.714291025
POSITIVE REGULATION OF MEMBRANE DEPOLARIZATION	8	0.050774133	0.861929668
MYD88 DEPENDENT TOLL LIKE RECEPTOR SIGNALING PATHWAY	29	0.194048988	0.21087349
REGULATION OF PROTEIN POLYMERIZATION	153	0.10502789	0.120156875
REGULATION OF PROTEIN TARGETING TO MEMBRANE	28	-0.072522158	0.633049256
KIDNEY EPITHELIUM DEVELOPMENT	109	-0.012277981	0.875594078
HISTONE H2A UBIQUITINATION	16	0.165497985	0.429179218
TOOTH MINERALIZATION	11	0.001914346	0.993812071
REGULATION OF HAIR CYCLE	20	0.446944351	0.008048051
NEGATIVE REGULATION OF BONE REMODELING	11	-0.179252704	0.448017861
POSITIVE REGULATION OF RECEPTOR MEDIATED ENDOCYTOSIS	39	0.160198663	0.233157229
ESTABLISHMENT OF MITOTIC SPINDLE ORIENTATION	19	-0.117988968	0.517110226
POSITIVE REGULATION OF PROTEIN OLIGOMERIZATION	20	0.114572272	0.537731482
SPINDLE CHECKPOINT	25	-0.302471525	0.03858813
POSITIVE REGULATION OF STAT CASCADE	60	-0.103773623	0.31413044
LEUKOTRIENE BIOSYNTHETIC PROCESS	12	0.206982534	0.386287954
POSITIVE REGULATION OF SUBSTRATE ADHESION DEPENDENT CELL SPREADING	26	-0.098021114	0.530986955
POSITIVE REGULATION OF THYMOCYTE AGGREGATION	9	-0.177039664	0.49156376
CATECHOLAMINE METABOLIC PROCESS	31	0.163579917	0.274373295
POSITIVE REGULATION OF PROTEIN COMPLEX DISASSEMBLY	24	0.051388808	0.760692775
BLASTOCYST FORMATION	30	0.029276352	0.84567181
REGULATION OF WATER LOSS VIA SKIN	15	-0.039841635	0.849084738
NEGATIVE REGULATION OF REGULATED SECRETORY PATHWAY	20	0.012797287	0.944410116
CELL DIFFERENTIATION INVOLVED IN METANEPHROS DEVELOPMENT	10	0.079800985	0.760578606
POSITIVE REGULATION OF ALCOHOL BIOSYNTHETIC PROCESS	19	0.141593571	0.45721529
REGULATION OF EPITHELIAL CELL DIFFERENTIATION INVOLVED IN KIDNEY DEVELOPMENT	13	0.104856001	0.649510657
MAGNESIUM ION TRANSPORT	13	-0.306949104	0.142821967
PYRIMIDINE RIBONUCLEOSIDE METABOLIC PROCESS	27	-0.340664145	0.015262784
PROTEIN LOCALIZATION TO CENTROSOME	15	-0.115270704	0.573287667
POSITIVE REGULATION OF B CELL PROLIFERATION	33	0.046467767	0.746577248
POSITIVE REGULATION OF ORGANELLE ORGANIZATION	516	-0.043345	0.232511686
MUSCLE CELL PROLIFERATION	19	0.191821818	0.317994856
VENTRAL SPINAL CORD INTERNEURON SPECIFICATION	5	0.587155341	0.105596552
AMINO ACID TRANSMEMBRANE TRANSPORT	54	0.068043066	0.546642236
CELLULAR RESPONSE TO ACID CHEMICAL	158	-0.023187413	0.721639537
PHAGOCYTOSIS ENGULFMENT	16	-0.053397665	0.791653262
REGULATION OF TOLERANCE INDUCTION	16	-0.037318871	0.853987407
REGULATION OF CORTICOSTEROID HORMONE SECRETION	7	0.12776755	0.684106314
CELLULAR SENESCENCE	29	-0.299883682	0.031059664
SULFATION	14	0.323111734	0.128714494
REGULATION OF MEIOTIC CELL CYCLE	32	0.072033598	0.623188976
DNA DEALKYLATION	19	0.066122779	0.72794774
ACTIN FILAMENT BASED MOVEMENT	76	0.024284425	0.797271858
POSITIVE REGULATION OF EPITHELIAL CELL PROLIFERATION	138	-0.070488222	0.305232409

SARCOMERE ORGANIZATION	22	-0.319275622	0.04930697
POSITIVE REGULATION OF B CELL MEDIATED IMMUNITY	23	-0.16376312	0.313702205
OVULATION CYCLE	97	-0.018723459	0.82147239
NEGATIVE REGULATION OF NITRIC OXIDE METABOLIC PROCESS	10	0.327583665	0.196356369
VENTRICULAR SYSTEM DEVELOPMENT	21	0.080286657	0.657333691
ADULT LOCOMOTORY BEHAVIOR	69	0.009304836	0.925041218
REGULATION OF ACTIVATED T CELL PROLIFERATION	34	-0.121368935	0.371556352
POSITIVE REGULATION OF HOMEOSTATIC PROCESS	181	-0.042649579	0.481366123
RESPONSE TO GRAVITY	8	0.109815488	0.70798679
REGULATION OF IMMUNOGLOBULIN SECRETION	13	-0.098260219	0.657287946
ACETYL COA METABOLIC PROCESS	24	-0.066652824	0.685024207
CREATINE METABOLIC PROCESS	7	-0.274159673	0.327449599
REGULATION OF CELLULAR COMPONENT BIOGENESIS	678	0.045496623	0.160509521
POSITIVE REGULATION OF MACROPHAGE DERIVED FOAM CELL DIFFERENTIATION	11	0.531207422	0.012653004
IRON ION TRANSPORT	49	-0.005037263	0.965636224
REGULATION OF RECEPTOR BINDING	16	0.068215421	0.741841126
POSITIVE REGULATION OF POTASSIUM ION TRANSPORT	32	-0.162966637	0.238597207
POSITIVE REGULATION OF NERVOUS SYSTEM DEVELOPMENT	374	0.031850248	0.459937626
REGULATION OF CARDIAC MUSCLE CELL PROLIFERATION	27	0.129369373	0.419328565
CELLULAR RESPONSE TO OXYGEN CONTAINING COMPOUND	702	-0.022304284	0.478562317
REGULATION OF INTERLEUKIN 10 SECRETION	8	0.477930328	0.06612445
REGULATION OF LAMELLIPODIUM ASSEMBLY	26	0.286964565	0.083025043
CELLULAR KETONE METABOLIC PROCESS	59	0.126654438	0.242475569
CRANIAL NERVE DEVELOPMENT	36	0.018424269	0.893037502
WNT SIGNALING PATHWAY CALCIUM MODULATING PATHWAY	36	-0.107034516	0.420289218
EPIDERMAL GROWTH FACTOR RECEPTOR SIGNALING PATHWAY	53	-0.050732154	0.648500492
CELL PROLIFERATION IN FOREBRAIN	23	0.05947497	0.730400109
RESPONSE TO MURAMYL DIPEPTIDE	14	-0.32382433	0.094914303
REGULATION OF POSITIVE CHEMOTAXIS	22	0.060550386	0.731288205
REGULATION OF IMMUNE RESPONSE	680	-0.027717309	0.385207222
NEGATIVE REGULATION OF NOTCH SIGNALING PATHWAY	27	0.214862671	0.1773333
MEMBRANE LIPID CATABOLIC PROCESS	18	0.203135246	0.296815545
POSITIVE REGULATION OF NEUROBLAST PROLIFERATION	17	0.049200947	0.80596451
CARTILAGE MORPHOGENESIS	10	0.410815319	0.104267591
PEPTIDE SECRETION	43	0.114572876	0.366751723
THIOESTER METABOLIC PROCESS	70	0.01136441	0.907901515
NEGATIVE REGULATION OF INTRINSIC APOPTOTIC SIGNALING PATHWAY IN RESPONSE TO DNA DAMAGE	25	-0.068407888	0.670576025
REGULATION OF T HELPER 2 CELL DIFFERENTIATION	10	0.044783572	0.863734481
NEGATIVE REGULATION OF CELLULAR AMIDE METABOLIC PROCESS	125	0.050746528	0.494247267
PURINE RIBONUCLEOSIDE BISPHOSPHATE METABOLIC PROCESS	17	0.103452316	0.607924413
PEPTIDYL CYSTEINE MODIFICATION	19	-0.132907122	0.460784565
LEUKOCYTE DIFFERENTIATION	249	0.005639948	0.914180542
RESPONSE TO EXOGENOUS DSRNA	34	-0.049640099	0.720511106
MORPHOGENESIS OF EMBRYONIC EPITHELIUM	127	-0.116476249	0.099891516
SKELETAL MUSCLE CONTRACTION	24	0.188784852	0.268142492
POSITIVE REGULATION OF HORMONE METABOLIC PROCESS	8	0.012219955	0.96639199
TONGUE DEVELOPMENT	16	-0.220049073	0.251435936
BONE GROWTH	17	0.237206124	0.235886947
ACTIN POLYMERIZATION OR DEPOLYMERIZATION	30	-0.229060651	0.100292452
UV PROTECTION	10	-0.264123106	0.257945698
PROTEIN MODIFICATION BY SMALL PROTEIN REMOVAL	102	-0.115044024	0.145597847
REGULATION OF NITRIC OXIDE BIOSYNTHETIC PROCESS	47	0.191197282	0.116187182
PHOSPHATIDYLGLYCEROL METABOLIC PROCESS	24	0.150472608	0.377369623
PYRIMIDINE NUCLEOSIDE BIOSYNTHETIC PROCESS	28	-0.286199008	0.043815995
TRANSLATIONAL TERMINATION	89	-0.179540761	0.029013546
CYTOKINETIC PROCESS	12	-0.450060367	0.031702824
ENDOCARDIAL CUSHION MORPHOGENESIS	21	0.136967801	0.450143383
REGULATION OF INNATE IMMUNE RESPONSE	308	-0.111559099	0.014927399
VESICLE MEDIATED TRANSPORT	1053	0.031689266	0.228053193
INTERLEUKIN 1 PRODUCTION	10	0.14244886	0.588266153
MODULATION BY HOST OF VIRAL PROCESS	18	0.093566333	0.632468493
REGULATION OF DNA REPAIR	70	0.012793929	0.896410456
REGULATION OF MEMBRANE LIPID DISTRIBUTION	31	0.541421057	8.17E-05
REFLEX	18	-0.249396187	0.159289363

LEUKOCYTE MEDIATED IMMUNITY	128	-0.0459563	0.522414587
ORGANOPHOSPHATE BIOSYNTHETIC PROCESS	391	-0.077910048	0.058358393
NEGATIVE REGULATION OF SERINE TYPE PEPTIDASE ACTIVITY	6	-0.020634317	0.950504722
PROTEIN LOCALIZATION TO CELL PERIPHERY	137	0.098669057	0.167274139
REGULATION OF PROTEIN GLYCOSYLATION	13	-0.169181047	0.430191174
NEGATIVE REGULATION OF FIBROBLAST PROLIFERATION	26	-0.220974728	0.141790212
REGULATION OF MUSCLE TISSUE DEVELOPMENT	93	0.152031352	0.080215069
ORGANIC ACID BIOSYNTHETIC PROCESS	218	-0.015040621	0.786858519
NEGATIVE REGULATION OF DNA DEPENDENT DNA REPLICATION	16	0.122942605	0.555898594
REGULATION OF LEUKOCYTE MIGRATION	131	-0.041421978	0.560198175
ORGAN MATURATION	18	0.036982026	0.84902734
REGULATION OF CELLULAR RESPONSE TO HEAT	75	-0.202589245	0.023549382
POSITIVE REGULATION OF ACUTE INFLAMMATORY RESPONSE	24	0.005587587	0.973358756
GASTRULATION WITH MOUTH FORMING SECOND	26	0.037736123	0.81558939
ENDOCARDIUM DEVELOPMENT	10	0.395745156	0.13579103
ENDOTHELIAL CELL DIFFERENTIATION	64	-0.064876531	0.520450046
NEGATIVE REGULATION OF CELLULAR PROTEIN LOCALIZATION	124	-0.131460298	0.065131464
INSEMINATION	9	0.141666445	0.609127797
REGULATION OF NUCLEOBASE CONTAINING COMPOUND TRANSPORT	12	0.173446211	0.475152864
PHOSPHATIDYLINOSITOL 3 PHOSPHATE BIOSYNTHETIC PROCESS	45	0.197565323	0.111381388
CYCLIC NUCLEOTIDE CATABOLIC PROCESS	16	0.12244886	0.556406087
RESPONSE TO EPIDERMAL GROWTH FACTOR	28	-0.261194243	0.067081883
POSITIVE REGULATION OF TRANSMEMBRANE TRANSPORT	106	-0.201532523	0.007321296
REGULATION OF NUCLEOTIDE CATABOLIC PROCESS	33	0.150808017	0.300066247
POSITIVE REGULATION OF CARBOHYDRATE METABOLIC PROCESS	65	0.042805258	0.676268274
WNT SIGNALING PATHWAY	313	-0.05030487	0.276180502
REGULATION OF NEUROTRANSMITTER LEVELS	150	0.066790114	0.325499283
POSITIVE REGULATION OF STRESS ACTIVATED PROTEIN KINASE SIGNALING CASCADE	119	-0.0387697	0.603345653
PEPTIDYL ARGININE MODIFICATION	13	-0.121534248	0.578932158
SPECIFICATION OF ORGAN IDENTITY	12	0.314164532	0.182217263
REGULATION OF CELLULAR AMINE METABOLIC PROCESS	74	-0.091918856	0.323329576
RRNA TRANSCRIPTION	18	-0.372872851	0.029023363
POSITIVE REGULATION OF LIPID TRANSPORT	39	0.095975699	0.470891004
ANATOMICAL STRUCTURE ARRANGEMENT	16	-0.133859048	0.497390655
EPITHELIAL TO MESENCHYMAL TRANSITION	54	0.094207501	0.405232041
PATHWAY RESTRICTED SMAD PROTEIN PHOSPHORYLATION	11	0.303031945	0.226457037
SINGLE ORGANISM MEMBRANE BUDDING	69	-0.123114133	0.197593131
NEGATIVE REGULATION OF MAPK CASCADE	135	-0.049778022	0.476535913
SPONGIOTROPHOBLAST LAYER DEVELOPMENT	13	0.387746724	0.078151672
NEGATIVE REGULATION OF NERVOUS SYSTEM DEVELOPMENT	232	-0.038707014	0.470605311
HISTONE H2A MONOUBIQUITINATION	12	0.260009355	0.279257912
REGULATION OF EXTRACELLULAR MATRIX ORGANIZATION	25	0.286965794	0.088064135
CELL DIFFERENTIATION IN SPINAL CORD	38	0.07820581	0.561672092
MITOTIC G2 M TRANSITION CHECKPOINT	18	-0.037750498	0.843484817
POSITIVE REGULATION OF INTERLEUKIN 8 SECRETION	10	-0.103798746	0.679331758
PYRIMIDINE NUCLEOBASE METABOLIC PROCESS	19	-0.228314521	0.190274295
RNA METHYLATION	51	-0.023921536	0.833972481
PURINE CONTAINING COMPOUND SALVAGE	15	-0.403364427	0.028261096
INNER EAR RECEPTOR STEREOCILIUM ORGANIZATION	15	0.109175574	0.612072976
NEGATIVE REGULATION OF CATION TRANSMEMBRANE TRANSPORT	53	-0.088131083	0.42328633
REGULATION OF PHOSPHOLIPID METABOLIC PROCESS	56	-0.061415841	0.569241203
NEGATIVE REGULATION OF CYTOPLASMIC TRANSPORT	104	-0.055067763	0.488261741
POSITIVE REGULATION OF CELLULAR RESPONSE TO TRANSFORMING GROWTH FACTOR BETA STIMULUS	24	0.077085149	0.649741263
AXON REGENERATION	24	-0.124471689	0.442164399
MICROTUBULE BASED PROCESS	452	-0.053594349	0.165152767
CALCIUM MEDIATED SIGNALING	78	-0.16500371	0.061860284
LOW DENSITY LIPOPROTEIN PARTICLE CLEARANCE	11	0.618910604	0.009141259
SPERM MOTILITY	32	0.238135368	0.102186048
NEGATIVE REGULATION OF SEQUENCE SPECIFIC DNA BINDING TRANSCRIPTION FACTOR ACTIVITY	124	-0.039403964	0.58983195
NEGATIVE REGULATION OF PROTEIN CATABOLIC PROCESS	105	-0.035951711	0.650896283
CELLULAR AMINO ACID METABOLIC PROCESS	293	-0.070486391	0.137526742
POSITIVE REGULATION OF DNA BINDING	41	0.164722067	0.20821582
NUCLEOTIDE TRANSPORT	23	-0.286563408	0.067774105

REGULATION OF HAIR FOLLICLE DEVELOPMENT	14	0.464328253	0.020075042
CRISTAE FORMATION	11	0.201095569	0.417954495
SYNAPTIC VESICLE RECYCLING	21	0.110210746	0.5443309
POSITIVE REGULATION OF NUCLEOSIDE METABOLIC PROCESS	21	0.213047236	0.245786134
DORSAL VENTRAL PATTERN FORMATION	73	0.163030849	0.095891574
REGULATION OF VITAMIN METABOLIC PROCESS	10	0.153051065	0.562091623
AEROBIC RESPIRATION	46	-0.351670265	0.001049694
CELLULAR RESPONSE TO INSULIN STIMULUS	133	-0.017908734	0.800765179
SEQUESTERING OF METAL ION	10	-0.066871945	0.792690814
COPPER ION TRANSPORT	17	-0.071097726	0.715624524
MEMBRANE ASSEMBLY	23	-0.235251771	0.13362178
REGULATION OF DNA DAMAGE RESPONSE SIGNAL TRANSDUCTION BY P53 CLASS MEDIATOR	28	-0.048952699	0.74887143
LIPID DIGESTION	15	0.456455002	0.022062312
POSITIVE REGULATION OF CELLULAR COMPONENT ORGANIZATION	1026	-0.007087216	0.788485184
POSITIVE REGULATION OF G1 S TRANSITION OF MITOTIC CELL CYCLE	23	-0.018871562	0.911600856
POSITIVE REGULATION OF GENE EXPRESSION	1541	0.003367495	0.878362779
NEUROMUSCULAR PROCESS CONTROLLING BALANCE	41	0.001392134	0.991320102
ORGAN OR TISSUE SPECIFIC IMMUNE RESPONSE	24	-0.235910003	0.130143125
REGULATION OF CARDIAC MUSCLE CONTRACTION BY REGULATION OF THE RELEASE OF SEQUESTERED CALCIUM ION	18	-0.40287061	0.013025868
NEGATIVE REGULATION OF ORGANELLE ASSEMBLY	20	0.020802726	0.909903119
RESPONSE TO INTERFERON BETA	22	-0.031641132	0.855065771
REGULATION OF ORGAN FORMATION	29	0.044380619	0.772282719
FOREBRAIN DEVELOPMENT	308	0.008651732	0.854496477
RHODOPSIN MEDIATED SIGNALING PATHWAY	8	0.142109738	0.629253538
ACTIVATION OF CYSTEINE TYPE ENDOPEPTIDASE ACTIVITY	86	-0.072763876	0.402658687
REGULATION OF MESONEPHROS DEVELOPMENT	23	-0.159904004	0.32661338
CARBOHYDRATE DERIVATIVE METABOLIC PROCESS	908	-0.07263802	0.00846517
REGULATION OF CHROMATIN BINDING	15	-0.211167651	0.28142146
STEM CELL PROLIFERATION	55	-0.069689755	0.521041687
NAD METABOLIC PROCESS	47	0.108350165	0.373779172
REGULATION OF EXTRACELLULAR MATRIX ASSEMBLY	11	0.310019498	0.218313768
PROTEIN LOCALIZATION TO LYSOSOME	20	0.216798181	0.25900969
MRNA MODIFICATION	11	0.485002511	0.046331495
SOMATIC DIVERSIFICATION OF IMMUNOGLOBULINS	26	-0.099887894	0.524358803
ERBB2 SIGNALING PATHWAY	36	0.704729937	1.02E-09
AMEBOIDAL TYPE CELL MIGRATION	139	0.011326169	0.871224809
HEMATOPOIETIC STEM CELL DIFFERENTIATION	11	-0.357996617	0.107842463
SPINAL CORD MOTOR NEURON DIFFERENTIATION	26	0.018294589	0.909631188
MONOCARBOXYLIC ACID METABOLIC PROCESS	399	0.062585455	0.13587854
CELLULAR RESPONSE TO TOPOLOGICALLY INCORRECT PROTEIN	113	-0.17377013	0.0184978
AMINOGLYCAN BIOSYNTHETIC PROCESS	95	-0.151718416	0.060325956
DICARBOXYLIC ACID METABOLIC PROCESS	86	-0.042872711	0.624379335
NEGATIVE REGULATION OF ASTROCYTE DIFFERENTIATION	12	0.062344299	0.794188709
POSITIVE REGULATION OF PROTEASOMAL PROTEIN CATABOLIC PROCESS	91	-0.029877545	0.726570345
REGULATION OF ENERGY HOMEOSTASIS	14	0.178888466	0.41992228
REGULATION OF MEMBRANE POTENTIAL	277	0.046249316	0.355399092
POSITIVE REGULATION OF RELEASE OF CYTOCHROME C FROM MITOCHONDRIA	27	-0.020196871	0.897545439
PEPTIDYL PROLINE HYDROXYLATION	11	0.577738535	0.016687096
MULTICELLULAR ORGANISMAL RESPONSE TO STRESS	52	0.067835886	0.55524685
NEGATIVE REGULATION OF SMALL GTPASE MEDIATED SIGNAL TRANSDUCTION	34	-0.112805863	0.407386019
ESTABLISHMENT OF LOCALIZATION BY MOVEMENT ALONG MICROTUBULE	89	-0.05899336	0.491659514
AMINO ACID BETAINE TRANSPORT	14	0.076029936	0.731785854
AMMONIUM TRANSMEMBRANE TRANSPORT	19	0.035117639	0.85267256
REGULATION OF GROWTH	565	0.055293718	0.11868743
CATION TRANSPORT	613	0.026472787	0.434895286
GABAERGIC NEURON DIFFERENTIATION	9	0.234527704	0.408077416
MACROAUTOPHAGY	249	-0.049251782	0.340665391
FATTY ACID BETA OXIDATION USING ACYL COA DEHYDROGENASE	15	0.037367524	0.86063664
MEMBRANE DOCKING	64	-0.069528608	0.490237288
EXCITATORY POSTSYNAPTIC POTENTIAL	22	0.112825473	0.524215599
GLUTAMINE FAMILY AMINO ACID METABOLIC PROCESS	60	-0.12943659	0.204825318
REGULATION OF NUCLEASE ACTIVITY	22	-0.037360823	0.829043344
RESPONSE TO INORGANIC SUBSTANCE	414	-0.019943935	0.623212617

NEGATIVE REGULATION OF HISTONE ACETYLATION	14	-0.0307695	0.887303615
PHAGOLYSOSOME ASSEMBLY	7	0.651750168	0.097373065
MODULATION BY SYMBIONT OF HOST CELLULAR PROCESS	28	0.037852301	0.80815829
REGULATION OF VESICLE MEDIATED TRANSPORT	387	0.022558561	0.59396464
ORGANONITROGEN COMPOUND CATABOLIC PROCESS	290	0.006941151	0.886369114
REGULATION OF ASTROCYTE DIFFERENTIATION	24	0.254382156	0.137296274
REGULATION OF HYDROGEN PEROXIDE INDUCED CELL DEATH	18	-0.021110195	0.912457242
ADP METABOLIC PROCESS	43	0.058298887	0.644267344
POSITIVE REGULATION OF CELL PROLIFERATION	704	-0.003634358	0.908299214
REGULATION OF SODIUM ION TRANSPORT	66	0.131234298	0.202490449
POSITIVE REGULATION OF TUMOR NECROSIS FACTOR SUPERFAMILY CYTOKINE PRODUCTION	52	-0.05030914	0.654090974
NEGATIVE REGULATION OF G PROTEIN COUPLED RECEPTOR PROTEIN SIGNALING PATHWAY	34	0.051664913	0.715577913
HISTONE DEUBIQUITINATION	20	0.116583277	0.531152722
GLUCOSAMINE CONTAINING COMPOUND METABOLIC PROCESS	22	0.178497988	0.315651971
ORGANIC ACID TRANSPORT	212	-0.023599268	0.675006999
FOREBRAIN REGIONALIZATION	18	-0.132820441	0.474427488
FOREBRAIN GENERATION OF NEURONS	54	0.20425508	0.07334991
CALCIUM DEPENDENT CELL CELL ADHESION VIA PLASMA MEMBRANE CELL ADHESION MOLECULES	19	0.240050494	0.202371439
PANCREAS DEVELOPMENT	59	0.005415128	0.959573311
LEUKOCYTE CHEMOTAXIS	96	0.019510156	0.816519695
GLUCOSE 6 PHOSPHATE METABOLIC PROCESS	21	-0.006416141	0.971307162
MITOCHONDRIAL TRANSMEMBRANE TRANSPORT	55	-0.102847976	0.338745156
NUCLEAR PORE ORGANIZATION	15	-0.356084496	0.055569357
CELLULAR RESPONSE TO NITRIC OXIDE	12	-0.188877182	0.398835842
INTRA S DNA DAMAGE CHECKPOINT	10	0.15733901	0.555646037
REGULATION OF SYNAPSE STRUCTURE OR ACTIVITY	193	0.044822232	0.453320737
HISTONE H3 K9 MODIFICATION	12	0.024824333	0.916744763
RESPONSE TO INTERFERON ALPHA	18	-0.33530036	0.053045719
NEGATIVE REGULATION OF TRANSLATIONAL INITIATION	21	-0.119484473	0.489925573
REGULATION OF TOLL LIKE RECEPTOR SIGNALING PATHWAY	42	0.120212368	0.35163019
CARDIAC CONDUCTION SYSTEM DEVELOPMENT	10	-0.123059806	0.623723017
REGULATION OF GLOMERULAR FILTRATION	10	-0.287387868	0.216572742
REGULATION OF FATTY ACID METABOLIC PROCESS	73	0.02046913	0.831804445
CYTOCHROME COMPLEX ASSEMBLY	24	-0.192521523	0.220413217
RESPONSE TO METAL ION	288	-0.010526863	0.828379223
CELL SUBSTRATE ADHESION	146	-0.038881391	0.564192392
NEGATIVE REGULATION OF DEVELOPMENTAL GROWTH	78	0.030464365	0.744381968
NEGATIVE REGULATION OF INTRACELLULAR SIGNAL TRANSDUCTION	399	-0.03782055	0.358220006
ENDOCARDIAL CUSHION FORMATION	15	0.176082632	0.410948546
REGULATION OF NUCLEAR CELL CYCLE DNA REPLICATION	11	-0.184207631	0.430990422
ORGANIC HYDROXY COMPOUND TRANSPORT	110	0.032559693	0.679453955
MITOCHONDRIAL CALCIUM ION HOMEOSTASIS	14	-0.126597809	0.547027186
REPLACEMENT OSSIFICATION	22	-0.153700916	0.356276091
REGULATION OF ACTIN FILAMENT BASED PROCESS	280	-0.014044131	0.775287478
WOUND HEALING	407	-0.045510115	0.263450584
MITOTIC CELL CYCLE ARREST	13	-0.102892036	0.640841056
HEXOSE CATABOLIC PROCESS	41	0.024223746	0.850536011
GLUTAMINE METABOLIC PROCESS	23	-0.22939081	0.153251502
RESPONSE TO NERVE GROWTH FACTOR	34	0.014082463	0.920369099
NEGATIVE REGULATION OF TELOMERASE ACTIVITY	14	-0.162634836	0.43366773
RNA PROCESSING	791	-0.121262715	3.12E-05
REGULATION OF IRE1 MEDIATED UNFOLDED PROTEIN RESPONSE	13	-0.039804173	0.859496367
POSITIVE REGULATION OF FATTY ACID BIOSYNTHETIC PROCESS	13	0.092063454	0.689621971
SYNAPTIC VESICLE CYCLE	72	0.020562838	0.832191836
NUCLEOBASE CONTAINING SMALL MOLECULE METABOLIC PROCESS	473	-0.103948487	0.005300491
POSITIVE REGULATION OF INTERLEUKIN 1 SECRETION	18	0.114538116	0.558081764
REGULATION OF CYTOKINE PRODUCTION INVOLVED IN IMMUNE RESPONSE	50	0.098331186	0.402875631
POSITIVE REGULATION OF LEUKOCYTE PROLIFERATION	115	-0.093734482	0.210017795
PSEUDOURIDINE SYNTHESIS	16	-0.242029211	0.199069844
CELLULAR PROTEIN COMPLEX ASSEMBLY	309	-0.028011828	0.548889534
DEFENSE RESPONSE	917	0.029907078	0.285948839
POLY A MRNA EXPORT FROM NUCLEUS	11	-0.292010122	0.19077971
PHOSPHOLIPID BIOSYNTHETIC PROCESS	208	-0.047589789	0.399484362

NEGATIVE REGULATION OF LEUKOCYTE APOPTOTIC PROCESS	38	0.0025701	0.98457593
POSITIVE REGULATION OF HYDROLASE ACTIVITY	776	-0.020911819	0.486125547
CELLULAR RESPONSE TO INTERLEUKIN 4	25	0.143094973	0.390393727
PORPHYRIN CONTAINING COMPOUND METABOLIC PROCESS	32	-0.275082251	0.037664259
SPERM CAPACITATION	11	0.153833373	0.540099906
POSITIVE REGULATION OF TELOMERASE ACTIVITY	26	0.305221345	0.062499949
NEGATIVE REGULATION OF INTERLEUKIN 6 PRODUCTION	27	-0.025286554	0.871764178
CELLULAR MONOVALENT INORGANIC CATION HOMEOSTASIS	78	0.13581475	0.151393306
RESPONSE TO METHYLMERCURY	10	-0.058560869	0.818367133
REGULATION OF CENTRIOLE REPLICATION	12	0.102999928	0.669115399
POSITIVE REGULATION OF MULTICELLULAR ORGANISMAL METABOLIC PROCESS	18	-0.34574837	0.040674739
REGULATION OF ACUTE INFLAMMATORY RESPONSE	60	0.068576887	0.522042974
CYTOKINE PRODUCTION	99	-0.009788189	0.90533971
TOLL LIKE RECEPTOR SIGNALING PATHWAY	77	0.006311814	0.946228708
POSITIVE REGULATION OF INTRACELLULAR TRANSPORT	320	-0.05887251	0.196769359
FOREBRAIN CELL MIGRATION	56	-0.007235218	0.947243185
DEMETHYLATION	47	0.047200498	0.695681358
CELL MOTILITY	713	-0.006774867	0.828869093
POSITIVE REGULATION OF ENDOTHELIAL CELL MIGRATION	63	0.081710605	0.435517539
DECIDUALIZATION	19	0.247054542	0.198853374
REGULATION OF MONONUCLEAR CELL MIGRATION	15	0.01823813	0.931476178
CERAMIDE BIOSYNTHETIC PROCESS	37	-0.087617103	0.505489792
NEGATIVE REGULATION OF ORGAN GROWTH	20	-0.233938575	0.167813339
NEGATIVE REGULATION OF RESPONSE TO BIOTIC STIMULUS	28	-0.215830407	0.136144961
ORGANOPHOSPHATE ESTER TRANSPORT	75	0.184276403	0.05813433
MICROTUBULE CYTOSKELETON ORGANIZATION	305	-0.023477017	0.61803234
HISTONE MONOUBIQUITINATION	23	-0.093043681	0.577212563
REGULATION OF CELL CYCLE G2 M PHASE TRANSITION	57	-0.020134543	0.852192392
EMBRYONIC VISCEROCRANIUM MORPHOGENESIS	10	0.138175788	0.598818494
SOMATIC CELL DNA RECOMBINATION	30	-0.097172947	0.505985367
PIGMENTATION	75	0.061117486	0.523208854
POSITIVE REGULATION OF PROTEIN LOCALIZATION TO CELL PERIPHERY	34	0.076580527	0.590598846
POSITIVE REGULATION OF TRANSLATIONAL INITIATION	20	-0.251422959	0.141042189
NEGATIVE REGULATION OF CELL AGING	17	-0.083498612	0.668054434
B CELL MEDIATED IMMUNITY	54	0.109529503	0.334743413
AORTA DEVELOPMENT	38	-0.056895493	0.664329354
CELL DIFFERENTIATION INVOLVED IN EMBRYONIC PLACENTA DEVELOPMENT	25	-0.031590562	0.845929064
ASYMMETRIC CELL DIVISION	12	-0.07600914	0.74273809
SPECIFICATION OF SYMMETRY	97	-0.042651266	0.605124346
PROTEIN LOCALIZATION TO ENDOPLASMIC RETICULUM	120	-0.166364829	0.020219438
BILE ACID BIOSYNTHETIC PROCESS	12	0.00313994	0.989402476
NEGATIVE REGULATION OF COAGULATION	40	0.104081252	0.428327056
HISTONE EXCHANGE	50	-0.167975547	0.126731222
LIPID HOMEOSTASIS	86	0.217200997	0.016206985
CELLULAR RESPIRATION	127	-0.191401295	0.00541018
NUCLEOTIDE EXCISION REPAIR DNA DAMAGE RECOGNITION	23	0.390172382	0.021751528
REGULATION OF ENDOTHELIAL CELL DEVELOPMENT	11	0.358882574	0.139013329
B CELL DIFFERENTIATION	71	0.02078306	0.831595243
NEURAL PRECURSOR CELL PROLIFERATION	60	0.035545905	0.738715093
NUCLEAR TRANSPORT	327	-0.052658602	0.244080824
PEPTIDE METABOLIC PROCESS	509	-0.170386498	1.45E-06
NEGATIVE REGULATION OF CELL SUBSTRATE ADHESION	49	-0.200858622	0.067522066
REGULATION OF EPITHELIAL CELL DIFFERENTIATION	104	0.106807579	0.192122701
ENDOTHELIAL CELL MIGRATION	52	0.001303642	0.99084938
ORGANIC HYDROXY COMPOUND CATABOLIC PROCESS	62	0.070946469	0.500777306
GLYCEROLIPID METABOLIC PROCESS	298	0.028428604	0.554740695
PEPTIDYL AMINO ACID MODIFICATION	759	-0.033422814	0.269442643
SECRETION	483	-0.042138489	0.26097894
REGULATION OF SEQUESTERING OF TRIGLYCERIDE	10	0.360425262	0.151037216
NEGATIVE REGULATION OF FIBROBLAST GROWTH FACTOR RECEPTOR SIGNALING PATHWAY	11	-0.167542091	0.475836472
NEGATIVE REGULATION OF PROTEIN OLIGOMERIZATION	10	0.350429368	0.167304804
CELL ACTIVATION INVOLVED IN IMMUNE RESPONSE	113	-0.014187821	0.853818223
POSITIVE REGULATION OF VASODILATION	23	-0.135517386	0.408390613
HOMOCYSTEINE METABOLIC PROCESS	10	-0.064074116	0.801120144

NEGATIVE REGULATION OF HISTONE MODIFICATION	34	0.08875439	0.533202887
COFACTOR CATABOLIC PROCESS	15	0.161312334	0.457415164
PYRIMIDINE CONTAINING COMPOUND CATABOLIC PROCESS	28	0.038158336	0.806586216
NEGATIVE REGULATION OF DNA BINDING	44	0.104172052	0.407126476
VENTRAL SPINAL CORD DEVELOPMENT	34	0.152470487	0.288345937
REGULATION OF CIRCADIAN RHYTHM	85	-0.042237505	0.631733573
ANION TRANSPORT	409	0.046655961	0.259394933
CELL CELL ADHESION	499	-0.002556586	0.94523899
MORPHOGENESIS OF AN ENDOTHELIUM	15	-0.039161313	0.851609106
GUANOSINE CONTAINING COMPOUND METABOLIC PROCESS	43	-0.158131949	0.184693172
POSITIVE REGULATION OF RELEASE OF SEQUESTERED CALCIUM ION INTO CYTOSOL	33	-0.265739838	0.043421048
REGULATION OF CYCLIC NUCLEOTIDE METABOLIC PROCESS	114	0.038204341	0.621891334
CHONDROITIN SULFATE PROTEOGLYCAN BIOSYNTHETIC PROCESS	28	-0.068564045	0.652259711
GLANDULAR EPITHELIAL CELL DEVELOPMENT	16	-0.041185622	0.838972964
RESPONSE TO MAGNESIUM ION	19	-0.055278778	0.765428504
CELLULAR RESPONSE TO ATP	12	-0.36057926	0.075706692
CHOLESTEROL EFFLUX	21	0.437519403	0.010266011
PROTEIN OLIGOMERIZATION	371	0.005299703	0.90201892
BETA CATENIN DESTRUCTION COMPLEX DISASSEMBLY	20	-0.205723139	0.231712274
INNER DYNEIN ARM ASSEMBLY	9	0.378259903	0.172726339
REGULATION OF EPITHELIAL CELL PROLIFERATION	250	-0.027460599	0.596411513
CHROMATIN ASSEMBLY OR DISASSEMBLY	151	-0.085854053	0.18969232
REGULATION OF NITRIC OXIDE SYNTHASE ACTIVITY	42	0.264216578	0.039356915
REGULATION OF ANDROGEN RECEPTOR SIGNALING PATHWAY	20	-0.175215665	0.314016993
LEFT RIGHT PATTERN FORMATION	20	0.233988788	0.207509264
REGULATION OF LIPID BIOSYNTHETIC PROCESS	108	0.144264152	0.073509955
RESPONSE TO XENOBIOTIC STIMULUS	80	0.048176813	0.602583948
REGULATION OF MACROPHAGE DERIVED FOAM CELL DIFFERENTIATION	22	0.457811312	0.007798042
EMBRYONIC DIGESTIVE TRACT MORPHOGENESIS	15	0.17516356	0.413635673
PLACENTA BLOOD VESSEL DEVELOPMENT	26	0.179573592	0.276130463
POSITIVE REGULATION OF GUANYLATE CYCLASE ACTIVITY	6	0.169748402	0.615120085
MELANIN METABOLIC PROCESS	10	0.08503074	0.745545674
POSITIVE REGULATION OF RESPONSE TO EXTERNAL STIMULUS	249	0.015692542	0.764751903
POSITIVE REGULATION OF CELL CYCLE G2 M PHASE TRANSITION	17	-0.023599321	0.904867156
REGULATION OF REMOVAL OF SUPEROXIDE RADICALS	11	-0.170339632	0.464491209
NEGATIVE REGULATION OF STEM CELL PROLIFERATION	16	-0.09819034	0.622351541
AGING	235	-0.041093061	0.440560471
REGULATION OF VACUOLAR TRANSPORT	29	-0.048559305	0.746764724
CELLULAR RESPONSE TO ELECTRICAL STIMULUS	12	-0.020223963	0.931459848
REGULATION OF OXIDATIVE PHOSPHORYLATION	15	0.067502186	0.752725095
NEGATIVE REGULATION OF CHROMOSOME SEGREGATION	27	-0.218871181	0.134129708
NEGATIVE REGULATION OF EXTRINSIC APOPTOTIC SIGNALING PATHWAY	92	-0.095699869	0.251508053
REGULATION OF BONE DEVELOPMENT	16	-0.024672189	0.903478929
POSITIVE REGULATION OF T CELL MEDIATED CYTOTOXICITY	14	-0.179537195	0.387339095
REGULATION OF LEUKOCYTE MEDIATED CYTOTOXICITY	44	0.00694258	0.955237159
REGULATION OF CHOLESTEROL BIOSYNTHETIC PROCESS	13	0.218568494	0.342009222
POLYSACCHARIDE CATABOLIC PROCESS	23	-0.035720082	0.832900121
NEGATIVE REGULATION OF AUTOPHAGY	48	-0.038981789	0.739399367
INNER CELL MASS CELL PROLIFERATION	11	-0.251262504	0.283636247
POSITIVE REGULATION OF MRNA SPLICING VIA SPLICEOSOME	13	0.041225639	0.857008029
POSTTRANSCRIPTIONAL REGULATION OF GENE EXPRESSION	423	-0.07440546	0.060842977
POSITIVE REGULATION OF DNA DEPENDENT DNA REPLICATION	11	-0.164939705	0.482697201
STARTLE RESPONSE	20	0.213571754	0.246631387
MOTOR NEURON AXON GUIDANCE	26	-0.109012848	0.484529029
TRANSCRIPTION FROM RNA POLYMERASE II PROMOTER	661	0.001369088	0.966446321
PYRIMIDINE RIBONUCLEOSIDE CATABOLIC PROCESS	8	-0.080295401	0.776653011
RESPONSE TO ENDOGENOUS STIMULUS	1247	-0.007704652	0.749713144
HYPOTHALAMUS DEVELOPMENT	17	0.207093935	0.303705574
PATTERN RECOGNITION RECEPTOR SIGNALING PATHWAY	98	-0.044282065	0.589369479
POSITIVE REGULATION OF MEIOTIC CELL CYCLE	11	0.152942627	0.544512242
LATERAL MESODERM DEVELOPMENT	10	0.188699723	0.474868633
POSITIVE REGULATION OF CELL MATRIX ADHESION	38	-0.110794121	0.389457736
EMBRYO DEVELOPMENT	790	-0.01254376	0.674041159
REGULATION OF STRIATED MUSCLE CONTRACTION	67	-0.127055026	0.188773984
REGULATION OF LEUKOCYTE DIFFERENTIATION	203	-0.033487093	0.559405778

REGULATION OF TRANSCRIPTION ELONGATION FROM RNA POLYMERASE II PROMOTER	23	-0.14500408	0.376858944
WATER TRANSPORT	11	0.027755149	0.910914735
ESTABLISHMENT OF CELL POLARITY	77	-0.063061261	0.493415877
BIOTIN METABOLIC PROCESS	14	0.078677235	0.722418128
STEM CELL DIVISION	28	0.23131173	0.139724757
REACTIVE OXYGEN SPECIES BIOSYNTHETIC PROCESS	20	0.174356953	0.347227377
POSITIVE REGULATION OF NITRIC OXIDE SYNTHASE BIOSYNTHETIC PROCESS	12	-0.03498787	0.881258746
DIGESTIVE SYSTEM DEVELOPMENT	129	0.017274481	0.811898188
POSITIVE REGULATION OF STEROID BIOSYNTHETIC PROCESS	11	0.166648437	0.504791946
DNA CONFORMATION CHANGE	239	-0.049972253	0.343248365
DETECTION OF MECHANICAL STIMULUS	31	0.178262809	0.236506256
SEXUAL REPRODUCTION	540	0.101363873	0.005440467
EPIDERMAL CELL DIFFERENTIATION	98	0.087597074	0.298498703
SYNAPTONEMAL COMPLEX ORGANIZATION	15	0.594355307	0.000724665
BASE EXCISION REPAIR	37	0.029353362	0.828419857
CELLULAR CARBOHYDRATE CATABOLIC PROCESS	29	-0.092043943	0.535435032
NEGATIVE REGULATION OF TYPE 2 IMMUNE RESPONSE	10	0.102898978	0.694823011
REGULATION OF NUCLEOSIDE METABOLIC PROCESS	46	0.056751289	0.642143755
TAXIS	394	0.093330704	0.027708541
CITRULLINE METABOLIC PROCESS	7	-0.132156986	0.655820337
NEGATIVE REGULATION OF AXON GUIDANCE	26	0.197034071	0.229525158
ORGAN FORMATION	31	0.201618213	0.176941068
NEGATIVE REGULATION OF CALCIUM ION TRANSPORT INTO CYTOSOL	16	-0.150952039	0.438673541
REGULATION OF CARDIAC MUSCLE CELL MEMBRANE REPOLARIZATION	16	-0.16118421	0.411928447
RESPONSE TO GLUCAGON	41	0.078793474	0.543241031
NEGATIVE REGULATION OF TISSUE REMODELING	15	-0.075943755	0.714458593
MEMBRANE TUBULATION	10	0.278754965	0.277563019
EMBRYONIC PLACENTA DEVELOPMENT	79	0.232444672	0.013714421
SMALL MOLECULE BIOSYNTHETIC PROCESS	369	-0.015228239	0.723077283
ANATOMICAL STRUCTURE MATURATION	38	-0.014145009	0.914950073
REGULATION OF AMYLOID PRECURSOR PROTEIN CATABOLIC PROCESS	9	0.719859417	0.000401398
CELLULAR EXTRAVASATION	25	-0.159653122	0.304333231
REGULATION OF TELOMERE MAINTENANCE	62	-0.084867683	0.405465813
REGULATION OF EXTRINSIC APOPTOTIC SIGNALING PATHWAY VIA DEATH DOMAIN RECEPTORS	52	-0.232169453	0.028413083
NEGATIVE REGULATION OF WOUND HEALING	49	0.090841157	0.444058985
REGULATION OF IMMUNOGLOBULIN PRODUCTION	40	0.029014069	0.823771144
FATTY ACYL COA METABOLIC PROCESS	41	0.036993357	0.77407287
NEGATIVE REGULATION OF MUSCLE CELL APOPTOTIC PROCESS	27	0.085116396	0.594538663
GPI ANCHOR METABOLIC PROCESS	29	-0.195107047	0.172306514
POSITIVE REGULATION OF HISTONE DEACETYLATION	14	-0.346089095	0.070368933
BLOOD COAGULATION FIBRIN CLOT FORMATION	21	0.214618759	0.233057949
POSITIVE REGULATION OF INSULIN SECRETION	53	-0.073356034	0.506771432
NEGATIVE REGULATION OF NEUROTRANSMITTER TRANSPORT	14	-0.202860127	0.325192645
AMYLOID PRECURSOR PROTEIN METABOLIC PROCESS	10	-0.0502927	0.844028306
NUCLEIC ACID PHOSPHODIESTER BOND HYDROLYSIS	229	0.004545992	0.933551642
CHEMICAL HOMEOSTASIS WITHIN A TISSUE	10	0.314127113	0.239323025
REGULATION OF MYELOID CELL DIFFERENTIATION	164	-0.04101535	0.519117213
REGULATION OF RESPONSE TO EXTERNAL STIMULUS	789	0.008061972	0.787865631
REGULATION OF RNA POLYMERASE II TRANSCRIPTIONAL PREINITIATION COMPLEX ASSEMBLY	12	-0.173155622	0.439380311
RETROGRADE PROTEIN TRANSPORT ER TO CYTOSOL	16	-0.494978685	0.020459656
EPITHELIAL CELL CELL ADHESION	13	-0.249931738	0.235016378
REGULATION OF MITOCHONDRION ORGANIZATION	194	-0.16811277	0.002864467
POLYAMINE METABOLIC PROCESS	13	-0.31514516	0.114002046
POSITIVE REGULATION OF MUSCLE CONTRACTION	37	0.000746746	0.995575754
NEGATIVE REGULATION OF T CELL RECEPTOR SIGNALING PATHWAY	16	-0.130744071	0.509499096
PTERIDINE CONTAINING COMPOUND BIOSYNTHETIC PROCESS	18	-0.00784511	0.967507343
BLOOD VESSEL ENDOTHELIAL CELL MIGRATION	23	-0.052665445	0.754780452
POSITIVE REGULATION OF PROTEIN LOCALIZATION TO NUCLEUS	116	0.04466426	0.561343233
POSITIVE REGULATION OF BMP SIGNALING PATHWAY	28	0.023230416	0.881221475
REGULATION OF INTERFERON GAMMA BIOSYNTHETIC PROCESS	10	0.454805514	0.051944924
PROXIMAL DISTAL PATTERN FORMATION	28	-0.07518001	0.620677612
STRIATED MUSCLE CONTRACTION	79	-0.038982871	0.669885948
NEGATIVE REGULATION OF ANION TRANSPORT	28	0.102722127	0.513327335

NEGATIVE REGULATION OF CD4 POSITIVE ALPHA BETA T CELL DIFFERENTIATION	11	-0.145645238	0.536127309
ECTODERM DEVELOPMENT	21	0.193768955	0.287840242
HYALURONAN METABOLIC PROCESS	27	0.007332157	0.96294348
LEUKOCYTE MIGRATION	217	-0.052087462	0.345891631
NEGATIVE REGULATION OF INTRACELLULAR PROTEIN TRANSPORT	87	-0.074957722	0.385446935
MEIOSIS I	73	0.050078141	0.605330943
SENSORY PERCEPTION OF PAIN	58	0.231717602	0.033219237
NEGATIVE REGULATION OF GENE EXPRESSION EPIGENETIC	96	-0.055741889	0.499986306
ALDITOL METABOLIC PROCESS	15	-0.134172846	0.508506034
MITOCHONDRIAL ELECTRON TRANSPORT UBIQUINOL TO CYTOCHROME C	13	-0.364152029	0.061380271
REGULATION OF ANATOMICAL STRUCTURE SIZE	413	0.048329131	0.240588048
REGULATION OF CELL PROLIFERATION INVOLVED IN KIDNEY DEVELOPMENT	13	-0.257009436	0.213061416
POSITIVE REGULATION OF MITOCHONDRION ORGANIZATION	146	-0.124127397	0.059328759
POSITIVE REGULATION OF CATALYTIC ACTIVITY	1310	-0.025880198	0.271589018
POSITIVE REGULATION OF SIGNAL TRANSDUCTION BY P53 CLASS MEDIATOR	17	-0.021835837	0.911982437
PEPTIDYL LYSINE MODIFICATION	294	-0.090947582	0.053830664
METHYLATION DEPENDENT CHROMATIN SILENCING	8	-0.29389389	0.263881422
REGULATION OF LIPOPROTEIN LIPASE ACTIVITY	11	-0.331832907	0.132575987
HORMONE MEDIATED SIGNALING PATHWAY	134	0.126141801	0.081669922
ANDROGEN BIOSYNTHETIC PROCESS	9	-0.043547325	0.871803208
NEGATIVE REGULATION OF PROTEIN BINDING	74	-0.071442195	0.445576644
POSITIVE REGULATION OF OLIGODENDROCYTE DIFFERENTIATION	11	-0.018485088	0.94003252
AXONEMAL DYNEIN COMPLEX ASSEMBLY	14	0.364860722	0.093795561
REGULATION OF LIPID METABOLIC PROCESS	242	0.052157045	0.329876483
HEMIDESMOSOME ASSEMBLY	12	-0.17383031	0.434955984
MAST CELL ACTIVATION	18	-0.250560197	0.16003667
RESPONSE TO EXTERNAL STIMULUS	1483	0.00563342	0.801351944
PROTEIN IMPORT INTO NUCLEUS TRANSLOCATION	23	-0.02258517	0.89422275
REGULATION OF ADENYLATE CYCLASE ACTIVITY	49	-0.048349771	0.675992664
POSITIVE REGULATION OF LEUKOCYTE DIFFERENTIATION	116	-0.021758232	0.774285133
MITOCHONDRIAL MEMBRANE ORGANIZATION	90	-0.091674697	0.27762435
POSITIVE REGULATION OF NEUTROPHIL MIGRATION	25	0.333917624	0.042600614
INTRA GOLGI VESICLE MEDIATED TRANSPORT	40	-0.150886237	0.226184904
REGULATION OF SPINDLE CHECKPOINT	14	-0.087387218	0.682830048
POSITIVE REGULATION OF STRIATED MUSCLE CONTRACTION	12	0.088706229	0.711079758
ANATOMICAL STRUCTURE REGRESSION	9	-0.075222533	0.77857464
CEREBRAL CORTEX NEURON DIFFERENTIATION	15	-0.065141765	0.754258165
OVARIAN FOLLICLE DEVELOPMENT	52	-0.033042672	0.76949715
REGULATION OF VENTRICULAR CARDIAC MUSCLE CELL ACTION POTENTIAL	11	-0.134001122	0.573152121
REGULATION OF MYOBLAST DIFFERENTIATION	40	0.038648724	0.767122363
GLUCAN METABOLIC PROCESS	53	0.062508997	0.583345996
CARDIAC ATRIUM DEVELOPMENT	25	0.127033294	0.446294763
NEGATIVE REGULATION OF CELLULAR RESPONSE TO GROWTH FACTOR STIMULUS	107	0.060663945	0.449857134
FATTY ACID HOMEOSTASIS	11	0.212708602	0.399930165
NUCLEOTIDE SUGAR METABOLIC PROCESS	32	0.122377382	0.406542197
ENERGY DERIVATION BY OXIDATION OF ORGANIC COMPOUNDS	192	-0.112780933	0.050818245
REGULATION OF PLATELET AGGREGATION	14	-0.180721664	0.386421211
POSITIVE REGULATION OF PROTEIN IMPORT INTO NUCLEUS TRANSLOCATION	12	-0.27827758	0.19977685
POSITIVE REGULATION OF CARDIAC MUSCLE CONTRACTION	10	0.146642511	0.576786259
REGULATION OF SUBSTRATE ADHESION DEPENDENT CELL SPREADING	41	-0.03439582	0.786379486
NEPHRIC DUCT DEVELOPMENT	14	0.061264918	0.781730072
POST GOLGI VESICLE MEDIATED TRANSPORT	79	-0.049783324	0.585310298
ERROR FREE TRANSLESION SYNTHESIS	19	0.303324067	0.128096036
EYE PHOTORECEPTOR CELL DIFFERENTIATION	34	0.004798519	0.972773032
RESPONSE TO TESTOSTERONE	30	-0.383577122	0.003920892
CENTRIOLE ASSEMBLY	17	-0.254971344	0.159982062
REGULATION OF HEMOPOIESIS	279	-0.041372556	0.398211753
ATRIAL SEPTUM DEVELOPMENT	16	0.152729447	0.463364971
DETECTION OF TEMPERATURE STIMULUS	11	0.575175837	0.007223548
ION TRANSMEMBRANE TRANSPORT	631	0.017230717	0.605704633
ENAMEL MINERALIZATION	8	0.055018764	0.850670268
POSITIVE REGULATION OF CALCIUM MEDIATED SIGNALING	30	-0.283990076	0.035704908
NEURAL CREST CELL MIGRATION	45	0.060696841	0.623079283
SPINAL CORD MOTOR NEURON CELL FATE SPECIFICATION	9	0.212839879	0.446493302

REGULATION OF GLYCOPROTEIN METABOLIC PROCESS	40	-0.106456405	0.398075427
INTRACELLULAR SIGNAL TRANSDUCTION	1383	-0.016878481	0.463232271
REGULATION OF BEHAVIOR	41	0.030758782	0.811125558
PYRIMIDINE NUCLEOTIDE METABOLIC PROCESS	43	-0.183287821	0.120980933
GLYCOLIPID CATABOLIC PROCESS	9	0.244815309	0.371354504
SYNAPTIC VESICLE ENDOCYTOSIS	17	0.277705329	0.166219613
POSITIVE REGULATION OF PROTEIN TYROSINE KINASE ACTIVITY	32	0.175990596	0.238265562
POSITIVE REGULATION OF HEART RATE	17	0.253782633	0.200601788
PALATE DEVELOPMENT	75	0.130821817	0.175591517
INNATE IMMUNE RESPONSE ACTIVATING CELL SURFACE RECEPTOR SIGNALING PATHWAY	97	-0.168608604	0.034235875
PATTERNING OF BLOOD VESSELS	30	0.036470035	0.808660173
REGULATION OF HOMEOSTATIC PROCESS	381	-0.03266262	0.438364159
FATTY ACID BETA OXIDATION USING ACYL COA OXIDASE	12	-0.069294958	0.764993774
POSITIVE REGULATION OF VASOCONSTRICTION	30	0.1241003	0.414141049
GOLGI TO PLASMA MEMBRANE TRANSPORT	38	-0.110688699	0.391656752
PROTEIN TARGETING TO MEMBRANE	153	-0.109179296	0.091498167
MITOTIC CELL CYCLE	728	-0.12588975	3.19E-05
REGULATION OF FEEDING BEHAVIOR	12	0.043675673	0.854482413
REGULATION OF THYMOCYTE AGGREGATION	22	-0.060135756	0.726524807
PROTEIN COMPLEX BIOGENESIS	999	-0.038251717	0.150178428
REGULATION OF MULTICELLULAR ORGANISM GROWTH	55	0.026967273	0.80822025
REGULATION OF KIDNEY DEVELOPMENT	51	0.005373325	0.962691415
MACROMOLECULAR COMPLEX DISASSEMBLY	167	-0.148366008	0.01521501
POSITIVE REGULATION OF CELLULAR COMPONENT BIOGENESIS	359	0.038441625	0.38253657
POSITIVE REGULATION OF MITOCHONDRIAL MEMBRANE PERMEABILITY	18	-0.031612529	0.868887385
COMPLEMENT ACTIVATION	30	0.321373827	0.029894991
REGULATION OF CATECHOLAMINE SECRETION	34	0.150514946	0.292393597
BONE DEVELOPMENT	135	-0.078098759	0.260090473
NEGATIVE REGULATION OF INTRACELLULAR STEROID HORMONE RECEPTOR SIGNALING PATHWAY	25	0.01371496	0.933437031
DERMATAN SULFATE PROTEOGLYCAN METABOLIC PROCESS	16	0.034385704	0.86741606
REGULATION OF BIOMINERAL TISSUE DEVELOPMENT	61	-0.131987648	0.19194273
REGULATION OF AMINE TRANSPORT	58	0.209835951	0.054421077
SMOOTH MUSCLE TISSUE DEVELOPMENT	16	-0.710721636	7.04E-06
NEGATIVE REGULATION OF HEMATOPOIETIC PROGENITOR CELL DIFFERENTIATION	22	0.134331802	0.448602564
NEGATIVE REGULATION OF NUCLEOSIDE METABOLIC PROCESS	18	0.144530643	0.461730642
NEGATIVE REGULATION OF CATION CHANNEL ACTIVITY	28	-0.205994221	0.154475641
NCRNA PROCESSING	370	-0.130242525	0.001783783
NEGATIVE REGULATION OF TRANSPORT	392	-0.035216266	0.396603342
THYMOCYTE AGGREGATION	42	0.012046798	0.924265089
LEYDIG CELL DIFFERENTIATION	8	-0.004545677	0.98745086
AMINE METABOLIC PROCESS	104	0.087424603	0.28420907
DNA MODIFICATION	64	0.043974164	0.670433507
PROTEIN LOCALIZATION TO MEMBRANE	351	0.012156786	0.783708984
RNA SURVEILLANCE	14	-0.219591068	0.280185222
MITOTIC SPINDLE ASSEMBLY	36	-0.216311258	0.090537602
REGULATION OF GRANULOCYTE DIFFERENTIATION	15	0.110437323	0.607220211
RESPONSE TO ALKALOID	115	-0.063790961	0.397549768
REGULATION OF MESENCHYMAL CELL PROLIFERATION	30	-0.1201969	0.406544755
MORPHOGENESIS OF AN EPITHELIAL FOLD	13	-0.108436117	0.623467465
AUTOPHAGY	357	-0.024959658	0.566829466
REGULATION OF CELL PROJECTION ORGANIZATION	498	0.034258845	0.361252592
REGULATION OF ACTIN FILAMENT LENGTH	137	0.037959852	0.591459289
REGULATION OF TRANSPORTER ACTIVITY	165	-0.070561175	0.262450805
EATING BEHAVIOR	23	0.247979864	0.147855384
GLUTATHIONE DERIVATIVE METABOLIC PROCESS	17	-0.076373832	0.694572072
CELL JUNCTION ORGANIZATION	166	-0.158259432	0.009708427
FOLIC ACID CONTAINING COMPOUND BIOSYNTHETIC PROCESS	13	0.007888025	0.972323399
EMBRYONIC HEART TUBE DEVELOPMENT	63	-0.112022576	0.263750627
SPHINGOID METABOLIC PROCESS	12	-0.224265651	0.304965117
PROTEIN DESTABILIZATION	32	0.010488777	0.94236706
NEGATIVE REGULATION OF RESPONSE TO FOOD	12	0.206560718	0.386353774
L AMINO ACID IMPORT	10	0.067233961	0.797636738
EAR DEVELOPMENT	156	0.029641646	0.654439476

NEGATIVE REGULATION OF CELL MATRIX ADHESION	27	-0.1482123	0.324972875
REGULATION OF MACROPHAGE CHEMOTAXIS	14	0.034663338	0.874901913
LAGGING STRAND ELONGATION	11	0.206836464	0.414211223
POSITIVE REGULATION OF SMOOTH MUSCLE CELL PROLIFERATION	52	-0.034276928	0.761085917
MEMBRANE BUDDING	109	0.014589997	0.853251127
REGULATION OF SYNAPTIC PLASTICITY	118	0.014216978	0.851271057
LONG TERM SYNAPTIC DEPRESSION	15	-0.293322836	0.127252679
GLYCOSYLCERAMIDE METABOLIC PROCESS	11	0.124496089	0.619918567
CELLULAR RESPONSE TO RETINOIC ACID	57	-0.068481608	0.52126545
NCRNA TRANSCRIPTION	88	-0.102451902	0.229420441
REGULATION OF INTERLEUKIN 1 BETA PRODUCTION	34	-0.125336256	0.355174225
NEGATIVE REGULATION OF SMOOTH MUSCLE CELL MIGRATION	16	-0.02225148	0.912957509
NEGATIVE REGULATION OF INTRINSIC APOPTOTIC SIGNALING PATHWAY	84	-0.027287745	0.759014105
LIPOXYGENASE PATHWAY	9	-0.071295374	0.789883657
NEGATIVE REGULATION OF EPITHELIAL CELL APOPTOTIC PROCESS	31	-0.008173492	0.955601214
VESICLE DOCKING INVOLVED IN EXOCYTOSIS	34	-0.074811319	0.587225491
CD4 POSITIVE ALPHA BETA T CELL ACTIVATION	30	0.046673626	0.757092052
ARTERY MORPHOGENESIS	46	0.054405647	0.655821424
NEGATIVE REGULATION OF DEFENSE RESPONSE TO VIRUS	17	-0.166233811	0.378402947
POSITIVE REGULATION OF MOLECULAR FUNCTION	1550	-0.020221318	0.354903745
POSITIVE REGULATION OF REGULATED SECRETORY PATHWAY	40	-0.149927624	0.225667647
SPROUTING ANGIOGENESIS	43	0.011536018	0.926604747
LYMPHOCYTE ACTIVATION	286	-0.069371472	0.148875193
METALLO SULFUR CLUSTER ASSEMBLY	16	-0.052601606	0.794436406
NEGATIVE REGULATION OF RETINOIC ACID RECEPTOR SIGNALING PATHWAY	15	-0.027440342	0.89602464
SPLICEOSOMAL TRI SNRNP COMPLEX ASSEMBLY	10	-0.326860955	0.161163777
REGULATION OF T CELL TOLERANCE INDUCTION	11	0.052661386	0.832550798
POSITIVE REGULATION OF RESPONSE TO OXIDATIVE STRESS	13	0.013182572	0.953813275
PROTEIN TRIMERIZATION	34	-0.052740366	0.703667885
REGULATION OF PEPTIDYL SERINE PHOSPHORYLATION	98	0.020338212	0.806995179
PROTEOGLYCAN METABOLIC PROCESS	75	0.031243962	0.742991918
NITROGEN COMPOUND TRANSPORT	427	-0.147975003	0.000134334
DOPAMINE METABOLIC PROCESS	20	0.124857243	0.501589998
DEFENSE RESPONSE TO OTHER ORGANISM	362	-0.044763443	0.298844563
POSITIVE REGULATION OF GLYCOGEN METABOLIC PROCESS	14	-0.185253301	0.370084279
LEUKOCYTE PROLIFERATION	69	-0.146713206	0.121084016
POSITIVE REGULATION OF MICROTUBULE POLYMERIZATION OR DEPOLYMERIZATION	20	0.071367321	0.700496689
L ASCORBIC ACID METABOLIC PROCESS	11	-0.236358175	0.297063518
CEREBELLAR GRANULAR LAYER DEVELOPMENT	10	-0.258999352	0.277847933
REGULATION OF GENE EXPRESSION BY GENETIC IMPRINTING	11	0.254304619	0.305584002
ADHERENS JUNCTION ASSEMBLY	32	-0.015685228	0.91341807
SECRETORY GRANULE ORGANIZATION	23	-0.121860633	0.45990593
NEGATIVE REGULATION OF POTASSIUM ION TRANSPORT	27	-0.082721477	0.592157126
POSITIVE REGULATION OF INTERLEUKIN 12 PRODUCTION	26	-0.051681866	0.744353715
REGULATION OF T CELL APOPTOTIC PROCESS	29	0.018818448	0.901878058
VESICLE FUSION TO PLASMA MEMBRANE	11	-0.036712988	0.880714552
VENTRICULAR CARDIAC MUSCLE TISSUE DEVELOPMENT	41	0.040214711	0.755192195
CELLULAR RESPONSE TO INTERFERON GAMMA	96	0.094300988	0.267913094
NEGATIVE REGULATION OF HISTONE METHYLATION	16	0.044871127	0.827873784
REGULATION OF GLIAL CELL PROLIFERATION	21	-0.294391879	0.067187982
IN UTERO EMBRYONIC DEVELOPMENT	288	0.038749324	0.42942735
NEGATIVE REGULATION OF BLOOD CIRCULATION	29	-0.099536063	0.501089276
ER NUCLEUS SIGNALING PATHWAY	33	-0.303019301	0.018983039
ETHANOL METABOLIC PROCESS	11	0.176201936	0.483382952
DICARBOXYLIC ACID BIOSYNTHETIC PROCESS	13	0.04227758	0.853391245
PROTEIN HETEROTRIMERIZATION	13	-0.21952815	0.303116184
VESICLE CYTOSKELETAL TRAFFICKING	38	0.06132602	0.648332015
REGULATION OF TRANSCRIPTION FACTOR IMPORT INTO NUCLEUS	87	0.153380515	0.087339041
EPITHELIAL CILIUM MOVEMENT	15	-0.023942274	0.909291034
NEGATIVE REGULATION OF MEIOTIC CELL CYCLE	15	-0.103131808	0.615438218
MESENCHYMAL CELL DIFFERENTIATION	124	0.137195364	0.067827832
NEGATIVE REGULATION OF DNA BIOSYNTHETIC PROCESS	31	-0.274778844	0.04084439
SHORT CHAIN FATTY ACID METABOLIC PROCESS	10	0.177126658	0.500226779
ATRIAL SEPTUM MORPHOGENESIS	12	0.415819776	0.062797936
REGULATION OF SMALL GTPASE MEDIATED SIGNAL TRANSDUCTION	240	-0.02882243	0.585789183

CELLULAR RESPONSE TO GROWTH HORMONE STIMULUS	17	-0.303286125	0.089400344
REGULATION OF INTERLEUKIN 2 BIOSYNTHETIC PROCESS	16	0.096617814	0.643666128
RNA MODIFICATION	108	-0.076583616	0.323458949
CELLULAR COMPONENT ASSEMBLY INVOLVED IN MORPHOGENESIS	207	-0.008629151	0.879971288
ORGANELLE FISSION	447	-0.056246167	0.147137996
EMBRYONIC FORELIMB MORPHOGENESIS	29	0.052799759	0.730929448
REGULATION OF FIBROBLAST APOPTOTIC PROCESS	14	0.23430043	0.295352637
NOSE DEVELOPMENT	11	0.323859205	0.19289734
CELL DIFFERENTIATION INVOLVED IN KIDNEY DEVELOPMENT	30	-0.088099634	0.54628923
REGULATION OF TRANSCRIPTION FROM RNA POLYMERASE III PROMOTER	22	0.215740486	0.224934896
MUSCLE STRUCTURE DEVELOPMENT	375	-0.007408715	0.86228929
NEGATIVE REGULATION OF CELL CYCLE PROCESS	201	-0.111774332	0.047912801
RESPONSE TO INCREASED OXYGEN LEVELS	20	0.033839708	0.854305747
REGULATION OF CHROMATIN SILENCING	19	0.020393151	0.913885399
MACROMOLECULAR COMPLEX REMODELING	17	0.472247631	0.008264953
RIBOSOMAL LARGE SUBUNIT ASSEMBLY	23	-0.118600151	0.4722929
MAINTENANCE OF LOCATION IN CELL	89	-0.096349307	0.25697121
CEREBELLAR CORTEX DEVELOPMENT	39	-0.172718624	0.165036356
REGULATION OF MICROTUBULE POLYMERIZATION	29	0.290371359	0.060485816
REGULATION OF MESODERM DEVELOPMENT	12	0.127746663	0.594227409
T HELPER 1 TYPE IMMUNE RESPONSE	17	0.126636401	0.529696563
POSITIVE REGULATION OF TYPE 2 IMMUNE RESPONSE	12	0.137107012	0.567713852
PLASMA LIPOPROTEIN PARTICLE CLEARANCE	19	0.574031379	0.000967827
CELLULAR RESPONSE TO OXYGEN LEVELS	134	0.111890742	0.121529372
DNA CATABOLIC PROCESS	23	-0.184698871	0.252932331
REGULATION OF INTRACELLULAR ESTROGEN RECEPTOR SIGNALING PATHWAY	24	0.038889579	0.817370522
CELLULAR RESPONSE TO HORMONE STIMULUS	486	0.078092212	0.041175509
RESPONSE TO PROSTAGLANDIN	30	0.341199752	0.020653026
CELL FATE COMMITMENT INVOLVED IN FORMATION OF PRIMARY GERM LAYER	25	-0.269357789	0.0705489
DNA REPAIR	441	-0.011294764	0.774602525
T CELL DIFFERENTIATION INVOLVED IN IMMUNE RESPONSE	27	0.033968873	0.830489179
INSULIN LIKE GROWTH FACTOR RECEPTOR SIGNALING PATHWAY	14	0.128054918	0.565958454
PROTEIN HETEROTETRAMERIZATION	35	-0.159304405	0.227668026
NEGATIVE REGULATION OF HEART CONTRACTION	17	0.006811755	0.972666473
SUBSTRATE INDEPENDENT TELENCEPHALIC TANGENTIAL MIGRATION	8	0.050277467	0.863384413
SULFUR COMPOUND CATABOLIC PROCESS	34	-0.014794839	0.915827905
CHLORIDE TRANSPORT	76	0.118159763	0.216915583
MICROTUBULE DEPOLYMERIZATION	10	-0.065292004	0.797578128
PROTEIN POLYMERIZATION	58	-0.046340945	0.663337752
CELLULAR RESPONSE TO CORTICOSTEROID STIMULUS	50	0.235588777	0.047359858
SIGNAL TRANSDUCTION IN RESPONSE TO DNA DAMAGE	92	-0.07671838	0.361073702
COBALAMIN METABOLIC PROCESS	17	0.067224048	0.737723812
NEGATIVE REGULATION OF SYSTEMIC ARTERIAL BLOOD PRESSURE	10	-0.045333105	0.859422288
MUSCLE CONTRACTION	183	0.00049659	0.993488263
TELOMERE LOCALIZATION	9	-0.016469663	0.951672251
TRANSITION METAL ION HOMEOSTASIS	91	0.122813104	0.161402246
REGULATION OF ADHERENS JUNCTION ORGANIZATION	48	-0.100124022	0.384666943
ENZYME LINKED RECEPTOR PROTEIN SIGNALING PATHWAY	605	0.0069476	0.838046086
PYRIMIDINE NUCLEOTIDE CATABOLIC PROCESS	12	0.019182087	0.935536961
PROTEIN KINASE C ACTIVATING G PROTEIN COUPLED RECEPTOR SIGNALING PATHWAY	26	0.21887316	0.179824018
REGULATION OF DEFENSE RESPONSE TO VIRUS BY VIRUS	26	-0.108192435	0.488888991
REGULATION OF RECEPTOR INTERNALIZATION	32	-0.072422835	0.610192574
REGULATION OF CALCIUM ION TRANSMEMBRANE TRANSPORT	93	-0.26764309	0.000610762
PROTEIN LOCALIZATION TO CHROMOSOME CENTROMERIC REGION	13	-0.426374158	0.027580746
RESPONSE TO BACTERIUM	391	0.041614291	0.324615213
REGULATION OF ANTIGEN PROCESSING AND PRESENTATION	16	-0.059349035	0.768324451
VASOCONSTRICTION	22	0.251585569	0.151128635
REGULATION OF MITOCHONDRIAL TRANSLATION	11	-0.256701753	0.254187088
POSITIVE REGULATION OF LEUKOCYTE MIGRATION	97	-0.066812314	0.415007299
CALCIUM ION IMPORT	58	-0.046610536	0.661356971
CELL MATURATION	97	0.05568016	0.508626691
BEHAVIORAL RESPONSE TO PAIN	8	0.146241043	0.620448841
REGULATION OF SECRETION	562	-0.017690875	0.613630217

CELLULAR RESPONSE TO TOXIC SUBSTANCE	22	-0.134137331	0.423739937
MODULATION OF TRANSCRIPTION IN OTHER ORGANISM INVOLVED IN SYMBIOTIC INTERACTION	22	0.021134755	0.904028086
APOPTOTIC SIGNALING PATHWAY	263	-0.039504283	0.433515032
NEURAL NUCLEUS DEVELOPMENT	58	-0.20997683	0.038118867
LYMPHOCYTE MEDIATED IMMUNITY	92	0.111344611	0.200954851
REGULATION OF HEART MORPHOGENESIS	25	0.086560761	0.602560576
S ADENOSYLMETHIONINE METABOLIC PROCESS	16	-0.048597043	0.810021283
FC EPSILON RECEPTOR SIGNALING PATHWAY	116	-0.129669125	0.078768759
HIPPOCAMPUS DEVELOPMENT	64	0.116907415	0.263950404
REGULATION OF PROTEIN LOCALIZATION TO CELL SURFACE	24	-0.188331284	0.236399685
RIBONUCLEOSIDE TRIPHOSPHATE BIOSYNTHETIC PROCESS	44	-0.322945936	0.003299771
PRIMARY AMINO COMPOUND METABOLIC PROCESS	13	-0.153630908	0.478389669
POSITIVE REGULATION OF TELOMERE CAPPING	14	0.382354388	0.089603275
NEGATIVE REGULATION OF AMINE TRANSPORT	21	0.210496335	0.24290163
REGULATION OF CHROMOSOME SEGREGATION	82	-0.135013357	0.121854832
POSITIVE REGULATION OF INSULIN RECEPTOR SIGNALING PATHWAY	10	-0.076917863	0.761444581
PONS DEVELOPMENT	9	-0.02528242	0.92570784
PROTEIN K11 LINKED UBIQUITINATION	26	-0.180380049	0.235766213
DETECTION OF CHEMICAL STIMULUS INVOLVED IN SENSORY PERCEPTION OF TASTE	19	0.184462155	0.331360015
REGULATION OF MESENCHYMAL CELL APOPTOTIC PROCESS	8	-0.044818995	0.875491641
RESPONSE TO INACTIVITY	10	0.212029832	0.420304911
SPHINGOLIPID METABOLIC PROCESS	124	-0.032213433	0.659953925
RESPONSE TO PAIN	19	0.183896971	0.334750416
PROTEIN ACETYLATION	119	0.065418864	0.390716163
POSITIVE REGULATION OF TRANSCRIPTION ELONGATION FROM RNA POLYMERASE II PROMOTER	13	-0.225367663	0.288895366
REGULATION OF NUCLEOCYTOPLASMIC TRANSPORT	201	-0.029425579	0.610111935
NEGATIVE REGULATION OF CALCIUM ION IMPORT	22	0.113233815	0.52308464
CELLULAR HORMONE METABOLIC PROCESS	79	0.095813477	0.305884131
PTERIDINE CONTAINING COMPOUND METABOLIC PROCESS	31	0.010191252	0.944873599
BONE REMODELING	31	-0.080561089	0.576223641
EMBRYONIC CAMERA TYPE EYE MORPHOGENESIS	19	-0.051262091	0.782313051
FORMATION OF TRANSLATION PREINITIATION COMPLEX	21	-0.400115578	0.01230404
CHEMOKINE MEDIATED SIGNALING PATHWAY	51	0.084307675	0.468213121
POSITIVE REGULATION OF INTERFERON BETA PRODUCTION	28	-0.208305949	0.153530697
NEGATIVE REGULATION OF LEUKOCYTE MIGRATION	27	0.220997579	0.169528674
REGULATION OF PROTEIN SECRETION	316	-0.077220489	0.090824761
REGULATION OF PEPTIDYL TYROSINE PHOSPHORYLATION	189	0.056435371	0.351108206
REGULATION OF NATURAL KILLER CELL ACTIVATION	25	-0.014987179	0.926824457
RESPONSE TO INSULIN	181	-0.037286784	0.538734265
POSITIVE REGULATION OF FILOPODIUM ASSEMBLY	23	-0.089865786	0.590169878
REGULATION OF MUSCLE CELL APOPTOTIC PROCESS	39	-0.009876919	0.939841198
POSITIVE REGULATION OF NIK NF KAPPAB SIGNALING	27	0.002095313	0.989395167
REGULATION OF CATABOLIC PROCESS	677	-0.034785599	0.276145934
REGULATION OF CARBOHYDRATE METABOLIC PROCESS	146	-0.026812264	0.691731423
NEGATIVE REGULATION OF SYNAPTIC TRANSMISSION	53	-0.049663212	0.655310917
NEGATIVE REGULATION OF CYTOKINE BIOSYNTHETIC PROCESS	19	0.038189296	0.840012704
ROSTROCAUDAL NEURAL TUBE PATTERNING	11	-0.183784536	0.430251267
B CELL ACTIVATION	112	-0.082759869	0.276570195
CELL DEATH IN RESPONSE TO OXIDATIVE STRESS	17	-0.265851011	0.138656947
REGULATION OF NEURON DIFFERENTIATION	483	0.017171286	0.651021695
NEGATIVE REGULATION OF INTERFERON GAMMA PRODUCTION	24	0.280958531	0.092497421
REGULATION OF FIBRINOLYSIS	12	0.224433439	0.347160979
NEGATIVE REGULATION OF NF KAPPAB TRANSCRIPTION FACTOR ACTIVITY	61	-0.055144676	0.594320326
PROTEIN POLYUBIQUITINATION	229	-0.07559515	0.157703148
RIBONUCLEOPROTEIN COMPLEX LOCALIZATION	113	-0.244974635	0.000726561
REGULATION OF MRNA 3 END PROCESSING	28	-0.097661489	0.517704835
REGULATION OF PEPTIDE TRANSPORT	219	-0.011571598	0.834967924
POSITIVE REGULATION OF ION TRANSPORT	198	-0.054338332	0.346959087
ATP BIOSYNTHETIC PROCESS	31	-0.229213023	0.091209199
CANONICAL WNT SIGNALING PATHWAY	86	-0.021202798	0.809689942
ACUTE INFLAMMATORY RESPONSE	57	0.021767306	0.841776772
NUCLEOBASE METABOLIC PROCESS	37	-0.113922487	0.382711473
HUMORAL IMMUNE RESPONSE	108	0.109708036	0.17165668

NEGATIVE REGULATION OF INTERLEUKIN 10 PRODUCTION	11	0.213887905	0.393581446
PHAGOSOME MATURATION	32	0.242988665	0.103021203
REGULATION OF CELLULAR RESPONSE TO HYPOXIA	11	0.017381694	0.944035908
REGULATION OF FEMALE GONAD DEVELOPMENT	9	-0.082676889	0.757051777
REGULATION OF THYMOCYTE APOPTOTIC PROCESS	12	-0.164100521	0.465616999
CHROMATIN MODIFICATION	497	-0.046202703	0.211178387
MYELOID LEUKOCYTE ACTIVATION	85	-0.075711297	0.385983542
REACTIVE NITROGEN SPECIES METABOLIC PROCESS	17	0.138367939	0.492134306
ANTERIOR POSTERIOR PATTERN SPECIFICATION	173	-0.089371413	0.143933884
POSITIVE REGULATION OF CELL DIVISION	107	-0.035824361	0.649010409
EYELID DEVELOPMENT IN CAMERA TYPE EYE	12	-0.274454198	0.215544623
PIGMENT BIOSYNTHETIC PROCESS	41	-0.143172895	0.243542764
ERBB SIGNALING PATHWAY	75	0.18201487	0.062824419
EMBRYONIC ORGAN MORPHOGENESIS	239	-0.015729013	0.767301597
REPRODUCTIVE SYSTEM DEVELOPMENT	344	0.114890212	0.011490018
CYTOSOLIC CALCIUM ION TRANSPORT	48	-0.122941439	0.281623291
REGULATION OF SPINDLE ORGANIZATION	19	0.238757631	0.208258785
POSITIVE REGULATION OF CATABOLIC PROCESS	365	-0.028582833	0.50701299
PHOSPHOLIPID SCRAMBLING	10	0.52930251	0.019113139
GLIOGENESIS	156	0.108461559	0.105554998
PEPTIDYL THREONINE MODIFICATION	46	-0.318814528	0.003177106
MITOCHONDRIAL FISSION	13	-0.400289801	0.037526058
MATURATION OF 5 8S RRNA FROM TRICISTRONIC RRNA TRANSCRIPT SSU RRNA 5 8S RRNA LSU RRNA	19	-0.11204562	0.537675482
NEGATIVE REGULATION OF SPROUTING ANGIOGENESIS	11	-0.285529686	0.19620733
POLYOL TRANSPORT	11	-0.065172195	0.788137217
REGULATION OF CARDIAC MUSCLE CONTRACTION BY CALCIUM ION SIGNALING	22	-0.404570523	0.005995067
ANTIGEN PROCESSING AND PRESENTATION	188	-0.068642418	0.245018654
CELLULAR AMINO ACID CATABOLIC PROCESS	96	0.016193912	0.847189514
TELENCEPHALON GLIAL CELL MIGRATION	20	-0.10764077	0.545177254
MAINTENANCE OF LOCATION	129	-0.052520124	0.462361792
NEPHRON EPITHELIUM DEVELOPMENT	78	-0.068913183	0.4503061
NEGATIVE REGULATION OF RECEPTOR ACTIVITY	27	-0.080704796	0.600635228
KERATINOCYTE DIFFERENTIATION	66	0.213371008	0.03990122
REGULATION OF NITRIC OXIDE SYNTHASE BIOSYNTHETIC PROCESS	17	0.02859822	0.886081681
POSITIVE REGULATION OF CALCIUM ION TRANSMEMBRANE TRANSPORT	49	-0.341856949	0.001131007
NEGATIVE REGULATION OF EPITHELIAL CELL MIGRATION	48	0.020703037	0.861608681
POSITIVE REGULATION OF SYNAPSE MATURATION	11	0.147063805	0.556976354
PERIPHERAL NERVOUS SYSTEM NEURON DIFFERENTIATION	12	-0.011416419	0.961358127
CELL REDOX HOMEOSTASIS	56	-0.267902015	0.00816158
POSITIVE REGULATION OF SMOOTH MUSCLE CONTRACTION	26	-0.022605985	0.887472564
NEGATIVE REGULATION OF GLIAL CELL PROLIFERATION	11	-0.175045294	0.450956364
POSITIVE REGULATION OF PROTEIN SECRETION	166	-0.001367194	0.982909746
POSITIVE REGULATION OF CYTOSKELETON ORGANIZATION	157	0.12411076	0.063356531
REGULATION OF STEROID HORMONE SECRETION	11	0.194537747	0.435983097
RESPONSE TO KETONE	155	-0.008365625	0.899024701
NEGATIVE REGULATION OF PROTEIN KINASE B SIGNALING	34	-0.068675284	0.61879052
LYMPHANGIOGENESIS	10	0.156662584	0.55336283
REGULATION OF POSTSYNAPTIC MEMBRANE POTENTIAL	44	0.032996834	0.790640854
POSITIVE REGULATION OF TOLL LIKE RECEPTOR SIGNALING PATHWAY	16	0.246624639	0.230247155
SENSORY PERCEPTION OF TASTE	33	0.251820737	0.078611442
FEAR RESPONSE	25	0.126052823	0.449306186
REGULATION OF CLATHRIN MEDIATED ENDOCYTOSIS	14	0.607177488	0.002933952
REGULATION OF ENDOTHELIAL CELL MIGRATION	104	0.003348335	0.9668425
LABYRINTHINE LAYER BLOOD VESSEL DEVELOPMENT	16	0.422132191	0.029355064
NEURON NEURON SYNAPTIC TRANSMISSION	43	-0.163611578	0.169221279
INNER MITOCHONDRIAL MEMBRANE ORGANIZATION	17	0.105712147	0.599526101
CILIUM MORPHOGENESIS	178	0.006727063	0.913271141
ENERGY COUPLED PROTON TRANSPORT DOWN ELECTROCHEMICAL GRADIENT	21	-0.205770932	0.217102762
POSITIVE REGULATION OF PHOSPHOPROTEIN PHOSPHATASE ACTIVITY	15	0.029066032	0.891235845
CELLULAR MODIFIED AMINO ACID BIOSYNTHETIC PROCESS	43	0.051136569	0.685044918
PROTEIN LOCALIZATION TO VACUOLE	45	0.101745256	0.414411605
METANEPHRIC RENAL VESICLE MORPHOGENESIS	9	-0.101577531	0.702292552
NEGATIVE REGULATION OF RHO PROTEIN SIGNAL TRANSDUCTION	12	-0.102521099	0.655125137
CELLULAR RESPONSE TO HYDROGEN PEROXIDE	56	-0.126117862	0.234199477

REGULATION OF ORGAN GROWTH	68	0.006258452	0.949875492
REGULATION OF SYNAPSE ASSEMBLY	61	0.044165805	0.676437721
NEGATIVE REGULATION OF NEUROTRANSMITTER SECRETION	10	-0.203485879	0.405209448
POSITIVE REGULATION OF CELL GROWTH	136	-0.111468544	0.103892517
NEGATIVE REGULATION OF OXIDOREDUCTASE ACTIVITY	18	0.383058061	0.037589945
EMBRYONIC DIGESTIVE TRACT DEVELOPMENT	30	0.156156434	0.30449614
PROTEIN O LINKED MANNOSYLATION	16	-0.191936638	0.31664201
GUANOSINE CONTAINING COMPOUND BIOSYNTHETIC PROCESS	13	-0.177032694	0.410379446
BONE MINERALIZATION	28	0.016953432	0.913057387
REGULATION OF TRANSMEMBRANE RECEPTOR PROTEIN SERINE THREONINE KINASE SIGNALING PATHWAY	179	0.153927224	0.014455219
CELLULAR RESPONSE TO ORGANIC SUBSTANCE	1598	-0.024520253	0.25520396
OVULATION CYCLE PROCESS	74	0.001577541	0.986801213
POSITIVE REGULATION OF CELL ADHESION	329	-0.050452002	0.263092039
PROTEIN LOCALIZATION TO CHROMATIN	13	-0.203448125	0.334449102
PROTEIN O LINKED FUCOSYLATION	9	0.111964146	0.686395853
CEREBRAL CORTEX DEVELOPMENT	96	-0.023116173	0.78146285
ENDOCRINE PANCREAS DEVELOPMENT	32	0.064680601	0.658950602
REGULATION OF INTERLEUKIN 8 PRODUCTION	48	-0.075008788	0.517893203
RNA PHOSPHODIESTER BOND HYDROLYSIS	109	0.027748588	0.725649858
ORGANIC ACID METABOLIC PROCESS	786	-0.042641101	0.151182505
REGULATION OF EXCRETION	20	0.19731582	0.288844036
AROMATIC AMINO ACID FAMILY METABOLIC PROCESS	25	0.175205803	0.29076539
CYCLIC NUCLEOTIDE MEDIATED SIGNALING	39	-0.088905774	0.487399961
POSITIVE REGULATION OF PHOSPHOLIPID METABOLIC PROCESS	38	-0.215423229	0.083087484
REGULATION OF ENDOCRINE PROCESS	32	0.151900864	0.302058869
RESPONSE TO INSECTICIDE	9	0.337547785	0.20390858
REGULATION OF NUCLEAR TRANSCRIBED MRNA POLY A TAIL SHORTENING	11	0.143412305	0.569659319
QUATERNARY AMMONIUM GROUP TRANSPORT	17	0.144949063	0.475303563
EXIT FROM MITOSIS	12	0.10227175	0.669701951
REGULATION OF SYMBIOSIS ENCOMPASSING MUTUALISM THROUGH PARASITISM	188	0.119834792	0.050283209
RESPONSE TO GROWTH HORMONE	27	-0.031850287	0.838642504
PROTEIN DEMANNOSYLATION	12	-0.423262616	0.044642645
G1 DNA DAMAGE CHECKPOINT	70	-0.072223374	0.453606304
POSITIVE REGULATION OF PRI MIRNA TRANSCRIPTION FROM RNA POLYMERASE II PROMOTER	11	-0.522785274	0.010356843
RESPONSE TO OXYGEN LEVELS	282	-0.037474388	0.442239482
POSITIVE REGULATION OF IMMUNE SYSTEM PROCESS	706	-0.040551533	0.194701619
PHOTOTRANSDUCTION VISIBLE LIGHT	12	-0.106304311	0.64381288
CELL COMMUNICATION INVOLVED IN CARDIAC CONDUCTION	35	-0.027660531	0.840632586
PROTEIN LOCALIZATION TO CHROMOSOME TELOMERIC REGION	13	-0.277873015	0.169877533
POSITIVE REGULATION OF CALCIUM ION TRANSMEMBRANE TRANSPORTER ACTIVITY	26	-0.550499336	1.96E-05
REGULATION OF RECEPTOR MEDIATED ENDOCYTOSIS	66	0.052516091	0.606489291
POSITIVE REGULATION OF LYMPHOCYTE MEDIATED IMMUNITY	58	-0.109842853	0.2929088
POSITIVE REGULATION OF BIOMINERAL TISSUE DEVELOPMENT	31	-0.1592306	0.256172486
RESPONSE TO TRANSFORMING GROWTH FACTOR BETA	131	-0.043903459	0.536686084
N ACETYLGLUCOSAMINE METABOLIC PROCESS	14	0.107694717	0.62859535
TRANSCRIPTION ELONGATION FROM RNA POLYMERASE II PROMOTER	78	-0.060136747	0.511116953
OXIDATIVE PHOSPHORYLATION	80	-0.247098188	0.003309611
APOPTOTIC NUCLEAR CHANGES	23	0.026590042	0.876884981
REGULATION OF RAC PROTEIN SIGNAL TRANSDUCTION	12	0.079389717	0.740896309
CELLULAR AMIDE METABOLIC PROCESS	645	-0.147433554	3.55E-06
PARENTAL BEHAVIOR	10	-0.429021515	0.047251134
REGULATION OF DNA BINDING	89	0.103813875	0.241023015
IMMUNOGLOBULIN PRODUCTION	38	0.016942127	0.898897597
REGULATION OF TRANSFORMING GROWTH FACTOR BETA PRODUCTION	23	-0.025183075	0.882036297
TISSUE MIGRATION	75	-0.052802671	0.572177873
ONE CARBON COMPOUND TRANSPORT	6	0.358928673	0.262619733
LEFT RIGHT AXIS SPECIFICATION	12	0.357479089	0.126701799
RESPONSE TO ISCHEMIA	27	0.039299836	0.804533377
ENSHEATHMENT OF NEURONS	80	0.071196928	0.443368118
POSITIVE REGULATION OF MAST CELL ACTIVATION INVOLVED IN IMMUNE RESPONSE	10	0.071653068	0.784265318
POSITIVE REGULATION OF PHOSPHORUS METABOLIC PROCESS	871	0.013014553	0.649344553

CHROMATIN DISASSEMBLY	14	-0.031403412	0.88505035
PLATELET MORPHOGENESIS	17	-0.298064341	0.099285151
DENDRITE MORPHOGENESIS	38	-0.069686888	0.593853478
RESPONSE TO AMPHETAMINE	24	-0.034287624	0.836119865
RESPONSE TO CAMP	87	0.009027685	0.918404008
MYOBLAST DIFFERENTIATION	32	0.071126243	0.628047995
POSITIVE REGULATION OF ACTIN NUCLEATION	15	0.439703392	0.033409587
REGULATION OF MEGAKARYOCYTE DIFFERENTIATION	25	0.302442045	0.06105583
NEGATIVE REGULATION OF HEMOPOIESIS	114	-0.068933356	0.36192616
REGULATION OF MUSCLE CELL DIFFERENTIATION	134	0.021765781	0.7601569
INNATE IMMUNE RESPONSE IN MUCOSA	16	0.003876975	0.984895477
REGULATION OF CHEMOKINE SECRETION	7	0.372431288	0.207852127
RESPONSE TO STEROL	21	0.184702171	0.309503858
REGULATION OF ENDOPLASMIC RETICULUM STRESS INDUCED INTRINSIC APOPTOTIC SIGNALING PATHWAY	30	-0.127501824	0.376660101
RIBOSOMAL SMALL SUBUNIT BIOGENESIS	56	-0.152649212	0.145374646
TRABECULA MORPHOGENESIS	37	0.073533177	0.590219258
PHOTORECEPTOR CELL MAINTENANCE	21	-0.020734428	0.907151648
REGULATION OF CALCIUM ION IMPORT	83	-0.186639318	0.028557633
HEPARAN SULFATE PROTEOGLYCAN METABOLIC PROCESS	24	0.115619198	0.496989019
INTESTINAL EPITHELIAL CELL DEVELOPMENT	11	-0.102129783	0.670994532
CELLULAR RESPONSE TO GLUCAGON STIMULUS	33	0.16637609	0.251733623
REGULATION OF METAL ION TRANSPORT	267	-0.056858553	0.254051241
POSITIVE REGULATION OF T HELPER 1 TYPE IMMUNE RESPONSE	12	0.078182998	0.74395667
MODIFICATION BY SYMBIONT OF HOST MORPHOLOGY OR PHYSIOLOGY	43	0.098463658	0.438297032
SISTER CHROMATID SEGREGATION	164	-0.184028579	0.002560797
REGULATION OF CELL MIGRATION INVOLVED IN SPROUTING ANGIOGENESIS	19	0.058971965	0.755979898
PHASIC SMOOTH MUSCLE CONTRACTION	11	0.376051866	0.111881537
AXON ENSHEATHMENT IN CENTRAL NERVOUS SYSTEM	9	0.14922666	0.59023845
MONOSACCHARIDE BIOSYNTHETIC PROCESS	48	-0.052846454	0.650765336
ORGANELLE MEMBRANE FUSION	80	-0.016930208	0.852980037
REGULATION OF ERK1 AND ERK2 CASCADE	197	0.040696845	0.491233683
REGULATION OF T CELL MEDIATED CYTOTOXICITY	19	-0.077256448	0.674928702
CARBOHYDRATE DERIVATIVE BIOSYNTHETIC PROCESS	509	-0.094188135	0.00908291
NEGATIVE REGULATION OF ACTIN FILAMENT BUNDLE ASSEMBLY	17	-0.242208975	0.182131225
POSITIVE REGULATION OF CELL JUNCTION ASSEMBLY	23	-0.18433986	0.251600153
NONRIBOSOMAL PEPTIDE BIOSYNTHETIC PROCESS	12	0.299557491	0.197516918
HEART MORPHOGENESIS	190	0.024801916	0.679440828
RAS PROTEIN SIGNAL TRANSDUCTION	133	0.018777282	0.792911956
NEGATIVE REGULATION OF DENDRITE DEVELOPMENT	24	-0.233218364	0.131631765
REGULATION OF HORMONE SECRETION	217	-0.012881404	0.817362729
REGULATION OF CALCIUM ION TRANSPORT	168	-0.155554825	0.010524752
SENSORY PERCEPTION OF LIGHT STIMULUS	141	0.079288698	0.2582213
TERPENOID METABOLIC PROCESS	75	0.080451615	0.40160316
CELL PROLIFERATION	589	0.01247614	0.717380175
REGULATION OF LIPID KINASE ACTIVITY	46	-0.251382066	0.024036351
POSITIVE REGULATION OF OSTEOCLAST DIFFERENTIATION	21	0.14356008	0.429813644
LYSOSOME LOCALIZATION	20	-0.221563277	0.196979278
VITAMIN D METABOLIC PROCESS	11	0.188057792	0.454834105
REGULATION OF FIBROBLAST PROLIFERATION	75	0.055283827	0.563586658
CELL AGGREGATION	15	0.209645296	0.32727587
RESPONSE TO HORMONE	768	0.050231586	0.100476989
REGULATION OF SYNCYTIUM FORMATION BY PLASMA MEMBRANE FUSION	23	-0.032506951	0.847825829
REGULATION OF HEXOKINASE ACTIVITY	9	-0.614349941	0.007759014
POSITIVE REGULATION OF EXTRINSIC APOPTOTIC SIGNALING PATHWAY IN ABSENCE OF LIGAND	13	-0.110354131	0.616475814
CATECHOLAMINE BIOSYNTHETIC PROCESS	14	0.0748034	0.735402677
POSITIVE REGULATION OF MUSCLE HYPERTROPHY	21	0.09039537	0.618865892
EMBRYONIC PATTERN SPECIFICATION	51	-0.078733663	0.483812425
RESPONSE TO PROTOZOAN	17	0.110075418	0.586860842
MULTIVESICULAR BODY ORGANIZATION	30	0.26285237	0.090622075
REGULATION OF CELL GROWTH	356	0.061007323	0.169222786
CENTROSOME CYCLE	41	-0.174428456	0.150483905
RESPONSE TO FIBROBLAST GROWTH FACTOR	105	-0.004409433	0.956063374
PROTEIN CARBOXYLATION	10	0.119452257	0.649003608

NEGATIVE REGULATION OF TELOMERE MAINTENANCE VIA TELOMERE LENGTHENING	17	-0.307005475	0.096848008
DEOXYRIBONUCLEOSIDE TRIPHOSPHATE METABOLIC PROCESS	17	0.142767115	0.47936597
NUCLEOTIDE EXCISION REPAIR PREINCISION COMPLEX ASSEMBLY	28	0.502620318	0.000582412
POSITIVE REGULATION OF EXTRACELLULAR MATRIX ORGANIZATION	16	0.313258159	0.135319742
LEARNING	113	-0.046895622	0.539294551
CILIUM ORGANIZATION	159	0.004968918	0.93933196
REGULATION OF PHOSPHOLIPASE ACTIVITY	52	-0.038351504	0.733502326
REGULATION OF RESPONSE TO FOOD	15	0.153313222	0.474483072
CHONDROCYTE DIFFERENTIATION	48	-0.001166412	0.992128552
REGULATION OF CELLULAR LOCALIZATION	1091	-0.063099733	0.01299897
ADRENERGIC RECEPTOR SIGNALING PATHWAY	15	0.004057113	0.984695696
OVULATION	14	0.004107839	0.985029419
NEGATIVE REGULATION OF TOR SIGNALING	30	0.154620481	0.311562996
SUBSTANTIA NIGRA DEVELOPMENT	41	-0.239619591	0.044230446
MATURATION OF SSU RRNA FROM TRICISTRONIC RRNA TRANSCRIPT SSU RRNA 5 8S RRNA LSU RRNA	33	-0.165270144	0.222943597
PRODUCTION OF MOLECULAR MEDIATOR INVOLVED IN INFLAMMATORY RESPONSE	11	-0.302173366	0.180442232
PREPULSE INHIBITION	8	0.1368716	0.640635217
REGULATION OF EARLY ENDOSOME TO LATE ENDOSOME TRANSPORT	16	-0.186999133	0.336720189
MIDDLE EAR MORPHOGENESIS	19	0.12017209	0.528867119
REGULATION OF RECEPTOR BIOSYNTHETIC PROCESS	19	0.086871208	0.648818586
JNK CASCADE	70	-0.002556158	0.979179567
RESPONSE TO SALT STRESS	16	0.39610048	0.041766843
ENDODERMAL CELL DIFFERENTIATION	37	-0.212159119	0.095524571
PROTEIN KINASE B SIGNALING	33	-0.058958193	0.674596019
SOMITOGENESIS	54	0.030237015	0.787527747
REGULATION OF MULTICELLULAR ORGANISMAL DEVELOPMENT	1456	0.018791837	0.406257888
REGULATION OF DENDRITIC SPINE DEVELOPMENT	51	-0.263069549	0.013433097
NEGATIVE REGULATION OF PROTEIN ACTIVATION CASCADE	9	0.105754043	0.703159729
TRICARBOXYLIC ACID METABOLIC PROCESS	31	-0.326208524	0.01403985
REGULATION OF TRIGLYCERIDE CATABOLIC PROCESS	8	0.329215763	0.245934317
NEGATIVE REGULATION OF RESPONSE TO EXTRACELLULAR STIMULUS	35	-0.056111418	0.681052211
HEMATOPOIETIC STEM CELL PROLIFERATION	12	-0.073560541	0.750994508
NEGATIVE REGULATION OF ESTABLISHMENT OF PROTEIN LOCALIZATION	177	-0.085249817	0.15920845
RENAL TUBULE DEVELOPMENT	66	0.002807626	0.977817331
CARDIAC VENTRICLE MORPHOGENESIS	55	0.211162375	0.060628054
ANTIGEN PROCESSING AND PRESENTATION OF PEPTIDE ANTIGEN VIA MHC CLASS I	83	-0.240538141	0.003853461
REGULATION OF THE FORCE OF HEART CONTRACTION	25	0.02479802	0.880213575
POSITIVE REGULATION OF EPITHELIAL CELL APOPTOTIC PROCESS	20	-0.398510785	0.011097538
MACROMOLECULAR COMPLEX ASSEMBLY	1249	-0.059731781	0.012457716
PROTEIN STABILIZATION	124	-0.095267671	0.186070576
POSITIVE REGULATION OF INTERLEUKIN 6 SECRETION	13	0.236778251	0.308366984
ASPARTATE FAMILY AMINO ACID CATABOLIC PROCESS	16	0.252235408	0.221981832
NEGATIVE REGULATION OF PROTEIN SECRETION	82	-0.119475019	0.172825904
NEGATIVE REGULATION OF LIPASE ACTIVITY	15	0.080391243	0.707715427
REGULATION OF CELLULAR KETONE METABOLIC PROCESS	146	-0.05890369	0.379729532
MATURATION OF LSU RRNA	13	-0.146133391	0.500656081
RNA SPLICING VIA ENDONUCLEOLYTIC CLEAVAGE AND LIGATION	16	-0.280176851	0.128819001
REGULATION OF SYNAPSE ORGANIZATION	91	0.08808044	0.312621892
MUSCLE ADAPTATION	22	-0.028668276	0.868651155
INTERFERON GAMMA PRODUCTION	13	-0.192043846	0.369160837
OPTIC NERVE DEVELOPMENT	9	0.232627884	0.400205463
PHOSPHOLIPASE C ACTIVATING G PROTEIN COUPLED RECEPTOR SIGNALING PATHWAY	57	0.101123112	0.359366818
REGULATION OF KERATINOCYTE PROLIFERATION	26	0.021782507	0.892564771
GAS TRANSPORT	12	0.190286966	0.429212464
REGULATION OF FIBROBLAST GROWTH FACTOR RECEPTOR SIGNALING PATHWAY	24	0.106277249	0.531514858
REGULATION OF MAST CELL ACTIVATION	33	-0.182873894	0.175850567
MIDBRAIN DEVELOPMENT	80	-0.153301316	0.081042705
NEURON FATE SPECIFICATION	19	0.207789711	0.279638527
ORGANIC ACID CATABOLIC PROCESS	171	0.065048468	0.306571589
REPRODUCTIVE BEHAVIOR	25	-0.017378056	0.91514919

ESTABLISHMENT OF TISSUE POLARITY	16	0.355210009	0.074224635
REGULATION OF NEURON APOPTOTIC PROCESS	178	-0.034001512	0.578527326
CELL SEPARATION AFTER CYTOKINESIS	16	0.143034731	0.49449019
REGULATION OF GLUCOSE IMPORT IN RESPONSE TO INSULIN STIMULUS	15	0.0325021	0.878573643
AMIDE BIOSYNTHETIC PROCESS	466	-0.175647132	1.86E-06
POSITIVE REGULATION OF EXOCYTOSIS	69	-0.025169956	0.797597228
MORPHOGENESIS OF AN EPITHELIAL SHEET	38	-0.014710075	0.911561914
CHEMOSENSORY BEHAVIOR	10	0.05296659	0.839417641
PROTEIN KINASE C SIGNALING	13	-0.035064715	0.876191575
GLUCOSE IMPORT	10	-0.358492955	0.1116641
SISTER CHROMATID COHESION	104	-0.191859693	0.011726714
REGULATION OF I KAPPAB KINASE NF KAPPAB SIGNALING	215	-0.115972265	0.033496007
REGULATION OF PROTEIN KINASE A SIGNALING	16	0.166137785	0.426282296
HEPATOCYTE APOPTOTIC PROCESS	13	-0.645027792	0.000127192
TETRAHYDROFOLATE METABOLIC PROCESS	18	-0.067091734	0.723409515
ORGAN MORPHOGENESIS	706	0.041574337	0.190769758
POSITIVE REGULATION OF MULTICELLULAR ORGANISMAL PROCESS	1195	0.046805079	0.059814799
REGULATION OF NECROTIC CELL DEATH	25	0.059051046	0.721308523
CELLULAR RESPONSE TO CAMP	45	-0.027602305	0.820064556
PROTEIN LOCALIZATION TO CHROMOSOME	43	-0.258631278	0.02312941
POSTSYNAPTIC MEMBRANE ORGANIZATION	22	-0.090554614	0.594761775
CARNITINE METABOLIC PROCESS	12	0.223730916	0.349729889
CYTOPLASMIC TRANSLATIONAL INITIATION	14	-0.293804847	0.151536095
COMPLEMENT ACTIVATION ALTERNATIVE PATHWAY	7	0.116716477	0.709835069
CARDIAC MUSCLE CELL DIFFERENTIATION	65	0.035910779	0.725854927
NUCLEOSIDE TRIPHOSPHATE METABOLIC PROCESS	206	-0.135350343	0.014465203
CILIUM MOVEMENT	27	0.231692219	0.148289967
BRANCHING INVOLVED IN URETERIC BUD MORPHOGENESIS	39	-0.149903296	0.233588773
VASCULAR SMOOTH MUSCLE CONTRACTION	12	0.200148909	0.403076463
REGULATION OF RESPONSE TO WOUNDING	339	0.093825908	0.039700516
POSITIVE T CELL SELECTION	15	0.142851777	0.512786107
CELLULAR RESPONSE TO ARSENIC CONTAINING SUBSTANCE	14	-0.462310906	0.013564097
POSITIVE REGULATION OF RECEPTOR BIOSYNTHETIC PROCESS	9	-0.127279375	0.630373023
POSITIVE REGULATION OF STEM CELL DIFFERENTIATION	47	0.112935659	0.353266253
REGULATION OF HEART RATE	71	-0.010756629	0.911774164
RESPONSE TO PLATELET DERIVED GROWTH FACTOR	18	-0.225618613	0.204199352
REGULATION OF EXTRINSIC APOPTOTIC SIGNALING PATHWAY	145	-0.012824525	0.850562901
INTRASPECIES INTERACTION BETWEEN ORGANISMS	40	-0.075610691	0.551775927
REGULATION OF EXTRINSIC APOPTOTIC SIGNALING PATHWAY IN ABSENCE OF LIGAND	43	0.080911133	0.522761934
PYRIMIDINE RIBONUCLEOTIDE METABOLIC PROCESS	22	-0.379880591	0.013559187
REGULATION OF B CELL DIFFERENTIATION	20	0.07251167	0.695591343
TRANSCRIPTION FROM MITOCHONDRIAL PROMOTER	10	0.036172281	0.889633936
MAINTENANCE OF PROTEIN LOCALIZATION IN ORGANELLE	26	-0.05409094	0.733042279
MRNA 3 END PROCESSING	70	-0.221218647	0.016131963
POSITIVE REGULATION OF TRANSCRIPTION FROM RNA POLYMERASE II PROMOTER IN RESPONSE TO STRESS	23	-0.068132838	0.684688412
REGULATION OF CERAMIDE BIOSYNTHETIC PROCESS	9	0.362307357	0.18174703
REGULATION OF LIPASE ACTIVITY	67	-0.089985622	0.358295368
POLARIZED EPITHELIAL CELL DIFFERENTIATION	11	-0.108752635	0.65335011
INNATE IMMUNE RESPONSE	446	0.067729944	0.088825829
CARDIAC RIGHT VENTRICLE MORPHOGENESIS	16	0.197747111	0.340645992
G PROTEIN COUPLED RECEPTOR SIGNALING PATHWAY	575	0.106814736	0.002516717
DIGESTIVE TRACT MORPHOGENESIS	41	0.027429698	0.831129894
SPERM EGG RECOGNITION	28	-0.000391011	0.997983621
GALACTOSE METABOLIC PROCESS	13	-0.320318261	0.123481411
GLOBAL GENOME NUCLEOTIDE EXCISION REPAIR	32	0.067889922	0.643470008
REGULATION OF ACYL COA BIOSYNTHETIC PROCESS	13	-0.406240109	0.031174228
REGULATION OF VASCULAR ENDOTHELIAL GROWTH FACTOR RECEPTOR SIGNALING PATHWAY	27	-0.158563836	0.292611703
NEGATIVE REGULATION OF MRNA METABOLIC PROCESS	33	-0.018592274	0.895810447
REGULATION OF TRANSCRIPTION FROM RNA POLYMERASE I PROMOTER	23	-0.149410889	0.362664495
REGULATION OF DNA TEMPLATED TRANSCRIPTION INITIATION	28	-0.0794886	0.600224945
REGULATION OF CELL ADHESION MEDIATED BY INTEGRIN	34	-0.164715863	0.218198548
NUCLEOSIDE TRIPHOSPHATE BIOSYNTHETIC PROCESS	54	-0.284514782	0.004813992
GLYOXYLATE METABOLIC PROCESS	24	-0.187431725	0.234376522

RELAXATION OF MUSCLE	18	-0.042314953	0.824647144
REGULATION OF PROTEIN MODIFICATION BY SMALL PROTEIN CONJUGATION OR REMOVAL	258	-0.028482825	0.576864687
CELLULAR ANION HOMEOSTASIS	13	-0.294316701	0.154696599
TOXIN METABOLIC PROCESS	11	0.043101734	0.86239152
COVALENT CHROMATIN MODIFICATION	315	-0.087383215	0.05572328
REGULATION OF N METHYL D ASPARTATE SELECTIVE GLUTAMATE RECEPTOR ACTIVITY	11	0.232752814	0.349973097
PURINE CONTAINING COMPOUND BIOSYNTHETIC PROCESS	116	-0.075070044	0.315682461
NEGATIVE REGULATION OF CELL CYCLE ARREST	19	0.091023341	0.633165983
DNA DEMETHYLATION	14	0.113894528	0.608878621
POST EMBRYONIC MORPHOGENESIS	11	0.38737294	0.101875862
SKELETAL MUSCLE ORGAN DEVELOPMENT	116	-0.041575365	0.58192098
POSITIVE REGULATION OF RECEPTOR ACTIVITY	41	-0.132669261	0.2847234
FC RECEPTOR SIGNALING PATHWAY	166	-0.041798588	0.508678582
NEGATIVE REGULATION OF HOMEOSTATIC PROCESS	113	-0.091958516	0.222949713
APPENDAGE DEVELOPMENT	150	-0.069112087	0.295234635
REGULATION OF B CELL MEDIATED IMMUNITY	36	0.044269654	0.74777352
REGULATION OF MITOCHONDRIAL FISSION	17	-0.191967738	0.304337544
NEUTRAL LIPID CATABOLIC PROCESS	19	0.359668579	0.047600686
POSITIVE REGULATION OF CATECHOLAMINE SECRETION	12	-0.05792399	0.803622693
NEGATIVE REGULATION OF PEPTIDYL THREONINE PHOSPHORYLATION	15	-0.307839966	0.097765589
REGULATION OF CHEMOKINE PRODUCTION	53	-0.115065061	0.292001255
PROTEIN AUTOUBIQUITINATION	48	0.065661903	0.58339047
PORE COMPLEX ASSEMBLY	15	-0.322430558	0.087937621
PROTEIN LIPID COMPLEX SUBUNIT ORGANIZATION	28	0.281541458	0.069831051
NEGATIVE REGULATION OF T CELL APOPTOTIC PROCESS	15	0.172127019	0.423158254
NEGATIVE REGULATION OF PHOSPHORUS METABOLIC PROCESS	484	-0.016032337	0.670384355
4 HYDROXYPROLINE METABOLIC PROCESS	11	0.050735649	0.838735837
REGULATION OF CELL CYCLE G1 S PHASE TRANSITION	139	-0.102103781	0.133436942
ANION HOMEOSTASIS	35	0.05963075	0.670181958
LIPOPROTEIN BIOSYNTHETIC PROCESS	72	-0.102944814	0.2734036
RESPONSE TO FOLLICLE STIMULATING HORMONE	9	0.645621461	0.00514807
CELLULAR POTASSIUM ION HOMEOSTASIS	11	-0.076240517	0.752877308
PROTEIN MANNOSYLATION	20	0.104328851	0.575587228
REGULATION OF FATTY ACID TRANSPORT	22	0.100323579	0.571446752
HORMONE BIOSYNTHETIC PROCESS	36	0.130026462	0.348755588
ELECTRON TRANSPORT CHAIN	84	-0.164499501	0.053384089
REGULATION OF CIRCADIAN SLEEP WAKE CYCLE NON REM SLEEP	9	0.209577842	0.449785178
REGULATION OF PATHWAY RESTRICTED SMAD PROTEIN PHOSPHORYLATION	47	0.136042329	0.26434522
EXTRACELLULAR REGULATION OF SIGNAL TRANSDUCTION	11	0.086217785	0.730224648
NUCLEOTIDE EXCISION REPAIR DNA GAP FILLING	24	0.325158135	0.06027528
GLYCOSPHINGOLIPID BIOSYNTHETIC PROCESS	22	-0.02189507	0.89966222
RESPONSE TO ORGANIC CYCLIC COMPOUND	780	0.025332727	0.40190698
CELL COMMUNICATION BY ELECTRICAL COUPLING	14	-0.141948918	0.498378251
ONE CARBON METABOLIC PROCESS	30	0.047035262	0.755140904
TRANSEPITHELIAL TRANSPORT	10	0.377312671	0.141948111
VACUOLAR ACIDIFICATION	14	0.051685308	0.814855609
CELL DIFFERENTIATION IN HINDBRAIN	17	-0.086375029	0.656517628
REGULATION OF INTERLEUKIN 1 PRODUCTION	43	-0.069270276	0.57263748
POSITIVE REGULATION OF ANTIGEN PROCESSING AND PRESENTATION	10	0.073184198	0.779843983
NEGATIVE REGULATION OF CELL MORPHOGENESIS INVOLVED IN DIFFERENTIATION	105	0.038596507	0.63251876
REGULATION OF PEROXISOME PROLIFERATOR ACTIVATED RECEPTOR SIGNALING PATHWAY	9	0.201456629	0.472286164
POSITIVE REGULATION OF CD4 POSITIVE ALPHA BETA T CELL ACTIVATION	24	0.070570486	0.676537795
GLYCOPROTEIN CATABOLIC PROCESS	12	-0.061941285	0.790000874
REGULATION OF LYMPHOCYTE CHEMOTAXIS	17	-0.452920714	0.008218885
RENAL WATER HOMEOSTASIS	28	-0.034952618	0.819815288
REGULATION OF DNA METHYLATION	15	0.166169177	0.441736203
HEART VALVE FORMATION	10	0.220564159	0.40221832
MITOTIC NUCLEAR DIVISION	339	-0.0918236	0.036867874
NEGATIVE REGULATION OF MYELOID LEUKOCYTE DIFFERENTIATION	38	-0.14179282	0.267650589
FAT SOLUBLE VITAMIN METABOLIC PROCESS	26	0.233453235	0.150009347
ACTOMYOSIN STRUCTURE ORGANIZATION	63	-0.127839762	0.200479564
NUCLEOTIDE TRANSMEMBRANE TRANSPORT	12	-0.226789921	0.306303412

POSITIVE REGULATION OF CELL CYCLE PROCESS	219	-0.059711941	0.27689118
REGULATION OF CHONDROCYTE DIFFERENTIATION	38	-0.158044435	0.211341734
REGULATION OF PSEUDOPODIUM ASSEMBLY	12	0.24904975	0.292150077
PALLIUM DEVELOPMENT	136	0.035355566	0.618379958
REGULATION OF RESPONSE TO STRESS	1295	-0.025032897	0.290174
CARDIAC CELL FATE COMMITMENT	9	0.263944828	0.337822256
POSITIVE REGULATION OF INTERLEUKIN 1 PRODUCTION	28	0.070252564	0.653712965
MYOFIBRIL ASSEMBLY	37	-0.15127567	0.242923932
NEGATIVE REGULATION OF CHEMOTAXIS	44	0.190043822	0.131576846
BLOOD COAGULATION INTRINSIC PATHWAY	15	0.243364843	0.249885794
GLOMERULUS DEVELOPMENT	41	-0.123141786	0.31968311
TELOMERE MAINTENANCE VIA RECOMBINATION	32	-0.065485406	0.64569866
REGULATION OF RELEASE OF CYTOCHROME C FROM MITOCHONDRIA	42	-0.152491058	0.208091039
CELL CELL SIGNALING INVOLVED IN CARDIAC CONDUCTION	20	-0.002731196	0.98808776
CELLULAR RESPONSE TO THYROID HORMONE STIMULUS	13	-0.339484371	0.082113795
PHOSPHATE CONTAINING COMPOUND METABOLIC PROCESS	1732	-0.02597612	0.211745649
POSITIVE REGULATION OF NF KAPPAB TRANSCRIPTION FACTOR ACTIVITY	116	0.047155262	0.539947536
MITOCHONDRIAL RNA PROCESSING	13	0.004457277	0.984347534
CELLULAR LIPID CATABOLIC PROCESS	126	0.120983117	0.104322955
REGULATION OF CARDIAC MUSCLE CELL ACTION POTENTIAL	17	-0.239724888	0.191724205
POSITIVE REGULATION OF HAIR CYCLE	10	0.353143262	0.160266369
SYMPATHETIC NERVOUS SYSTEM DEVELOPMENT	19	0.011743945	0.950256164
REGULATION OF ALPHA BETA T CELL DIFFERENTIATION	40	0.109246747	0.407801643
MULTICELLULAR ORGANISM GROWTH	70	0.030926974	0.753815139
GRANULOCYTE MIGRATION	59	0.113777123	0.293368286
POSITIVE REGULATION OF ANTIGEN RECEPTOR MEDIATED SIGNALING PATHWAY	14	-0.087841235	0.681781521
NCRNA METABOLIC PROCESS	507	-0.129409664	0.000307009
PHOSPHATE ION TRANSPORT	12	-0.068460765	0.768095743
NEGATIVE REGULATION OF BMP SIGNALING PATHWAY	36	0.07450656	0.590283341
POSITIVE REGULATION OF NEURAL PRECURSOR CELL PROLIFERATION	32	-0.054719168	0.701683448
REGULATION OF CELLULAR EXTRAVASATION	20	0.146847508	0.429997254
CARDIAC MUSCLE TISSUE MORPHOGENESIS	47	0.215632524	0.076100049
GRANULOCYTE ACTIVATION	18	-0.082692388	0.661115278
REGULATION OF MACROPHAGE CYTOKINE PRODUCTION	11	0.279326948	0.261597192
PURINE NUCLEOBASE BIOSYNTHETIC PROCESS	11	0.021191096	0.931848139
MITOTIC G2 DNA DAMAGE CHECKPOINT	13	0.032173017	0.888003227
POSITIVE REGULATION OF PROTEIN AUTOPHOSPHORYLATION	21	0.151132711	0.406070105
NEGATIVE REGULATION OF NUCLEOCYTOPLASMIC TRANSPORT	65	0.039566464	0.699429408
POSITIVE REGULATION OF CELL ACTIVATION	248	-0.076698197	0.135778603
CARBOHYDRATE BIOSYNTHETIC PROCESS	110	-0.062937646	0.414277136
AMINO SUGAR METABOLIC PROCESS	35	0.045529386	0.744359827
POSITIVE REGULATION OF NUCLEOTIDE CATABOLIC PROCESS	15	-0.010284065	0.961096942
REGULATION OF CYTOKINESIS	53	-0.080768605	0.46413856
VITAMIN BIOSYNTHETIC PROCESS	15	-0.248937328	0.200255822
POSITIVE REGULATION OF NON CANONICAL WNT SIGNALING PATHWAY	10	0.647963659	0.004326601
MUSCLE ORGAN MORPHOGENESIS	61	0.195599828	0.067580449
AMINO ACID TRANSPORT	103	-0.065923562	0.407408583
CELLULAR RESPONSE TO EPINEPHRINE STIMULUS	10	0.294914635	0.249228563
POSITIVE REGULATION OF CELL ADHESION MEDIATED BY INTEGRIN	14	0.007697654	0.971971932
REGULATION OF MICROTUBULE BASED MOVEMENT	12	-0.223531823	0.304306583
EXOCRINE SYSTEM DEVELOPMENT	39	0.05404306	0.683450645
AMINOGLYCAN METABOLIC PROCESS	143	-0.075329704	0.264207671
NEGATIVE REGULATION OF MYOBLAST DIFFERENTIATION	19	-0.157407003	0.38185171
REGULATION OF FEVER GENERATION	11	-0.046146626	0.849905329
DNA TEMPLATED TRANSCRIPTIONAL PREINITIATION COMPLEX ASSEMBLY	10	-0.080991702	0.74888712
MACROPHAGE ACTIVATION	27	0.091820934	0.565348708
CARDIOBLAST DIFFERENTIATION	12	0.216588231	0.380555046
CELL CYCLE G1 S PHASE TRANSITION	108	-0.18878695	0.011975757
RNA SPLICING VIA TRANSESTERIFICATION REACTIONS	255	-0.140201335	0.004882334
BLOOD VESSEL MORPHOGENESIS	332	0.024536218	0.590685368
REGULATION OF URINE VOLUME	17	0.010599706	0.957511274
PHOTORECEPTOR CELL DEVELOPMENT	31	0.009934847	0.946255706
SYNAPTIC TRANSMISSION DOPAMINERGIC	10	0.081658483	0.755154746
PROTEIN PHOSPHORYLATION	822	0.019964554	0.497877347
CENTRAL NERVOUS SYSTEM NEURON DIFFERENTIATION	133	0.070373146	0.329498174

REGULATION OF CELL DIFFERENTIATION	1292	0.038963807	0.103976844
MAINTENANCE OF GASTROINTESTINAL EPITHELIUM	13	0.09242898	0.687980663
ENDOTHELIUM DEVELOPMENT	80	-0.113129755	0.204633504
DOUBLE STRAND BREAK REPAIR	154	0.01961905	0.768059524
NUCLEOBASE CONTAINING COMPOUND TRANSPORT	185	-0.242023313	2.19E-05
REGULATION OF NEURON MIGRATION	25	0.256962883	0.12357575
METHIONINE BIOSYNTHETIC PROCESS	14	0.041936229	0.849191764
MULTI ORGANISM ORGANELLE ORGANIZATION	23	0.381623481	0.031400529
MESONEPHRIC TUBULE MORPHOGENESIS	47	-0.094493754	0.417759147
RELEASE OF CYTOCHROME C FROM MITOCHONDRIA	20	-0.010321966	0.954925178
REGULATION OF CYTOKINE SECRETION INVOLVED IN IMMUNE RESPONSE	10	0.214335188	0.410065929
RESPONSE TO INTERLEUKIN 1	97	-0.003342822	0.967976519
EMBRYONIC HINDLIMB MORPHOGENESIS	24	0.142733675	0.401397099
WATER SOLUBLE VITAMIN METABOLIC PROCESS	81	-0.105070226	0.235371301
SIGNAL TRANSDUCTION IN ABSENCE OF LIGAND	29	0.235196215	0.133059074
RNA CAPPING	37	-0.142736935	0.269234446
MALE MEIOSIS	28	0.134252421	0.393688509
LIVER REGENERATION	16	-0.161597266	0.411278396
REGULATION OF REACTIVE OXYGEN SPECIES METABOLIC PROCESS	139	0.029442497	0.674536529
REGULATION OF RENAL SODIUM EXCRETION	17	0.309819114	0.113594752
NEGATIVE REGULATION OF DEFENSE RESPONSE	121	-0.03842072	0.603623186
SPINAL CORD PATTERNING	16	0.216989655	0.30193646
DNA REPLICATION INITIATION	28	-0.031101216	0.839540246
ERYTHROCYTE MATURATION	8	-0.110918259	0.694221819
ETHANOLAMINE CONTAINING COMPOUND METABOLIC PROCESS	64	0.115396197	0.268281689
POSITIVE REGULATION OF DENDRITE DEVELOPMENT	59	-0.140636169	0.171784975
MEMBRANE DEPOLARIZATION DURING CARDIAC MUSCLE CELL ACTION POTENTIAL	13	-0.354744953	0.072425864
BASIC AMINO ACID TRANSPORT	10	-0.137522138	0.579455697
REGULATION OF CAMP MEDIATED SIGNALING	19	0.261802521	0.166090181
CELL MIGRATION INVOLVED IN GASTRULATION	13	0.091987519	0.689392076
ASTROCYTE DIFFERENTIATION	33	0.272835301	0.060640423
PROTEIN MONOUBIQUITINATION	50	-0.03037958	0.791810187
POSITIVE REGULATION OF ALPHA BETA T CELL PROLIFERATION	17	-0.073583728	0.705777987
FACE DEVELOPMENT	45	0.047779529	0.698428827
RNA DESTABILIZATION	15	-0.179698114	0.367299227
RESPONSE TO ESTROGEN	190	0.001575714	0.978958294
DETOXIFICATION	65	-0.099517497	0.31451052
REGULATION OF OSTEOBLAST PROLIFERATION	21	0.24886215	0.167543888
CILIUM OR FLAGELLUM DEPENDENT CELL MOTILITY	10	0.013580977	0.958261723
VERY LONG CHAIN FATTY ACID METABOLIC PROCESS	25	0.235711773	0.154533164
ESTABLISHMENT OF PROTEIN LOCALIZATION TO MEMBRANE	251	-0.032869059	0.524867104
SMALL GTPASE MEDIATED SIGNAL TRANSDUCTION	317	0.024587948	0.598262256
REGULATION OF PEPTIDASE ACTIVITY	323	-0.0146879	0.748792966
POSITIVE REGULATION OF PROTEIN SERINE THREONINE KINASE ACTIVITY	259	-0.062446184	0.216582022
BROWN FAT CELL DIFFERENTIATION	27	0.257474216	0.105554648
LIPID TRANSLOCATION	17	0.680001966	0.000143585
INOSITOL METABOLIC PROCESS	10	-0.0214266	0.933682357
REGULATION OF CENTROSOME CYCLE	36	0.002330035	0.986387841
POSITIVE REGULATION OF TRANSFERASE ACTIVITY	555	-0.051822325	0.13858885
REGULATION OF IMMUNE SYSTEM PROCESS	1155	-0.054049304	0.029255453
PROTEIN ADP RIBOSYLATION	18	-0.154387011	0.403672911
CELLULAR SODIUM ION HOMEOSTASIS	17	0.386446659	0.052165027
NEGATIVE REGULATION OF CARTILAGE DEVELOPMENT	21	-0.095336048	0.584120632
CHONDROITIN SULFATE BIOSYNTHETIC PROCESS	24	-0.068456339	0.677051504
PROTEIN SUMOYLATION	114	-0.224852407	0.001805643
MEGAKARYOCYTE DEVELOPMENT	14	-0.371423424	0.050745369
REGULATION OF HISTONE H3 K9 METHYLATION	16	0.146059317	0.485770612
VASODILATION	22	0.187431604	0.28986039
NEGATIVE REGULATION OF MITOTIC CELL CYCLE	190	-0.131823041	0.022523554
NEGATIVE REGULATION OF MOLECULAR FUNCTION	921	-0.030656939	0.267539255
NEURONAL ION CHANNEL CLUSTERING	9	-0.068026293	0.799563509
POSITIVE REGULATION OF AXON EXTENSION	36	-0.065308511	0.626853378
REGULATION OF ION HOMEOSTASIS	163	-0.093080316	0.138994119
PIGMENT CELL DIFFERENTIATION	26	0.277865539	0.08304757
ORGAN GROWTH	60	-0.023894258	0.820390954

REGULATION OF LYMPHOCYTE DIFFERENTIATION	118	0.030352174	0.68984491
REGULATION OF BONE REMODELING	36	-0.088047193	0.509677609
GLUCOCORTICOID BIOSYNTHETIC PROCESS	4	0.185229973	0.65349627
REGULATION OF T CELL PROLIFERATION	125	-0.082705435	0.25060035
NERVE DEVELOPMENT	59	-0.027753113	0.793540664
REGULATION OF NEUROBLAST PROLIFERATION	22	-0.089714839	0.598436165
DETECTION OF MOLECULE OF BACTERIAL ORIGIN	7	0.160976133	0.60825399
PHAGOSOME ACIDIFICATION	24	0.287179982	0.091004866
REGULATION OF CELL SIZE	158	-0.059641766	0.355292157
TETRAPYRROLE METABOLIC PROCESS	49	-0.164857074	0.1391722
REGULATION OF CENTROSOME DUPLICATION	29	0.13402699	0.388091656
VENTRAL SPINAL CORD INTERNEURON DIFFERENTIATION	10	0.461098907	0.076040141
NEGATIVE REGULATION OF TRANSFERASE ACTIVITY	316	-0.145227517	0.001206972
BICELLULAR TIGHT JUNCTION ASSEMBLY	28	-0.200221594	0.169300935
POSITIVE REGULATION OF CARDIAC MUSCLE TISSUE DEVELOPMENT	27	0.166893236	0.298538757
RIBONUCLEOPROTEIN COMPLEX SUBUNIT ORGANIZATION	189	-0.23850265	1.94E-05
REGULATION OF CALCIUM ION TRANSPORT INTO CYTOSOL	74	-0.291119142	0.000772146
MULTICELLULAR ORGANISMAL WATER HOMEOSTASIS	45	-0.032708654	0.787248552
REGULATION OF ALTERNATIVE MRNA SPLICING VIA SPLICEOSOME	32	0.173889176	0.242157392
KERATAN SULFATE CATABOLIC PROCESS	9	-0.027236726	0.919954451
BLASTOCYST DEVELOPMENT	59	-0.066879545	0.52427503
REGULATION OF GRANULOCYTE CHEMOTAXIS	36	0.179496533	0.19595984
CHROMATIN ORGANIZATION	592	-0.024620558	0.470645144
POSITIVE REGULATION OF CYTOKINESIS	26	-0.10944993	0.482838455
EPHRIN RECEPTOR SIGNALING PATHWAY	77	-0.244007691	0.005262636
ORGANIC ANION TRANSPORT	315	0.036045545	0.442192686
STAT CASCADE	44	-0.108845167	0.364503865
RESPONSE TO OXYGEN CONTAINING COMPOUND	1176	-0.016671533	0.50081782
BICARBONATE TRANSPORT	32	0.067624749	0.64421045
POSITIVE REGULATION OF EXCITATORY POSTSYNAPTIC POTENTIAL	16	-0.214212019	0.258566898
NEGATIVE REGULATION OF PATHWAY RESTRICTED SMAD PROTEIN PHOSPHORYLATION	10	0.540433872	0.022216565
SEGMENTATION	77	0.058372298	0.536573781
POSITIVE REGULATION OF PEPTIDASE ACTIVITY	139	-0.070641264	0.302749801
POSITIVE REGULATION OF T CELL CYTOKINE PRODUCTION	13	0.003755179	0.986810855
PYRIMIDINE CONTAINING COMPOUND SALVAGE	11	-0.199895644	0.385724121
REGULATION OF OLIGODENDROCYTE DIFFERENTIATION	26	-0.029724091	0.852154288
TRNA METHYLATION	22	-0.200461908	0.220615861
INTERACTION WITH HOST	129	0.015782403	0.827834074
GASTRULATION	139	-0.119377217	0.077438171
RESPONSE TO UV	115	-0.260460974	0.000226085
REGULATION OF NEUROTRANSMITTER TRANSPORT	52	0.004350553	0.969488825
NEGATIVE REGULATION OF VASCULATURE DEVELOPMENT	72	0.070913751	0.468592724
SKIN EPIDERMIS DEVELOPMENT	63	0.065569155	0.530327268
VIRAL GENOME REPLICATION	17	0.148142161	0.467405821
NUCLEOTIDE EXCISION REPAIR	106	0.102170066	0.207846422
POSITIVE REGULATION OF REACTIVE OXYGEN SPECIES METABOLIC PROCESS	78	0.079198124	0.40009588
NEGATIVE REGULATION OF RELEASE OF CYTOCHROME C FROM MITOCHONDRIA	16	-0.441169653	0.011699156
GOLGI TO ENDOSOME TRANSPORT	18	-0.189217309	0.308286047
REGULATION OF MUSCLE HYPERTROPHY	33	0.105687876	0.467098587
CARBOHYDRATE TRANSMEMBRANE TRANSPORT	17	-0.266928025	0.137239005
SULFUR COMPOUND TRANSPORT	25	-0.351118752	0.015153626
FATTY ACID BIOSYNTHETIC PROCESS	88	-0.025349891	0.770641956
HEART FORMATION	11	0.093098009	0.709905119
RECEPTOR INTERNALIZATION	41	0.110886904	0.394409457
POSITIVE REGULATION OF MAST CELL ACTIVATION	13	0.068358611	0.76582415
REGULATION OF ODONTOGENESIS	17	-0.119413077	0.534473904
MULTICELLULAR ORGANISM METABOLIC PROCESS	73	-0.133831264	0.14836787
PARTURITION	17	-0.003051905	0.987725771
NEGATIVE REGULATION OF ANDROGEN RECEPTOR SIGNALING PATHWAY	11	-0.256393886	0.253327578
ALPHA LINOLENIC ACID METABOLIC PROCESS	11	0.329747764	0.171102296
REGULATION OF ARP2 3 COMPLEX MEDIATED ACTIN NUCLEATION	14	-0.016264865	0.9405111
HOMEOSTASIS OF NUMBER OF CELLS	155	-0.09962091	0.122432675
MEIOTIC CHROMOSOME SEPARATION	9	-0.543855092	0.007194643
REGULATION OF CALCINEURIN NFAT SIGNALING CASCADE	16	0.074747396	0.718336046

DIOL METABOLIC PROCESS	10	-0.085833094	0.734209027
REGULATION OF RYANODINE SENSITIVE CALCIUM RELEASE CHANNEL ACTIVITY	23	-0.300482092	0.047973302
OXYGEN TRANSPORT	9	0.057859682	0.833760725
REGULATION OF LIPID TRANSPORT	74	0.211149478	0.030832427
POSITIVE REGULATION OF TRIGLYCERIDE BIOSYNTHETIC PROCESS	13	0.202375145	0.385909697
REGULATION OF REPRODUCTIVE PROCESS	105	0.046753249	0.563025942
REGULATION OF MEMBRANE PROTEIN ECTODOMAIN PROTEOLYSIS	19	0.19303306	0.309348461
POSITIVE REGULATION OF HORMONE SECRETION	95	0.007393709	0.930129694
ORGAN INDUCTION	14	-0.303307046	0.12200276
DNA STRAND ELONGATION	30	0.164595645	0.281144143
NEURON PROJECTION MORPHOGENESIS	350	0.109110532	0.015456643
REGULATION OF CELL DIVISION	235	-0.03827966	0.47281154
MULTIVESICULAR BODY SORTING PATHWAY	11	0.16669531	0.505900161
REGULATION OF LYMPHOCYTE MEDIATED IMMUNITY	95	-0.017761578	0.832259615
SOMATIC DIVERSIFICATION OF IMMUNE RECEPTORS	37	-0.099668006	0.448312728
NOTCH SIGNALING PATHWAY	98	-0.039171871	0.633546806
OUTER DYNEIN ARM ASSEMBLY	7	0.528168455	0.07907331
POSITIVE REGULATION OF CELLULAR RESPONSE TO INSULIN STIMULUS	20	-0.079471929	0.657790166
RESPONSE TO RADIATION	356	-0.076813428	0.074708478
BILE ACID AND BILE SALT TRANSPORT	19	0.125258897	0.512706282
HEART DEVELOPMENT	422	-0.029066687	0.468973727
TISSUE REGENERATION	43	0.201367303	0.114505626
MESODERM MORPHOGENESIS	59	-0.021337005	0.840761134
NEGATIVE REGULATION OF SMOOTHENED SIGNALING PATHWAY	23	0.053001629	0.758628858
RECEPTOR METABOLIC PROCESS	71	0.046717423	0.634254443
NUCLEOSIDE SALVAGE	16	-0.303092229	0.097356339
INFLAMMATORY RESPONSE	340	0.011063814	0.805672301
TRNA TRANSPORT	33	-0.371982427	0.00811465
REGULATION OF NIK NF KAPPAB SIGNALING	38	-0.07512965	0.564277431
NEGATIVE REGULATION OF ANOIKIS	16	-0.046830244	0.816972332
POSITIVE REGULATION OF LIGASE ACTIVITY	101	-0.096770777	0.224357047
LACTATE TRANSMEMBRANE TRANSPORT	9	-0.488690881	0.031381859
REGULATION OF TRANSCRIPTION FROM RNA POLYMERASE II PROMOTER IN RESPONSE TO HYPOXIA	29	0.247444774	0.115826037
REGULATION OF INTRACELLULAR PROTEIN TRANSPORT	337	-0.072261303	0.103052145
PIGMENT GRANULE ORGANIZATION	21	0.134517554	0.459307202
REGULATION OF INTERLEUKIN 13 PRODUCTION	15	0.412551827	0.062513127
REGULATION OF CELL CYCLE	868	-0.047951156	0.090532546
TUBE DEVELOPMENT	486	-0.003776349	0.920187981
LOCOMOTION	955	0.009527304	0.728043374
ACROSOME ASSEMBLY	9	-0.005028083	0.985276084
REGULATION OF INOSITOL PHOSPHATE BIOSYNTHETIC PROCESS	11	0.094680545	0.704909463
REGULATION OF INTRINSIC APOPTOTIC SIGNALING PATHWAY IN RESPONSE TO DNA DAMAGE BY P53 CLASS MEDIATOR	14	-0.164114953	0.429294304
POSITIVE REGULATION OF EPIDERMIS DEVELOPMENT	28	0.200490567	0.203936772
RESPONSE TO EXTRACELLULAR STIMULUS	389	0.009783164	0.816259189
REGULATION OF PROTEIN COMPLEX ASSEMBLY	334	0.078415739	0.087571935
CALCIUM ION REGULATED EXOCYTOSIS OF NEUROTRANSMITTER	24	0.03178504	0.850092836
REGULATION OF REGULATED SECRETORY PATHWAY	104	-0.075343129	0.34021666
POSITIVE REGULATION OF RECEPTOR INTERNALIZATION	21	-0.003483501	0.98442968
CARDIAC MUSCLE CELL CONTRACTION	24	-0.166738761	0.296891113
AMINO SUGAR CATABOLIC PROCESS	9	0.29691469	0.287235571
POSITIVE REGULATION OF MACROPHAGE CHEMOTAXIS	10	0.02669579	0.918263058
REGULATION OF B CELL APOPTOTIC PROCESS	17	-0.328510695	0.069294325
MITOCHONDRIAL RNA METABOLIC PROCESS	27	-0.029566484	0.850139988
MODULATION OF EXCITATORY POSTSYNAPTIC POTENTIAL	24	-0.039599187	0.810928398
NUCLEUS LOCALIZATION	19	0.110992387	0.562947419
RESPONSE TO HEPATOCYTE GROWTH FACTOR	12	-0.200060093	0.363934952
REGULATION OF SYNAPTIC VESICLE EXOCYTOSIS	17	-0.009467342	0.961882074
ORGANELLE ASSEMBLY	431	-0.038732403	0.328593211
MITOTIC DNA INTEGRITY CHECKPOINT	95	-0.102527768	0.21189492
FATTY ACID DERIVATIVE TRANSPORT	17	0.015394753	0.938381065
NATURAL KILLER CELL ACTIVATION INVOLVED IN IMMUNE RESPONSE	12	0.086828705	0.716833551
CARDIAC VENTRICLE DEVELOPMENT	96	0.079412943	0.350107653
MALE MEIOSIS I	12	-0.130573648	0.565274331

GLYCEROLIPID BIOSYNTHETIC PROCESS	183	-0.064714136	0.279901847
REGULATION OF GLUCOSE METABOLIC PROCESS	94	-0.074233257	0.371751935
POSITIVE REGULATION OF SODIUM ION TRANSMEMBRANE TRANSPORT	15	-0.070718577	0.733717759
POSITIVE REGULATION OF HEART CONTRACTION	30	0.255065051	0.089870894
CELLULAR RESPONSE TO PROSTAGLANDIN E STIMULUS	17	0.4023312	0.034127383
EXTRINSIC APOPTOTIC SIGNALING PATHWAY VIA DEATH DOMAIN RECEPTORS	35	-0.210051684	0.106139174
PURINE NUCLEOSIDE BIOSYNTHETIC PROCESS	78	-0.0932614	0.303470105
FEMALE SEX DIFFERENTIATION	98	-0.016140439	0.845084093
POSITIVE REGULATION OF CIRCADIAN RHYTHM	14	0.285019554	0.191018916
ECTODERMAL PLACODE DEVELOPMENT	12	-0.279424854	0.199872756
POSITIVE REGULATION OF PHAGOCYTOSIS	37	0.154062138	0.260625614
CELLULAR RESPONSE TO ETHANOL	13	0.095782533	0.677076802
RESPIRATORY BURST	10	-0.066426937	0.793879449
POSITIVE REGULATION OF DNA REPAIR	35	0.10646378	0.450023345
CEREBRAL CORTEX GABAERGIC INTERNEURON DIFFERENTIATION	7	-0.029278863	0.924075995
NIK NF KAPPAB SIGNALING	79	-0.167471593	0.056821008
MAMMARY GLAND DEVELOPMENT	102	-0.075498108	0.344156531
NEGATIVE REGULATION OF SECRETION	161	0.0144571	0.824013072
MITOTIC SPINDLE ORGANIZATION	64	-0.124752328	0.209755785
REGULATION OF PROTEIN CATABOLIC PROCESS	370	-0.02742516	0.521732778
REGULATION OF CD4 POSITIVE ALPHA BETA T CELL ACTIVATION	33	0.142501442	0.327443802
MAINTENANCE OF CELL POLARITY	11	0.138428728	0.581541575
MRNA CLEAVAGE	20	-0.057025269	0.752198114
POSITIVE REGULATION OF URINE VOLUME	12	0.092207977	0.700395698
GLYCINE METABOLIC PROCESS	16	0.148806936	0.474489815
POSITIVE REGULATION OF CHEMOKINE PRODUCTION	40	-0.171832372	0.164381785
REGULATION OF GLYCOGEN METABOLIC PROCESS	30	-0.07465146	0.610991097
ESTABLISHMENT OF NUCLEUS LOCALIZATION	14	0.12080728	0.589806383
ENDODERM DEVELOPMENT	66	-0.219297446	0.020816277
VESICLE COATING	72	-0.099082122	0.292983155
CYTOPLASMIC SEQUESTERING OF TRANSCRIPTION FACTOR	19	0.263930403	0.1765662
PROTEIN TRANSPORT ALONG MICROTUBULE	23	-0.042824933	0.799973491
AUDITORY RECEPTOR CELL DIFFERENTIATION	22	-0.1231718	0.465343324
REGULATION OF LIPOPOLYSACCHARIDE MEDIATED SIGNALING PATHWAY	17	-0.223809157	0.22603016
CAMERA TYPE EYE PHOTORECEPTOR CELL DIFFERENTIATION	11	0.042667255	0.863806787
REGULATION OF GROWTH HORMONE SECRETION	8	-0.260966352	0.327630088
PYRIMIDINE CONTAINING COMPOUND METABOLIC PROCESS	68	-0.204457831	0.028503967
MICROGLIAL CELL ACTIVATION	10	0.200868112	0.44049878
OXIDATION REDUCTION PROCESS	749	-0.014903163	0.62588443
RESPONSE TO ATP	24	-0.340344928	0.019314443
WATER HOMEOSTASIS	50	0.034488864	0.767531691
VOCALIZATION BEHAVIOR	13	0.064174995	0.779778998
GOLGI TO PLASMA MEMBRANE PROTEIN TRANSPORT	23	-0.078320083	0.640187606
LONG TERM MEMORY	21	-0.058206767	0.741052401
ADRENAL GLAND DEVELOPMENT	17	-0.023333559	0.90595287
CELLULAR RESPONSE TO CHOLESTEROL	11	0.34026419	0.167908959
POSITIVE REGULATION OF POTASSIUM ION TRANSMEMBRANE TRANSPORTER ACTIVITY	13	-0.070470869	0.75220938
VIRAL LIFE CYCLE	282	-0.007034643	0.886078744
REGULATION OF SMOOTH MUSCLE CELL MIGRATION	46	0.031923242	0.792832815
ENTRAINMENT OF CIRCADIAN CLOCK	23	-0.170477023	0.298123961
REGULATION OF CGMP METABOLIC PROCESS	21	0.079998043	0.65828278
REGULATION OF CYTOKINE SECRETION	111	0.057338452	0.466617069
CELLULAR RESPONSE TO AMINO ACID STARVATION	25	-0.111060661	0.484653973
POSITIVE REGULATION OF IMMUNOGLOBULIN PRODUCTION	27	-0.111354995	0.46743824
REGULATION OF ESTABLISHMENT OF PROTEIN LOCALIZATION TO MITOCHONDRION	109	-0.125190445	0.099572805
REGULATION OF SENSORY PERCEPTION	30	-0.026043895	0.86086553
REGULATION OF REGULATORY T CELL DIFFERENTIATION	10	-0.002017574	0.993776379
CHONDROITIN SULFATE CATABOLIC PROCESS	14	0.056976267	0.796423705
RESPONSE TO AUDITORY STIMULUS	16	-0.271276585	0.14742889
PRESYNAPTIC PROCESS INVOLVED IN SYNAPTIC TRANSMISSION	89	-0.001759751	0.983850139
PROTEIN UBIQUITINATION	570	-0.022547787	0.516645032
MAINTENANCE OF CELL NUMBER	123	0.013341998	0.857428747
CELL MORPHOGENESIS INVOLVED IN DIFFERENTIATION	454	0.081435342	0.039500396
NEGATIVE REGULATION OF CHROMOSOME ORGANIZATION	91	-0.107850862	0.197249413

COENZYME BIOSYNTHETIC PROCESS	110	-0.11467801	0.130881278
EPIBOLY	19	-0.073565633	0.690250267
NEGATIVE REGULATION OF INFLAMMATORY RESPONSE	81	0.12649166	0.172699318
REGULATION OF CALCIUM ION DEPENDENT EXOCYTOSIS	65	-0.01380761	0.891656938
CENTRAL NERVOUS SYSTEM PROJECTION NEURON AXONOGENESIS	19	-0.001362435	0.99420952
INFLAMMATORY RESPONSE TO ANTIGENIC STIMULUS	20	0.068667388	0.710598727
INTESTINAL EPITHELIAL CELL DIFFERENTIATION	16	0.061470633	0.766482165
ORGANOPHOSPHATE CATABOLIC PROCESS	103	0.038405676	0.63747119
NEGATIVE REGULATION OF BEHAVIOR	9	0.395200818	0.125823839
POSITIVE REGULATION OF P38MAPK CASCADE	12	0.299543186	0.209280428
MEMBRANE PROTEIN INTRACELLULAR DOMAIN PROTEOLYSIS	14	-0.394657478	0.040553316
POSITIVE REGULATION OF CHROMATIN MODIFICATION	81	-0.119552344	0.175487425
RESPONSE TO CAFFEINE	18	-0.310187103	0.073707518
DISTAL TUBULE DEVELOPMENT	9	0.493316506	0.045206881
CRANIAL SKELETAL SYSTEM DEVELOPMENT	51	0.047973789	0.67863966
DNA TEMPLATED TRANSCRIPTION INITIATION	189	0.096961529	0.11107605
REGULATION OF HORMONE BIOSYNTHETIC PROCESS	15	0.290858422	0.171705561
DEOXYRIBONUCLEOTIDE METABOLIC PROCESS	33	0.027946655	0.84543062
POSITIVE REGULATION OF NEUROTRANSMITTER SECRETION	10	-0.172653338	0.481543592
GENETIC IMPRINTING	15	0.229641206	0.280939495
CELLULAR PROCESS INVOLVED IN REPRODUCTION IN MULTICELLULAR ORGANISM	193	0.08284903	0.168146777
POSITIVE REGULATION OF CELL DEATH	536	-0.028953688	0.418138699
TROPHOBLAST GIANT CELL DIFFERENTIATION	12	0.003459807	0.988323818
ER ASSOCIATED UBIQUITIN DEPENDENT PROTEIN CATABOLIC PROCESS	58	-0.247246801	0.013877052
ISOTYPE SWITCHING	15	-0.34555672	0.074254438
TUMOR NECROSIS FACTOR MEDIATED SIGNALING PATHWAY	103	-0.144767155	0.062373807
DEFENSE RESPONSE TO GRAM NEGATIVE BACTERIUM	31	-0.051723891	0.721763148
SIGNAL RELEASE	133	-0.020988023	0.767254261
REGULATION OF EXOSOMAL SECRETION	16	0.218768259	0.308566735
REGULATION OF INCLUSION BODY ASSEMBLY	14	0.101792652	0.647027662
REGULATION OF CHRONIC INFLAMMATORY RESPONSE	10	-0.053302898	0.834748657
REGULATION OF T CELL DIFFERENTIATION	95	0.058811289	0.48956502
ADAPTIVE IMMUNE RESPONSE	192	-0.003771118	0.949350996
TRANSLESION SYNTHESIS	39	-0.083629936	0.515771486
REGULATION OF GLOMERULUS DEVELOPMENT	13	0.010443318	0.963382247
METHIONINE METABOLIC PROCESS	17	0.014249402	0.942946412
DICARBOXYLIC ACID TRANSPORT	61	-0.18122908	0.068298408
NEGATIVE REGULATION OF SKELETAL MUSCLE TISSUE DEVELOPMENT	10	0.416681648	0.098308707
DNA RECOMBINATION	187	-0.040343852	0.498756414
POST TRANSLATIONAL PROTEIN MODIFICATION	31	-0.004967233	0.97302813
PROTEIN FOLDING	197	-0.114098824	0.045340904
GLYCOSPHINGOLIPID METABOLIC PROCESS	61	0.004144921	0.968524194
REGULATION OF MRNA POLYADENYLATION	11	-0.119525659	0.617019755
NEURON MIGRATION	95	-0.140140275	0.083406259
REGULATION OF ATTACHMENT OF SPINDLE MICROTUBULES TO KINETOCHORE	11	0.245509568	0.337469688
POSITIVE REGULATION OF RESPONSE TO TUMOR CELL	7	-0.226945463	0.425527115
REGULATION OF ACTION POTENTIAL	32	-0.137486542	0.325049418
REGULATION OF NF KAPPAB IMPORT INTO NUCLEUS	42	0.123005053	0.339586647
POSITIVE REGULATION OF TISSUE REMODELING	24	0.086430648	0.610710072
REGULATION OF PROTEASOMAL PROTEIN CATABOLIC PROCESS	173	-0.015750821	0.800527648
DORSAL VENTRAL NEURAL TUBE PATTERNING	16	-0.045818781	0.820957103
TOLL LIKE RECEPTOR 4 SIGNALING PATHWAY	14	0.139200415	0.530813704
POSITIVE REGULATION OF MYELOID CELL DIFFERENTIATION	71	0.053220514	0.58815864
CALCIUM ION REGULATED EXOCYTOSIS	64	-0.005111677	0.960170468
CELLULAR RESPONSE TO NITROGEN COMPOUND	452	-0.065623976	0.088193523
RETINAL METABOLIC PROCESS	11	0.152007323	0.544465221
MOLTING CYCLE	73	0.099430569	0.307701714
CHROMOSOME ORGANIZATION INVOLVED IN MEIOTIC CELL CYCLE	40	0.21984667	0.095237949
POSITIVE REGULATION OF PEPTIDYL THREONINE PHOSPHORYLATION	23	0.12487257	0.472614984
OOCYTE MATURATION	14	0.12257962	0.581839822
DNA BIOSYNTHETIC PROCESS	107	-0.03802905	0.628861541
SUBSTRATE ADHESION DEPENDENT CELL SPREADING	35	-0.124525913	0.352293883
SPERMATID NUCLEUS DIFFERENTIATION	13	-0.10807145	0.624085794
MULTI ORGANISM LOCALIZATION	66	-0.048101593	0.629966203
NEGATIVE REGULATION OF CELL DEATH	770	-0.031234225	0.298957409

TRANSCRIPTION INITIATION FROM RNA POLYMERASE II PROMOTER	142	0.06095427	0.382267808
POSITIVE REGULATION OF DEVELOPMENTAL GROWTH	143	0.015517503	0.821945987
REGULATION OF ERYTHROCYTE DIFFERENTIATION	33	0.002533593	0.985828052
METANEPHRIC NEPHRON DEVELOPMENT	26	0.096423343	0.554162984
POSITIVE REGULATION OF NEUROLOGICAL SYSTEM PROCESS	20	0.254661773	0.171095573
TRACHEA DEVELOPMENT	19	0.123895148	0.519170865
NEUROGENESIS	1216	0.031752204	0.196882667
POSITIVE REGULATION OF T HELPER CELL DIFFERENTIATION	16	-0.02758949	0.8920602
POSITIVE REGULATION OF SEQUENCE SPECIFIC DNA BINDING TRANSCRIPTION FACTOR ACTIVITY	201	0.051941677	0.375938916
NEGATIVE REGULATION OF HUMORAL IMMUNE RESPONSE	12	0.067546911	0.777577603
POSITIVE REGULATION OF AMINE TRANSPORT	25	0.156877822	0.347157467
ENDOMEMBRANE SYSTEM ORGANIZATION	432	0.003393122	0.932331222
APOPTOTIC PROCESS INVOLVED IN DEVELOPMENT	19	0.333454388	0.070261375
RESPONSE TO LOW DENSITY LIPOPROTEIN PARTICLE	13	-0.244703042	0.250861974
RETINOIC ACID METABOLIC PROCESS	15	0.280722309	0.18074979
GLYCEROPHOSPHOLIPID METABOLIC PROCESS	254	0.00605256	0.907036554
REGULATION OF PROTEIN ACETYLATION	59	0.011698064	0.912916943
REGULATION OF NEUROLOGICAL SYSTEM PROCESS	58	0.141077184	0.197712711
INTRINSIC APOPTOTIC SIGNALING PATHWAY	140	-0.01083733	0.875723349
SODIUM ION HOMEOSTASIS	24	0.329542703	0.048888411
CELLULAR RESPONSE TO HEAT	32	-0.415610911	0.001178483
T CELL RECEPTOR SIGNALING PATHWAY	124	-0.137658268	0.0527703
POSITIVE REGULATION OF LAMELLIPODIUM ASSEMBLY	15	0.171812241	0.43147224
PURINE NUCLEOTIDE TRANSPORT	10	-0.180248477	0.465236278
REGULATION OF PROTEIN STABILITY	206	-0.01566743	0.78413038
REGULATION OF DNA METABOLIC PROCESS	309	-0.018504491	0.692762909
NADH METABOLIC PROCESS	30	0.309206661	0.041750424
POSITIVE REGULATION OF NUCLEOCYTOPLASMIC TRANSPORT	109	0.013859264	0.860500668
REGULATION OF BLOOD VESSEL ENDOTHELIAL CELL MIGRATION	49	0.022724079	0.84677107
NATURAL KILLER CELL MEDIATED IMMUNITY	20	0.291347026	0.111639074
TELOMERE MAINTENANCE VIA TELOMERASE	16	-0.073288949	0.715608861
REGULATION OF GRANULOCYTE MACROPHAGE COLONY STIMULATING FACTOR PRODUCTION	14	0.155307613	0.484235095
GROWTH	353	0.048804676	0.272421664
EMBRYONIC MORPHOGENESIS	468	-0.071546858	0.058408196
LIPID PARTICLE ORGANIZATION	14	0.213208518	0.335657793
NEGATIVE REGULATION OF MULTICELLULAR ORGANISM GROWTH	11	0.094105276	0.707669271
NEGATIVE REGULATION OF LYMPHOCYTE APOPTOTIC PROCESS	25	-0.0208644	0.898127484
OOCYTE DIFFERENTIATION	31	0.083598821	0.574999092
JAK STAT CASCADE INVOLVED IN GROWTH HORMONE SIGNALING PATHWAY	14	-0.2833945	0.155591408
INTERSTRAND CROSS LINK REPAIR	41	-0.054112782	0.668411998
REGULATION OF STEM CELL POPULATION MAINTENANCE	15	0.136965077	0.524649338
POSITIVE REGULATION OF TELOMERE MAINTENANCE VIA TELOMERE LENGTHENING	30	-0.01052036	0.943769301
GANGLIOSIDE BIOSYNTHETIC PROCESS	16	-0.050311635	0.803369218
POSITIVE REGULATION OF ENDOPLASMIC RETICULUM UNFOLDED PROTEIN RESPONSE	13	-0.06934342	0.756088469
ANDROGEN METABOLIC PROCESS	23	-0.065222469	0.697605238
NEGATIVE REGULATION OF EPIDERMIS DEVELOPMENT	15	0.309041769	0.146054479
INDOLE CONTAINING COMPOUND METABOLIC PROCESS	22	0.20971117	0.236712291
NEGATIVE REGULATION OF T CELL PROLIFERATION	44	-0.041967645	0.731490577
REGULATION OF T CELL CYTOKINE PRODUCTION	19	0.067032843	0.72408006
REGULATION OF CHEMOKINE BIOSYNTHETIC PROCESS	11	-0.230253923	0.309034682
POSITIVE REGULATION OF CARTILAGE DEVELOPMENT	24	-0.191585173	0.221231787
MUSCLE ATROPHY	8	-0.135650622	0.624433964
REGULATION OF INSULIN SECRETION INVOLVED IN CELLULAR RESPONSE TO GLUCOSE STIMULUS	41	0.144363952	0.267426794
NEGATIVE REGULATION OF CANONICAL WNT SIGNALING PATHWAY	147	0.036431719	0.593658407
REGULATION OF CELLULAR RESPONSE TO TRANSFORMING GROWTH FACTOR BETA STIMULUS	91	0.178230523	0.0433138
PHOSPHATIDYLINOSITOL DEPHOSPHORYLATION	22	-0.136200769	0.417073375
DIGESTIVE SYSTEM PROCESS	50	0.168445988	0.15660689
CALCIUM ION TRANSPORT	181	0.059135153	0.338908826
ANTIGEN PROCESSING AND PRESENTATION OF EXOGENOUS PEPTIDE ANTIGEN VIA MHC CLASS I	59	-0.293180835	0.002320363

NEGATIVE REGULATION OF CATALYTIC ACTIVITY	694	-0.049447307	0.116061054
PYRIMIDINE NUCLEOSIDE TRIPHOSPHATE METABOLIC PROCESS	19	-0.368477338	0.026777237
CARDIAC MUSCLE TISSUE DEVELOPMENT	123	0.045668776	0.541085547
LEUKOCYTE APOPTOTIC PROCESS	22	0.262968416	0.139510374
NEGATIVE REGULATION OF EMBRYONIC DEVELOPMENT	23	0.098488964	0.57031909
POSITIVE REGULATION OF NUCLEOTIDE METABOLIC PROCESS	98	0.132466103	0.116713243
PEPTIDYL GLUTAMIC ACID MODIFICATION	24	0.05969765	0.723773873
REGULATION OF NOTCH SIGNALING PATHWAY	61	0.180348615	0.092439122
INTERFERON GAMMA MEDIATED SIGNALING PATHWAY	60	-0.020747813	0.843840613
POSITIVE REGULATION OF CYTOKINE SECRETION	74	0.185900674	0.055330279
NEGATIVE REGULATION OF MAST CELL ACTIVATION	8	-0.327000004	0.197692803
MICROTUBULE BUNDLE FORMATION	56	0.160986904	0.1498187
NEGATIVE REGULATION OF CYSTEINE TYPE ENDOPEPTIDASE ACTIVITY	81	-0.162059384	0.062453579
EMBRYONIC SKELETAL JOINT DEVELOPMENT	13	-0.118332433	0.5893453
RESPONSE TO CARBOHYDRATE	147	-0.058566255	0.381128797
ORGANIC ACID TRANSMEMBRANE TRANSPORT	84	-0.080221454	0.360366034
SYSTEM PROCESS	1087	0.04618145	0.075336867
REGULATION OF TUMOR NECROSIS FACTOR BIOSYNTHETIC PROCESS	15	-0.140807286	0.488442197
REGULATION OF PROTEIN MATURATION	66	0.082882602	0.417903381
MONOCARBOXYLIC ACID BIOSYNTHETIC PROCESS	133	0.013461272	0.850550695
COLUMNAR CUBOIDAL EPITHELIAL CELL DEVELOPMENT	42	-0.136224457	0.264105954
POSITIVE REGULATION OF VASCULAR PERMEABILITY	8	-0.170768299	0.531224858
EAR MORPHOGENESIS	90	0.009463713	0.913038062
MOVEMENT OF CELL OR SUBCELLULAR COMPONENT	1088	0.008334917	0.746503309
NEURON CELL CELL ADHESION	13	-0.073077138	0.742622847
TERMINATION OF RNA POLYMERASE II TRANSCRIPTION	61	-0.259277694	0.006952727
CELLULAR RESPONSE TO NITROGEN LEVELS	9	0.352113614	0.181740334
SYNAPTIC SIGNALING	314	0.081305855	0.085630496
MITOTIC CELL CYCLE CHECKPOINT	133	-0.113361672	0.10169368
RESPONSE TO LIPID	754	0.059206342	0.055341394
RESPONSE TO NICOTINE	36	-0.128782795	0.329198984
NEGATIVE REGULATION OF MACROAUTOPHAGY	21	-0.193500615	0.250459581
POSITIVE REGULATION OF GLYCOPROTEIN METABOLIC PROCESS	18	-0.260156554	0.145009754
REGULATION OF MITOPHAGY	39	-0.203956781	0.099783582
FATTY ACID METABOLIC PROCESS	235	0.065935584	0.225529598
ACTIVATION OF IMMUNE RESPONSE	333	-0.020821307	0.644520796
VASCULATURE DEVELOPMENT	424	0.003953523	0.92190745
POSITIVE REGULATION OF PROTEIN METABOLIC PROCESS	1298	-0.026722033	0.258215756
CELL CYCLE G2 M PHASE TRANSITION	137	-0.120952957	0.075955931
NEGATIVE REGULATION OF CYTOKINE SECRETION	33	-0.039677711	0.778726019
VITAMIN TRANSPORT	29	-0.004173238	0.978083714
REGULATION OF CYSTEINE TYPE ENDOPEPTIDASE ACTIVITY INVOLVED IN APOPTOTIC SIGNALING PATHWAY	22	-0.25204253	0.113975008
BLASTODERM SEGMENTATION	13	-0.070618051	0.751274767
NATURAL KILLER CELL ACTIVATION	34	-0.065488838	0.635478046
NEGATIVE REGULATION OF BINDING	122	-0.042011807	0.568349119
POSITIVE REGULATION BY HOST OF VIRAL PROCESS	11	-0.009814805	0.968199897
POSITIVE REGULATION OF RESPONSE TO EXTRACELLULAR STIMULUS	43	-0.047152606	0.702688645
POSITIVE REGULATION OF CALCIUM ION TRANSPORT INTO CYTOSOL	42	-0.296464113	0.010034389
REGULATION OF SMOOTHENED SIGNALING PATHWAY	57	0.168389328	0.129642161
RETINA LAYER FORMATION	19	-0.102489587	0.574648956
EPIDERMIS DEVELOPMENT	195	0.107948979	0.072044617
CELLULAR RESPONSE TO ZINC ION	14	-0.045260487	0.834222758
REGULATION OF GASTRULATION	32	0.318013126	0.0282209
CELLULAR RESPONSE TO IONIZING RADIATION	49	-0.254193939	0.018564116
ACTIN MEDIATED CELL CONTRACTION	59	-0.075023006	0.473606584
POSITIVE REGULATION OF INNATE IMMUNE RESPONSE	219	-0.114625177	0.034065591
POSITIVE REGULATION OF NEUROTRANSMITTER TRANSPORT	12	-0.085497594	0.711226205
NEUTRAL LIPID BIOSYNTHETIC PROCESS	25	-0.017649483	0.913826667
CELLULAR RESPONSE TO NUTRIENT	37	-0.124036165	0.34113045
POSITIVE REGULATION OF GLUCOSE TRANSPORT	35	-0.110053671	0.414458149
ADENYLATE CYCLASE ACTIVATING DOPAMINE RECEPTOR SIGNALING PATHWAY	9	0.315876689	0.258597071
REGULATION OF DIGESTIVE SYSTEM PROCESS	24	0.373543021	0.029341036
REGULATION OF PLASMA MEMBRANE ORGANIZATION	69	0.069308954	0.488177922
RESPONSE TO AMINO ACID	104	-0.093638843	0.23358227

DETECTION OF BIOTIC STIMULUS	14	-0.026431656	0.90322302
RESPONSE TO VITAMIN A	18	0.431742763	0.015674566
POSITIVE REGULATION OF LYMPHOCYTE DIFFERENTIATION	73	-0.000661021	0.994503913
POSITIVE REGULATION OF IMMUNOGLOBULIN SECRETION	8	-0.040115422	0.888749229
SARCOPLASMIC RETICULUM CALCIUM ION TRANSPORT	11	0.011301114	0.963553689
VENTRICULAR SEPTUM DEVELOPMENT	50	-0.10595894	0.348196638
POSITIVE REGULATION OF LONG TERM SYNAPTIC POTENTIATION	11	-0.0449027	0.854002864
INSULIN RECEPTOR SIGNALING PATHWAY	73	-0.03176925	0.738798163
VIRION ASSEMBLY	36	0.428098757	0.002102111
LIPID METABOLIC PROCESS	938	0.022972339	0.406872843
REGULATION OF PROTEIN LOCALIZATION	828	-0.078231827	0.006563584
GLYCEROPHOSPHOLIPID CATABOLIC PROCESS	11	-0.044532628	0.855365233
NEGATIVE REGULATION OF CARBOHYDRATE METABOLIC PROCESS	42	-0.048158395	0.700004446
KERATAN SULFATE BIOSYNTHETIC PROCESS	24	-0.464424844	0.001047792
NEGATIVE REGULATION OF CIRCADIAN RHYTHM	13	0.056778586	0.804412249
POSITIVE REGULATION OF LIPID CATABOLIC PROCESS	21	0.265749611	0.139206326
SIGNAL TRANSDUCTION BY P53 CLASS MEDIATOR	119	-0.071439059	0.334309983
BLOOD VESSEL REMODELING	30	0.21387856	0.160200418
POSITIVE REGULATION OF TYROSINE PHOSPHORYLATION OF STAT3 PROTEIN	32	0.025937481	0.858566376
TRIGLYCERIDE CATABOLIC PROCESS	14	0.491759541	0.011972703
REGULATION OF SKELETAL MUSCLE ADAPTATION	11	0.13792392	0.581792238
GLIAL CELL MIGRATION	36	-0.052427379	0.697245602
MODULATION BY VIRUS OF HOST MORPHOLOGY OR PHYSIOLOGY	35	-0.023252834	0.865909173
PROTEIN K48 LINKED DEUBIQUITINATION	18	-0.593555663	0.000422215
RNA SECONDARY STRUCTURE UNWINDING	41	-0.220658015	0.064913525
ENDOPLASMIC RETICULUM CALCIUM ION HOMEOSTASIS	18	-0.261298548	0.13663138
POSITIVE REGULATION OF RESPONSE TO WOUNDING	131	0.152089434	0.03744062
STEROID METABOLIC PROCESS	189	0.040334419	0.503867987
DIVALENT INORGANIC CATION HOMEOSTASIS	274	0.010088841	0.84001663
REGULATION OF ANION TRANSMEMBRANE TRANSPORT	24	0.242800875	0.153496975
NEGATIVE REGULATION OF GLYCOPROTEIN METABOLIC PROCESS	14	0.223481083	0.316027797
TOXIN TRANSPORT	33	-0.000488887	0.997263434
DETECTION OF LIGHT STIMULUS	38	0.070297561	0.601245822
REGULATION OF HISTONE PHOSPHORYLATION	12	-0.255537348	0.239959835
REGULATION OF PROTEIN IMPORT	164	0.002516391	0.96876383
NEUROLOGICAL SYSTEM PROCESS INVOLVED IN REGULATION OF SYSTEMIC ARTERIAL BLOOD PRESSURE	8	0.139725554	0.63473851
NEGATIVE REGULATION OF RESPONSE TO CYTOKINE STIMULUS	40	-0.004387582	0.972949134
NEGATIVE REGULATION OF DEPHOSPHORYLATION	71	0.070231603	0.47615072
DRUG METABOLIC PROCESS	23	-0.085561526	0.608040046
CELLULAR RESPONSE TO CYTOKINE STIMULUS	504	-0.024663759	0.503534066
FAT CELL DIFFERENTIATION	95	-0.023274315	0.781094452
RESPONSE TO ABIOTIC STIMULUS	875	-0.056066438	0.046606486
AMELOGENESIS	13	-0.227366719	0.28018908
CELLULAR RESPONSE TO INORGANIC SUBSTANCE	134	-0.078895112	0.256851047
RESPONSE TO PROGESTERONE	43	-0.027476218	0.824851358
REGULATION OF PROTON TRANSPORT	12	-0.222320321	0.315455234
REGULATION OF NEURON PROJECTION REGENERATION	18	0.244509081	0.212437711
NEGATIVE REGULATION OF INTERLEUKIN 8 PRODUCTION	12	-0.353747236	0.096975637
POSITIVE REGULATION OF SMAD PROTEIN IMPORT INTO NUCLEUS	9	-0.164318472	0.532123497
GENE SILENCING	191	-0.130738536	0.023327645
NEGATIVE REGULATION OF SMOOTH MUSCLE CONTRACTION	13	0.189444398	0.410502981
LEUKOCYTE ADHESION TO VASCULAR ENDOTHELIAL CELL	13	-0.175170918	0.413583018
ZINC II ION TRANSPORT	22	0.046709786	0.790867615
INTERLEUKIN 1 MEDIATED SIGNALING PATHWAY	13	-0.246691016	0.234308835
POSITIVE REGULATION OF SMOOTH MUSCLE CELL MIGRATION	28	-0.00543793	0.971932034
VENTRICULAR CARDIAC MUSCLE CELL ACTION POTENTIAL	11	0.175402811	0.487102688
RESPONSE TO FATTY ACID	73	0.188333168	0.053320422
DNA METHYLATION INVOLVED IN GAMETE GENERATION	13	0.336244446	0.128826332
NUCLEOSIDE MONOPHOSPHATE BIOSYNTHETIC PROCESS	77	-0.161852391	0.069558744
EPITHELIAL CELL APOPTOTIC PROCESS	23	-0.465009924	0.000959255
DNA SYNTHESIS INVOLVED IN DNA REPAIR	70	-0.058592274	0.544404079
COLUMNAR CUBOIDAL EPITHELIAL CELL DIFFERENTIATION	93	0.073256463	0.395393447
RETROGRADE TRANSPORT ENDOSOME TO PLASMA MEMBRANE	12	0.114339236	0.634779253
TRANSFORMING GROWTH FACTOR BETA RECEPTOR SIGNALING PATHWAY	88	0.039367352	0.655148385
DRUG TRANSPORT	22	0.070860039	0.688713999

POSITIVE REGULATION OF GROWTH	215	-0.027253644	0.625587689
NEGATIVE REGULATION OF CELL PROJECTION ORGANIZATION	131	-0.048880912	0.490903258
TISSUE REMODELING	78	0.028639565	0.759128623
REGULATION OF CYTOPLASMIC TRANSPORT	417	-0.079007036	0.047719958
CD4 POSITIVE OR CD8 POSITIVE ALPHA BETA T CELL LINEAGE COMMITMENT	8	0.187714938	0.532025919
SEGMENT SPECIFICATION	13	0.339382512	0.130081863
CELLULAR RESPONSE TO STEROL	13	0.390280097	0.079711037
T CELL MIGRATION	11	0.18807088	0.451305598
OSSIFICATION	222	-0.010107414	0.854689634
MULTI MULTICELLULAR ORGANISM PROCESS	181	-0.079467818	0.185215361
REGULATION OF ENDOCYTOSIS	170	0.012483139	0.84356863
REGULATION OF EPIDERMAL CELL DIFFERENTIATION	38	0.071020823	0.597698328
MYELOID LEUKOCYTE MEDIATED IMMUNITY	37	-0.387468151	0.00118961
ACTION POTENTIAL	74	-0.070078633	0.454565629
GOLGI TO VACUOLE TRANSPORT	26	-0.225049119	0.138406648
IMMUNOGLOBULIN PRODUCTION INVOLVED IN IMMUNOGLOBULIN MEDIATED IMMUNE RESPONSE	20	-0.151370954	0.390856662
NEGATIVE T CELL SELECTION	10	0.142303825	0.589893196
LIPOPROTEIN LOCALIZATION	11	0.2287665	0.360649214
REGULATION OF CYTOKINE BIOSYNTHETIC PROCESS	76	0.069932993	0.463055711
NEGATIVE REGULATION OF I KAPPAB KINASE NF KAPPAB SIGNALING	44	-0.07398625	0.542090729
DETECTION OF VISIBLE LIGHT	26	-0.016151625	0.919584273
REGULATION OF PROTEIN LOCALIZATION TO CHROMOSOME TELOMERIC REGION	13	-0.189152614	0.375411857
NUCLEAR TRANSCRIBED MRNA CATABOLIC PROCESS DEADENYLATION DEPENDENT DECAY	55	-0.063549446	0.559159896
HISTONE H3 K4 METHYLATION	28	-0.148352944	0.316854976
NEGATIVE REGULATION OF BLOOD PRESSURE	33	0.174003304	0.229966002
ALCOHOL METABOLIC PROCESS	293	0.044371048	0.361906806
NEGATIVE REGULATION OF INTERLEUKIN 12 PRODUCTION	12	0.096310166	0.687776656
CELL SUBSTRATE JUNCTION ASSEMBLY	39	-0.092625894	0.469458561
MONOSACCHARIDE TRANSPORT	39	-0.088288014	0.490929133
REGULATION OF B CELL ACTIVATION	91	-0.064651122	0.445190331
REGULATION OF CELLULAR RESPONSE TO VASCULAR ENDOTHELIAL GROWTH FACTOR STIMULUS	12	0.203019084	0.398346672
REGULATION OF STEROL TRANSPORT	30	0.300880971	0.056099955
POSITIVE REGULATION OF INTRACELLULAR SIGNAL TRANSDUCTION	764	-0.062313618	0.037661452
NEGATIVE REGULATION OF GROWTH	210	0.059856768	0.29768269
NEGATIVE REGULATION OF MACROPHAGE DERIVED FOAM CELL DIFFERENTIATION	11	0.378844008	0.140301733
REGULATION OF SYSTEM PROCESS	417	0.092500607	0.024983196
REGULATION OF CYTOSOLIC CALCIUM ION CONCENTRATION	160	0.039651974	0.544993171
REGULATION OF CARBOHYDRATE BIOSYNTHETIC PROCESS	76	-0.044396131	0.633434848
RESPONSE TO BMP	77	0.039241574	0.676923154
IMIDAZOLE CONTAINING COMPOUND METABOLIC PROCESS	9	-0.060180643	0.822622629
RESPONSE TO PH	30	-0.093234986	0.522119001
SMALL MOLECULE CATABOLIC PROCESS	277	0.045534171	0.362699569
OXALATE TRANSPORT	7	-0.301285059	0.266616024
CELLULAR RESPONSE TO EXOGENOUS DSRNA	12	-0.296041034	0.159817803
RESPONSE TO TEMPERATURE STIMULUS	123	-0.050144805	0.493282197
REGULATION OF TOR SIGNALING	67	0.009256708	0.926508854
DNA REPLICATION CHECKPOINT	13	-0.280467199	0.165593584
RESPONSE TO ACETYLCHOLINE	14	-0.403152759	0.03947991
CELLULAR MODIFIED AMINO ACID METABOLIC PROCESS	179	0.021597214	0.726631282
REGULATION OF ISOTYPE SWITCHING	22	-0.07736402	0.651289906
CELLULAR GLUCOSE HOMEOSTASIS	67	-0.187906845	0.047557297
HEAD DEVELOPMENT	619	-0.017337196	0.604317671
REGULATION OF HEMATOPOIETIC PROGENITOR CELL DIFFERENTIATION	37	0.150530046	0.271232765
REGULATION OF VASCULAR ENDOTHELIAL GROWTH FACTOR PRODUCTION	30	0.06337076	0.675303254
CYCLIC NUCLEOTIDE BIOSYNTHETIC PROCESS	22	0.163361905	0.357086494
REGULATION OF APPETITE	17	0.133532939	0.507244494
PROTEIN DEGLYCOSYLATION	20	-0.270433816	0.107687154
POSITIVE REGULATION OF TRANSFORMING GROWTH FACTOR BETA PRODUCTION	15	-0.161089392	0.42324741
REGULATION OF PHOSPHOLIPASE A2 ACTIVITY	10	0.210508734	0.42176497
RESPONSE TO VITAMIN D	32	-0.042413109	0.767252527

TRANSLATIONAL ELONGATION	103	-0.206445444	0.006489236
REGULATION OF CELL SUBSTRATE ADHESION	161	-0.048515966	0.449014492
CYTOKINE SECRETION	29	-0.148719914	0.310098704
POSITIVE REGULATION OF OXIDOREDUCTASE ACTIVITY	41	-0.039228404	0.757006198
LYMPHOCYTE CHEMOTAXIS	24	0.186211585	0.27021219
MESODERMAL CELL DIFFERENTIATION	23	-0.077815375	0.641777264
METANEPHRIC MESENCHYME DEVELOPMENT	13	0.135526186	0.557819387
BENZENE CONTAINING COMPOUND METABOLIC PROCESS	19	0.249994983	0.184931347
AUDITORY RECEPTOR CELL DEVELOPMENT	15	-0.25345728	0.194397105
REGULATION OF ACROSOME REACTION	12	-0.295411338	0.172367237
NEURONAL STEM CELL POPULATION MAINTENANCE	17	0.110320057	0.585143952
ORGANIC CYCLIC COMPOUND CATABOLIC PROCESS	392	-0.060107439	0.145533924
ACTIN FILAMENT POLYMERIZATION	20	-0.365435459	0.023122065
SMOOTH MUSCLE CONTRACTION	36	0.183634121	0.185841912
MACROPHAGE CHEMOTAXIS	12	0.023416217	0.921405355
NEUTRAL LIPID METABOLIC PROCESS	66	0.112803956	0.271602963
POSITIVE REGULATION OF INTERLEUKIN 4 PRODUCTION	16	0.315135481	0.141401509
DEFENSE RESPONSE TO GRAM POSITIVE BACTERIUM	46	-0.224887092	0.046581172
RESPONSE TO LIPOPROTEIN PARTICLE	19	-0.319544786	0.064427682
AMINO ACID BETAINE METABOLIC PROCESS	17	0.159252271	0.429868974
ALPHA BETA T CELL ACTIVATION	45	-0.03946305	0.744382426
RIBONUCLEOPROTEIN COMPLEX DISASSEMBLY	11	-0.244606947	0.278891227
CELL SUBSTRATE ADHERENS JUNCTION ASSEMBLY	22	-0.154771431	0.355537643
ENTEROENDOCRINE CELL DIFFERENTIATION	16	0.224582729	0.283432653
REGULATION OF JNK CASCADE	142	0.022063047	0.750139222
CELLULAR ALDEHYDE METABOLIC PROCESS	77	0.00520308	0.955653074
RHYTHMIC BEHAVIOR	13	0.19906184	0.389433941
DEVELOPMENTAL PIGMENTATION	34	0.136679864	0.339484636
ARGININE METABOLIC PROCESS	15	-0.175412513	0.37902636
ACIDIC AMINO ACID TRANSPORT	21	0.03606245	0.841148701
NON RECOMBINATIONAL REPAIR	68	0.060094651	0.550174769
CARBOHYDRATE METABOLIC PROCESS	552	-0.03360389	0.340046911
REGULATION OF ACTIN FILAMENT BUNDLE ASSEMBLY	70	-0.09094302	0.341615968
OLFACTORY LOBE DEVELOPMENT	30	0.148973169	0.331914015
RESPONSE TO INTERLEUKIN 6	23	0.151644216	0.387740565
VASCULOGENESIS	58	0.025259435	0.815374653
LIPOPOLYSACCHARIDE MEDIATED SIGNALING PATHWAY	28	-0.015228876	0.921334373
REGULATION OF CELL CELL ADHESION	329	-0.016878258	0.710279106
BRANCHING INVOLVED IN LABYRINTHINE LAYER MORPHOGENESIS	11	0.38870585	0.109678323
REGULATION OF SMOOTH MUSCLE CELL PROLIFERATION	88	-0.039795554	0.646057254
IMMUNE RESPONSE	805	0.029994144	0.314155396
REGULATION OF T CELL MEDIATED IMMUNITY	43	-0.108142282	0.372578108
POSITIVE REGULATION OF CAMP METABOLIC PROCESS	64	0.084546111	0.415823875
MECHANORECEPTOR DIFFERENTIATION	40	-0.064831724	0.610920516
HISTONE UBIQUITINATION	33	-0.112932505	0.415059165
CELLULAR RESPONSE TO ANTIBIOTIC	14	-0.075893625	0.723331668
NEGATIVE REGULATION OF LYASE ACTIVITY	19	-0.169597362	0.340537968
NEURON APOPTOTIC PROCESS	33	-0.24263994	0.068123494
REGULATION OF VASOCONSTRICTION	57	0.096725844	0.379732655
CELLULAR RESPONSE TO FATTY ACID	46	0.198929534	0.104332853
MONOVALENT INORGANIC ANION HOMEOSTASIS	15	-0.157836314	0.436127093
REGULATION OF PH	70	0.105863582	0.287785002
CYCLOOXYGENASE PATHWAY	9	-0.299478068	0.218191557
OXIDOREDUCTION COENZYME METABOLIC PROCESS	94	-0.027945761	0.739685972
CELL SURFACE RECEPTOR SIGNALING PATHWAY INVOLVED IN CELL CELL SIGNALING	51	0.040182827	0.728185673
POSITIVE REGULATION OF RESPONSE TO CYTOKINE STIMULUS	31	-0.241175423	0.073935294
PROTEOGLYCAN BIOSYNTHETIC PROCESS	52	-0.066207705	0.553697834
GRANULOCYTE DIFFERENTIATION	13	0.153390018	0.507161537
GOLGI RIBBON FORMATION	11	-0.322564025	0.138542145
NEGATIVE REGULATION OF ENDOPLASMIC RETICULUM UNFOLDED PROTEIN RESPONSE	11	0.073444087	0.768935881
DEOXYRIBONUCLEOTIDE BIOSYNTHETIC PROCESS	12	-0.038075354	0.870809778
PROTEIN LOCALIZATION TO GOLGI APPARATUS	26	0.071148328	0.661858836
DE NOVO POSTTRANSLATIONAL PROTEIN FOLDING	14	0.11843014	0.595504289
POSITIVE REGULATION OF PROTEIN COMPLEX ASSEMBLY	174	0.041135005	0.513020989

PERK MEDIATED UNFOLDED PROTEIN RESPONSE	12	-0.378444137	0.056577476
RESPONSE TO CORTICOSTERONE	24	-0.138858579	0.388086118
REGULATION OF VIRAL TRANSCRIPTION	61	0.042859333	0.685416497
NEGATIVE REGULATION OF PROTEIN COMPLEX DISASSEMBLY	154	-0.00976766	0.88255414
POSITIVE REGULATION OF CYSTEINE TYPE ENDOPEPTIDASE ACTIVITY INVOLVED IN APOPTOTIC SIGNALING PATHWAY	17	-0.207418844	0.262242313
TUBE FORMATION	121	-0.117676068	0.104527089
CELLULAR RESPONSE TO STEROID HORMONE STIMULUS	193	0.100905576	0.09435797
RIBONUCLEOPROTEIN COMPLEX BIOGENESIS	423	-0.138785336	0.000379492
MEIOTIC CELL CYCLE	155	0.026884653	0.685577769
POSITIVE REGULATION OF RENAL SODIUM EXCRETION	9	0.428287081	0.090020954
RESPONSE TO STIMULUS INVOLVED IN REGULATION OF MUSCLE ADAPTATION	13	0.365095138	0.100973332
CELL FATE DETERMINATION	29	0.398505407	0.009260075
DETECTION OF MECHANICAL STIMULUS INVOLVED IN SENSORY PERCEPTION OF SOUND	10	-0.067427129	0.790984344
REGULATION OF OSTEOBLAST DIFFERENTIATION	106	0.013507152	0.865853399
POSITIVE REGULATION OF TELOMERE MAINTENANCE	43	-0.052765113	0.668954697
POSITIVE REGULATION OF EPIDERMAL GROWTH FACTOR ACTIVATED RECEPTOR ACTIVITY	10	0.115038724	0.665909433
MRNA TRANSCRIPTION	19	0.051612366	0.785670971
COENZYME METABOLIC PROCESS	231	-0.019100673	0.723614074
CELLULAR LIPID METABOLIC PROCESS	753	0.026573219	0.387268877
GDP METABOLIC PROCESS	13	0.247927265	0.281837479
REGULATION OF PROTEIN HOMOOLIGOMERIZATION	16	0.224148857	0.279430243
REGULATION OF FATTY ACID BIOSYNTHETIC PROCESS	31	0.036923612	0.803175557
POTASSIUM ION IMPORT	21	0.257985719	0.159037658
POSTTRANSCRIPTIONAL GENE SILENCING	42	-0.161645873	0.184092103
CELLULAR RESPONSE TO DSRNA	37	-0.208528574	0.097786089
INTERSPECIES INTERACTION BETWEEN ORGANISMS	630	-0.076933639	0.018880118
POSITIVE REGULATION OF INTERFERON GAMMA BIOSYNTHETIC PROCESS	9	0.402223912	0.114162644
LAMELLIPODIUM ORGANIZATION	36	-0.28792581	0.020319511
ADENYLATE CYCLASE MODULATING G PROTEIN COUPLED RECEPTOR SIGNALING PATHWAY	98	0.124032687	0.141483962
PROTEIN TARGETING TO PLASMA MEMBRANE	23	-0.135062531	0.413918284
REGULATION OF DNA DAMAGE CHECKPOINT	13	0.583121901	0.007927429
NEGATIVE REGULATION OF LYMPHOCYTE MEDIATED IMMUNITY	30	-0.073088121	0.618530509
NEGATIVE REGULATION OF LIPID TRANSPORT	22	-0.000197653	0.999096376
REGULATION OF HEART RATE BY CARDIAC CONDUCTION	26	-0.190010507	0.211130561
KETONE BIOSYNTHETIC PROCESS	20	0.028836668	0.875512491
I KAPPAB PHOSPHORYLATION	11	0.082823965	0.740614461
POSITIVE REGULATION OF MRNA 3 END PROCESSING	17	-0.119378889	0.535376929
GROOMING BEHAVIOR	10	0.372148411	0.133566973
REGULATION OF HISTONE H3 K4 METHYLATION	24	-0.209730404	0.178695935
GLUCOSE METABOLIC PROCESS	104	-0.013145478	0.869897739
POSITIVE REGULATION OF BLOOD PRESSURE	31	0.206528767	0.165413182
REGULATION OF ANOIKIS	23	0.110805328	0.52370716
POSITIVE REGULATION OF CARDIAC MUSCLE CELL PROLIFERATION	17	0.326752518	0.095870659
NEGATIVE REGULATION OF VIRAL ENTRY INTO HOST CELL	16	0.206452315	0.322516488
NEGATIVE REGULATION OF B CELL PROLIFERATION	14	-0.249425091	0.231549039
PURINE NUCLEOBASE METABOLIC PROCESS	19	0.04191642	0.824755195
MITOCHONDRIAL CALCIUM ION TRANSPORT	11	0.007878552	0.974569239
NUCLEOBASE CONTAINING SMALL MOLECULE INTERCONVERSION	20	-0.403939471	0.013054486
MITOCHONDRIAL FUSION	17	0.18766682	0.358303099
REGULATION OF DEPHOSPHORYLATION	141	0.048006196	0.49199558
CARBOHYDRATE HOMEOSTASIS	145	-0.063323014	0.346167652
NEURON PROJECTION REGENERATION	31	-0.086438583	0.548486075
CARTILAGE DEVELOPMENT INVOLVED IN ENDOCHONDRAL BONE MORPHOGENESIS	15	-0.00042055	0.998411976
NEGATIVE REGULATION OF EPIDERMAL CELL DIFFERENTIATION	12	0.259874847	0.279832875
LONG TERM SYNAPTIC POTENTIATION	32	-0.012658378	0.930122971
MONOCARBOXYLIC ACID TRANSPORT	101	0.027330416	0.739267798
REGULATION OF CYTOSKELETON ORGANIZATION	453	0.026167688	0.504686144
SEX DIFFERENTIATION	210	0.055872164	0.330676223
NEGATIVE REGULATION OF HORMONE SECRETION	59	0.024733181	0.81759748
CELL CYCLE	1212	-0.077103712	0.001416212
CELLULAR RESPONSE TO PH	13	0.036001161	0.874817655

DSRNA FRAGMENTATION	22	-0.295500772	0.058437608
NEGATIVE REGULATION OF CATECHOLAMINE SECRETION	12	0.200217064	0.402590739
REGULATION OF INTRINSIC APOPTOTIC SIGNALING PATHWAY IN RESPONSE TO DNA DAMAGE	32	0.038776308	0.790377285
RESPONSE TO VITAMIN E	14	0.209803188	0.351258921
POSITIVE REGULATION OF PROTEIN KINASE B SIGNALING	74	-0.164791306	0.069019522
LYMPH VESSEL MORPHOGENESIS	12	0.175246839	0.467608856
POSITIVE REGULATION OF CALCIUM ION IMPORT	45	-0.208096998	0.069558941
NUCLEAR IMPORT	119	-0.099810787	0.174441299
DETECTION OF ABIOTIC STIMULUS	78	0.129603368	0.170696912
OLIGOSACCHARIDE BIOSYNTHETIC PROCESS	11	-0.440714227	0.041994213
CARDIAC MUSCLE CELL ACTION POTENTIAL	32	-0.237286292	0.080171066
NEGATIVE REGULATION OF CELL CYCLE G2 M PHASE TRANSITION	24	-0.003329113	0.984094513
PURINE NUCLEOSIDE CATABOLIC PROCESS	13	-0.080924926	0.715857557
NEGATIVE REGULATION OF CYTOSKELETON ORGANIZATION	201	-0.01313066	0.820660509
RESPONSE TO MUSCLE ACTIVITY	18	0.084211274	0.667158992
NEGATIVE REGULATION OF JNK CASCADE	31	0.096293509	0.51935646
REGULATION OF MYOTUBE DIFFERENTIATION	48	0.100991406	0.400358186
REGULATION OF CATENIN IMPORT INTO NUCLEUS	22	-0.16070137	0.335167388
FUSION OF SPERM TO EGG PLASMA MEMBRANE	5	-0.027748943	0.93917026
TRACHEA MORPHOGENESIS	11	0.288865316	0.252192576
POSITIVE REGULATION OF RESPONSE TO BIOTIC STIMULUS	29	-0.108768483	0.460620867
ENDOTHELIAL CELL PROLIFERATION	22	-0.101789275	0.549396589
LIPID PHOSPHORYLATION	88	0.210512822	0.018441464
CELLULAR CARBOHYDRATE METABOLIC PROCESS	127	-0.086690413	0.224510026
NUCLEOSIDE PHOSPHATE CATABOLIC PROCESS	64	0.147654267	0.158387133
CELLULAR RESPONSE TO STARVATION	112	-0.023607448	0.759680946
VESICLE MEDIATED TRANSPORT BETWEEN ENDOSOMAL COMPARTMENTS	20	0.257341754	0.168156031
RESPONSE TO DRUG	384	0.008558125	0.839889086
LIPID BIOSYNTHETIC PROCESS	452	-0.036877679	0.341272537
AMMONIUM TRANSPORT	44	-0.109848764	0.360433974
PHOTORECEPTOR CELL DIFFERENTIATION	39	0.037969823	0.773792279
ORGANONITROGEN COMPOUND METABOLIC PROCESS	1559	-0.098200488	5.02E-06
MEGAKARYOCYTE DIFFERENTIATION	18	-0.363836791	0.02929239
PROTEIN DEPOLYMERIZATION	20	-0.073916034	0.681052476
CELLULAR RESPONSE TO CALCIUM ION	42	-0.101921746	0.407997474
DNA METHYLATION OR DEMETHYLATION	49	0.131957082	0.268279359
NEGATIVE REGULATION OF EXTRINSIC APOPTOTIC SIGNALING PATHWAY VIA DEATH DOMAIN RECEPTORS	31	-0.293007396	0.027805613
POSITIVE REGULATION OF CANONICAL WNT SIGNALING PATHWAY	107	0.028429683	0.721699722
GLOMERULAR EPITHELIUM DEVELOPMENT	16	-0.357960266	0.043674424
MITOTIC RECOMBINATION	41	-0.188540806	0.119356629
STRAND DISPLACEMENT	25	-0.043069165	0.790418007
PURINERGIC NUCLEOTIDE RECEPTOR SIGNALING PATHWAY	16	0.0030924	0.987949885
REGULATION OF NEURON PROJECTION DEVELOPMENT	364	0.031027295	0.47739383
MICROTUBULE ORGANIZING CENTER ORGANIZATION	78	-0.179626022	0.041099497
PIRNA METABOLIC PROCESS	11	0.249030329	0.313807799
NEGATIVE REGULATION OF GLUCOSE TRANSPORT	17	0.003599499	0.985544495
VACUOLE ORGANIZATION	153	0.052822967	0.431531022
CARDIOCYTE DIFFERENTIATION	85	0.102201009	0.258727902
RESPONSE TO GROWTH FACTOR	421	-0.059143116	0.138296809
POSITIVE REGULATION OF WOUND HEALING	44	0.192865954	0.124868956
REGULATION OF LEUKOCYTE MEDIATED IMMUNITY	133	-0.057793235	0.41071675
NEGATIVE REGULATION OF CELLULAR SENESCENCE	11	0.321314023	0.202439362
POSITIVE REGULATION OF ACTIVATED T CELL PROLIFERATION	23	-0.048231705	0.774936154
RETROGRADE VESICLE MEDIATED TRANSPORT GOLGI TO ER	71	-0.047021899	0.625238875
NEGATIVE REGULATION OF CELL CYCLE PHASE TRANSITION	140	-0.118385645	0.079226574
DIENCEPHALON DEVELOPMENT	61	-0.040579204	0.696230024
POSITIVE REGULATION OF MYOBLAST DIFFERENTIATION	19	0.026548156	0.888182116
MITOCHONDRION ORGANIZATION	549	-0.08579365	0.013878615
HAIR CELL DIFFERENTIATION	27	-0.126681324	0.404892968
REGULATION OF STEM CELL PROLIFERATION	76	-0.045055948	0.628308628
REGULATION OF CHROMATIN ORGANIZATION	140	-0.047661555	0.487790185
MEMBRANE PROTEIN ECTODOMAIN PROTEOLYSIS	20	-0.527341462	0.001297156
REGULATION OF MITOCHONDRIAL MEMBRANE POTENTIAL	48	0.009562182	0.935686272
PERICARDIUM DEVELOPMENT	16	0.007217533	0.971904854

CELL FATE COMMITMENT	179	0.171083549	0.006607857
PEROXISOMAL TRANSPORT	17	-0.075653091	0.697756517
POSITIVE REGULATION OF ADHERENS JUNCTION ORGANIZATION	20	-0.253919544	0.127382965
POSITIVE REGULATION OF PEPTIDYL TYROSINE PHOSPHORYLATION	142	0.05066779	0.467001205
REGULATION OF MYOBLAST FUSION	18	-0.1035013	0.580883962
NEGATIVE REGULATION OF HYDROLASE ACTIVITY	327	0.053661974	0.245296848
REGULATION OF NEUROTRANSMITTER UPTAKE	12	0.135201115	0.573856237
ACTIVIN RECEPTOR SIGNALING PATHWAY	19	0.212561514	0.268731887
POSITIVE REGULATION OF PROTEIN ACETYLATION	34	0.063526172	0.654673011
NEGATIVE REGULATION OF CALCIUM ION TRANSPORT	40	0.00760959	0.953222181
MULTI ORGANISM REPRODUCTIVE PROCESS	678	0.068082107	0.036411911
RENAL SYSTEM VASCULATURE DEVELOPMENT	16	-0.031591492	0.876373723
REGULATION OF PLASMA LIPOPROTEIN PARTICLE LEVELS	36	0.351737131	0.010214076
DETECTION OF STIMULUS	216	0.140990622	0.013816761
ESTABLISHMENT OF PROTEIN LOCALIZATION TO VACUOLE	31	-0.037056867	0.799498281
FUCOSE METABOLIC PROCESS	12	-0.168923647	0.450743363
REGULATION OF AMINO ACID TRANSPORT	24	0.250259545	0.134821645
APOPTOTIC DNA FRAGMENTATION	14	-0.040055754	0.853330311
HETEROPHILIC CELL CELL ADHESION VIA PLASMA MEMBRANE CELL ADHESION MOLECULES	36	-0.212562009	0.096137535
NEGATIVE REGULATION OF LIPID STORAGE	16	0.196996126	0.349175131
REGULATION OF MICROVILLUS ORGANIZATION	13	-0.123418776	0.573498034
SCF DEPENDENT PROTEASOMAL UBIQUITIN DEPENDENT PROTEIN CATABOLIC PROCESS	23	-0.024073083	0.887225554
REGULATION OF LEUKOCYTE CHEMOTAXIS	87	-0.101745036	0.235071423
ENTERIC NERVOUS SYSTEM DEVELOPMENT	9	0.218259682	0.431926714
MUSCLE CELL FATE COMMITMENT	12	0.355007698	0.124537932
PERIPHERAL NERVOUS SYSTEM AXON ENSHEATHMENT	21	-0.007373916	0.967019353
IRON ION IMPORT	11	-0.192277904	0.41121485
REGULATION OF METANEPHROS DEVELOPMENT	21	-0.003994157	0.982145557
NUCLEAR TRANSCRIBED MRNA POLY A TAIL SHORTENING	24	0.064810203	0.701558084
CELLULAR RESPONSE TO AMINO ACID STIMULUS	52	-0.10797495	0.328371641
DNA GEOMETRIC CHANGE	76	0.078052744	0.413609946
REGULATION OF GUANYLATE CYCLASE ACTIVITY	7	0.20140911	0.517610715
REGULATION OF MONOCYTE DIFFERENTIATION	13	-0.022104787	0.922027865
NUCLEOTIDE EXCISION REPAIR DNA INCISION	38	0.208163286	0.126451936
CERAMIDE METABOLIC PROCESS	62	-0.066137932	0.518382889
REGULATION OF LAMELLIPODIUM ORGANIZATION	36	0.185292958	0.185726918
REGULATION OF B CELL PROLIFERATION	50	-0.072275403	0.525512077
REGULATION OF CELL COMMUNICATION BY ELECTRICAL COUPLING	12	-0.223017996	0.309288523
SPINAL CORD ASSOCIATION NEURON DIFFERENTIATION	7	-0.283549968	0.321790003
CYTOKINE PRODUCTION INVOLVED IN IMMUNE RESPONSE	15	-0.198683331	0.312581966
DEOXYRIBOSE PHOSPHATE CATABOLIC PROCESS	20	0.06746082	0.715620471
HEART VALVE DEVELOPMENT	30	0.24570515	0.107264713
GLYCOSYL COMPOUND METABOLIC PROCESS	332	-0.107420569	0.015076941
REGULATION OF TUMOR NECROSIS FACTOR MEDIATED SIGNALING PATHWAY	45	0.010045612	0.934581808
PEPTIDYL TYROSINE MODIFICATION	157	0.026723397	0.685480708
LEUKOCYTE CELL CELL ADHESION	212	-0.049050135	0.380683161
NEGATIVE REGULATION OF LEUKOCYTE DEGRANULATION	10	-0.187445785	0.442175856
NUCLEAR PORE COMPLEX ASSEMBLY	11	-0.416735956	0.047268939
PROTEIN LOCALIZATION TO ORGANELLE	514	-0.088038976	0.01447523
PHOSPHATIDYLETHANOLAMINE BIOSYNTHETIC PROCESS	12	-0.478963586	0.012498049
ENDOCYTIC RECYCLING	24	0.226081887	0.185567832
CELLULAR RESPONSE TO RADIATION	121	-0.178535128	0.011643033
POSITIVE REGULATION OF TRANSCRIPTION REGULATORY REGION DNA BINDING	14	0.123302024	0.580499725
REGULATION OF INTERLEUKIN 17 PRODUCTION	18	0.029880337	0.877651066
POSITIVE REGULATION OF DENDRITE MORPHOGENESIS	29	-0.245674673	0.084045603
SUPEROXIDE ANION GENERATION	7	0.286271244	0.355765079
CELLULAR RESPONSE TO KETONE	65	0.161145997	0.121588143
POSITIVE REGULATION OF INTERLEUKIN 17 PRODUCTION	11	0.050220544	0.840164522
NEGATIVE REGULATION OF APOPTOTIC SIGNALING PATHWAY	186	-0.113639126	0.052518088
MULTICELLULAR ORGANISMAL HOMEOSTASIS	215	0.06635014	0.24335176
NEGATIVE REGULATION OF CELL CYCLE G1 S PHASE TRANSITION	93	-0.15017329	0.06720721
REGULATION OF MITOCHONDRIAL OUTER MEMBRANE PERMEABILIZATION INVOLVED IN APOPTOTIC SIGNALING PATHWAY	42	-0.105176064	0.392043071

REGULATION OF SIGNAL TRANSDUCTION BY P53 CLASS MEDIATOR	157	-0.029788435	0.647720635
URONIC ACID METABOLIC PROCESS	13	0.272101247	0.229954829
REGULATION OF DELAYED RECTIFIER POTASSIUM CHANNEL ACTIVITY	15	-0.307616236	0.101456706
POSITIVE REGULATION OF CYTOKINE PRODUCTION	311	0.014313904	0.760746123
RESPONSE TO YEAST	8	-0.274155038	0.293251639
GAMETE GENERATION	444	0.109527156	0.006333254
KIDNEY MESENCHYME DEVELOPMENT	17	0.107196066	0.595475507
REPLICATIVE SENESCENCE	12	-0.060906792	0.793552074
REGULATION OF CELLULAR AMIDE METABOLIC PROCESS	329	-0.009992044	0.82619085
DENDRITIC SPINE ORGANIZATION	12	-0.269002516	0.218420639
COCHLEA DEVELOPMENT	34	-0.128177592	0.34421162
HISTONE H2A ACETYLATION	14	-0.174991727	0.401061132
DNA REPLICATION DEPENDENT NUCLEOSOME ORGANIZATION	31	0.051756649	0.727410362
CELLULAR COMPONENT MORPHOGENESIS	783	0.044744065	0.1393956
CELL AGING	61	-0.173236848	0.083047135
NEGATIVE REGULATION OF MEGAKARYOCYTE DIFFERENTIATION	17	0.276550204	0.160783545
ACTIVATION OF MAPKKK ACTIVITY	11	-0.009060045	0.970649223
RRNA 3 END PROCESSING	13	-0.20318405	0.33629362
GLUTATHIONE METABOLIC PROCESS	43	-0.011277589	0.927889068
POSITIVE REGULATION OF PROTEIN MODIFICATION PROCESS	977	-0.022163272	0.410780873
POSITIVE REGULATION OF MICROTUBULE POLYMERIZATION	17	0.194952152	0.33899854
PYRIMIDINE CONTAINING COMPOUND TRANSMEMBRANE TRANSPORT	12	-0.31781668	0.140003293
BRANCHED CHAIN AMINO ACID METABOLIC PROCESS	22	-0.225652729	0.160858295
ENERGY HOMEOSTASIS	13	0.171610055	0.45839745
NEGATIVE REGULATION OF T CELL MEDIATED IMMUNITY	12	0.017734725	0.940378556
DEPHOSPHORYLATION	261	-0.081995228	0.10171782
REGULATION OF CELL PROLIFERATION INVOLVED IN HEART MORPHOGENESIS	15	0.002213394	0.991646977
MATERNAL PROCESS INVOLVED IN FEMALE PREGNANCY	51	-0.148125834	0.179429152
UBIQUINONE METABOLIC PROCESS	11	0.096919072	0.69848298
POSITIVE REGULATION OF CELL CELL ADHESION	207	-0.066742009	0.236303711
EARLY ENDOSOME TO GOLGI TRANSPORT	12	0.079949696	0.738365248
POSITIVE REGULATION OF CATION TRANSMEMBRANE TRANSPORT	78	-0.259540368	0.00255169
MORPHOGENESIS OF A BRANCHING STRUCTURE	149	-0.010740947	0.872996685
INTRACELLULAR RECEPTOR SIGNALING PATHWAY	151	0.037711006	0.575911943
BEHAVIORAL RESPONSE TO COCAINE	8	0.375337271	0.180626802
HISTONE MRNA METABOLIC PROCESS	28	0.006656524	0.965735537
REGULATION OF ESTABLISHMENT OF PROTEIN LOCALIZATION TO PLASMA MEMBRANE	46	0.160072497	0.194378174
PYRIMIDINE DEOXYRIBONUCLEOTIDE METABOLIC PROCESS	16	0.065862281	0.750220821
RESPONSE TO PARATHYROID HORMONE	13	0.340462906	0.11950562
HINDBRAIN MORPHOGENESIS	33	-0.111312885	0.421342268
POSITIVE REGULATION OF PRODUCTION OF MOLECULAR MEDIATOR OF IMMUNE RESPONSE	53	0.005559616	0.960654119
HIGH DENSITY LIPOPROTEIN PARTICLE REMODELING	12	0.455997133	0.034545526
POSITIVE REGULATION OF PROTEIN DEPOLYMERIZATION	18	0.037054023	0.84877771
MUSCLE HYPERTROPHY	21	0.007364942	0.967164489
HUMORAL IMMUNE RESPONSE MEDIATED BY CIRCULATING IMMUNOGLOBULIN	28	0.201596604	0.200375031
NEGATIVE REGULATION OF PROTEIN LOCALIZATION TO CELL PERIPHERY	19	-0.030134422	0.871614068
REGULATION OF PHOSPHOLIPID BIOSYNTHETIC PROCESS	12	0.12084941	0.61568444
MULTICELLULAR ORGANISMAL SIGNALING	96	0.119130755	0.162563467
NEGATIVE REGULATION OF DEVELOPMENTAL PROCESS	691	0.032346415	0.312873996
REGULATION OF HOMOTYPIC CELL CELL ADHESION	263	-0.02369625	0.639732107
ORGANIC HYDROXY COMPOUND BIOSYNTHETIC PROCESS	142	-0.028995534	0.672159105
CDP DIACYLGLYCEROL METABOLIC PROCESS	14	-0.0804138	0.707792005
FUCOSYLATION	17	-0.100219856	0.603765542
REGULATION OF TYPE I INTERFERON MEDIATED SIGNALING PATHWAY	30	-0.17213927	0.228140882
RHYTHMIC PROCESS	258	0.00636079	0.901582562
NEUROMUSCULAR PROCESS CONTROLLING POSTURE	13	0.188205424	0.416851964
MYOTUBE DIFFERENTIATION	47	0.009137357	0.93917219
STEROID HORMONE MEDIATED SIGNALING PATHWAY	112	0.135438964	0.087716007
REGULATION OF RESPONSE TO BIOTIC STIMULUS	203	-0.125684301	0.024589594
POSITIVE REGULATION OF CELL SIZE	13	-0.012477802	0.956036962
DETECTION OF LIGHT STIMULUS INVOLVED IN SENSORY PERCEPTION	11	0.234905838	0.343563242
PROTEIN LOCALIZATION TO CELL SURFACE	18	-0.151722297	0.411079045
REGULATION OF INTERLEUKIN 8 SECRETION	14	-0.107668713	0.611543578

REGULATION OF SUPEROXIDE METABOLIC PROCESS	21	-0.031183312	0.860393542
PEPTIDYL PROLINE MODIFICATION	49	0.008003158	0.945581345
POSITIVE REGULATION OF NEURON MIGRATION	11	0.406765226	0.092805703
REGULATION OF LONG TERM NEURONAL SYNAPTIC PLASTICITY	21	0.205331959	0.259255487
PHOSPHOLIPID TRANSPORT	47	0.462784554	7.74E-05
ACROSOME REACTION	10	0.121592215	0.642959042
SOMATIC STEM CELL DIVISION	21	0.233629296	0.195566631
HISTONE MRNA CATABOLIC PROCESS	14	0.201289348	0.370848599
ACETYL COA BIOSYNTHETIC PROCESS	11	-0.427261744	0.035150224
RESPONSE TO ARSENIC CONTAINING SUBSTANCE	29	-0.310963218	0.024225953
REGULATION OF DNA BIOSYNTHETIC PROCESS	90	-0.058829972	0.490310962
FIBROBLAST GROWTH FACTOR RECEPTOR SIGNALING PATHWAY	78	-0.043919978	0.632821817
CARDIOLIPIN METABOLIC PROCESS	12	-0.060760669	0.793885353
REGULATION OF CELL CYCLE ARREST	101	-0.082820696	0.300694062
REGULATION OF MRNA SPLICING VIA SPLICEOSOME	58	0.003925014	0.970931092
REGULATION OF ANATOMICAL STRUCTURE MORPHOGENESIS	919	0.006458294	0.816805441
FEMALE GAMETE GENERATION	76	0.044363893	0.640004015
PLASMA MEMBRANE FUSION	18	-0.025963767	0.892315076
EXTRACELLULAR MATRIX DISASSEMBLY	55	-0.045342697	0.678320521
PROTEIN HOMOOLIGOMERIZATION	210	-0.020443949	0.7179043
REGULATION OF OXIDATIVE STRESS INDUCED CELL DEATH	41	0.096142335	0.459858666
TRANSCRIPTION FROM RNA POLYMERASE I PROMOTER	34	0.139176377	0.329887567
REGULATION OF PEPTIDYL THREONINE PHOSPHORYLATION	35	-0.040877048	0.765566601
NONMOTILE PRIMARY CILIUM ASSEMBLY	21	0.030455366	0.865432996
HEART TRABECULA FORMATION	13	0.158140106	0.498156029
BODY FLUID SECRETION	64	-0.095228731	0.341877604
TOR SIGNALING	16	0.037899762	0.854143432
MEMBRANE HYPERPOLARIZATION	10	0.25814428	0.317253821
CELLULAR WATER HOMEOSTASIS	7	0.131124128	0.675914278
POSITIVE REGULATION OF CHROMOSOME ORGANIZATION	140	-0.070923423	0.298964351
ADENYLATE CYCLASE INHIBITING G PROTEIN COUPLED RECEPTOR SIGNALING PATHWAY	46	0.16792393	0.171251033
RESPONSE TO VITAMIN	89	0.14906317	0.092431699
REGULATION OF ATPASE ACTIVITY	57	-0.064722995	0.544757516
NEGATIVE REGULATION OF STRESS ACTIVATED PROTEIN KINASE SIGNALING CASCADE	39	-0.088642717	0.489534655
STRIATED MUSCLE CELL PROLIFERATION	15	0.068851848	0.747713494
MUSCLE FIBER DEVELOPMENT	41	0.105533138	0.416599813
THALAMUS DEVELOPMENT	13	0.129740426	0.573691982
STEROL CATABOLIC PROCESS	9	-0.03040799	0.910595128
POSITIVE REGULATION OF CYTOKINE BIOSYNTHETIC PROCESS	49	0.127747153	0.284916823
NEGATIVE REGULATION OF CHEMOKINE PRODUCTION	14	0.011530501	0.958059017
DETERMINATION OF ADULT LIFESPAN	13	-0.364667081	0.071563738
REGULATION OF PROTEIN KINASE C SIGNALING	12	0.145694069	0.543226629
REGULATION OF CYSTEINE TYPE ENDOPEPTIDASE ACTIVITY	195	-0.100244198	0.081375992
REGULATION OF CELLULAR RESPONSE TO STRESS	647	-0.025255444	0.439955059
POSITIVE REGULATION OF EPITHELIAL CELL MIGRATION	95	0.080955723	0.343102922
MISMATCH REPAIR	26	-0.002959763	0.985283845
MACROPHAGE DIFFERENTIATION	17	0.241488822	0.238529043
PHARYNGEAL SYSTEM DEVELOPMENT	16	0.141407789	0.497396202
REGULATION OF ESTABLISHMENT OR MAINTENANCE OF CELL POLARITY	22	0.155513649	0.380403821
MYELIN ASSEMBLY	17	-0.061288911	0.754018024
HOMOTYPIC CELL CELL ADHESION	47	-0.066488111	0.571645371
NEGATIVE REGULATION OF EPITHELIAL CELL DIFFERENTIATION	33	0.110130212	0.447767997
ERROR PRONE TRANSLESION SYNTHESIS	19	0.239919658	0.216147221
MAST CELL MEDIATED IMMUNITY	16	-0.263463972	0.162390181
POSITIVE REGULATION OF PROTEIN DEACETYLATION	18	-0.286525147	0.097916968
NEGATIVE REGULATION OF MITOTIC NUCLEAR DIVISION	33	-0.280985434	0.028868055
HOMEOSTASIS OF NUMBER OF CELLS WITHIN A TISSUE	28	0.128880971	0.414716819
POSITIVE REGULATION OF IMMUNE EFFECTOR PROCESS	130	-0.097043468	0.167159497
UROGENITAL SYSTEM DEVELOPMENT	262	0.057359044	0.265598897
NEGATIVE REGULATION OF CELL ACTIVATION	134	-0.09557372	0.167724855
CELLULAR RESPONSE TO ALCOHOL	106	0.067307564	0.404433139
NEGATIVE REGULATION OF MONOOXYGENASE ACTIVITY	10	0.423325823	0.083491816
PEPTIDYL TYROSINE AUTOPHOSPHORYLATION	32	-0.268263414	0.04646097
CELL JUNCTION ASSEMBLY	117	-0.170581673	0.018625101

LATERAL VENTRICLE DEVELOPMENT	10	0.209795043	0.421937313
MACROMOLECULE CATABOLIC PROCESS	831	-0.063258776	0.028192313
INTERACTION WITH SYMBIONT	50	0.107173139	0.362954543
REGULATION OF POLYSACCHARIDE METABOLIC PROCESS	37	-0.035133402	0.792509713
SMOOTHENED SIGNALING PATHWAY	61	0.08160457	0.442816198
CELL MIGRATION IN HINDBRAIN	13	-0.143787342	0.510258239
EYE DEVELOPMENT	274	0.002411149	0.961460347
TRIPARTITE REGIONAL SUBDIVISION	10	-0.166131163	0.498458807
ORGANIC CATION TRANSPORT	15	0.21611346	0.312882206
SLEEP	11	-0.102258924	0.670000153
DEVELOPMENTAL PROCESS INVOLVED IN REPRODUCTION	489	0.107792676	0.004876051
URATE METABOLIC PROCESS	9	-0.317332965	0.189411942
PROTEIN DEPHOSPHORYLATION	180	-0.098583988	0.099807567
REGULATION OF CELLULAR PROTEIN CATABOLIC PROCESS	260	-0.090580671	0.070558917
REGULATION OF PROTEIN AUTOPHOSPHORYLATION	33	0.17364697	0.232552269
REGULATION OF NEURON DEATH	228	-0.007578341	0.889410088
TETRAHYDROFOLATE INTERCONVERSION	9	-0.173193949	0.501149416
RESPONSE TO GAMMA RADIATION	48	0.020621503	0.862152768
REGULATION OF CYCLIN DEPENDENT PROTEIN KINASE ACTIVITY	88	-0.23903406	0.003347655
REGULATION OF CELL DEATH	1291	-0.025848339	0.275350143
REGULATION OF RHODOPSIN MEDIATED SIGNALING PATHWAY	19	-0.153410508	0.389800827
NEGATIVE REGULATION OF STRESS FIBER ASSEMBLY	14	-0.223393653	0.268319837
NEGATIVE REGULATION OF NF KAPPAB IMPORT INTO NUCLEUS	16	-0.004415093	0.98276847
POSITIVE REGULATION OF PROTEIN IMPORT	92	0.033576136	0.696606043
MULTI ORGANISM METABOLIC PROCESS	137	-0.22424816	0.000678546
REGULATION OF KERATINOCYTE DIFFERENTIATION	24	0.232931797	0.171505796
MONOVALENT INORGANIC CATION HOMEOSTASIS	98	0.128708356	0.127386929
REGULATION OF BMP SIGNALING PATHWAY	67	0.051484276	0.610757441
HEART PROCESS	73	-0.083446423	0.374413252
MUSCLE SYSTEM PROCESS	222	-0.001431177	0.979350558
MESENCHYME DEVELOPMENT	167	0.162040972	0.012561415
TRANSCRIPTION FROM RNA POLYMERASE III PROMOTER	40	-0.011235971	0.930695558
GLIAL CELL FATE COMMITMENT	7	0.067821897	0.828647616
POSITIVE REGULATION OF PROTEOLYSIS	330	-0.080287406	0.072429372
REGULATION OF CILIUM ASSEMBLY	46	0.053626604	0.660298964
ACTIVATION OF PROTEIN KINASE B ACTIVITY	20	-0.090748323	0.611477363
REGULATION OF TRANSLATIONAL INITIATION IN RESPONSE TO STRESS	13	-0.131365638	0.547819132
ANTERIOR POSTERIOR AXIS SPECIFICATION	41	-0.263321486	0.024913775
REGULATION OF LONG TERM SYNAPTIC POTENTIATION	16	-0.131657685	0.503309579
REGULATION OF PROTEIN KINASE B SIGNALING	111	-0.114269044	0.130264129
HOMOLOGOUS CHROMOSOME SEGREGATION	37	0.296417195	0.028678468
MUSCLE CELL CELLULAR HOMEOSTASIS	15	-0.370787176	0.045150335
T CELL LINEAGE COMMITMENT	10	0.364339745	0.179694826
NUCLEOSIDE BISPHOSPHATE BIOSYNTHETIC PROCESS	15	0.036282489	0.864617525
RESPONSE TO LITHIUM ION	22	-0.035290706	0.838419996
PROTEIN FOLDING IN ENDOPLASMIC RETICULUM	10	-0.695691738	0.000105208
REGULATION OF FATTY ACID OXIDATION	27	0.236359567	0.136928534
MORPHOGENESIS OF A POLARIZED EPITHELIUM	26	0.021163806	0.895606041
REGULATION OF DNA TEMPLATED TRANSCRIPTION IN RESPONSE TO STRESS	63	0.077682843	0.458439999
ENDODERM FORMATION	47	-0.289693944	0.008708822
POSITIVE REGULATION OF SPROUTING ANGIOGENESIS	14	0.229133832	0.302627721
REGULATION OF DEVELOPMENT HETEROCHRONIC	10	0.240198892	0.360896634
ESTABLISHMENT OF ENDOTHELIAL BARRIER	29	-0.23343962	0.101521335
REGULATION OF HUMORAL IMMUNE RESPONSE	38	0.14371665	0.28711196
REGULATION OF MAP KINASE ACTIVITY	289	-0.054317499	0.257737301
NEGATIVE REGULATION OF INTERLEUKIN 1 BETA PRODUCTION	7	-0.40438513	0.13909062
FEMALE MEIOTIC DIVISION	22	0.260962397	0.14726524
REGULATION OF GLUTAMATE SECRETION	13	0.23693156	0.296800833
NLS BEARING PROTEIN IMPORT INTO NUCLEUS	22	-0.059783974	0.728213381
VACUOLE FUSION	22	0.247721705	0.162804766
SEMAPHORIN PLEXIN SIGNALING PATHWAY INVOLVED IN NEURON PROJECTION GUIDANCE	13	0.361593481	0.102923814
POSITIVE REGULATION OF BONE REMODELING	12	0.326747074	0.163798078
HISTONE H3 K4 TRIMETHYLATION	12	-0.369559738	0.071751575
PRE MIRNA PROCESSING	13	-0.376780943	0.055825718
CELLULAR RESPONSE TO ESTROGEN STIMULUS	36	0.187590325	0.178553165

LYTIC VACUOLE ORGANIZATION	44	0.072321442	0.56353184
REGULATION OF CHEMOTAXIS	164	0.005748087	0.928773383
NEGATIVE REGULATION OF RESPONSE TO STIMULUS	1197	-0.001693109	0.945201692
MEIOTIC CELL CYCLE PROCESS	128	0.014529199	0.841879705
REGULATION OF PLATELET ACTIVATION	28	-0.089173837	0.555298224
NEGATIVE REGULATION OF ACTIN FILAMENT POLYMERIZATION	37	0.028942725	0.830751283
DEVELOPMENTAL CELL GROWTH	69	0.087706079	0.381736849
REGULATION OF BONE RESORPTION	28	-0.006261089	0.967679797
SPINDLE ASSEMBLY	64	0.00572325	0.95551381
REGULATION OF SODIUM ION TRANSMEMBRANE TRANSPORTER ACTIVITY	34	-0.075163129	0.585741497
NEGATIVE REGULATION OF ANTIGEN RECEPTOR MEDIATED SIGNALING PATHWAY	20	0.006834975	0.970256156
CONVERGENT EXTENSION	12	0.233732221	0.32703872
SPHINGOLIPID BIOSYNTHETIC PROCESS	69	-0.048183441	0.621365098
REGULATION OF PHOSPHOPROTEIN PHOSPHATASE ACTIVITY	54	-0.07234488	0.509337602
REGULATION OF MULTI ORGANISM PROCESS	412	-0.00303468	0.940762129
REGULATION OF TRANSLATIONAL FIDELITY	15	-0.265371079	0.175638264
POSITIVE REGULATION OF CELL CYCLE ARREST	79	-0.100582812	0.263528082
CONNECTIVE TISSUE DEVELOPMENT	167	0.124722195	0.054507423
CYTOPLASMIC PATTERN RECOGNITION RECEPTOR SIGNALING PATHWAY	31	-0.115121629	0.421126261
PROTEIN LOCALIZATION	1621	-0.034963438	0.101981968
REGULATION OF STEROID BIOSYNTHETIC PROCESS	40	0.165965753	0.208140041
NUCLEAR ENVELOPE ORGANIZATION	80	-0.299549619	0.000446039
ASYMMETRIC PROTEIN LOCALIZATION	17	0.229116421	0.262450982
REGULATION OF GLIAL CELL DIFFERENTIATION	52	0.107254421	0.354449347
BIOMINERAL TISSUE DEVELOPMENT	55	-0.039214749	0.720196479
CELLULAR RESPONSE TO ENDOGENOUS STIMULUS	891	0.011020769	0.69706353
SCHWANN CELL DEVELOPMENT	25	-0.036749159	0.820914294
POSITIVE REGULATION OF B CELL DIFFERENTIATION	13	0.109957702	0.633737776
POSITIVE REGULATION OF HISTONE METHYLATION	32	-0.21692812	0.110290396
NEGATIVE REGULATION OF T CELL DIFFERENTIATION	28	-0.035033167	0.819394049
RNA POLYADENYLATION	28	0.020809677	0.893486324
NEUROTRANSMITTER METABOLIC PROCESS	17	0.504974855	0.006490305
ORGANELLE INHERITANCE	11	-0.321978266	0.144447729
AUTONOMIC NERVOUS SYSTEM DEVELOPMENT	35	-0.039203343	0.774999052
ASPARTATE FAMILY AMINO ACID METABOLIC PROCESS	49	0.038928323	0.741350116
EXPLORATION BEHAVIOR	17	0.157926219	0.435226462
POSITIVE REGULATION OF MUSCLE TISSUE DEVELOPMENT	52	0.198519582	0.087272446
MRNA METABOLIC PROCESS	577	-0.136214528	5.19E-05
RESPONSE TO THYROID HORMONE	20	-0.218990452	0.194682028
PROTEIN TRANSMEMBRANE TRANSPORT	48	0.154695274	0.199787678
INTRINSIC APOPTOTIC SIGNALING PATHWAY BY P53 CLASS MEDIATOR	50	0.030536156	0.793373776
RESPONSE TO RETINOIC ACID	94	0.018116526	0.831133213
MAMMARY GLAND LOBULE DEVELOPMENT	15	-0.249985671	0.201170443
PARAXIAL MESODERM DEVELOPMENT	16	0.31778996	0.114833585
ODONTOGENESIS OF DENTIN CONTAINING TOOTH	61	0.0438166	0.678909781
DIACYLGLYCEROL METABOLIC PROCESS	11	-0.119583832	0.616270782
COCHLEA MORPHOGENESIS	19	-0.180682648	0.309575416
NEGATIVE REGULATION OF LIPID CATABOLIC PROCESS	18	0.257933884	0.183013838
REGULATION OF TRANSLATIONAL INITIATION	78	-0.124693167	0.165901632
MOTILE CILIUM ASSEMBLY	12	-0.104597132	0.649108875
POSITIVE REGULATION OF FATTY ACID OXIDATION	13	0.38826745	0.075280637
NEGATIVE REGULATION OF ALPHA BETA T CELL ACTIVATION	20	0.09984876	0.590676076
MYELOID CELL ACTIVATION INVOLVED IN IMMUNE RESPONSE	35	-0.076284852	0.57412069
POSITIVE REGULATION OF SYNAPTIC TRANSMISSION	88	-0.013328922	0.878511346
CELL RECOGNITION	92	-0.026805616	0.752547603
REGULATION OF RETROGRADE PROTEIN TRANSPORT ER TO CYTOSOL	12	-0.586852729	0.001417178
PROTEIN K63 LINKED UBIQUITINATION	34	0.047938411	0.735154186
NEGATIVE REGULATION OF PHOSPHORYLATION	381	-0.044177479	0.293340795
NEGATIVE REGULATION OF NUCLEAR DIVISION	42	-0.226271014	0.053308526
NEGATIVE REGULATION OF ENDOPLASMIC RETICULUM STRESS INDUCED INTRINSIC APOPTOTIC SIGNALING PATHWAY	19	-0.078167095	0.670964432
REGULATION OF GENERATION OF PRECURSOR METABOLITES AND ENERGY	79	0.021320316	0.818065778
MESENCHYMAL CELL PROLIFERATION	11	-0.199010843	0.388395315
LIPID CATABOLIC PROCESS	195	0.091167029	0.127669406
REVERSE CHOLESTEROL TRANSPORT	12	0.52162585	0.011404265

POSITIVE REGULATION OF LIPID METABOLIC PROCESS	110	0.07404341	0.350610957
REGULATION OF CHOLESTEROL STORAGE	12	0.05053842	0.832438286
PROTEIN LOCALIZATION TO SYNAPSE	12	-0.058604351	0.80121926
PROSTATE GLAND GROWTH	11	-0.340524997	0.109184268
NEGATIVE REGULATION OF CARDIAC MUSCLE CELL APOPTOTIC PROCESS	11	-0.073575548	0.761182071
REGULATION OF INSULIN LIKE GROWTH FACTOR RECEPTOR SIGNALING PATHWAY	19	-0.008567749	0.963539967
RESPIRATORY SYSTEM DEVELOPMENT	175	0.081777559	0.195126673
ION HOMEOSTASIS	466	0.066552493	0.08754313
EPITHELIAL TO MESENCHYMAL TRANSITION INVOLVED IN ENDOCARDIAL CUSHION FORMATION	11	0.275155616	0.263477539
NEGATIVE REGULATION OF STAT CASCADE	37	0.010075317	0.940478894
REGULATION OF SYNAPTIC TRANSMISSION GABAERGIC	19	0.008429065	0.964262375
NUCLEOSIDE TRIPHOSPHATE CATABOLIC PROCESS	11	0.395001532	0.108130486
NEUROEPITHELIAL CELL DIFFERENTIATION	52	0.034284769	0.764412739
NUCLEOSIDE MONOPHOSPHATE METABOLIC PROCESS	224	-0.122653885	0.021415074
REGULATION OF GLUCOSE TRANSPORT	90	-0.199930242	0.014726802
MATURE B CELL DIFFERENTIATION INVOLVED IN IMMUNE RESPONSE	12	0.059993447	0.801711019
CATECHOLAMINE TRANSPORT	9	-0.114458203	0.665245311
NEGATIVE REGULATION OF MYELOID CELL APOPTOTIC PROCESS	13	0.076790135	0.738191483
NEGATIVE REGULATION OF TRANSFORMING GROWTH FACTOR BETA RECEPTOR SIGNALING PATHWAY	59	0.107986057	0.320636747
ADHERENS JUNCTION ORGANIZATION	66	-0.06474756	0.514531316
EPITHELIAL CELL MORPHOGENESIS	40	0.125536774	0.342824768
POSITIVE REGULATION OF OSTEOBLAST DIFFERENTIATION	57	-0.060531128	0.571585048
OSTEOBLAST DEVELOPMENT	16	0.063103887	0.760299542
MYD88 INDEPENDENT TOLL LIKE RECEPTOR SIGNALING PATHWAY	29	0.295478969	0.054134673
NEGATIVE REGULATION OF SIGNAL TRANSDUCTION BY P53 CLASS MEDIATOR	27	-0.010902055	0.944705887
POSITIVE REGULATION OF DNA RECOMBINATION	16	-0.186617129	0.336691001
REGULATION OF PEPTIDYL SERINE PHOSPHORYLATION OF STAT PROTEIN	9	0.325761378	0.225609174
HISTONE H4 K16 ACETYLATION	19	0.294780325	0.128874138
POSITIVE REGULATION OF NATURAL KILLER CELL ACTIVATION	17	0.004473432	0.982038624
POSITIVE REGULATION OF MESENCHYMAL CELL PROLIFERATION	23	-0.216294756	0.175721943
CHRONIC INFLAMMATORY RESPONSE	13	-0.344651155	0.077564251
POSITIVE REGULATION OF LIPASE ACTIVITY	50	-0.125700754	0.262561656
POSITIVE REGULATION OF ERK1 AND ERK2 CASCADE	138	0.055952588	0.428391442
MEMBRANE ORGANIZATION	806	-0.025968507	0.378108827
CAMP METABOLIC PROCESS	28	0.115459493	0.462644634
MITOCHONDRIAL ATP SYNTHESIS COUPLED PROTON TRANSPORT	17	-0.219779101	0.233340993
CELLULAR RESPONSE TO OXYGEN RADICAL	12	-0.30984026	0.134472057
REGULATION OF CILIUM MOVEMENT	8	-0.149901889	0.586017152
PURINE NUCLEOSIDE MONOPHOSPHATE BIOSYNTHETIC PROCESS	57	-0.147079443	0.157323124
POSITIVE REGULATION OF PEPTIDE SECRETION	75	-0.016964311	0.857283429
RESPONSE TO DEXAMETHASONE	29	-0.030770659	0.838478314
STEROL TRANSPORT	39	0.412755202	0.002112886
ORGANONITROGEN COMPOUND BIOSYNTHETIC PROCESS	906	-0.15056437	2.92E-08
SOMATIC DIVERSIFICATION OF IMMUNE RECEPTORS VIA SOMATIC MUTATION	13	-0.44982696	0.022747724
REGULATION OF CHROMOSOME ORGANIZATION	259	-0.060744987	0.229570127
EMBRYONIC EYE MORPHOGENESIS	28	-0.002181859	0.98874449
BLASTOCYST GROWTH	16	-0.173262446	0.376508463
ESTROGEN BIOSYNTHETIC PROCESS	9	0.499487244	0.041332519
REGULATION OF CELL MATRIX ADHESION	84	-0.018127127	0.838856296
VISUAL BEHAVIOR	42	0.015646467	0.901806523
LIPOSACCHARIDE METABOLIC PROCESS	99	-0.068116541	0.40079373
REGULATION OF CELL ADHESION	558	-0.038856941	0.26692256
INTRACELLULAR LIPID TRANSPORT	19	-0.173996266	0.332000714
POSITIVE REGULATION OF INTERFERON ALPHA PRODUCTION	15	-0.19344061	0.330893311
NEGATIVE REGULATION OF FATTY ACID BIOSYNTHETIC PROCESS	13	0.131583006	0.568221042
NEGATIVE REGULATION OF TELOMERE MAINTENANCE	25	-0.310791145	0.038839637
POSITIVE REGULATION OF TRANSCRIPTION FROM RNA POLYMERASE II PROMOTER	888	0.017319685	0.541801778
REGULATION OF ACTIN FILAMENT DEPOLYMERIZATION	42	0.014006134	0.912023604
ENDOCRINE SYSTEM DEVELOPMENT	99	0.003618276	0.965038623
REGULATION OF MYELOID LEUKOCYTE DIFFERENTIATION	94	-0.064308477	0.440206509
HYDROGEN PEROXIDE METABOLIC PROCESS	19	-0.142877725	0.426808887
RESPONSE TO DSRNA	60	-0.109289586	0.287094636

REGULATION OF INTRINSIC APOPTOTIC SIGNALING PATHWAY BY P53 CLASS MEDIATOR	22	0.162559798	0.361190221
GLYCEROLIPID CATABOLIC PROCESS	28	0.197530442	0.213155714
POSITIVE REGULATION OF ACTIN FILAMENT POLYMERIZATION	60	0.060335927	0.572769355
AMINE CATABOLIC PROCESS	18	0.0443729	0.819568862
REGULATION OF STEROID METABOLIC PROCESS	61	0.205161284	0.054336842
MATURE B CELL DIFFERENTIATION	16	0.147941063	0.478647083
NEGATIVE REGULATION OF MUSCLE TISSUE DEVELOPMENT	31	0.037590185	0.799744609
C21 STEROID HORMONE BIOSYNTHETIC PROCESS	10	0.001389925	0.995715645
EPITHELIAL CELL DIFFERENTIATION INVOLVED IN KIDNEY DEVELOPMENT	19	-0.299202105	0.074471283
STEROL BIOSYNTHETIC PROCESS	38	-0.133158557	0.297958707
DE NOVO PROTEIN FOLDING	19	0.022440088	0.905321077
REGULATION OF PHAGOCYTOSIS	55	0.106759981	0.341632381
DNA INTEGRITY CHECKPOINT	137	-0.100102757	0.144127378
MEMBRANE PROTEIN PROTEOLYSIS	30	-0.401291888	0.002615049
POSITIVE REGULATION OF OSTEOBLAST PROLIFERATION	10	0.352196943	0.166279968
TRNA THREONYLCARBAMOYLADENOSINE METABOLIC PROCESS	10	-0.281975049	0.221118569
TUBE MORPHOGENESIS	288	-0.032280678	0.504153667
ARP2 3 COMPLEX MEDIATED ACTIN NUCLEATION	16	0.226102942	0.278259101
REGULATION OF HYDROGEN PEROXIDE METABOLIC PROCESS	13	-0.139894584	0.521559386
REGULATION OF EMBRYONIC DEVELOPMENT	106	0.019275336	0.809717355
SENSORY ORGAN DEVELOPMENT	406	0.002873142	0.944375427
RETINA VASCULATURE DEVELOPMENT IN CAMERA TYPE EYE	13	-0.234230431	0.262527072
POSITIVE REGULATION OF MAP KINASE ACTIVITY	183	-0.028312639	0.639553386
BONE MORPHOGENESIS	63	-0.077772332	0.442214208
CELL PROJECTION ORGANIZATION	781	0.021783504	0.470479732
REGULATION OF MEMBRANE INVAGINATION	11	0.411435802	0.074066275
ISOPRENOID BIOSYNTHETIC PROCESS	23	0.160825649	0.354675308
NEGATIVE REGULATION OF PROTEIN METABOLIC PROCESS	952	-0.010496003	0.700993838
NEGATIVE REGULATION OF NUCLEOTIDE CATABOLIC PROCESS	13	0.358432189	0.104048959
RETROGRADE TRANSPORT ENDOSOME TO GOLGI	68	0.048704243	0.627457706
MITOCHONDRIAL GENOME MAINTENANCE	23	-0.20009822	0.216767143
COENZYME CATABOLIC PROCESS	10	0.514920676	0.034933319
POSITIVE REGULATION OF MYELOID LEUKOCYTE CYTOKINE PRODUCTION INVOLVED IN IMMUNE RESPONSE	11	0.345729892	0.151930732
NEGATIVE REGULATION OF NEURAL PRECURSOR CELL PROLIFERATION	19	0.042388556	0.822867827
GLUCAN BIOSYNTHETIC PROCESS	23	0.240527953	0.166924784
LINOLEIC ACID METABOLIC PROCESS	10	0.533212995	0.015567044
NEGATIVE REGULATION OF COFACTOR METABOLIC PROCESS	10	0.192897671	0.460269704
MRNA CIS SPLICING VIA SPLICEOSOME	15	-0.479672551	0.007097249
VIRAL LATENCY	11	-0.233586977	0.307988103
CYTOPLASMIC SEQUESTERING OF PROTEIN	38	0.024728504	0.85311485
REGULATION OF SPINDLE ASSEMBLY	14	0.268214826	0.223882353
NEGATIVE REGULATION OF STEROL TRANSPORT	9	0.119632561	0.669468696
REGULATION OF INFLAMMATORY RESPONSE	236	0.111055569	0.041867104
OLIGODENDROCYTE DEVELOPMENT	28	-0.029023078	0.850243316
RESPONSE TO INTERFERON GAMMA	114	0.104536628	0.181646724
CARDIAC EPITHELIAL TO MESENCHYMAL TRANSITION	23	0.231653622	0.181637851
PROTEIN LOCALIZATION TO MITOCHONDRION	65	0.068040609	0.508416205
NEGATIVE REGULATION OF MULTICELLULAR ORGANISMAL METABOLIC PROCESS	10	-0.502365695	0.02042618
NEGATIVE REGULATION OF ENDOTHELIAL CELL PROLIFERATION	28	-0.126687858	0.396270723
POSITIVE REGULATION OF BLOOD CIRCULATION	76	0.133501359	0.162844331
MICROTUBULE BASED MOVEMENT	174	-0.042700715	0.489416605
SENSORY ORGAN MORPHOGENESIS	198	-0.004730617	0.93550571
PROTEIN DNA COMPLEX SUBUNIT ORGANIZATION	203	0.006448656	0.911290954
RESPONSE TO HYDROPEROXIDE	14	-0.001237351	0.995485179
PROTEIN COMPLEX SUBUNIT ORGANIZATION	1350	-0.048199636	0.037174442
REGULATION OF ACTIN FILAMENT BASED MOVEMENT	30	-0.147118541	0.304496394
CHROMATIN SILENCING	79	-0.016386507	0.858542998
MAINTENANCE OF PROTEIN LOCATION IN NUCLEUS	13	-0.111353757	0.614107469
CELLULAR RESPONSE TO ESTRADIOL STIMULUS	27	0.217535278	0.178102681
LOCOMOTORY BEHAVIOR	144	0.040114243	0.56111435
RESPONSE TO EPINEPHRINE	14	0.34191265	0.106947666
REGULATION OF MACROPHAGE DIFFERENTIATION	17	0.036324626	0.855857827
REGULATION OF PROTEIN LOCALIZATION TO NUCLEUS	198	-0.012171314	0.83481068

NUCLEUS ORGANIZATION	124	-0.189674516	0.007039449
TRABECULA FORMATION	22	0.115616267	0.515961761
SUBSTRATE DEPENDENT CELL MIGRATION	26	0.019421847	0.904102783
SYNAPTIC TRANSMISSION GLUTAMATERGIC	18	-0.076502477	0.686112132
REGULATION OF SEQUENCE SPECIFIC DNA BINDING TRANSCRIPTION FACTOR ACTIVITY	326	0.01613172	0.725551883
RETINA HOMEOSTASIS	46	0.013656258	0.910243118
POSITIVE REGULATION OF HUMORAL IMMUNE RESPONSE	11	0.078534405	0.753140668
MITOCHONDRION MORPHOGENESIS	17	0.078813163	0.695116723
COPPER ION HOMEOSTASIS	14	0.079496327	0.720258192
STEM CELL DIFFERENTIATION	174	0.116835858	0.065732592
DOPAMINERGIC NEURON DIFFERENTIATION	25	-0.184320463	0.237640501
MAGNESIUM ION TRANSMEMBRANE TRANSPORT	16	0.177098955	0.398557883
ENDOPLASMIC RETICULUM ORGANIZATION	35	0.179041903	0.206168976
REGULATION OF CHOLESTEROL METABOLIC PROCESS	20	0.255761068	0.165239017
SCHWANN CELL DIFFERENTIATION	30	-0.020998988	0.887766033
DIVALENT INORGANIC ANION HOMEOSTASIS	9	0.094539214	0.733064285
PROTEIN COMPLEX LOCALIZATION	45	0.143744486	0.251449329
NEGATIVE REGULATION OF GTPASE ACTIVITY	39	0.03625695	0.78369276
PROTEIN HOMOTRIMERIZATION	16	0.103424165	0.618651597
RESPONSE TO MINERALOCORTICOID	33	0.058332706	0.685961888
POSITIVE REGULATION OF INTRINSIC APOPTOTIC SIGNALING PATHWAY	47	0.085853029	0.479471672
REGULATION OF TYROSINE PHOSPHORYLATION OF STAT5 PROTEIN	17	-0.002878962	0.988421708
NEGATIVE REGULATION OF LEUKOCYTE CHEMOTAXIS	10	0.208968797	0.425030644
NEGATIVE REGULATION OF MULTI ORGANISM PROCESS	127	0.019678163	0.78803093
COENZYME A BIOSYNTHETIC PROCESS	10	0.049771434	0.848846793
NEGATIVE REGULATION OF DIGESTIVE SYSTEM PROCESS	12	0.316708559	0.185196524
NUCLEOSIDE TRANSPORT	16	-0.10158206	0.610455518
NEURON DIFFERENTIATION	751	0.060109736	0.052188162
FLAVONOID METABOLIC PROCESS	16	0.350721954	0.076271182
PROTEASOMAL PROTEIN CATABOLIC PROCESS	257	-0.069780205	0.168328454
UDP N ACETYLGLUCOSAMINE METABOLIC PROCESS	13	-0.015258622	0.946218268
FERTILIZATION	100	-0.004286679	0.958307892
POSITIVE REGULATION OF INTERLEUKIN 2 PRODUCTION	31	0.141909338	0.345689866
GOLGI LOCALIZATION	12	-0.256607625	0.237542135
G2 DNA DAMAGE CHECKPOINT	31	0.206998876	0.167809614
ACTIVATION OF INNATE IMMUNE RESPONSE	183	-0.123885113	0.03545807
REGULATION OF CELL PROJECTION ASSEMBLY	137	0.050292075	0.477983985
REGULATION OF HEART GROWTH	38	0.120703703	0.371677763
IONOTROPIC GLUTAMATE RECEPTOR SIGNALING PATHWAY	19	-0.138601533	0.441648062
NEURON FATE COMMITMENT	49	0.311465047	0.008795243
REGULATION OF CELLULAR RESPIRATION	20	0.155746059	0.405014329
REGULATION OF TELOMERE CAPPING	20	0.264608755	0.159801561
TROPHECTODERMAL CELL DIFFERENTIATION	14	-0.070997662	0.741504689
SPERMATID DIFFERENTIATION	90	0.055452544	0.525676311
GOLGI VESICLE TRANSPORT	295	-0.043207499	0.3644906
RESPONSE TO X RAY	26	-0.370575508	0.009456594
NUCLEOPHAGY	18	0.014587937	0.939909343
APOPTOTIC PROCESS INVOLVED IN MORPHOGENESIS	15	0.390714142	0.054793753
GLIAL CELL DIFFERENTIATION	118	0.153985293	0.046348581
PHOSPHATIDYLSERINE METABOLIC PROCESS	23	0.099000165	0.568047515
NEURAL TUBE FORMATION	92	-0.178865456	0.028320895
REGULATION OF CELL CYCLE PHASE TRANSITION	305	-0.100357154	0.029829978
PROTEIN K63 LINKED DEUBIQUITINATION	22	-0.22798905	0.166467563
ATP HYDROLYSIS COUPLED TRANSMEMBRANE TRANSPORT	32	0.056572696	0.698914319
SEMAPHORIN PLEXIN SIGNALING PATHWAY	35	0.142180604	0.316644878
REGULATION OF REACTIVE OXYGEN SPECIES BIOSYNTHETIC PROCESS	59	0.131442121	0.226011146
REGULATION OF NEUROTRANSMITTER RECEPTOR ACTIVITY	25	0.042139863	0.798561662
POSITIVE REGULATION OF FATTY ACID METABOLIC PROCESS	27	0.269375586	0.091311287
CEREBELLAR CORTEX FORMATION	18	-0.227224458	0.205591599
REGULATION OF PEPTIDE SECRETION	178	-0.046463891	0.446326507
PYRIMIDINE NUCLEOSIDE CATABOLIC PROCESS	17	0.118308059	0.557213785
NEGATIVE REGULATION OF ORGANIC ACID TRANSPORT	17	0.224311167	0.264645271
KETONE BODY METABOLIC PROCESS	7	0.089594029	0.775238637
POSITIVE REGULATION OF INTERLEUKIN 13 PRODUCTION	11	0.310477523	0.235859529
REGULATION OF TYROSINE PHOSPHORYLATION OF STAT3 PROTEIN	39	-0.048491327	0.708529202

HEART GROWTH	22	0.071788973	0.684908998
LYMPHOCYTE MIGRATION	32	0.168245473	0.250819351
LOW DENSITY LIPOPROTEIN PARTICLE REMODELING	8	0.651679074	0.003068689
POSITIVE REGULATION OF PROTEIN SUMOYLATION	10	-0.087489798	0.729141522
ADULT HEART DEVELOPMENT	10	-0.179634174	0.464782902
NEGATIVE REGULATION OF PROTEIN ACETYLATION	17	-0.169782303	0.366547075
POSITIVE REGULATION OF DEVELOPMENTAL PROCESS	1001	0.023537763	0.381115455
RESPONSE TO BIOTIC STIMULUS	692	-0.014414116	0.64976178
OUTFLOW TRACT MORPHOGENESIS	50	0.094885564	0.420031147
NUCLEAR EXPORT	141	-0.195376693	0.00308131
MEMBRANE INVAGINATION	26	0.051021042	0.753193976
NEGATIVE REGULATION OF ERAD PATHWAY	12	-0.608157829	0.000843248
AROMATIC AMINO ACID FAMILY CATABOLIC PROCESS	18	0.288912359	0.130377138
RESPONSE TO TRANSITION METAL NANOPARTICLE	125	-0.071088604	0.324620942
REGULATION OF ACTIN CYTOSKELETON REORGANIZATION	29	-0.109587873	0.458185631
HYDROGEN TRANSPORT	106	-0.167130241	0.027870313
REGULATION OF OXIDATIVE STRESS INDUCED NEURON DEATH	11	0.110029167	0.660738778
ER TO GOLGI VESICLE MEDIATED TRANSPORT	159	-0.10126814	0.111370265
RESPONSE TO TOXIC SUBSTANCE	213	-0.071258639	0.198936151
REGULATION OF PROTEIN MODIFICATION PROCESS	1483	-0.010179186	0.648482325
CELLULAR RESPONSE TO INTERLEUKIN 6	19	0.1344575	0.486198882
POSITIVE REGULATION OF TRANSCRIPTION FROM RNA POLYMERASE I PROMOTER	14	-0.04170321	0.847367041
CELL MORPHOGENESIS INVOLVED IN NEURON DIFFERENTIATION	319	0.107172785	0.022974332
STRIATUM DEVELOPMENT	15	-0.11649127	0.56894509
NEUTROPHIL MEDIATED IMMUNITY	18	-0.526091129	0.001590764
NEGATIVE REGULATION OF CHROMATIN MODIFICATION	42	0.094169382	0.4631973
CELLULAR CHEMICAL HOMEOSTASIS	461	0.014941447	0.70024249
CELLULAR RESPONSE TO LEPTIN STIMULUS	16	0.059354282	0.77397305
EPITHELIAL CELL PROLIFERATION	80	-0.035480566	0.696494866
NEGATIVE REGULATION OF PEPTIDYL SERINE PHOSPHORYLATION	23	0.227084498	0.193498703
REGULATION OF LIPOPROTEIN METABOLIC PROCESS	11	0.256025819	0.301943051
POSITIVE REGULATION OF NUCLEAR DIVISION	55	-0.050185865	0.645817825
REGULATION OF COFACTOR METABOLIC PROCESS	45	-0.101892368	0.391338845
MYOTUBE CELL DEVELOPMENT	25	-0.021345722	0.895769476
PHOSPHATIDYLGLYCEROL ACYL CHAIN REMODELING	12	0.289122262	0.226663359
FIBRINOLYSIS	17	0.103198244	0.60808593
MONOCARBOXYLIC ACID CATABOLIC PROCESS	79	0.158597944	0.090845185
THIOESTER BIOSYNTHETIC PROCESS	43	-0.127674833	0.290155756
KERATINIZATION	23	0.378191032	0.022246525
POSITIVE REGULATION OF CELL DEVELOPMENT	415	0.017921424	0.660988612
MONOSACCHARIDE METABOLIC PROCESS	169	-0.0248697	0.692688476
REGULATION OF CELL FATE SPECIFICATION	10	0.197752363	0.452352633
CELLULAR CARBOHYDRATE BIOSYNTHETIC PROCESS	47	-0.014552199	0.902771954
TRANSMEMBRANE RECEPTOR PROTEIN TYROSINE KINASE SIGNALING PATHWAY	440	-0.015590181	0.69270924
REGULATION OF INTERLEUKIN 6 BIOSYNTHETIC PROCESS	12	0.089749343	0.708220226
NEGATIVE REGULATION OF MUSCLE HYPERTROPHY	11	0.337849562	0.17781015
POSITIVE REGULATION OF CATENIN IMPORT INTO NUCLEUS	9	0.143727315	0.60506623
RECOMBINATIONAL REPAIR	72	-0.011005561	0.909108544
RECIPROCAL DNA RECOMBINATION	35	-0.035620523	0.795310581
POSITIVE REGULATION OF CELL KILLING	33	0.036263313	0.800594696
POSITIVE REGULATION OF NUCLEASE ACTIVITY	15	-0.07709184	0.710167458
REGULATION OF MICROTUBULE POLYMERIZATION OR DEPOLYMERIZATION	162	0.024489283	0.706105042
NEGATIVE REGULATION OF OSSIFICATION	62	0.045406377	0.66540194
REGULATION OF SKELETAL MUSCLE CONTRACTION	7	-0.518268045	0.039890391
CELLULAR RESPONSE TO PEPTIDE	239	0.004267378	0.936288473
REGULATION OF DNA REPLICATION	148	-0.008088543	0.904555177
C TERMINAL PROTEIN AMINO ACID MODIFICATION	11	0.083057997	0.739753195
REGULATION OF ORGANELLE ASSEMBLY	134	0.014284161	0.840981415
NUCLEAR TRANSCRIBED MRNA CATABOLIC PROCESS NONSENSE MEDIATED DECAY	117	-0.17484412	0.015637763
NEGATIVE REGULATION OF CELL ADHESION	198	-0.047723162	0.409658156
NEGATIVE REGULATION OF ALPHA BETA T CELL DIFFERENTIATION	13	-0.04635673	0.836314642
CELLULAR TRANSITION METAL ION HOMEOSTASIS	63	0.065226214	0.532711962
CELL CELL RECOGNITION	36	0.035258806	0.797561981

NEGATIVE REGULATION OF DNA METABOLIC PROCESS	99	-0.074443374	0.358243369
SKELETAL MUSCLE CELL DIFFERENTIATION	45	0.105578995	0.395070626
RNA 3 END PROCESSING	95	-0.224564632	0.004307812
RESPONSE TO ANTIBIOTIC	43	-0.084158738	0.49129153
REGULATION OF SYSTEMIC ARTERIAL BLOOD PRESSURE BY RENIN ANGIOTENSIN	16	0.149598749	0.471229162
POSITIVE REGULATION OF LIPID BIOSYNTHETIC PROCESS	54	0.144799743	0.20325364
CELL ADHESION MEDIATED BY INTEGRIN	12	-0.316506713	0.141694426
CELL FATE SPECIFICATION	53	0.015304829	0.892188017
CELLULAR RESPONSE TO GLUCOSE STARVATION	31	-0.076654982	0.595193546
REGULATION OF ENDOPLASMIC RETICULUM UNFOLDED PROTEIN RESPONSE	28	-0.044951323	0.769029498
WOUND HEALING SPREADING OF EPIDERMAL CELLS	8	0.106859136	0.719512492
POSITIVE REGULATION OF TRANSPORT	790	-0.020991356	0.480772009
MONOCYTE CHEMOTAXIS	27	-0.108866218	0.476119619
CEREBRAL CORTEX CELL MIGRATION	42	-0.029716389	0.812902311
POSITIVE REGULATION OF WNT SIGNALING PATHWAY	137	-0.02030639	0.771400152
FOLIC ACID CONTAINING COMPOUND METABOLIC PROCESS	25	0.020807687	0.899303539
MYELOID CELL DIFFERENTIATION	169	-0.07445689	0.231246598
REGULATION OF CELLULAR COMPONENT MOVEMENT	696	0.002569389	0.935528329
TELENCEPHALON REGIONALIZATION	8	-0.188841633	0.485932182
CELLULAR RESPONSE TO OSMOTIC STRESS	19	-0.20009196	0.260481223
REGULATION OF INTERFERON GAMMA SECRETION	8	0.433558543	0.11161087
REGULATION OF RESPONSE TO INTERFERON GAMMA	22	-0.348343492	0.029327371
REGULATION OF DENDRITIC SPINE MORPHOGENESIS	26	-0.300932251	0.039996201
CELLULAR COPPER ION HOMEOSTASIS	11	0.031242007	0.899890555
RESPONSE TO ACIDIC PH	15	-0.124606533	0.540377449
REGULATION OF RENAL SYSTEM PROCESS	30	0.025679426	0.864312133
NEGATIVE REGULATION OF IMMUNE SYSTEM PROCESS	323	-0.045654587	0.316115823
PERIPHERAL NERVOUS SYSTEM DEVELOPMENT	59	-0.010465683	0.921689523
CARDIAC CHAMBER FORMATION	10	0.047305921	0.8561579
PLACENTA DEVELOPMENT	128	0.202728533	0.006437185
POSITIVE REGULATION OF JUN KINASE ACTIVITY	57	-0.075234086	0.480159526
POSITIVE REGULATION OF ALPHA BETA T CELL DIFFERENTIATION	32	0.070377657	0.631247266
LENS MORPHOGENESIS IN CAMERA TYPE EYE	16	-0.251866618	0.17680909
ERAD PATHWAY	71	-0.181860272	0.049971618
MYELIN MAINTENANCE	12	0.039216771	0.869137587
CELL CELL JUNCTION ASSEMBLY	64	-0.210370806	0.028838683
LIPID STORAGE	26	0.102519559	0.529528124
MEMBRANE LIPID METABOLIC PROCESS	162	-0.055685775	0.382400223
REGULATION OF EXTENT OF CELL GROWTH	99	0.135156764	0.108517841
ACTIN FILAMENT ORGANIZATION	146	-0.051598586	0.442666351
REGULATION OF INTERLEUKIN 8 BIOSYNTHETIC PROCESS	9	0.391350574	0.130641432
CELL VOLUME HOMEOSTASIS	19	-0.160563725	0.369475089
REGULATION OF RELEASE OF SEQUESTERED CALCIUM ION INTO CYTOSOL	63	-0.266852874	0.004879552
GMP METABOLIC PROCESS	20	-0.03090411	0.864953137
RENAL ABSORPTION	9	-0.19473471	0.450917322
REGULATION OF ENDOTHELIAL CELL CHEMOTAXIS	15	0.264160329	0.217473267
POSITIVE REGULATION OF MITOCHONDRIAL OUTER MEMBRANE PERMEABILIZATION INVOLVED IN APOPTOTIC SIGNALING PATHWAY	35	-0.046679597	0.733112884
EPITHELIAL STRUCTURE MAINTENANCE	19	0.168796627	0.381954229
PEPTIDYL LYSINE TRIMETHYLATION	23	-0.163059722	0.317786208
PROTEIN REFOLDING	19	-0.120369068	0.507742472
RRNA CATABOLIC PROCESS	15	-0.001434799	0.994580945
NEGATIVE REGULATION OF ENDOTHELIAL CELL MIGRATION	35	-0.11927682	0.372597543
REGULATION OF LEUKOCYTE DEGRANULATION	36	-0.192139644	0.135796345
UNSATURATED FATTY ACID METABOLIC PROCESS	76	0.029091481	0.758477031
RESPONSE TO SALT	14	0.072159119	0.744539524
REGULATION OF CYTOPLASMIC TRANSLATION	12	0.310090797	0.202087613
RESPONSE TO FUNGUS	36	0.044394389	0.747044501
ENDOCHONDRAL BONE MORPHOGENESIS	38	-0.151612755	0.232251044
DETECTION OF TEMPERATURE STIMULUS INVOLVED IN SENSORY PERCEPTION	9	0.553686547	0.02036236
GENERATION OF PRECURSOR METABOLITES AND ENERGY	256	-0.077760086	0.124498108
POSITIVE REGULATION OF ENDOTHELIAL CELL APOPTOTIC PROCESS	12	-0.272671518	0.20458258
SULFUR AMINO ACID METABOLIC PROCESS	36	-0.14549157	0.265704931
RESPONSE TO OXYGEN RADICAL	13	-0.292347044	0.145564007

ACTIVATION OF CYSTEINE TYPE ENDOPEPTIDASE ACTIVITY INVOLVED IN APOPTOTIC SIGNALING PATHWAY	13	-0.201371628	0.343650417
POSITIVE REGULATION OF T CELL APOPTOTIC PROCESS	12	-0.170819272	0.445120081
REGULATION OF HORMONE METABOLIC PROCESS	20	0.228866169	0.217936492
PROTEIN ACYLATION	146	0.008518779	0.900526006
CRANIOFACIAL SUTURE MORPHOGENESIS	12	-0.14969994	0.508031752
REGULATION OF LIPID CATABOLIC PROCESS	44	0.299317997	0.015432288
KIDNEY MORPHOGENESIS	70	0.098772604	0.321311579
LOCALIZATION WITHIN MEMBRANE	112	-0.127934119	0.088109861
CYTOKINESIS	77	-0.003470106	0.970360181
REGULATION OF PROTEIN EXIT FROM ENDOPLASMIC RETICULUM	16	-0.255321946	0.189383485
HEME BIOSYNTHETIC PROCESS	19	-0.409306523	0.011592295
NEURON PROJECTION GUIDANCE	176	0.138340435	0.028802939
POSITIVE REGULATION OF MEMBRANE PROTEIN ECTODOMAIN PROTEOLYSIS	13	0.085269031	0.710733925
CHONDROITIN SULFATE PROTEOGLYCAN METABOLIC PROCESS	40	0.059721244	0.648391118
EPITHELIAL CELL DIFFERENTIATION INVOLVED IN PROSTATE GLAND DEVELOPMENT	10	0.358855905	0.171372343
PROTEIN PALMITOYLATION	21	-0.235805242	0.151804317
DORSAL VENTRAL AXIS SPECIFICATION	18	0.252999757	0.194042724
PROTEIN CHROMOPHORE LINKAGE	6	-0.096032682	0.767588876
REGULATION OF P38MAPK CASCADE	22	0.157627586	0.375293181
FEEDING BEHAVIOR	66	0.125701671	0.220199592
REGULATION OF PROTEIN SERINE THREONINE KINASE ACTIVITY	427	-0.101104306	0.009982845
REGULATION OF INTERLEUKIN 1 SECRETION	25	0.076971341	0.642423556
REGULATION OF TRANSPORT	1500	-0.004074454	0.854635745
ESTABLISHMENT OR MAINTENANCE OF BIPOLAR CELL POLARITY	34	0.058079112	0.682757105
FOREBRAIN NEURON FATE COMMITMENT	9	0.499506359	0.043636832
SULFUR AMINO ACID BIOSYNTHETIC PROCESS	18	-0.004381114	0.981865241
CELLULAR RESPONSE TO VITAMIN	25	0.058429314	0.724070465
PEPTIDE HORMONE PROCESSING	25	0.064827548	0.695391281
CELLULAR RESPONSE TO VITAMIN D	14	-0.149648672	0.474158628
NEGATIVE REGULATION OF IMMUNE RESPONSE	101	-0.177651248	0.021907287
REGULATION OF RESPONSE TO OXIDATIVE STRESS	59	-0.008270826	0.938106104
POSITIVE REGULATION OF PROTEIN EXPORT FROM NUCLEUS	19	-0.348445275	0.034152447
GTP METABOLIC PROCESS	20	-0.246878429	0.143649961
PROTEIN K48 LINKED UBIQUITINATION	46	-0.211334342	0.063357713
MACROMITOPHAGY	113	-0.117832451	0.115294514
NUCLEOTIDE EXCISION REPAIR DNA DUPLEX UNWINDING	22	0.501326571	0.002239528
T CELL MEDIATED IMMUNITY	24	0.032606568	0.84628132
DNA DAMAGE RESPONSE DETECTION OF DNA DAMAGE	36	0.131313601	0.350715717
MITOCHONDRION LOCALIZATION	33	-0.142682754	0.298375052
MATURATION OF 5 8S RRNA	28	-0.126177793	0.397536463
REGULATION OF POTASSIUM ION TRANSMEMBRANE TRANSPORT	47	-0.013046123	0.912823109
ASPARTATE METABOLIC PROCESS	9	-0.329325265	0.177095117
REGULATION OF DENDRITE EXTENSION	17	-0.027402528	0.889532302
MECHANOSENSORY BEHAVIOR	9	-0.391259205	0.104589735
BONE RESORPTION	18	-0.176124207	0.338589552
AXONEME ASSEMBLY	32	0.321808387	0.025291453
REGULATION OF IMMUNE EFFECTOR PROCESS	358	-0.047956747	0.267894639
GANGLION DEVELOPMENT	12	-0.04121732	0.860133437
REGULATION OF SECONDARY METABOLIC PROCESS	9	-0.136458231	0.601530713
REGULATION OF NUCLEOTIDE METABOLIC PROCESS	165	0.029352841	0.64856902
CALCIUM ION TRANSMEMBRANE TRANSPORT	129	0.024468005	0.736417703
HOMOPHILIC CELL ADHESION VIA PLASMA MEMBRANE ADHESION MOLECULES	116	0.038519258	0.616021851
DEVELOPMENTAL GROWTH INVOLVED IN MORPHOGENESIS	93	0.012402916	0.88440535
CARDIAC SEPTUM MORPHOGENESIS	44	0.177144656	0.158903777
REGULATION OF TRANSCRIPTION INVOLVED IN CELL FATE COMMITMENT	13	0.13425152	0.562990693
CENTRAL NERVOUS SYSTEM NEURON DEVELOPMENT	61	0.117826195	0.270770567
POSITIVE REGULATION OF T CELL MEDIATED IMMUNITY	28	-0.133992807	0.36799855
EXOCYTOSIS	261	-0.059779326	0.23524301
REGULATION OF HISTONE H4 ACETYLATION	11	0.008834292	0.971491196
POSITIVE REGULATION OF LIPID STORAGE	16	-0.179549123	0.356630657
PYRIMIDINE NUCLEOSIDE METABOLIC PROCESS	43	-0.170708002	0.15142447
REGULATION OF SISTER CHROMATID COHESION	17	-0.336436454	0.056764758
HEPARAN SULFATE PROTEOGLYCAN BIOSYNTHETIC PROCESS	19	0.087829481	0.644952061

REGULATION OF CARDIAC MUSCLE CONTRACTION	59	-0.141740923	0.166609782
ZINC ION HOMEOSTASIS	18	0.259838637	0.181343848
POSITIVE REGULATION OF I KAPPAB KINASE NF KAPPAB SIGNALING	167	-0.114329631	0.064185706
EYE PHOTORECEPTOR CELL DEVELOPMENT	26	-0.038053283	0.810969499
NEGATIVE REGULATION OF OSTEOBLAST DIFFERENTIATION	38	0.110145827	0.414204617
REGULATION OF VIRAL INDUCED CYTOPLASMIC PATTERN RECOGNITION RECEPTOR SIGNALING PATHWAY	12	-0.255002621	0.238668546
GLYCOSYLATION	228	-0.074454263	0.165147123
ANGIOGENESIS INVOLVED IN WOUND HEALING	13	-0.465557529	0.025783653
REGULATION OF RECEPTOR RECYCLING	18	-0.201963296	0.266995035
REGULATION OF INTERLEUKIN 2 PRODUCTION	46	0.066427399	0.587251084
REGULATION OF PROTEIN COMPLEX DISASSEMBLY	199	0.001303711	0.982186762
NEGATIVE REGULATION OF MYELOID CELL DIFFERENTIATION	77	-0.054262815	0.556444564
POSITIVE REGULATION OF SMOOTHENED SIGNALING PATHWAY	21	0.097258218	0.59248419
NEUTROPHIL ACTIVATION INVOLVED IN IMMUNE RESPONSE	9	-0.027014039	0.920600532
POSITIVE REGULATION OF EXTRINSIC APOPTOTIC SIGNALING PATHWAY	51	-0.008268756	0.942446077
REGULATION OF MUSCLE SYSTEM PROCESS	168	0.014488381	0.819955356
ARACHIDONATE TRANSPORT	11	0.232970366	0.345803784
LONG CHAIN FATTY ACID TRANSPORT	38	0.217341179	0.106222913
ACTIN NUCLEATION	22	0.12885276	0.46878793
CHROMATIN REMODELING	141	-0.187300331	0.004335444
IMPORT INTO CELL	32	-0.083877385	0.55393723
CALCIUM INDEPENDENT CELL CELL ADHESION VIA PLASMA MEMBRANE CELL ADHESION MOLECULES	14	0.094291884	0.671125074
PHOSPHOLIPID CATABOLIC PROCESS	26	-0.116763452	0.454023359
REGULATION OF EXTRACELLULAR MATRIX DISASSEMBLY	12	0.100235329	0.676847387
REGULATION OF PRI MIRNA TRANSCRIPTION FROM RNA POLYMERASE II PROMOTER	15	-0.286561811	0.14364553
COLLECTING DUCT DEVELOPMENT	10	0.547366592	0.026500098
HEXOSE METABOLIC PROCESS	137	-0.048775665	0.482191555
REGULATION OF TRIGLYCERIDE BIOSYNTHETIC PROCESS	18	0.180483683	0.360074334
REGULATION OF RESPONSE TO CYTOKINE STIMULUS	127	-0.103466691	0.145299432
NEUROMUSCULAR PROCESS	78	0.058303573	0.534442146
ACTIN CYTOSKELETON REORGANIZATION	51	-0.009034925	0.937112991
PURINE CONTAINING COMPOUND METABOLIC PROCESS	350	-0.075060368	0.08411161
NEGATIVE REGULATION OF CD4 POSITIVE ALPHA BETA T CELL ACTIVATION	14	-0.039644347	0.854781975
LIPID LOCALIZATION	218	0.204888601	0.000332368
HETEROCHROMATIN ORGANIZATION	11	0.051650755	0.835984139
GLAND DEVELOPMENT	339	-0.025863114	0.562754809
RESPONSE TO MERCURY ION	12	0.14040804	0.55768265
REGULATION OF UBIQUITIN PROTEIN LIGASE ACTIVITY	17	0.034719654	0.862087916
BETA CATENIN TCF COMPLEX ASSEMBLY	41	-0.096374274	0.439365761
ACTIVATION OF PROTEIN KINASE ACTIVITY	246	-0.007451574	0.887136328
REGULATION OF CELL CYCLE PROCESS	511	-0.058791904	0.105756436
ESTABLISHMENT OR MAINTENANCE OF CELL POLARITY	125	-0.01104706	0.880196475
IMMUNE RESPONSE REGULATING CELL SURFACE RECEPTOR SIGNALING PATHWAY	251	-0.05579414	0.277742511
VESICLE DOCKING	52	-0.063128288	0.572536432
OLIGODENDROCYTE DIFFERENTIATION	49	0.12572315	0.292211847
NEUROMUSCULAR SYNAPTIC TRANSMISSION	16	-0.26882275	0.144860419
CELL KILLING	43	0.187959576	0.138702195
CELL CYCLE CHECKPOINT	184	-0.118508318	0.044301298
NEPHRON TUBULE FORMATION	13	0.15012965	0.51746711
NEGATIVE REGULATION OF CARDIAC MUSCLE TISSUE DEVELOPMENT	16	0.040393401	0.844703826
NEGATIVE REGULATION OF ION TRANSPORT	110	-0.006597559	0.932738026
CEREBRAL CORTEX RADIALLY ORIENTED CELL MIGRATION	28	-0.128242572	0.391618388
LYMPH VESSEL DEVELOPMENT	18	0.006315692	0.973920349
RESPONSE TO CADMIUM ION	39	-0.017116104	0.895774772
RESPONSE TO CYTOKINE	604	-0.030867172	0.360476558
REGULATION OF CARDIAC CONDUCTION	54	-0.041751109	0.705340796
POSITIVE REGULATION OF EXTRINSIC APOPTOTIC SIGNALING PATHWAY VIA DEATH DOMAIN RECEPTORS	17	-0.325613871	0.063454401
SPLICEOSOMAL COMPLEX ASSEMBLY	51	-0.159231114	0.146732533
SNRNA PROCESSING	22	-0.093333202	0.583852789
POSITIVE REGULATION OF CELL DIFFERENTIATION	727	0.021913769	0.482835342
ATRIOVENTRICULAR VALVE DEVELOPMENT	17	0.238594333	0.234673204

RRNA MODIFICATION	24	-0.193761159	0.227979405
POSITIVE REGULATION OF MONOOXYGENASE ACTIVITY	24	0.097875952	0.564288895
POSITIVE REGULATION OF DENDRITIC SPINE MORPHOGENESIS	12	-0.439734269	0.035789846
PROSTATE GLAND DEVELOPMENT	38	-0.027339788	0.835971425
MICROVILLUS ORGANIZATION	20	-0.149684655	0.394146105
ADENOHYPOPHYSIS DEVELOPMENT	10	0.086510501	0.741352025
REGULATION OF T HELPER 1 TYPE IMMUNE RESPONSE	16	0.136479965	0.511966368
REGULATION OF INTERLEUKIN 6 PRODUCTION	84	0.016897068	0.850775543
SECONDARY METABOLIC PROCESS	38	0.073544299	0.58431198
NEGATIVE REGULATION OF GLUCONEOGENESIS	10	0.039399777	0.879921061
REGULATION OF TRANSMISSION OF NERVE IMPULSE	12	0.380712576	0.09905761
FATTY ACID DERIVATIVE BIOSYNTHETIC PROCESS	31	-0.005127951	0.97215479
GLUTAMATE RECEPTOR SIGNALING PATHWAY	33	-0.111413019	0.420595122
PYRUVATE METABOLIC PROCESS	53	-0.037314959	0.738071634
POSITIVE REGULATION OF ERBB SIGNALING PATHWAY	31	0.132493603	0.381861549
REGULATION OF POTASSIUM ION TRANSMEMBRANE TRANSPORTER ACTIVITY	35	-0.023598154	0.86393833
NITRIC OXIDE MEDIATED SIGNAL TRANSDUCTION	14	0.131920105	0.554976754
POSITIVE REGULATION OF TYROSINE PHOSPHORYLATION OF STAT5 PROTEIN	13	-0.058595563	0.793278924
NEGATIVE REGULATION OF ENDOTHELIAL CELL APOPTOTIC PROCESS	24	-0.015482111	0.925929738
ESTABLISHMENT OF MITOTIC SPINDLE LOCALIZATION	23	-0.085064412	0.610753773
STEROL METABOLIC PROCESS	103	0.023308447	0.774245654
RETINA DEVELOPMENT IN CAMERA TYPE EYE	109	-0.022353582	0.775111432
REGULATION OF POSTTRANSCRIPTIONAL GENE SILENCING	20	0.026270493	0.886499775
POST ANAL TAIL MORPHOGENESIS	15	0.091976777	0.66747651
PEPTIDYL ASPARAGINE MODIFICATION	37	-0.261303126	0.036941296
POSITIVE REGULATION OF ENDOTHELIAL CELL PROLIFERATION	63	-0.047810845	0.639475243
CYTOLYSIS	15	0.224734978	0.292178419
REGULATION OF VIRAL GENOME REPLICATION	69	-0.041075641	0.674420371
MAMMARY GLAND EPITHELIAL CELL PROLIFERATION	12	-0.337383393	0.101137708
REPRODUCTION	1020	0.070056458	0.009095425
REGULATION OF STAT CASCADE	113	-0.027286573	0.722264107
REGULATION OF ION TRANSPORT	472	0.058595615	0.129688165
DORSAL SPINAL CORD DEVELOPMENT	11	-0.066479883	0.784365082
NEURON DEATH	44	-0.172531498	0.143076453
POSITIVE REGULATION OF AUTOPHAGY	71	-0.073859996	0.439489879
SIGNAL TRANSDUCTION BY PROTEIN PHOSPHORYLATION	354	0.014945476	0.734855063
RECEPTOR MEDIATED ENDOCYTOSIS	176	0.188106017	0.003012114
POSITIVE REGULATION OF ACTIN FILAMENT BUNDLE ASSEMBLY	44	-0.049024227	0.687861441
2 OXOGLUTARATE METABOLIC PROCESS	17	-0.184174206	0.325102726
TRYPTOPHAN METABOLIC PROCESS	11	0.358489395	0.1336807
INOSITOL PHOSPHATE MEDIATED SIGNALING	15	-0.021767749	0.917544382
REGULATION OF MEMBRANE PERMEABILITY	69	-0.124323983	0.192434886
ANDROGEN RECEPTOR SIGNALING PATHWAY	38	0.088511328	0.512802183
NEGATIVE REGULATION OF DNA TEMPLATED TRANSCRIPTION ELONGATION	12	0.007623369	0.974298714
POSITIVE REGULATION OF STEM CELL PROLIFERATION	51	-0.09605616	0.390704919
GERM CELL DEVELOPMENT	158	0.082500852	0.213690537
NEGATIVE REGULATION OF RECEPTOR MEDIATED ENDOCYTOSIS	14	-0.336728362	0.083210996
DNA LIGATION	16	0.233394402	0.266298701
REGULATION OF SPROUTING ANGIOGENESIS	28	0.03898551	0.80256678
CELLULAR MACROMOLECULE LOCALIZATION	1126	-0.055309929	0.027451551
REGULATION OF CELLULAR RESPONSE TO INSULIN STIMULUS	51	-0.071504675	0.525810911
NEPHRON DEVELOPMENT	97	-0.036994524	0.654305848
POSITIVE REGULATION OF RESPONSE TO DNA DAMAGE STIMULUS	61	0.043091346	0.683931538
PURINE CONTAINING COMPOUND CATABOLIC PROCESS	46	0.0694531	0.570132826
REGULATION OF LIPOPROTEIN PARTICLE CLEARANCE	10	-0.072719562	0.774347245
PLATELET DEGRANULATION	91	-0.072648153	0.389650005
POSITIVE REGULATION OF INFLAMMATORY RESPONSE	88	0.123364668	0.165248597
LIPID OXIDATION	61	0.137992203	0.195860049
INTEGRIN MEDIATED SIGNALING PATHWAY	73	-0.063889922	0.498861614
NOTOCHORD DEVELOPMENT	15	-0.24057194	0.213948795
DICARBOXYLIC ACID CATABOLIC PROCESS	13	-0.115591912	0.598843322
NAD BIOSYNTHETIC PROCESS	12	-0.33711	0.114037175
REGULATION OF SULFUR METABOLIC PROCESS	20	-0.433756669	0.003633043
SKIN DEVELOPMENT	167	0.125852433	0.052641461
ADENYLATE CYCLASE ACTIVATING G PROTEIN COUPLED RECEPTOR SIGNALING PATHWAY	46	0.153621863	0.212250498

MESODERM DEVELOPMENT	99	0.040539712	0.625842242
GENITALIA DEVELOPMENT	34	0.105309285	0.460502733
NEGATIVE REGULATION OF CALCIUM MEDIATED SIGNALING	18	0.026987009	0.889346392
REGULATION OF TYROSINE PHOSPHORYLATION OF STAT1 PROTEIN	15	0.247583736	0.256335691
CELLULAR RESPONSE TO STRESS	1420	-0.050773462	0.024704071
PATTERN SPECIFICATION PROCESS	352	0.009249999	0.834237808
REGULATION OF ALCOHOL BIOSYNTHETIC PROCESS	40	0.231657437	0.077740179
PHOSPHATIDIC ACID METABOLIC PROCESS	27	0.155325168	0.334945276
NEGATIVE REGULATION OF VIRAL GENOME REPLICATION	43	-0.122497381	0.310623932
ANGIOGENESIS	266	0.029706755	0.559486818
EPITHELIAL CELL DIFFERENTIATION	405	0.056460209	0.17518743
REGULATION OF NATURAL KILLER CELL MEDIATED IMMUNITY	27	-0.163831874	0.274271557
LYMPHOID PROGENITOR CELL DIFFERENTIATION	11	0.296068893	0.240897546
METHYLATION	244	-0.039485329	0.450514904
CELL SURFACE RECEPTOR SIGNALING PATHWAY INVOLVED IN HEART DEVELOPMENT	16	0.326056468	0.109102733
PROTEIN IMPORT INTO MITOCHONDRIAL MATRIX	12	-0.131528867	0.562327014
MODULATION OF SYNAPTIC TRANSMISSION	244	0.083148323	0.120575682
CELLULAR RESPONSE TO REACTIVE NITROGEN SPECIES	16	-0.121159727	0.540569055
POSITIVE REGULATION OF VASCULAR ENDOTHELIAL GROWTH FACTOR RECEPTOR SIGNALING PATHWAY	16	-0.030220713	0.881752278
REGULATION OF CATECHOLAMINE METABOLIC PROCESS	14	-0.062611746	0.771101434
MELANOCYTE DIFFERENTIATION	17	0.236699069	0.237016536
RNA CATABOLIC PROCESS	221	-0.12468715	0.020238963
DNA DOUBLE STRAND BREAK PROCESSING	19	-0.00493124	0.979027546
ESTABLISHMENT OF MITOCHONDRION LOCALIZATION	17	-0.159471285	0.40326607
UBIQUITIN DEPENDENT PROTEIN CATABOLIC PROCESS VIA THE MULTIVESICULAR BODY SORTING PATHWAY	17	0.247135702	0.231019096
FATTY ACID TRANSPORT	51	0.178051398	0.127425198
VENTRICULAR TRABECULA MYOCARDIUM MORPHOGENESIS	10	-0.090879462	0.719683753
REGULATION OF CELL SHAPE	125	0.024378221	0.741277145
ARACHIDONIC ACID METABOLIC PROCESS	33	0.092276934	0.523271547
RESPONSE TO ETHER	9	0.221290407	0.426615304
POSITIVE REGULATION OF TRANSCRIPTION FROM RNA POLYMERASE II PROMOTER INVOLVED IN CELLULAR RESPONSE TO CHEMICAL STIMULUS	26	-0.024242852	0.879322861
POSITIVE REGULATION OF LAMELLIPODIUM ORGANIZATION	23	0.055737614	0.746806689
REGULATION OF BLOOD VOLUME BY RENIN ANGIOTENSIN	8	0.360499916	0.19300868
RESPONSE TO NUTRIENT	170	0.05789803	0.36395196
ORGANELLE LOCALIZATION	377	-0.050218197	0.234014919
VENOUS BLOOD VESSEL DEVELOPMENT	14	-0.095664914	0.654306161
EXECUTION PHASE OF APOPTOSIS	51	-0.053755382	0.635037635
CELL DEVELOPMENT	1210	0.052346409	0.034438546
PRODUCTION OF MOLECULAR MEDIATOR OF IMMUNE RESPONSE	52	-0.026229463	0.816387281
TELENCEPHALON DEVELOPMENT	198	0.05064527	0.391476504
PROSTANOID METABOLIC PROCESS	24	-0.122945783	0.445505993
MEMBRANE REPOLARIZATION	12	-0.224334299	0.31236636
CEREBELLAR PURKINJE CELL LAYER MORPHOGENESIS	12	-0.206532773	0.353072523
CLATHRIN MEDIATED ENDOCYTOSIS	35	0.181009182	0.198985433
PHOSPHATIDYLETHANOLAMINE ACYL CHAIN REMODELING	16	0.188041234	0.365479428
RESPONSE TO GONADOTROPIN	23	0.289079437	0.098481869
CAMERA TYPE EYE MORPHOGENESIS	83	-0.027453015	0.758981588
RESPONSE TO COBALT ION	13	0.031160764	0.891499723
NEUROTROPHIN TRK RECEPTOR SIGNALING PATHWAY	13	-0.50012554	0.007077208
MATERNAL PLACENTA DEVELOPMENT	29	0.065546661	0.670430084
MACROMOLECULE METHYLATION	179	-0.020240855	0.740988975
STRESS ACTIVATED PROTEIN KINASE SIGNALING CASCADE	89	0.047444923	0.588798082
REGULATION OF PLATELET DERIVED GROWTH FACTOR RECEPTOR SIGNALING PATHWAY	14	-0.282080998	0.156756383
POSITIVE REGULATION OF SMALL GTPASE MEDIATED SIGNAL TRANSDUCTION	34	0.222383053	0.12516132
CHROMATIN SILENCING AT RDNA	35	0.066695285	0.633801102
RESPONSE TO FOLIC ACID	12	0.181412181	0.448901447
GROWTH PLATE CARTILAGE DEVELOPMENT	9	0.209153921	0.447202953
O GLYCAN PROCESSING	49	0.004562722	0.968936168
POSITIVE REGULATION OF CYCLIC NUCLEOTIDE METABOLIC PROCESS	77	0.11010454	0.246299923
REACTIVE OXYGEN SPECIES METABOLIC PROCESS	74	-0.017568649	0.853210766
REGULATION OF AUTOPHAGOSOME ASSEMBLY	32	0.163482929	0.268735152

NEUTRAL AMINO ACID TRANSPORT	25	-0.000966953	0.995287245
POSITIVE REGULATION OF MULTI ORGANISM PROCESS	144	0.090831507	0.19195678
MESENCHYME MORPHOGENESIS	36	0.231722432	0.092205086
CENTROSOME DUPLICATION	28	-0.173981973	0.234337999
REGULATION OF BINDING	264	0.052499515	0.306201779
POSITIVE REGULATION OF IMMUNE RESPONSE	443	-0.017550459	0.655208005
POSITIVE REGULATION OF CHEMOTAXIS	112	-0.062624336	0.412484879
REGULATION OF RHO PROTEIN SIGNAL TRANSDUCTION	94	-0.144041491	0.07637985
NEGATIVE REGULATION OF REACTIVE OXYGEN SPECIES BIOSYNTHETIC PROCESS	14	0.274147469	0.207684256
REGULATION OF ARF PROTEIN SIGNAL TRANSDUCTION	15	-0.187068597	0.354237828
PROTEIN AUTOPHOSPHORYLATION	175	-0.003757381	0.951790984
CELLULAR COMPONENT DISASSEMBLY	455	-0.14923942	7.00E-05
REGULATION OF GLIOGENESIS	80	-0.016476062	0.856897643
AMINOGLYCAN CATABOLIC PROCESS	56	-0.00317401	0.976863071
POSITIVE REGULATION OF ORGANIC ACID TRANSPORT	25	0.172567334	0.29823863
MUSCLE CELL DIFFERENTIATION	205	0.006117473	0.915417614
ACTIVATION OF TRANSMEMBRANE RECEPTOR PROTEIN TYROSINE KINASE ACTIVITY	9	0.448055317	0.129884667
PHOSPHATIDYLCHOLINE ACYL CHAIN REMODELING	18	0.223547521	0.254480716
RNA LOCALIZATION	172	-0.250213123	2.16E-05
MULTI ORGANISM MEMBRANE ORGANIZATION	30	0.208360061	0.174957148
POSITIVE REGULATION OF EMBRYONIC DEVELOPMENT	29	-0.017287502	0.909129555
HISTONE H4 ACETYLATION	47	0.214564351	0.080637538
MATING BEHAVIOR	19	0.248319045	0.190753332
RIBOSOMAL LARGE SUBUNIT BIOGENESIS	49	-0.052124914	0.652002458
REGULATION OF PROTEIN POLYUBIQUITINATION	13	0.094878529	0.680069234
INACTIVATION OF MAPK ACTIVITY	26	-0.270624758	0.069513135
PEPTIDYL ARGININE METHYLATION	11	-0.088819291	0.712211119
NEGATIVE REGULATION OF OXIDATIVE STRESS INDUCED INTRINSIC APOPTOTIC SIGNALING PATHWAY	19	-0.004126497	0.982452568
HISTONE PHOSPHORYLATION	23	-0.541443795	8.48E-05
POSITIVE REGULATION OF TRANSMEMBRANE RECEPTOR PROTEIN SERINE THREONINE KINASE SIGNALING PATHWAY	85	0.077865134	0.388344885
MULTICELLULAR ORGANISM REPRODUCTION	586	0.063587343	0.068207025
POSITIVE REGULATION OF INTERFERON GAMMA PRODUCTION	55	0.184388797	0.100385975
NEGATIVE REGULATION OF NECROTIC CELL DEATH	11	0.031700239	0.89838278
REGULATION OF ORGANELLE ORGANIZATION	1058	-0.029061743	0.26249111
PHOSPHORYLATION	1073	-0.001601714	0.950693079
REGULATION OF STRIATED MUSCLE CELL APOPTOTIC PROCESS	19	0.132013283	0.490177467
STRIATED MUSCLE ADAPTATION	17	0.091992246	0.647637791
BRANCHING MORPHOGENESIS OF AN EPITHELIAL TUBE	119	0.01175193	0.876260396
REGULATION OF RAS PROTEIN SIGNAL TRANSDUCTION	161	-0.111217265	0.077604834
NEGATIVE REGULATION OF CYTOKINE PRODUCTION	173	-0.038083201	0.539118227
POSITIVE REGULATION OF DENDRITIC SPINE DEVELOPMENT	31	-0.225143103	0.103715054
POSITIVE REGULATION OF VIRAL PROCESS	92	0.125722418	0.150538506
PROSTATE GLANDULAR ACINUS DEVELOPMENT	10	0.01860025	0.942916461
REGULATION OF PROTEOLYSIS	613	-0.0418068	0.211159727
ENDOCYTOSIS	403	0.085908789	0.040559496
PROTEIN LOCALIZATION TO NUCLEUS	143	-0.07513027	0.266000728
PEPTIDE CATABOLIC PROCESS	23	-0.006153072	0.971206705
REGULATION OF MHC CLASS II BIOSYNTHETIC PROCESS	12	0.034194676	0.885696046
RAC PROTEIN SIGNAL TRANSDUCTION	19	0.181303127	0.345576494
NEGATIVE REGULATION OF POTASSIUM ION TRANSMEMBRANE TRANSPORT	17	-0.081603613	0.675267331
REGULATION OF VIRAL ENTRY INTO HOST CELL	25	0.167620172	0.31656967
CELLULAR COMPONENT DISASSEMBLY INVOLVED IN EXECUTION PHASE OF APOPTOSIS	41	-0.074247589	0.554306722
POSITIVE REGULATION OF SECRETION	298	0.010967442	0.819151456
NEGATIVE REGULATION OF WNT SIGNALING PATHWAY	176	0.050869773	0.416572195
POSITIVE REGULATION OF CELL CYCLE	299	-0.013047183	0.784231906
REGULATION OF CELLULAR PROTEIN LOCALIZATION	501	-0.08258247	0.023638108
POSITIVE REGULATION OF B CELL ACTIVATION	61	-0.023395188	0.822672815
NEURONAL ACTION POTENTIAL	19	0.181015504	0.343075586
REGULATION OF TELOMERE MAINTENANCE VIA TELOMERE LENGTHENING	46	-0.04885988	0.682511283
RETROGRADE TRANSPORT VESICLE RECYCLING WITHIN GOLGI	19	0.024103032	0.898383972
POSITIVE REGULATION OF INTERLEUKIN 10 PRODUCTION	28	0.224464851	0.155825655

PARASYMPATHETIC NERVOUS SYSTEM DEVELOPMENT	15	0.021349861	0.919877971
BASEMENT MEMBRANE ORGANIZATION	11	0.53936166	0.02067561
SOMITE DEVELOPMENT	67	0.092885693	0.360868108
NEGATIVE REGULATION OF IMMUNE EFFECTOR PROCESS	90	-0.067372648	0.428262507
NEGATIVE REGULATION OF VIRAL RELEASE FROM HOST CELL	15	0.126949347	0.555152969
SERINE FAMILY AMINO ACID METABOLIC PROCESS	37	-0.023798636	0.858942413
SERTOLI CELL DIFFERENTIATION	15	0.065388084	0.759737649
NEGATIVE REGULATION OF CALCIUM ION TRANSMEMBRANE TRANSPORT	26	-0.016314696	0.918768212
RNA SPLICING	347	-0.140531027	0.001051092
VENTRICULAR CARDIAC MUSCLE CELL DEVELOPMENT	11	0.076626507	0.75924537
POSITIVE REGULATION OF PROTEIN POLYMERIZATION	79	0.096569512	0.302984469
REGULATION OF MUSCLE ADAPTATION	56	0.139457033	0.212210084
NEGATIVE REGULATION OF RESPONSE TO WOUNDING	129	0.101051762	0.168909497
OLFACTORY BULB INTERNEURON DEVELOPMENT	11	0.187813909	0.459019609
NEGATIVE REGULATION OF MULTICELLULAR ORGANISMAL PROCESS	836	0.013666208	0.639595831
FEMALE GENITALIA DEVELOPMENT	13	0.16872513	0.465323615
NEUROBLAST PROLIFERATION	27	0.071015708	0.656397437
ERYTHROSE 4 PHOSPHATE PHOSPHOENOLPYRUVATE FAMILY AMINO ACID METABOLIC PROCESS	10	0.104845829	0.689391494
MIDGUT DEVELOPMENT	13	0.157161762	0.495167871
MITOCHONDRION DISTRIBUTION	10	-0.176060887	0.473348925
PEPTIDYL METHIONINE MODIFICATION	11	-0.245825935	0.279524762
IMMUNOLOGICAL SYNAPSE FORMATION	6	0.058738911	0.861755959
VITAMIN METABOLIC PROCESS	107	-0.033679956	0.668849012
MUSCLE CELL DEVELOPMENT	107	-0.054327661	0.48801022
REGULATION OF DEFENSE RESPONSE TO VIRUS BY HOST	119	-0.105567052	0.149419278
REGULATION OF NEURAL PRECURSOR CELL PROLIFERATION	60	-0.097971529	0.341972883
NUCLEOTIDE SUGAR BIOSYNTHETIC PROCESS	18	0.337078203	0.081034401
NEGATIVE REGULATION OF AXONOGENESIS	59	0.018092187	0.865862671
LEUKOCYTE MIGRATION INVOLVED IN INFLAMMATORY RESPONSE	10	0.121522517	0.643061799
OSTEOCLAST DIFFERENTIATION	26	-0.004127429	0.979473952
CARBOHYDRATE DERIVATIVE TRANSPORT	46	0.024462525	0.840308873
DEFINITIVE HEMOPOIESIS	15	0.170116951	0.431390223
RENAL FILTRATION	9	-0.348980126	0.144775047
LYMPHOCYTE ACTIVATION INVOLVED IN IMMUNE RESPONSE	79	-0.025643623	0.779866038
POSITIVE REGULATION OF LEUKOCYTE CHEMOTAXIS	75	-0.118157887	0.197780959
RESPONSE TO TUMOR NECROSIS FACTOR	197	-0.114942918	0.043467068
POSITIVE REGULATION OF CELL SUBSTRATE ADHESION	92	-0.049651881	0.557015488
PROTEIN ALKYLATION	102	-0.114394686	0.147570081
ATRIOVENTRICULAR VALVE MORPHOGENESIS	15	0.204247766	0.341466
REGULATION OF INTRACELLULAR TRANSPORT	546	-0.083792841	0.016684709
CARDIAC CONDUCTION	68	0.089952068	0.37318332
AORTA MORPHOGENESIS	19	-0.009183242	0.960918206
RESPONSE TO ALCOHOL	316	-0.020300242	0.661088252
CELLULAR HOMEOSTASIS	553	-0.023278519	0.509243864
POSITIVE REGULATION OF MULTICELLULAR ORGANISM GROWTH	26	0.093107021	0.567525684
MEMBRANE LIPID BIOSYNTHETIC PROCESS	100	-0.100450282	0.208625262
POSITIVE REGULATION OF DEPHOSPHORYLATION	44	0.120267211	0.33809931
PROTEIN INSERTION INTO MEMBRANE	19	0.231217292	0.223555243
ACTIVATION OF MAPK ACTIVITY	117	-0.009483654	0.900386883
EMBRYONIC ORGAN DEVELOPMENT	355	0.068413154	0.124041178
POSITIVE REGULATION OF NATURAL KILLER CELL MEDIATED IMMUNITY	16	-0.143763007	0.463719569
POSITIVE REGULATION OF ORGAN GROWTH	34	0.069864418	0.623037881
EPOXYGENASE P450 PATHWAY	12	0.089481608	0.708518656
MAMMARY GLAND DUCT MORPHOGENESIS	24	-0.106540112	0.511337523
NEGATIVE REGULATION OF ESTABLISHMENT OF PROTEIN LOCALIZATION TO PLASMA MEMBRANE	12	0.071958384	0.763575871
AMINO ACID IMPORT	13	0.149891047	0.519935255
IMP BIOSYNTHETIC PROCESS	11	-0.059508032	0.806817067
POSITIVE REGULATION OF CELL CYCLE G1 S PHASE TRANSITION	27	-0.016248921	0.917570057
LYMPHOCYTE APOPTOTIC PROCESS	18	0.291051592	0.136940435
NEGATIVE REGULATION OF TRANSMEMBRANE RECEPTOR PROTEIN SERINE THREONINE KINASE SIGNALING PATHWAY	90	0.105869256	0.229449225
FATTY ACID DERIVATIVE METABOLIC PROCESS	65	0.069241203	0.500853708
REGULATION OF INTERFERON ALPHA PRODUCTION	18	-0.109680677	0.558003215
NEGATIVE REGULATION OF INTERLEUKIN 17 PRODUCTION	9	-0.084986624	0.750499201

GLYCOSYL COMPOUND CATABOLIC PROCESS	36	0.016032503	0.906811902
PROTEIN HYDROXYLATION	15	0.215025915	0.327639162
PEPTIDYL LYSINE METHYLATION	66	-0.037820354	0.705457324
REGULATION OF RESPONSE TO TUMOR CELL	9	-0.146545366	0.574132852
PROTEIN TETRAMERIZATION	117	-0.006627319	0.930339824
REGULATION OF TISSUE REMODELING	55	-0.080192684	0.459233277
RESPONSE TO ISOQUINOLINE ALKALOID	23	-0.132387276	0.421345454
POSITIVE REGULATION OF PEPTIDYL SERINE PHOSPHORYLATION	70	-0.030623939	0.752982284
HISTONE H3 ACETYLATION	46	0.109997654	0.371101494
ENDOTHELIAL TUBE MORPHOGENESIS	11	-0.210629785	0.359636128
REGULATION OF ANION TRANSPORT	109	0.194438417	0.015159778
CELLULAR RESPONSE TO DEXAMETHASONE STIMULUS	23	0.011046167	0.94852397
NEGATIVE REGULATION OF DNA REPLICATION	51	-0.143453267	0.193575972
RESPONSE TO OSMOTIC STRESS	58	-0.109554379	0.29513421
REGULATION OF TRANSCRIPTION REGULATORY REGION DNA BINDING	34	0.012468944	0.929442853
POSITIVE REGULATION OF INTERLEUKIN 6 PRODUCTION	57	0.070530286	0.521180616
RUFFLE ORGANIZATION	20	-0.183646099	0.286932257
DNA PACKAGING	166	-0.123762882	0.044945049
MUSCLE CELL MIGRATION	17	-0.043181858	0.826031213
BRANCHING INVOLVED IN SALIVARY GLAND MORPHOGENESIS	16	-0.029426519	0.884871623
NEGATIVE REGULATION OF TRANSCRIPTION FROM RNA POLYMERASE II PROMOTER	659	0.02118086	0.517405938
SINGLE ORGANISM CATABOLIC PROCESS	797	-0.021589883	0.466428183
NEGATIVE REGULATION OF PROTEIN MATURATION	30	0.018310607	0.902884163
BINDING OF SPERM TO ZONA PELLUCIDA	25	-0.005910388	0.971168669
RNA PHOSPHODIESTER BOND HYDROLYSIS EXONUCLEOLYTIC	35	0.029057525	0.834687821
ADAPTATION OF SIGNALING PATHWAY	17	-0.057731732	0.76795152
TRANSCYTOSIS	9	0.33853778	0.211346214
EPITHELIAL CELL MATURATION	13	0.296806996	0.227268359
NEGATIVE REGULATION OF GENE SILENCING	17	0.090069936	0.655703413
REGULATION OF CELL DEVELOPMENT	727	0.040279091	0.198608893
REGULATION OF INTRINSIC APOPTOTIC SIGNALING PATHWAY	133	0.022527741	0.752959903
ESTABLISHMENT OF PROTEIN LOCALIZATION TO PLASMA MEMBRANE	83	-0.01122628	0.900532462
REGULATION OF MEMBRANE REPOLARIZATION	25	-0.099614961	0.533466644
NEGATIVE REGULATION OF ORGANELLE ORGANIZATION	357	-0.06694397	0.120770674
SNRNA METABOLIC PROCESS	82	-0.031308718	0.727762431
NEGATIVE REGULATION OF CELL DIFFERENTIATION	519	0.0478923	0.193934251
PEPTIDYL LYSINE DIMETHYLATION	12	0.499693411	0.032354125
PROTEIN IMPORT INTO PEROXISOME MATRIX	12	0.226932609	0.346716859
POSITIVE REGULATION OF ATPASE ACTIVITY	38	-0.164700291	0.194449524
ESTABLISHMENT OF PROTEIN LOCALIZATION TO GOLGI	15	0.161133495	0.456292836
POSITIVE REGULATION OF POTASSIUM ION TRANSMEMBRANE TRANSPORT	21	-0.17949872	0.291250163
LIMBIC SYSTEM DEVELOPMENT	84	0.09933929	0.275555089
NEGATIVE REGULATION OF CELL KILLING	15	-0.279187348	0.148237592
POSITIVE REGULATION OF MONOCYTE CHEMOTAXIS	15	0.101235945	0.636389653
REGULATION OF CELLULAR PH	58	0.093836762	0.390400339
NEGATIVE REGULATION OF LEUKOCYTE DIFFERENTIATION	70	-0.106545895	0.263276011
AXIS ELONGATION	26	-0.08794434	0.575226527
REGULATION OF MITOTIC CELL CYCLE	441	-0.081406256	0.035943879
CATABOLIC PROCESS	1521	-0.042831517	0.051089282
T CELL DIFFERENTIATION	109	0.027210815	0.730759033
REGULATION OF CATION CHANNEL ACTIVITY	68	-0.297112425	0.000896547
POSITIVE REGULATION OF RECEPTOR RECYCLING	11	0.073001517	0.770374397
RESPONSE TO MISFOLDED PROTEIN	12	0.635174481	0.002918281
TRANSMEMBRANE TRANSPORT	862	0.022611068	0.432629184
REGULATION OF GENE EXPRESSION EPIGENETIC	204	-0.081100291	0.151598175
NEGATIVE REGULATION OF NEURON DEATH	157	-0.028141841	0.666062357
NEGATIVE REGULATION OF CELLULAR COMPONENT ORGANIZATION	622	-0.035633194	0.283847028
DEFENSE RESPONSE TO FUNGUS	23	-0.015504454	0.927375346
RIBONUCLEOSIDE CATABOLIC PROCESS	21	-0.080739997	0.64434175
POSITIVE REGULATION OF CHONDROCYTE DIFFERENTIATION	15	-0.084109356	0.683752893
POSITIVE REGULATION OF PHOSPHATIDYLINOSITOL 3 KINASE SIGNALING	57	-0.025735901	0.811509126
REGULATION OF FILOPODIUM ASSEMBLY	34	0.000125401	0.999287623
REGULATION OF CELL PROJECTION SIZE	9	0.069372142	0.801570352
NUCLEAR TRANSCRIBED MRNA CATABOLIC PROCESS EXONUCLEOLYTIC	31	-0.106094007	0.457760348

NEGATIVE REGULATION OF RYANODINE SENSITIVE CALCIUM RELEASE CHANNEL ACTIVITY	12	-0.112525079	0.622285562
POSITIVE REGULATION OF NITRIC OXIDE SYNTHASE ACTIVITY	17	0.064726602	0.747356663
POSITIVE REGULATION OF CELL CYCLE PHASE TRANSITION	63	-0.094573109	0.348297288
MYELOID DENDRITIC CELL ACTIVATION	23	0.052745967	0.759743548
MESONEPHROS DEVELOPMENT	82	0.017444909	0.847824342
EMBRYONIC HEART TUBE MORPHOGENESIS	54	-0.142498875	0.183047931
MESODERMAL CELL FATE COMMITMENT	11	-0.085140775	0.723926997
REGULATION OF DENDRITE DEVELOPMENT	108	-0.086708527	0.262205876
RESPONSE TO LIGHT STIMULUS	235	-0.11232843	0.031745647
UTP METABOLIC PROCESS	9	-0.441820862	0.056445721
REGULATION OF AXONOGENESIS	154	0.119048131	0.077962455
RIBOSOMAL SMALL SUBUNIT ASSEMBLY	16	-0.224282084	0.238671248
REGULATION OF ACTIVIN RECEPTOR SIGNALING PATHWAY	24	-0.036879115	0.82382694
REGULATION OF SYSTEMIC ARTERIAL BLOOD PRESSURE BY HORMONE	29	0.128365891	0.405308667
REGULATION OF NEUTROPHIL MIGRATION	30	0.333730021	0.025528
CELLULAR RESPONSE TO MECHANICAL STIMULUS	70	-0.055144423	0.568604552
PENTOSE METABOLIC PROCESS	11	-0.190991527	0.412093402
POSITIVE REGULATION OF DEFENSE RESPONSE	307	-0.077425382	0.094549
PHAGOCYTOSIS	131	0.053914058	0.457454022
BRANCHING INVOLVED IN MAMMARY GLAND DUCT MORPHOGENESIS	18	-0.215479747	0.228471149
ALKALOID METABOLIC PROCESS	13	-0.165025435	0.441730521
POSITIVE REGULATION OF OXIDATIVE STRESS INDUCED CELL DEATH	9	0.014135958	0.958789718
CENTROMERE COMPLEX ASSEMBLY	47	-0.178031173	0.115362602
POSITIVE REGULATION OF MYELOID LEUKOCYTE MEDIATED IMMUNITY	15	0.057600979	0.787201544
RRNA CONTAINING RIBONUCLEOPROTEIN COMPLEX EXPORT FROM NUCLEUS	11	0.167471086	0.514516299
MITOTIC SISTER CHROMATID COHESION	12	-0.212216037	0.337533967
KINETOCHORE ORGANIZATION	12	-0.223234103	0.305609872
NEGATIVE REGULATION OF GLUCOSE IMPORT	14	0.22659102	0.304238869
GLUCOSE CATABOLIC PROCESS	25	0.116597118	0.483552594
REGULATION OF MYOBLAST PROLIFERATION	8	0.41030087	0.162319304
ANTIGEN PROCESSING AND PRESENTATION OF ENDOGENOUS ANTIGEN	15	-0.446771329	0.012166547
REGULATION OF CAMP DEPENDENT PROTEIN KINASE ACTIVITY	13	-0.251147897	0.232252998
REGULATION OF TRANSCRIPTION INVOLVED IN G1 S TRANSITION OF MITOTIC CELL CYCLE	26	-0.176795855	0.248408876
POSITIVE REGULATION OF STRIATED MUSCLE CELL DIFFERENTIATION	45	-0.03519457	0.771369442
INORGANIC ION TRANSMEMBRANE TRANSPORT	448	0.03021124	0.443945954
POSITIVE REGULATION OF INSULIN SECRETION INVOLVED IN CELLULAR RESPONSE TO GLUCOSE STIMULUS	20	0.074801083	0.686532204
NEGATIVE REGULATION OF CELL CYCLE	401	-0.1056866	0.008915444
POSITIVE REGULATION OF CYCLIN DEPENDENT PROTEIN KINASE ACTIVITY	32	-0.240499525	0.076557285
REGULATION OF ALPHA BETA T CELL PROLIFERATION	21	0.025220965	0.888294121
AXONAL FASCICULATION	15	-0.024489876	0.907208164
INTRINSIC APOPTOTIC SIGNALING PATHWAY IN RESPONSE TO ENDOPLASMIC RETICULUM STRESS	30	-0.203344916	0.147784641
REGULATION OF CAMP METABOLIC PROCESS	94	0.03378969	0.691645344
REGULATION OF DEFENSE RESPONSE TO BACTERIUM	7	-0.0738356	0.807810172
POSITIVE REGULATION OF PATHWAY RESTRICTED SMAD PROTEIN PHOSPHORYLATION	37	0.023957119	0.859462091
FOLIC ACID METABOLIC PROCESS	17	0.006881477	0.972387121
REGIONALIZATION	267	0.048824265	0.33818001
CENTRAL NERVOUS SYSTEM DEVELOPMENT	755	0.018318388	0.550089662
RESPONSE TO WATER	15	-0.40355003	0.029879175
REGULATION OF CELL KILLING	52	0.083434265	0.468957228
REGULATION OF ORGAN MORPHOGENESIS	214	-0.058491217	0.292463404
REGULATION OF CARBOHYDRATE CATABOLIC PROCESS	38	0.018799381	0.887920598
REGULATION OF RESPIRATORY GASEOUS EXCHANGE	20	-0.240564544	0.150563852
GLYCOSYL COMPOUND BIOSYNTHETIC PROCESS	106	-0.132528356	0.084924914
CELLULAR GLUCURONIDATION	10	0.394247507	0.110128633
RESPONSE TO OXIDATIVE STRESS	319	-0.037697357	0.411586179
REGULATION OF JUN KINASE ACTIVITY	74	-0.042337137	0.653742958
CEREBELLAR PURKINJE CELL LAYER DEVELOPMENT	22	-0.129009881	0.442989551
PHOSPHOLIPID EFFLUX	10	0.696569202	0.000430853
MUCOPOLYSACCHARIDE METABOLIC PROCESS	95	-0.127503861	0.117200642
REGULATION OF PHOSPHORUS METABOLIC PROCESS	1384	-0.003793451	0.86931011
TRNA PROCESSING	104	-0.145632058	0.059271942

REGULATION OF SYNAPSE MATURATION	13	0.077601797	0.735533206
REGULATION OF VASCULAR PERMEABILITY	27	-0.074132076	0.631401429
RESPONSE TO MECHANICAL STIMULUS	175	-0.027284901	0.658970505
SUBPALLIUM DEVELOPMENT	19	-0.054015603	0.770676412
SOMATIC RECOMBINATION OF IMMUNOGLOBULIN GENE SEGMENTS	20	-0.141123482	0.426207328
B CELL RECEPTOR SIGNALING PATHWAY	28	-0.067530823	0.657216173
RECEPTOR CATABOLIC PROCESS	16	-0.018030572	0.929498625
PYRIDINE NUCLEOTIDE BIOSYNTHETIC PROCESS	15	-0.297794031	0.121561347
SIGNAL PEPTIDE PROCESSING	21	0.040812369	0.820634178
EPITHELIAL CELL FATE COMMITMENT	12	0.239253955	0.322559541
SODIUM ION TRANSPORT	105	0.124702109	0.126232542
POSITIVE REGULATION OF INTERLEUKIN 2 BIOSYNTHETIC PROCESS	12	0.058425918	0.807199786
REGULATION OF CGMP BIOSYNTHETIC PROCESS	14	0.287915661	0.183128236
APOPTOTIC MITOCHONDRIAL CHANGES	54	-0.027466	0.804250085
PROTEIN EXIT FROM ENDOPLASMIC RETICULUM	20	-0.383674132	0.027571372
T CELL APOPTOTIC PROCESS	15	0.143958212	0.504132861
RETINOIC ACID RECEPTOR SIGNALING PATHWAY	15	-0.1531253	0.450258165
REGULATION OF NECROPTOTIC PROCESS	11	-0.098058188	0.682585884
NEGATIVE REGULATION OF HEART GROWTH	11	0.011855143	0.961773615
POSITIVE REGULATION OF ASTROCYTE DIFFERENTIATION	11	0.468710154	0.056012096
SKELETAL SYSTEM MORPHOGENESIS	172	0.030291333	0.631232735
REGULATION OF MULTICELLULAR ORGANISMAL METABOLIC PROCESS	30	-0.405076779	0.001675468
LEUKOTRIENE METABOLIC PROCESS	18	0.188403868	0.334665255
POSITIVE REGULATION OF CHROMOSOME SEGREGATION	24	-0.024280829	0.883841222
RRNA METHYLATION	16	-0.046804152	0.817149799
GENE SILENCING BY RNA	134	-0.131365292	0.055604336
POSITIVE REGULATION OF TRIGLYCERIDE METABOLIC PROCESS	17	0.308601203	0.128510612
REGULATION OF ENDOTHELIAL CELL PROLIFERATION	89	-0.043929902	0.609696184
REGULATED EXOCYTOSIS	187	-0.033496818	0.574970332
CELL CELL ADHESION VIA PLASMA MEMBRANE ADHESION MOLECULES	152	0.002185066	0.973873457
POSITIVE REGULATION OF NEURON APOPTOTIC PROCESS	44	-0.049708202	0.683882316
TEMPERATURE HOMEOSTASIS	22	0.107162068	0.545178684
EMBRYONIC CAMERA TYPE EYE FORMATION	10	-0.100681343	0.690783245
PROTEIN O LINKED GLYCOSYLATION	88	0.022413975	0.798635683
PURINERGIC RECEPTOR SIGNALING PATHWAY	19	-0.027716498	0.881907692
NEGATIVE REGULATION OF RECEPTOR BINDING	11	0.141170105	0.572932917
POSITIVE REGULATION OF SYNAPTIC TRANSMISSION GABAERGIC	6	-0.201864539	0.519429423
DOPAMINE TRANSPORT	7	0.187600368	0.550534094
REGULATION OF CALCIUM ION TRANSMEMBRANE TRANSPORTER ACTIVITY	54	-0.315709534	0.001547551
CIRCULATORY SYSTEM DEVELOPMENT	709	-0.012308361	0.694996014
CELLULAR RESPONSE TO VASCULAR ENDOTHELIAL GROWTH FACTOR STIMULUS	30	0.213739661	0.159147022
POSITIVE REGULATION OF GLUCONEOGENESIS	13	0.059584679	0.795108542
REGULATION OF CELL PROLIFERATION	1297	-0.008085804	0.733217509
EMBRYONIC CAMERA TYPE EYE DEVELOPMENT	29	0.0946574	0.540040943
POSITIVE REGULATION OF EXECUTION PHASE OF APOPTOSIS	11	0.35408667	0.14807462
POSITIVE REGULATION OF DNA TEMPLATED TRANSCRIPTION ELONGATION	22	-0.169676617	0.307270603
N TERMINAL PROTEIN AMINO ACID MODIFICATION	23	-0.23559538	0.132652429
REGULATION OF TYPE I INTERFERON PRODUCTION	103	-0.044021603	0.582471459
NUCLEOTIDE EXCISION REPAIR PREINCISION COMPLEX STABILIZATION	20	0.304199383	0.099431371
EMBRYONIC PLACENTA MORPHOGENESIS	21	0.295228457	0.098874969
DOPAMINE RECEPTOR SIGNALING PATHWAY	24	0.097220852	0.567271933
REGULATION OF ERAD PATHWAY	27	-0.239491013	0.102379712
BEHAVIOR	418	0.025626896	0.529730895
CYTOSKELETON DEPENDENT INTRACELLULAR TRANSPORT	107	-0.060186614	0.441903562
MANNOSYLATION	30	-0.00416826	0.977736506
DNA REPLICATION INDEPENDENT NUCLEOSOME ORGANIZATION	52	-0.108713983	0.324379718
AXON EXTENSION	33	0.006221526	0.965235614
DERMATAN SULFATE METABOLIC PROCESS	13	-0.0337853	0.88071879
TRIVALENT INORGANIC CATION TRANSPORT	35	-0.000358298	0.997934722
POSITIVE REGULATION OF PROTEIN TARGETING TO MEMBRANE	22	-0.035956207	0.835410004
BILE ACID METABOLIC PROCESS	22	0.019720686	0.91039354
REGULATION OF SODIUM ION TRANSMEMBRANE TRANSPORT	42	0.008050107	0.949306101
INNERVATION	21	-0.171945651	0.316325251
RESPONSE TO VIRUS	208	-0.093455583	0.093878328
DEVELOPMENTAL MATURATION	158	0.055497339	0.401274398

REGULATION OF CELLULAR AMINO ACID METABOLIC PROCESS	56	-0.208380048	0.041631145
ALCOHOL BIOSYNTHETIC PROCESS	96	-0.054468237	0.509971964
REGULATION OF INSULIN RECEPTOR SIGNALING PATHWAY	37	-0.083817124	0.524603267
ADIPOSE TISSUE DEVELOPMENT	31	0.062781717	0.672983441
PYRIDINE CONTAINING COMPOUND BIOSYNTHETIC PROCESS	18	-0.35749121	0.036260425
PROTEIN TARGETING TO LYSOSOME	15	0.194547142	0.38157165
ISOPRENOID METABOLIC PROCESS	93	0.084588158	0.326613355
NEGATIVE REGULATION OF SIGNAL TRANSDUCTION IN ABSENCE OF LIGAND	30	0.128408904	0.398096837
RENAL SYSTEM PROCESS INVOLVED IN REGULATION OF SYSTEMIC ARTERIAL BLOOD PRESSURE	18	-0.020835557	0.913586356
NEGATIVE REGULATION OF PROTEIN MODIFICATION PROCESS	559	-0.023209646	0.508323433
NEGATIVE REGULATION OF B CELL APOPTOTIC PROCESS	11	-0.183398628	0.434645002
RESPONSE TO UV B	13	-0.035823259	0.873513179
CELLULAR RESPONSE TO DRUG	59	-0.050878881	0.62933823
REGULATION OF RESPONSE TO DNA DAMAGE STIMULUS	138	0.023983588	0.732985782
AUTOPHAGOSOME ORGANIZATION	41	-0.087955399	0.48196466
POSITIVE REGULATION OF BLOOD VESSEL ENDOTHELIAL CELL MIGRATION	25	0.085460007	0.607066135
PROTEIN AUTOPROCESSING	12	0.505370584	0.02286517
ERYTHROCYTE DEVELOPMENT	21	-0.274670008	0.098953728
CELLULAR RESPONSE TO DNA DAMAGE STIMULUS	664	-0.020182106	0.532528609
PHOSPHATIDYLINOSITOL ACYL CHAIN REMODELING	11	0.309279091	0.206517703
POLYOL BIOSYNTHETIC PROCESS	24	0.112299236	0.509046303
NEGATIVE REGULATION OF TUMOR NECROSIS FACTOR SUPERFAMILY CYTOKINE PRODUCTION	34	0.175984506	0.219138183
ENDODERMAL CELL FATE COMMITMENT	12	-0.36735337	0.075649684
MONOAMINE TRANSPORT	15	-0.038602731	0.853725731
RESPONSE TO STARVATION	143	-0.005385242	0.937477327
REGULATION OF GTPASE ACTIVITY	570	0.027690148	0.430364363
POSITIVE REGULATION OF CAMP MEDIATED SIGNALING	9	0.171474938	0.535786859
RESPONSE TO REDOX STATE	12	0.143858824	0.548578305
REGULATION OF GLUCONEOGENESIS	34	-0.077591877	0.5731851
POST EMBRYONIC DEVELOPMENT	82	-0.154200384	0.076299411
CELLULAR DEFENSE RESPONSE	42	0.124270024	0.333311131
BIOLOGICAL ADHESION	848	-0.004288074	0.882007824
CORTICAL CYTOSKELETON ORGANIZATION	34	-0.090255578	0.510399812
HORMONE METABOLIC PROCESS	126	0.107212055	0.148994362
RESPONSE TO PURINE CONTAINING COMPOUND	136	-0.133567644	0.049276533
G PROTEIN COUPLED RECEPTOR SIGNALING PATHWAY COUPLED TO CYCLIC NUCLEOTIDE SECOND MESSENGER	115	0.121613597	0.118408889
UTERUS DEVELOPMENT	16	0.083309746	0.687777991
THYROID GLAND DEVELOPMENT	23	0.104540209	0.548100472
DNA REPLICATION	197	-0.129440608	0.022461683
NEURAL TUBE PATTERNING	31	-0.116773526	0.412897448
RESPONSE TO HISTAMINE	9	0.005307727	0.984492102
RESPONSE TO MANGANESE ION	15	-0.207913988	0.291148446
REGULATION OF SISTER CHROMATID SEGREGATION	65	-0.133298813	0.173381196
CELL CYCLE PROCESS	1000	-0.093604361	0.000370378
UNSATURATED FATTY ACID BIOSYNTHETIC PROCESS	39	-0.088184682	0.491451207
POSITIVE REGULATION OF MUSCLE CELL APOPTOTIC PROCESS	12	-0.077495332	0.737734147
SIALYLATION	17	-0.039425227	0.841077952
REGULATION OF GENE SILENCING	50	-0.020061671	0.86194177
POSITIVE REGULATION OF AMINO ACID TRANSPORT	12	0.328739441	0.155907673
ENDOCRINE PROCESS	36	0.153563047	0.268765636
PRIMARY ALCOHOL METABOLIC PROCESS	38	0.075374779	0.575268871
PLATELET AGGREGATION	37	-0.123520776	0.341959872
TISSUE DEVELOPMENT	1280	0.036532245	0.128871092
RNA INTERFERENCE	12	-0.259424401	0.230931774
POSITIVE REGULATION OF ESTABLISHMENT OF PROTEIN LOCALIZATION	434	-0.007323575	0.853943828
WALKING BEHAVIOR	27	0.184222895	0.249841092
REGULATION OF TELOMERASE ACTIVITY	40	0.1356223	0.304637405
REGULATION OF CELL AGING	33	-0.130884026	0.342550502
CELL GROWTH	120	0.06036029	0.426098222
COFACTOR BIOSYNTHETIC PROCESS	146	-0.130195497	0.04737348
LYSINE METABOLIC PROCESS	12	0.454489998	0.039693693
NEGATIVE REGULATION OF CHONDROCYTE DIFFERENTIATION	15	-0.33840591	0.068372543
POSITIVE REGULATION OF AXONOGENESIS	68	0.089338002	0.37645816

TRANSMEMBRANE RECEPTOR PROTEIN SERINE THREONINE KINASE SIGNALING PATHWAY	167	0.02074339	0.745559662
CHAPERONE MEDIATED PROTEIN FOLDING	43	-0.044933719	0.716243115
POSITIVE REGULATION OF STEROID METABOLIC PROCESS	19	0.345711597	0.058222821
REGULATION OF LYMPHOCYTE MIGRATION	34	-0.20382542	0.126486274
BONE MATURATION	14	-0.001919155	0.992996432
CELLULAR RESPONSE TO REACTIVE OXYGEN SPECIES	93	-0.175351847	0.030358922
NEGATIVE REGULATION OF CELL COMMUNICATION	1052	-0.012494283	0.631969646
HEMOSTASIS	270	-0.053620898	0.279817529
ZYMOGEN ACTIVATION	99	-0.066090541	0.415681814
AZOLE TRANSPORT	15	-0.354625519	0.07006369
CELLULAR MACROMOLECULAR COMPLEX ASSEMBLY	656	-0.074244955	0.020861152
NEGATIVE REGULATION OF CYTOSOLIC CALCIUM ION CONCENTRATION	7	-0.174033758	0.549948624
POSITIVE REGULATION OF PHOSPHATASE ACTIVITY	26	0.096453166	0.554020363
LYMPHOCYTE DIFFERENTIATION	176	0.02445466	0.694813791
REGULATION OF POTASSIUM ION TRANSPORT	67	-0.036863042	0.710516832
CELLULAR RESPONSE TO OXIDATIVE STRESS	169	-0.125851957	0.040021137
NEURAL CREST CELL DIFFERENTIATION	68	0.179638804	0.076286675
ACTIVATION OF ANAPHASE PROMOTING COMPLEX ACTIVITY	12	0.11470533	0.632879527
CALCIUM ION IMPORT INTO CYTOSOL	38	-0.128189074	0.316855673
MICROTUBULE POLYMERIZATION	23	0.174244461	0.318955903
POSITIVE REGULATION OF MYELINATION	9	0.412730895	0.120600894
SPLICEOSOMAL SNRNP ASSEMBLY	35	-0.229918332	0.072880351
OLIGOSACCHARIDE LIPID INTERMEDIATE BIOSYNTHETIC PROCESS	19	-0.161239575	0.368647781
MONOCYTE DIFFERENTIATION	15	-0.227093815	0.244137004
REGULATION OF GONADOTROPIN SECRETION	7	0.55552396	0.03073389
RESPONSE TO LAMINAR FLUID SHEAR STRESS	13	-0.133692609	0.545268578
RESPONSE TO LEAD ION	18	0.044098458	0.820643516
DEFENSE RESPONSE TO BACTERIUM	140	0.029482892	0.673028062
REGULATION OF RELEASE OF SEQUESTERED CALCIUM ION INTO CYTOSOL BY SARCOPLASMIC RETICULUM	22	-0.331711924	0.030038936
NUCLEOTIDE SALVAGE	14	0.003911687	0.985743582
ALCOHOL CATABOLIC PROCESS	50	0.053394628	0.648192845
MYELOID DENDRITIC CELL DIFFERENTIATION	20	0.089151663	0.631872753
CELLULAR METABOLIC COMPOUND SALVAGE	36	-0.199144135	0.120838959
COPULATION	12	0.363144879	0.117027737
REGULATION OF CATION TRANSMEMBRANE TRANSPORT	169	-0.153218494	0.011679883
REGULATION OF VIRAL RELEASE FROM HOST CELL	30	0.2659797	0.084661103
NEPHRIC DUCT MORPHOGENESIS	11	-0.038679136	0.874267502
POSITIVE REGULATION OF NF KAPPAB IMPORT INTO NUCLEUS	23	0.18767814	0.279618095
POSITIVE REGULATION OF NEURON PROJECTION DEVELOPMENT	213	-0.036100121	0.519158393
ESTROGEN METABOLIC PROCESS	21	0.09017923	0.618856438
IRON ION HOMEOSTASIS	59	0.112395449	0.301306545
PROTEOLYSIS	967	-0.039743449	0.140771923
SYNAPSIS	28	0.432367585	0.00378207
ORGANELLE DISASSEMBLY	161	-0.11267682	0.07322188
REGULATION OF ER TO GOLGI VESICLE MEDIATED TRANSPORT	11	0.200603981	0.433526595
RESPONSE TO IRON ION	31	-0.11015325	0.441123405
PHOSPHATIDYLINOSITOL 3 KINASE SIGNALING	23	0.051810071	0.76368964
LOCOMOTORY EXPLORATION BEHAVIOR	9	-0.054905654	0.838266177
PYRIMIDINE CONTAINING COMPOUND BIOSYNTHETIC PROCESS	38	-0.223285614	0.071859626
NUCLEAR CHROMOSOME SEGREGATION	206	-0.110474721	0.047738286
AUDITORY RECEPTOR CELL MORPHOGENESIS	10	-0.322564384	0.172664834
REGULATION OF ODONTOGENESIS OF DENTIN CONTAINING TOOTH	7	0.010348569	0.973363926
REGULATION OF WNT SIGNALING PATHWAY PLANAR CELL POLARITY PATHWAY	10	0.361934495	0.156004113
NEUROTROPHIN SIGNALING PATHWAY	21	-0.395882272	0.011185243
CELLULAR RESPONSE TO INTERFERON BETA	15	-0.02405122	0.90887948
MITOCHONDRIAL ELECTRON TRANSPORT CYTOCHROME C TO OXYGEN	14	-0.477456307	0.005401061
NEGATIVE REGULATION OF VIRAL TRANSCRIPTION	24	0.30663568	0.064779223
FILOPODIUM ASSEMBLY	14	-0.156319195	0.45550528
RESPONSE TO TOPOLOGICALLY INCORRECT PROTEIN	151	-0.077678365	0.236973068
STABILIZATION OF MEMBRANE POTENTIAL	10	0.237973563	0.363740759
ERK1 AND ERK2 CASCADE	18	0.117647352	0.548622149
LENS FIBER CELL DEVELOPMENT	9	-0.072175662	0.787301149
SYNCYTIUM FORMATION	21	0.194030463	0.294398521

POSITIVE REGULATION OF MESONEPHROS DEVELOPMENT	19	-0.130381488	0.471896504
GLYCOSIDE METABOLIC PROCESS	13	0.15957431	0.490791622
HYDROGEN ION TRANSMEMBRANE TRANSPORT	85	-0.146915604	0.085286571
HETEROTYPIC CELL CELL ADHESION	24	0.045547468	0.78705063
NEGATIVE REGULATION OF HOMOTYPIC CELL CELL ADHESION	89	0.017616048	0.840056392
REGULATION OF SMOOTH MUSCLE CELL APOPTOTIC PROCESS	14	-0.175870701	0.394729657
PROSTANOID BIOSYNTHETIC PROCESS	17	-0.160437638	0.395544045
POSITIVE REGULATION OF ERAD PATHWAY	13	0.166234229	0.473192605
CENTROSOME LOCALIZATION	18	0.050070722	0.797208588
ASTROCYTE DEVELOPMENT	18	0.101982212	0.603407404
RESPONSE TO MONOAMINE	32	0.276416004	0.060566554
TYROSINE PHOSPHORYLATION OF STAT PROTEIN	9	0.099434357	0.720035525
METAPHASE PLATE CONGRESSION	40	0.126327263	0.339032408
POSITIVE REGULATION OF MYELOID LEUKOCYTE DIFFERENTIATION	43	0.032897989	0.793605899
POSITIVE REGULATION OF APOPTOTIC SIGNALING PATHWAY	157	-0.044251891	0.49574991
POSITIVE REGULATION OF NFAT PROTEIN IMPORT INTO NUCLEUS	10	0.053645083	0.837354252
CELLULAR RESPONSE TO ABIOTIC STIMULUS	224	-0.120489295	0.023964272
RESPONSE TO HYDROGEN PEROXIDE	101	-0.056585904	0.4826206
REGULATION OF DEVELOPMENTAL PIGMENTATION	16	-0.09364069	0.639141138
EXTRACELLULAR FIBRIL ORGANIZATION	12	0.338278803	0.144604489
NEGATIVE REGULATION OF STRIATED MUSCLE CELL APOPTOTIC PROCESS	15	0.118058876	0.583399986
RIBONUCLEOSIDE DIPHOSPHATE METABOLIC PROCESS	59	0.061950947	0.565883323
POSITIVE REGULATION OF ANION TRANSPORT	45	0.148087353	0.232536179
INNER EAR RECEPTOR CELL DEVELOPMENT	28	-0.111838359	0.456382175
REGULATION OF MONOCYTE CHEMOTAXIS	20	0.031380465	0.864724389
PEPTIDE TRANSPORT	51	0.038074068	0.74184858
T CELL SELECTION	29	0.092231789	0.551267784
NEGATIVE CHEMOTAXIS	37	0.326109751	0.016607954
PHAGOCYTOSIS RECOGNITION	11	-0.122254526	0.607762129
MISFOLDED OR INCOMPLETELY SYNTHESIZED PROTEIN CATABOLIC PROCESS	14	0.48222429	0.029634023
RESPONSE TO MOLECULE OF BACTERIAL ORIGIN	277	0.029755824	0.550956194
POST EMBRYONIC ORGAN DEVELOPMENT	13	0.118830495	0.606638395
DEVELOPMENTAL GROWTH	292	0.046983766	0.335457293
REGULATION OF PROTEIN TARGETING	268	-0.036026822	0.471323357
THYROID HORMONE GENERATION	10	0.097130443	0.711364813
REGULATION OF SYSTEMIC ARTERIAL BLOOD PRESSURE MEDIATED BY A CHEMICAL SIGNAL	37	0.08717682	0.522768386
CENTRAL NERVOUS SYSTEM NEURON AXONOGENESIS	24	-0.002612769	0.987518661
V D J RECOMBINATION	14	0.298548081	0.181023257
METANEPHROS MORPHOGENESIS	23	0.018732636	0.912951513
GLYCOPROTEIN METABOLIC PROCESS	304	-0.053914568	0.249554302
POSITIVE REGULATION OF GLUCOSE IMPORT IN RESPONSE TO INSULIN STIMULUS	11	-0.011103795	0.964017686
SINGLE ORGANISM CELLULAR LOCALIZATION	815	-0.029478743	0.314186182
RETINA MORPHOGENESIS IN CAMERA TYPE EYE	36	0.007107859	0.958534017
ANTIGEN PROCESSING AND PRESENTATION OF ENDOGENOUS PEPTIDE ANTIGEN	13	-0.521151019	0.004767047
HISTONE H3 DEACETYLATION	20	0.114339509	0.540825493
REGULATION OF SYNAPTIC VESICLE TRANSPORT	25	0.116703744	0.482966251
POSITIVE REGULATION OF CARDIAC MUSCLE CELL DIFFERENTIATION	13	0.053648564	0.815015754
REGULATION OF DNA TEMPLATED TRANSCRIPTION ELONGATION	40	-0.030023811	0.81542237
LENS DEVELOPMENT IN CAMERA TYPE EYE	56	-0.111433221	0.293776526
NEGATIVE REGULATION OF CATABOLIC PROCESS	190	0.005364616	0.928506439
REGULATION OF OXIDATIVE STRESS INDUCED INTRINSIC APOPTOTIC SIGNALING PATHWAY	25	0.138769575	0.406069283
POSITIVE REGULATION OF MACROPHAGE DIFFERENTIATION	11	-0.181824833	0.437234227
REGULATION OF RESPONSE TO REACTIVE OXYGEN SPECIES	34	-0.108652707	0.425779642
NEGATIVE REGULATION OF INTRACELLULAR TRANSPORT	127	-0.103239906	0.145918554
VASCULAR PROCESS IN CIRCULATORY SYSTEM	138	0.062333734	0.378048672
MULTI ORGANISM BEHAVIOR	64	0.001071288	0.991660562
REGULATION OF BICELLULAR TIGHT JUNCTION ASSEMBLY	12	0.338151583	0.15615756
DNA METABOLIC PROCESS	676	-0.036394998	0.254689867
INTESTINAL ABSORPTION	22	0.412095478	0.019149329
MALE SEX DIFFERENTIATION	118	0.114453264	0.136958025
REGULATION OF SKELETAL MUSCLE CELL PROLIFERATION	10	-0.272203911	0.246425478
NEGATIVE REGULATION OF KERATINOCYTE PROLIFERATION	12	-0.265069747	0.216637818

LAMELLIPODIUM ASSEMBLY	30	-0.237750051	0.086122502
RESPONSE TO WATER DEPRIVATION	8	-0.601750033	0.011510548
MITOTIC CHROMOSOME CONDENSATION	14	-0.344820457	0.073431412
ANATOMICAL STRUCTURE HOMEOSTASIS	238	0.017219594	0.748174767
REGULATION OF TRANSMEMBRANE TRANSPORT	330	0.015269393	0.738132351
REGULATION OF EPITHELIAL TO MESENCHYMAL TRANSITION	61	0.150754076	0.159953727
DNA ALKYLATION	36	0.079514551	0.565575562
POSITIVE REGULATION OF TRANSCRIPTION FROM RNA POLYMERASE II PROMOTER IN RESPONSE TO ENDOPLASMIC RETICULUM STRESS	12	-0.204039468	0.357788565
RENAL SYSTEM PROCESS	81	-0.118858097	0.177709371
LEUKOCYTE HOMEOSTASIS	51	-0.121593039	0.274252459
POSITIVE REGULATION OF ORGANELLE ASSEMBLY	44	-0.138612442	0.243944357
LUNG ALVEOLUS DEVELOPMENT	37	-0.020326639	0.879472277
PROGESTERONE METABOLIC PROCESS	10	-0.005239981	0.98382554
SPLEEN DEVELOPMENT	35	0.09147852	0.516380806
RHO PROTEIN SIGNAL TRANSDUCTION	44	-0.003659253	0.976344133
RESPONSE TO LEPTIN	19	-0.059468367	0.747782453
NEUROPEPTIDE SIGNALING PATHWAY	58	0.129404245	0.236691779
ACTIVATION OF PROTEIN KINASE A ACTIVITY	14	-0.165025374	0.427321534
POSITIVE REGULATION OF VASCULAR ENDOTHELIAL GROWTH FACTOR PRODUCTION	25	0.085219806	0.608282825
AMP METABOLIC PROCESS	14	0.047417327	0.829946426
DEVELOPMENT OF PRIMARY SEXUAL CHARACTERISTICS	172	0.037708158	0.550703734
NEGATIVE REGULATION OF OSTEOCLAST DIFFERENTIATION	21	-0.271413608	0.094092081
REGULATION OF RNA SPLICING	92	0.008622479	0.91986089
REGULATION OF BLOOD CIRCULATION	243	0.085024334	0.113151184
NEGATIVE REGULATION OF MITOCHONDRION ORGANIZATION	38	-0.231364713	0.060434701
NEGATIVE REGULATION OF SUBSTRATE ADHESION DEPENDENT CELL SPREADING	13	0.143325189	0.536989437
EMBRYONIC CRANIAL SKELETON MORPHOGENESIS	43	0.040441299	0.748065399
CELLULAR PROTEIN COMPLEX LOCALIZATION	20	0.405898673	0.036353638
CELLULAR RESPONSE TO GONADOTROPIN STIMULUS	11	0.550398318	0.01067744
REGULATION OF HISTONE H3 K9 ACETYLATION	14	0.106441667	0.631908994
INTRACELLULAR ESTROGEN RECEPTOR SIGNALING PATHWAY	17	0.132723534	0.512823691
NEGATIVE REGULATION OF DNA DAMAGE RESPONSE SIGNAL TRANSDUCTION BY P53 CLASS MEDIATOR	13	0.037516986	0.869689485
CARDIAC CELL DEVELOPMENT	41	-0.008028233	0.949872815
REGULATION OF EXECUTION PHASE OF APOPTOSIS	22	0.159188729	0.373593376
RESPONSE TO CALCIUM ION	100	-0.045074199	0.578883098
NEGATIVE REGULATION OF FATTY ACID METABOLIC PROCESS	22	0.154984533	0.382040607
REGULATION OF INTERLEUKIN 12 PRODUCTION	41	-0.050399107	0.689965478
POSITIVE REGULATION OF ALPHA BETA T CELL ACTIVATION	45	0.028401148	0.817146376
REGULATION OF TRANSCRIPTION INITIATION FROM RNA POLYMERASE II PROMOTER	20	-0.176643791	0.30930255
POSITIVE REGULATION OF CYTOSOLIC CALCIUM ION CONCENTRATION INVOLVED IN PHOSPHOLIPASE C ACTIVATING G PROTEIN COUPLED SIGNALING PATHWAY	11	0.034317075	0.890116056
INNER EAR MORPHOGENESIS	72	-0.025630821	0.789644466
RESPONSE TO ACTIVITY	59	0.055146692	0.609014012
ESTROUS CYCLE	17	-0.119098285	0.534776237
ORGANELLE FUSION	111	0.021833664	0.780312924
REGULATION OF PROTEIN PHOSPHATASE TYPE 2A ACTIVITY	23	-0.160367242	0.32846878
STEROID CATABOLIC PROCESS	21	0.056831541	0.752790094
REGULATION OF CELL FATE COMMITMENT	21	0.270801975	0.133828617
REGULATION OF CELL MORPHOGENESIS	500	0.046228369	0.218058875
DNA DAMAGE RESPONSE SIGNAL TRANSDUCTION RESULTING IN TRANSCRIPTION	15	-0.273570619	0.152561684
ACUTE PHASE RESPONSE	32	-0.008038621	0.955637791
POSITIVE REGULATION OF INSULIN LIKE GROWTH FACTOR RECEPTOR SIGNALING PATHWAY	10	0.015453239	0.952533256
NEGATIVE REGULATION OF PROTEIN SERINE THREONINE KINASE ACTIVITY	116	-0.188514112	0.009331752
S ADENOSYLHOMOCYSTEINE METABOLIC PROCESS	12	-0.199384271	0.366195765
CELLULAR RESPONSE TO ALKALOID	31	0.035084666	0.812678238
NEGATIVE REGULATION OF PEPTIDASE ACTIVITY	187	0.016101714	0.789759139
MYOBLAST FUSION	15	0.032754317	0.877653054
TRNA MODIFICATION	54	-0.133731463	0.213017319

CYTOSOLIC TRANSPORT	190	-0.018317836	0.75812612
INOSITOL LIPID MEDIATED SIGNALING	111	0.002840234	0.970944643
POSITIVE REGULATION OF ACTIN FILAMENT DEPOLYMERIZATION	12	0.148467273	0.537355289
DNA CATABOLIC PROCESS ENDONUCLEOLYTIC	16	0.011125973	0.956737984
POSITIVE REGULATION OF GLIOGENESIS	40	0.074844209	0.569007976
LACTATION	36	-0.320895626	0.00984229
NEGATIVE REGULATION OF RESPONSE TO EXTERNAL STIMULUS	235	0.065838987	0.226248788
REGULATION OF NUCLEAR TRANSCRIBED MRNA CATABOLIC PROCESS DEADENYLATION DEPENDENT DECAY	14	0.195565546	0.381633198
REGULATION OF NEUTROPHIL CHEMOTAXIS	25	0.277838282	0.092692814
MRNA TRANSCRIPTION FROM RNA POLYMERASE II PROMOTER	13	-0.354322542	0.075742248
EMBRYO IMPLANTATION	32	0.073382014	0.616667939
RECEPTOR CLUSTERING	35	-0.003192862	0.981586113
PIGMENT ACCUMULATION	12	0.210848612	0.376316674
FATTY ACID ELONGATION	11	0.0886688	0.723218637
CYTOSKELETON DEPENDENT CYTOKINESIS	36	-0.216701358	0.089473773
QUINONE METABOLIC PROCESS	25	0.020462654	0.900956657
FLUID TRANSPORT	17	-0.031145629	0.874395744
SYNAPSE ASSEMBLY	59	-0.037615786	0.72205936
PIGMENT METABOLIC PROCESS	50	-0.158905934	0.150679094
IMMUNE EFFECTOR PROCESS	369	-0.006237711	0.884817635
ODONTOGENESIS	89	0.04747591	0.588493137
CORPUS CALLOSUM DEVELOPMENT	9	-0.185256938	0.469862617
REGULATION OF SYNAPTIC TRANSMISSION DOPAMINERGIC	13	0.043746116	0.848405392
POSITIVE REGULATION OF CELL MORPHOGENESIS INVOLVED IN DIFFERENTIATION	152	0.007163357	0.914586775
POSITIVE REGULATION OF SODIUM ION TRANSMEMBRANE TRANSPORTER ACTIVITY	11	-0.172899541	0.465261369
NUCLEOBASE BIOSYNTHETIC PROCESS	17	-0.17469875	0.353241505
NEGATIVE REGULATION OF LYMPHOCYTE DIFFERENTIATION	34	-0.068424618	0.619904863
GOLGI ORGANIZATION	82	0.035525997	0.696968614
POSITIVE REGULATION OF CELL PROJECTION ORGANIZATION	276	-0.020353478	0.680809854
REGULATION OF OSSIFICATION	160	-0.033660359	0.601873638
EXTRACELLULAR STRUCTURE ORGANIZATION	258	0.010566619	0.837374407
DIVALENT INORGANIC CATION TRANSPORT	221	0.036165881	0.517088624
B CELL ACTIVATION INVOLVED IN IMMUNE RESPONSE	33	-0.014008507	0.921480196
REGULATION OF TRANSCRIPTION FROM RNA POLYMERASE II PROMOTER	1559	0.009938359	0.650197657
NEGATIVE REGULATION OF NUCLEOTIDE METABOLIC PROCESS	52	0.038311145	0.73787931
PROTEIN HETEROOLIGOMERIZATION	103	-0.058100133	0.466202981
REGULATION OF APOPTOTIC SIGNALING PATHWAY	335	-0.049331527	0.26975725
SIGNAL TRANSDUCTION INVOLVED IN REGULATION OF GENE EXPRESSION	15	0.597062127	0.001183742
ACTIVATION OF MAPKK ACTIVITY	49	-0.142598725	0.207721375
GLANDULAR EPITHELIAL CELL DIFFERENTIATION	33	0.204943029	0.159979884
POSITIVE REGULATION OF PROTEIN MODIFICATION BY SMALL PROTEIN CONJUGATION OR REMOVAL	179	-0.032037144	0.599819185
NEGATIVE REGULATION OF CELL PROLIFERATION	566	-0.010002339	0.774895716
REGULATION OF T CELL MIGRATION	22	-0.214661073	0.192758995
POSITIVE REGULATION OF LEUKOCYTE MEDIATED IMMUNITY	72	-0.078705277	0.405807309
CAMP MEDIATED SIGNALING	33	-0.115755744	0.400895392
POSITIVE REGULATION OF GLUCOSE METABOLIC PROCESS	33	-0.056327918	0.688640166
REGULATION OF PRODUCTION OF MOLECULAR MEDIATOR OF IMMUNE RESPONSE	83	0.071197885	0.434886662
PROTEIN TARGETING TO MITOCHONDRION	47	0.012441664	0.917297776
POSITIVE REGULATION OF OSSIFICATION	75	-0.085252815	0.357792446
RESPONSE TO REACTIVE OXYGEN SPECIES	171	-0.079749053	0.196322311
REGULATION OF VASCULATURE DEVELOPMENT	214	0.012397772	0.826203651
DETECTION OF CALCIUM ION	12	-0.068381351	0.768548579
RNA PHOSPHODIESTER BOND HYDROLYSIS ENDONUCLEOLYTIC	53	0.055386502	0.626217106
FRUCTOSE METABOLIC PROCESS	12	0.056375432	0.813286668
EYE MORPHOGENESIS	115	0.007075526	0.926488268
NEGATIVE REGULATION OF INTERLEUKIN 2 PRODUCTION	14	-0.074894698	0.727435939
POSITIVE CHEMOTAXIS	33	-0.020000323	0.887915459
NEGATIVE REGULATION OF MRNA SPLICING VIA SPLICEOSOME	18	-0.092792697	0.623068951
RESPONSE TO NITRIC OXIDE	16	-0.201704286	0.296005397
HEAD MORPHOGENESIS	31	0.017688587	0.904623584
REGULATION OF FATTY ACID BETA OXIDATION	16	0.201519702	0.331879546

REGULATION OF STEM CELL DIFFERENTIATION	102	0.159382232	0.05479202
BRAIN MORPHOGENESIS	28	-0.06322224	0.677931692
AMINE BIOSYNTHETIC PROCESS	16	-0.189009834	0.324482603

Table S3. miRNA GO cellular compartment.

GO Term	Genes Tested	Model Coefficient	p-value
FILOPODIUM	84	-0.222707517	0.00838746
NUCLEAR CHROMOSOME TELOMERIC REGION	127	0.006482312	0.929237192
CLATHRIN SCULPTED VESICLE	10	-0.214481703	0.373653491
I BAND	101	-0.018575293	0.819342727
AGGRESOME	31	0.095352418	0.523559116
MULTIVESICULAR BODY	31	0.04796439	0.746575375
RNA CAP BINDING COMPLEX	12	-0.286765339	0.177951616
LOW DENSITY LIPOPROTEIN PARTICLE	10	0.5703163	0.014005636
EXTRACELLULAR MATRIX	327	0.099146105	0.032922821
PHAGOCYTIC VESICLE	78	0.06758257	0.472387857
ENDOLYSOSOME	14	-0.016305434	0.940354479
90S PRERIBOSOME	23	-0.102218288	0.537851558
CYTOCHROME COMPLEX	17	-0.432078086	0.008849098
CHROMOSOME	813	-0.034575047	0.237874805
CONDENSED NUCLEAR CHROMOSOME	73	-0.070282839	0.456366848
NUCLEAR ENVELOPE	381	-0.140212734	0.000643603
RETROMER COMPLEX	20	0.249413409	0.19563816
PROTEASOME COMPLEX	73	-0.133574981	0.149890729
NUCLEAR EXOSOME RNASE COMPLEX	15	-0.188890813	0.344579886
ER TO GOLGI TRANSPORT VESICLE	65	-0.335135065	0.00022396
TRANSCRIPTION FACTOR TFIID COMPLEX	23	-0.012173736	0.942992
CENTRIOLE	93	-0.077671805	0.352143459
MICROTUBULE CYTOSKELETON	951	-0.041359601	0.128114283
LYSOSOMAL LUMEN	79	-0.100774048	0.261646576
PRE AUTOPHAGOSOMAL STRUCTURE	29	0.138816037	0.372567124
CELL CELL CONTACT ZONE	58	-0.345241032	0.000304234
MUSCLE MYOSIN COMPLEX	13	0.276121385	0.218773802
VOLTAGE GATED CALCIUM CHANNEL COMPLEX	31	0.286731857	0.053245027
INFLAMMASOME COMPLEX	7	-0.028190645	0.926878375
IMMUNOLOGICAL SYNAPSE	28	-0.035682662	0.816123474
GABA RECEPTOR COMPLEX	13	0.138980231	0.546214171
ENDOPLASMIC RETICULUM TUBULAR NETWORK	12	0.08244124	0.731182963
FILOPODIUM MEMBRANE	17	-0.271581128	0.136502196
PHOSPHATASE COMPLEX	44	-0.030519612	0.803487693
TRANSCRIPTION EXPORT COMPLEX	13	-0.453983673	0.012463189
SPERM PART	100	0.162324316	0.053522101
U1 SNRNP	17	-0.462082347	0.006052632
ANCHORED COMPONENT OF EXTERNAL SIDE OF PLASMA MEMBRANE	10	-0.164515562	0.507629659
NUCLEOID	43	0.003547591	0.977362397
KINESIN COMPLEX	44	-0.173515262	0.140025747
HOLO TFIIH COMPLEX	11	0.075453006	0.762435037
BASOLATERAL PLASMA MEMBRANE	167	0.006404441	0.91996486
NUCLEAR OUTER MEMBRANE	18	-0.084905836	0.652826167
CATION TRANSPORTING ATPASE COMPLEX	13	0.102275655	0.657459247
CYTOPLASMIC STRESS GRANULE	30	-0.205366326	0.15339435
SECRETORY GRANULE	278	0.064741367	0.196207406
INTRINSIC COMPONENT OF MITOCHONDRIAL OUTER MEMBRANE	20	-0.109339186	0.538004339
OXIDOREDUCTASE COMPLEX	85	-0.13936077	0.103096836
VESICLE MEMBRANE	445	0.032902404	0.406253427
SAGA COMPLEX	8	0.105722303	0.718991108
CYTOPLASMIC UBIQUITIN LIGASE COMPLEX	12	-0.376462737	0.064577428
AUTOPHAGOSOME	71	-0.047230866	0.623558055
LARGE RIBOSOMAL SUBUNIT	90	-0.295721248	0.000150864
H4 HISTONE ACETYLTRANSFERASE COMPLEX	18	-0.359851971	0.037288323
ZYMOGEN GRANULE	12	-0.018572723	0.937075109
TRANS GOLGI NETWORK TRANSPORT VESICLE MEMBRANE	12	0.15989427	0.508126945
ENDOSOME LUMEN	20	0.013201973	0.942657797
INTERCALATED DISC	46	-0.295595011	0.007044394
LATE ENDOSOME MEMBRANE	97	0.011523707	0.890271107

EXCITATORY SYNAPSE	176	-0.068452548	0.261969893
TRANSFERASE COMPLEX TRANSFERRING PHOSPHORUS CONTAINING GROUPS	227	-0.130794111	0.013460542
CHROMOSOME CENTROMERIC REGION	167	-0.180795226	0.002769079
DNA POLYMERASE COMPLEX	13	-0.089375637	0.68676271
PLATELET ALPHA GRANULE	64	-0.101642879	0.307622668
TELOMERASE HOLOENZYME COMPLEX	18	-0.425804854	0.008372328
INTRINSIC COMPONENT OF ORGANELLE MEMBRANE	249	-0.145438331	0.00381883
ADA2 GCN5 ADA3 TRANSCRIPTION ACTIVATOR COMPLEX	15	0.355694221	0.081339664
SARCOPLASMIC RETICULUM MEMBRANE	29	-0.03860004	0.797789598
U2 TYPE SPLICEOSOMAL COMPLEX	30	-0.416506438	0.001032299
PLASMA MEMBRANE RECEPTOR COMPLEX	136	-0.040874878	0.558164485
ACROSOMAL MEMBRANE	14	0.537599563	0.005730613
MICROBODY MEMBRANE	52	0.01073153	0.924930767
BASAL LAMINA	17	0.060833227	0.76192336
COPI VESICLE COAT	13	-0.305716105	0.14459162
CYTOPLASMIC SIDE OF MEMBRANE	150	-0.03793242	0.568670095
PLATELET ALPHA GRANULE MEMBRANE	13	-0.1614868	0.453907912
NEURON PROJECTION TERMINUS	112	0.088738227	0.260086342
CYTOPLASMIC REGION	256	-0.059676405	0.240552637
PROTEASOME REGULATORY PARTICLE BASE SUBCOMPLEX	10	-0.610367584	0.001217673
SECRETORY GRANULE LUMEN	65	0.025479877	0.803118741
RIBOSOME	214	-0.181518861	0.000703086
MHC PROTEIN COMPLEX	22	-0.280789449	0.075527108
ACTIN CYTOSKELETON	389	-0.037575285	0.367261904
GLYCOPROTEIN COMPLEX	18	-0.12900942	0.489768873
COATED PIT	65	0.172474429	0.097903138
EXOCYTIC VESICLE	125	0.165561376	0.027206906
RNA POLYMERASE COMPLEX	119	-0.092123517	0.210274815
MRNA CLEAVAGE FACTOR COMPLEX	17	0.27037301	0.191427653
PERINUCLEAR REGION OF CYTOPLASM	574	0.005985357	0.863650003
NUCLEOLAR PART	58	-0.101551294	0.33270143
RNA POLYMERASE II TRANSCRIPTION FACTOR COMPLEX	94	-0.119032619	0.14617303
INO80 TYPE COMPLEX	21	-0.063577981	0.717567394
CELL CORTEX PART	107	-0.097796746	0.206040145
SMN COMPLEX	13	0.066340851	0.772589982
GOLGI STACK	114	-0.092180634	0.220284887
SCF UBIQUITIN LIGASE COMPLEX	32	-0.186593069	0.170737235
CD40 RECEPTOR COMPLEX	10	-0.290635459	0.208901473
BLOC 1 COMPLEX	15	0.018801591	0.929375106
T CELL RECEPTOR COMPLEX	14	0.061589809	0.780618994
CYTOSOLIC PART	197	-0.157878007	0.004947233
VACUOLE	1036	-0.050665856	0.052041183
SECRETORY VESICLE	372	0.094817503	0.029688958
NUCLEAR SPECK	187	-0.126257408	0.030514065
NUCLEAR TRANSCRIPTIONAL REPRESSOR COMPLEX	21	-0.371584827	0.022258487
NUCLEAR CYCLIN DEPENDENT PROTEIN KINASE HOLOENZYME COMPLEX	15	-0.3678848	0.054285912
SITE OF POLARIZED GROWTH	133	-0.076326519	0.275062572
ENDOCYTIC VESICLE	220	0.133821682	0.018301645
CHROMOSOMAL REGION	314	-0.121890441	0.007073206
INCLUSION BODY	65	-0.020750757	0.837553051
MITOTIC SPINDLE	50	-0.178651887	0.104669402
ANAPHASE PROMOTING COMPLEX	21	-0.125532776	0.466830678
NUCLEAR INCLUSION BODY	12	-0.484492804	0.016736234
CELL PROJECTION CYTOPLASM	48	0.025163702	0.832358129
PROTON TRANSPORTING TWO SECTOR ATPASE COMPLEX PROTON TRANSPORTING DOMAIN	19	-0.141259368	0.432267778
MALE GERM CELL NUCLEUS	10	0.441366172	0.122037536
MYOFILAMENT	19	0.067017805	0.72412173
GEMINI OF COILED BODIES	11	0.054646533	0.826439517
CELL PROJECTION MEMBRANE	240	-0.047700035	0.365011556
APICAL PART OF CELL	297	-0.008341062	0.861633861
INTRINSIC COMPONENT OF ENDOPLASMIC RETICULUM MEMBRANE	123	-0.228798177	0.000943116
SPINDLE POLE	115	-0.06249149	0.407412919
FANCONI ANAEMIA NUCLEAR COMPLEX	13	-0.01023132	0.963963995
SEX CHROMOSOME	25	-0.340117713	0.019252816
MRNA CLEAVAGE AND POLYADENYLATION SPECIFICITY FACTOR COMPLEX	12	0.478215403	0.056758945

NEURON PART	1069	0.021260693	0.414536834
CAJAL BODY	50	-0.180967172	0.101139268
ROUGH ENDOPLASMIC RETICULUM	61	-0.103312617	0.312253456
TRANSFERASE COMPLEX	660	-0.081277177	0.011099675
VACUOLAR PROTON TRANSPORTING V TYPE ATPASE COMPLEX	13	-0.07248603	0.744919469
APICAL JUNCTION COMPLEX	107	-0.048977593	0.532367843
MICROBODY	114	-0.003162943	0.967172127
GERM CELL NUCLEUS	13	-0.018359658	0.935292933
INTRINSIC COMPONENT OF PLASMA MEMBRANE	1217	0.048142295	0.050930475
PORE COMPLEX	14	0.222245664	0.314610644
LUMENAL SIDE OF MEMBRANE	27	-0.293221946	0.039153672
GOLGI ASSOCIATED VESICLE MEMBRANE	45	-0.13967811	0.236048522
CYTOPLASMIC EXOSOME RNASE COMPLEX	15	-0.150829824	0.454468
LIPID PARTICLE	55	0.038013533	0.732814028
INTRINSIC COMPONENT OF PEROXISOMAL MEMBRANE	11	0.235853019	0.343777837
VESICLE COAT	43	-0.092563423	0.448619149
POSTSYNAPSE	319	-0.063124241	0.166532091
PRECATALYTIC SPLICEOSOME	21	-0.213831611	0.195553169
AXON	359	0.069562283	0.116000095
CLATHRIN COAT	43	0.077549423	0.540509275
BASAL PLASMA MEMBRANE	28	0.058974559	0.706206166
MCM COMPLEX	11	0.023868611	0.923299941
NUCLEAR BODY	331	-0.133539524	0.002426439
AMPA GLUTAMATE RECEPTOR COMPLEX	23	0.127037648	0.464577444
CLATHRIN COATED VESICLE	134	0.097139089	0.178473453
PROTEIN KINASE COMPLEX	84	-0.23579599	0.004914178
VESICLE LUMEN	84	0.041509893	0.64541276
PROTEIN COMPLEX INVOLVED IN CELL ADHESION	25	-0.077472789	0.62967165
TRANS GOLGI NETWORK	181	0.12311134	0.048805778
PHOTORECEPTOR OUTER SEGMENT MEMBRANE	9	0.16049216	0.565844172
HOPS COMPLEX	12	0.05184732	0.827856307
IONOTROPIC GLUTAMATE RECEPTOR COMPLEX	38	0.093422885	0.488453549
ORGANELLE MEMBRANE CONTACT SITE	10	-0.103606673	0.68049147
SODIUM CHANNEL COMPLEX	14	-0.008413922	0.969257731
EUKARYOTIC TRANSLATION INITIATION FACTOR 3 COMPLEX	15	-0.586959517	0.004607628
CCR4 NOT COMPLEX	14	0.081083402	0.715127989
M BAND	16	0.024206005	0.906285586
NPBAF COMPLEX	12	-0.017621899	0.940305429
OUTER MITOCHONDRIAL MEMBRANE PROTEIN COMPLEX	11	-0.194280714	0.401072897
CORTICAL CYTOSKELETON	74	-0.156905595	0.084724122
MYOSIN FILAMENT	15	0.250473608	0.238749194
NEURONAL POSTSYNAPTIC DENSITY	48	0.003308705	0.977695441
INTERMEDIATE FILAMENT	97	0.139568725	0.100104063
CYTOSOLIC SMALL RIBOSOMAL SUBUNIT	41	-0.117442139	0.344203724
SNARE COMPLEX	49	-0.137890118	0.220208582
DNA REPLICATION FACTOR A COMPLEX	12	0.789269212	1.64E-05
UBIQUITIN LIGASE COMPLEX	239	-0.03256697	0.538580219
ROUGH ENDOPLASMIC RETICULUM MEMBRANE	19	-0.145140989	0.420636786
AXON INITIAL SEGMENT	11	0.052863537	0.831985282
U2 SNRNP	19	-0.450376683	0.002817156
ACTIN FILAMENT BUNDLE	51	0.079555764	0.493604517
PERINUCLEAR ENDOPLASMIC RETICULUM	12	-0.180396609	0.422747697
CILIARY TIP	37	0.030954609	0.819293004
PRONUCLEUS	14	-0.390548392	0.041873855
ORGANELLE INNER MEMBRANE	465	-0.135930119	0.000262295
GOLGI CISTERNA	84	-0.118279687	0.173203876
NUCLEAR NUCLEOSOME	33	0.106026796	0.464534569
PCG PROTEIN COMPLEX	43	-0.052618304	0.669877343
GOLGI APPARATUS PART	787	-0.01999573	0.502682621
POTASSIUM CHANNEL COMPLEX	65	0.135209273	0.192104479
SMALL RIBOSOMAL SUBUNIT	65	-0.14171143	0.147603707
COPI COATED VESICLE MEMBRANE	17	-0.37882877	0.032494046
PROTON TRANSPORTING TWO SECTOR ATPASE COMPLEX	43	-0.145476453	0.22448826
CENTROSOME	445	-0.064445629	0.09670238
SET1C COMPASS COMPLEX	9	0.072916133	0.791669986
CYTOPLASMIC MRNA PROCESSING BODY	65	-0.221187107	0.020443721

PROTEIN PHOSPHATASE TYPE 1 COMPLEX	10	-0.301480933	0.208255354
NODE OF RANVIER	13	0.204440221	0.378997216
COMPACT MYELIN	13	-0.216705704	0.30717231
MOTILE CILIUM	69	0.160131922	0.113628358
MICROVILLUS	59	0.149378241	0.171333289
DENDRITIC SHAFT	31	-0.0159237	0.913472728
CYTOSOLIC PROTEASOME COMPLEX	10	-0.514633521	0.008978746
PML BODY	90	-0.185483597	0.023822337
CELL CORTEX REGION	15	0.165348022	0.4406775
NEURON SPINE	104	-0.078820111	0.318060971
ENDOPLASMIC RETICULUM PART	1003	-0.062953204	0.017128335
NUCLEAR CHROMOSOME	481	0.00141968	0.970133823
ENDOCYTIC VESICLE MEMBRANE	131	0.104406773	0.153234861
HIGH DENSITY LIPOPROTEIN PARTICLE	16	0.279523473	0.171175183
NMDA SELECTIVE GLUTAMATE RECEPTOR COMPLEX	8	-0.025953909	0.92808496
BLOOD MICROPARTICLE	83	0.035930207	0.691959816
PRERIBOSOME	58	-0.168766592	0.10048392
PARANODE REGION OF AXON	11	0.269032611	0.279251553
AXONAL GROWTH CONE	19	0.144465428	0.448783277
PROTEIN DNA COMPLEX	153	0.042574375	0.525255674
APICAL DENDRITE	15	0.151712593	0.480512412
ACTOMYOSIN	58	0.083592598	0.443263857
STEREOCILIUM BUNDLE	33	0.063021088	0.66191223
ORGANELLAR RIBOSOME	69	-0.175158154	0.061200519
TRANSCRIPTIONAL REPRESSOR COMPLEX	70	-0.037168065	0.702072874
NUCLEAR REPLICATION FORK	36	0.214040765	0.123996449
INVADOPODIUM	9	-0.285871057	0.251765786
EXON EXON JUNCTION COMPLEX	20	0.023053235	0.900233804
CILIARY PART	235	-0.026438422	0.620970013
COSTAMERE	19	-0.195113341	0.277240198
SWI SNF SUPERFAMILY TYPE COMPLEX	68	-0.095866123	0.32365361
CHROMATIN	411	0.01328381	0.74606627
BASEMENT MEMBRANE	80	-0.037876112	0.67693245
BRUSH BORDER	87	0.02855103	0.746901138
MITOCHONDRIAL MATRIX	372	-0.124746939	0.002731143
PLATELET DENSE TUBULAR NETWORK	9	-0.257762044	0.302320342
CELL JUNCTION	1006	-0.05256358	0.046685082
NUCLEAR CHROMATIN	268	0.020253081	0.689118001
COMPLEX OF COLLAGEN TRIMERS	22	-0.153844278	0.358855121
EARLY ENDOSOME MEMBRANE	98	0.0558562	0.505080653
TRANSCRIPTION FACTOR COMPLEX	263	-0.091035765	0.067466812
MYELIN SHEATH	159	-0.019829735	0.76008287
SPLICEOSOMAL TRI SNRNP COMPLEX	26	0.033704859	0.834819537
PLATELET ALPHA GRANULE LUMEN	45	-0.096895845	0.415222928
MICROVILLUS MEMBRANE	15	-0.412051649	0.028405884
RIBONUCLEOPROTEIN GRANULE	139	-0.1291181	0.056171908
CLATHRIN COATED ENDOCYTIC VESICLE	49	0.199645303	0.095894359
ARP2 3 PROTEIN COMPLEX	11	-0.148022605	0.52887562
LEADING EDGE MEMBRANE	121	-0.035766658	0.629155204
CONTRACTILE FIBER	174	0.030839878	0.623122948
TRANSCRIPTION FACTOR TFTC COMPLEX	14	-0.041552464	0.847817791
CYTOSOLIC LARGE RIBOSOMAL SUBUNIT	59	-0.31523784	0.000946878
CLUSTER OF ACTIN BASED CELL PROJECTIONS	114	0.044678017	0.564636997
PROTEINACEOUS EXTRACELLULAR MATRIX	275	0.100547078	0.046952591
PHAGOCYTIC CUP	17	0.021502077	0.914124845
PROTON TRANSPORTING TWO SECTOR ATPASE COMPLEX CATALYTIC DOMAIN	15	0.090210371	0.674083088
CYTOSKELETAL PART	1191	-0.018912442	0.442218565
MICROTUBULE	346	-0.041711887	0.344003499
CYTOSKELETON	1657	-0.016799852	0.4289257
COPI COATED VESICLE	23	-0.270015277	0.08842752
NUCLEAR EUCHROMATIN	21	0.143744226	0.429915219
CORTICAL ACTIN CYTOSKELETON	53	-0.159361867	0.137887896
PERICENTRIC HETEROCHROMATIN	15	-0.30097274	0.111896898
GLIAL CELL PROJECTION	14	-0.074025513	0.730112702
SPECIFIC GRANULE	14	-0.07659907	0.720995484
HETEROTRIMERIC G PROTEIN COMPLEX	29	0.270440285	0.078077147

ENDOPLASMIC RETICULUM LUMEN	173	0.009779614	0.876015055
LATERAL PLASMA MEMBRANE	46	-0.140738482	0.225907927
BANDED COLLAGEN FIBRIL	11	0.395756846	0.099814098
EXTRINSIC COMPONENT OF ORGANELLE MEMBRANE	24	0.107905271	0.526061803
PLATELET DENSE GRANULE LUMEN	10	0.21143348	0.417862797
INTRACELLULAR VESICLE	1075	0.027454807	0.291337845
MYOSIN COMPLEX	51	0.087178158	0.453862554
VOLTAGE GATED SODIUM CHANNEL COMPLEX	12	-0.018826918	0.936206436
DNA REPAIR COMPLEX	37	-0.008783184	0.947890888
CLATHRIN VESICLE COAT	21	0.234564589	0.199525717
SIN3 COMPLEX	13	0.099269073	0.668447406
ESCRT COMPLEX	26	0.263995979	0.112048
CELL SUBSTRATE JUNCTION	386	-0.140801815	0.000556802
CILIARY TRANSITION ZONE	21	-0.169690806	0.316936247
U5 SNRNP	16	-0.230398542	0.226245661
ENDOCYTIC VESICLE LUMEN	16	0.235036978	0.257417069
HISTONE METHYLTRANSFERASE COMPLEX	67	-0.172739773	0.069882107
MICROTUBULE PLUS END	17	0.152394143	0.452764349
SAGA TYPE COMPLEX	31	-0.016586076	0.909873402
CELL DIVISION SITE	51	-0.170755522	0.116689168
EXTRACELLULAR MATRIX COMPONENT	110	0.029298522	0.709835677
NUCLEAR HETEROCHROMATIN	30	-0.03426729	0.81726822
TCTN B9D COMPLEX	11	0.055302358	0.824429349
PRC1 COMPLEX	12	0.171125253	0.476991201
GOLGI ASSOCIATED VESICLE	75	-0.139948774	0.126446358
INTRINSIC COMPONENT OF GOLGI MEMBRANE	51	-0.170406258	0.118311125
SYNAPSE	637	0.008518313	0.797344904
CHLORIDE CHANNEL COMPLEX	37	0.028754636	0.831840961
CLATHRIN COAT OF COATED PIT	15	0.413759045	0.04239775
SIN3 TYPE COMPLEX	16	0.057669196	0.780634004
POSTSYNAPTIC MEMBRANE	162	-0.043470358	0.496773863
SMALL SUBUNIT PROCESSOME	32	-0.104461757	0.458069445
RUFFLE MEMBRANE	75	-0.082853798	0.371821999
EXTERNAL ENCAPSULATING STRUCTURE	9	0.4838951	0.200878725
CELL TRAILING EDGE	11	-0.586538021	0.004368541
CATION CHANNEL COMPLEX	127	0.108850829	0.14199864
MLL1 2 COMPLEX	28	0.106663743	0.497103911
METHYLTRANSFERASE COMPLEX	86	-0.107322838	0.212124319
NUCLEOPLASM PART	665	-0.093805223	0.003179186
SYNAPTONEMAL COMPLEX	27	0.204226093	0.20147164
ORGANELLAR SMALL RIBOSOMAL SUBUNIT	25	-0.211721134	0.166261373
CELL CELL ADHERENS JUNCTION	49	-0.17648617	0.11290639
N TERMINAL PROTEIN ACETYLTRANSFERASE COMPLEX	10	-0.133411003	0.59215885
PROTON TRANSPORTING ATP SYNTHASE COMPLEX COUPLING FACTOR F O	10	-0.300421579	0.19093862
CONNEXON COMPLEX	13	-0.088244167	0.690366517
GOLGI MEMBRANE	628	-0.01999543	0.547091508
COATED VESICLE MEMBRANE	124	-0.140856559	0.047940166
SMOOTH ENDOPLASMIC RETICULUM	27	-0.190197696	0.200735819
ENDORIBONUCLEASE COMPLEX	20	-0.060590141	0.736811568
ATPASE COMPLEX	20	0.283393375	0.128661196
NUCLEAR INNER MEMBRANE	46	-0.057003548	0.63255539
LATE ENDOSOME	190	-0.049641201	0.400581435
VACUOLAR LUMEN	98	-0.054313601	0.506706822
LAMELLIPODIUM MEMBRANE	17	0.347603917	0.080203584
EXOSOME RNASE COMPLEX	21	-0.130393243	0.448579206
ER TO GOLGI TRANSPORT VESICLE MEMBRANE	49	-0.34647215	0.000960646
SPINDLE MIDZONE	25	-0.415859216	0.003776031
PRESYNAPTIC MEMBRANE	46	-0.129352867	0.266840905
NUCLEAR MEMBRANE PART	10	0.148209628	0.573870214
XY BODY	13	-0.315504966	0.122982837
MITOCHONDRIAL MEMBRANE PART	157	-0.216163426	0.000428137
COP9 SIGNALOSOME	29	-0.11578207	0.432186502
INNER MITOCHONDRIAL MEMBRANE PROTEIN COMPLEX	96	-0.182124163	0.02139088
NEURON PROJECTION	812	0.001938233	0.947654313
BLOC COMPLEX	20	-0.032191999	0.859341864
BETA CATENIN DESTRUCTION COMPLEX	13	0.256558923	0.265700043

APICOLATERAL PLASMA MEMBRANE	15	-0.015559043	0.941101167
CATALYTIC COMPLEX	961	-0.041251366	0.127294531
FILOPODIUM TIP	9	-0.18394439	0.474745983
MISMATCH REPAIR COMPLEX	10	-0.011651561	0.963995049
AXONEME PART	15	0.005413979	0.979584176
KINETOCHORE	116	-0.142078034	0.052553972
INTERSTITIAL MATRIX	12	0.152197751	0.528533386
EXTRACELLULAR SPACE	997	0.07794982	0.004113222
CYTOPLASMIC DYNEIN COMPLEX	14	0.293616508	0.183703787
ENDOLYSOSOME MEMBRANE	10	0.031822636	0.902724273
PRESPLICEOSOME	21	-0.35222208	0.024924457
CHD TYPE COMPLEX	17	-0.295424428	0.109050617
SPERM PRINCIPAL PIECE	12	0.171113061	0.477559094
CATALYTIC STEP 2 SPLICEOSOME	85	-0.213544996	0.010844425
VIRION	3	0.421916987	0.332800205
VACUOLAR PART	622	-0.021390029	0.521282539
MITOCHONDRIAL PART	856	-0.130475013	3.12E-06
MITOCHONDRION	1478	-0.096911764	1.08E-05
CELL SURFACE	589	0.009419734	0.784506267
HEMOGLOBIN COMPLEX	6	0.027881315	0.933838671
DENDRITE MEMBRANE	15	-0.356906501	0.051422198
RESPIRATORY CHAIN	71	-0.206652078	0.023358637
U4 U6 X U5 TRI SNRNP COMPLEX	21	0.080531169	0.656606525
PRP19 COMPLEX	12	0.010741207	0.963817325
CONDENSED CHROMOSOME CENTROMERIC REGION	98	-0.200338917	0.010236597
TRICARBOXYLIC ACID CYCLE ENZYME COMPLEX	11	-0.249301676	0.27027014
STAGA COMPLEX	14	-0.143392813	0.494000873
SPERM MIDPIECE	9	-0.214738462	0.400569568
SECRETORY GRANULE MEMBRANE	63	0.126653222	0.228051068
DNA PACKAGING COMPLEX	92	0.069271328	0.423662842
POLE PLASM	12	0.13764331	0.565767686
MEMBRANE MICRODOMAIN	246	-0.090813719	0.077579729
TRANSPORTER COMPLEX	237	0.072022476	0.18417821
SIDE OF MEMBRANE	331	-0.012226806	0.787444125
PHOTORECEPTOR CONNECTING CILIUM	22	-0.09087556	0.593148858
CONDENSED CHROMOSOME OUTER KINETOCHORE	12	-0.150678091	0.505095927
CLATHRIN COATED VESICLE MEMBRANE	68	0.139503724	0.169298948
MEMBRANE PROTEIN COMPLEX	865	-0.029421732	0.301581385
INTRINSIC COMPONENT OF THE CYTOPLASMIC SIDE OF THE PLASMA MEMBRANE	12	-0.105269183	0.645981117
NUCLEAR OUTER MEMBRANE ENDOPLASMIC RETICULUM MEMBRANE NETWORK	867	-0.075214556	0.007610014
TRANSCRIPTIONALLY ACTIVE CHROMATIN	18	0.096255395	0.623398234
U2 TYPE PRESPLICEOSOME	16	-0.408969198	0.020794994
ENDOPLASMIC RETICULUM CHAPERONE COMPLEX	11	-0.255955197	0.26752236
SCHMIDT LANTERMAN INCISURE	9	-0.108946178	0.680535581
SEMAPHORIN RECEPTOR COMPLEX	11	0.347087559	0.153178042
AXON CYTOPLASM	32	0.014610374	0.919856316
LAMELLIPODIUM	163	-0.038860461	0.542784394
NUCLEAR TRANSCRIPTION FACTOR COMPLEX	116	-0.135774534	0.064399179
MIDBODY	123	-0.072701221	0.317878828
HETEROCHROMATIN	64	-0.09041084	0.366971923
DNA HELICASE COMPLEX	14	0.157785529	0.477945209
CHROMOCENTER	12	-0.630641605	0.000793487
SPLICEOSOMAL COMPLEX	164	-0.141295536	0.022432641
NADH DEHYDROGENASE COMPLEX	41	-0.069961732	0.577640469
SARCOPLASM	57	-0.082930538	0.435093785
DNA DIRECTED RNA POLYMERASE II CORE COMPLEX	17	-0.084952219	0.661786399
CELL CELL JUNCTION	323	-0.101152107	0.024236293
CONDENSED NUCLEAR CHROMOSOME CENTROMERIC REGION	15	-0.549627483	0.001615612
PHOSPHATIDYLINOSITOL 3 KINASE COMPLEX	19	0.111780345	0.558222204
INTRACILIARY TRANSPORT PARTICLE B	15	-0.104859672	0.60890485
DNA DIRECTED RNA POLYMERASE I COMPLEX	13	0.169633408	0.463138151
REPLICATION FORK	59	0.028449179	0.790952051
IMMUNOGLOBULIN COMPLEX	3	0.226914725	0.631143804
SMALL NUCLEOLAR RIBONUCLEOPROTEIN COMPLEX	18	-0.377843195	0.022029284
SMN SM PROTEIN COMPLEX	17	-0.153550645	0.415830265
RECEPTOR COMPLEX	261	0.070275759	0.174288704

INTRACILIARY TRANSPORT PARTICLE	24	-0.117794627	0.465433741
ANCHORING JUNCTION	466	-0.126142079	0.000742484
PRERIBOSOME LARGE SUBUNIT PRECURSOR	20	-0.263498098	0.121844496
ENDOPLASMIC RETICULUM GOLGI INTERMEDIATE COMPARTMENT MEMBRANE	57	-0.11549046	0.272498247
EXTRINSIC COMPONENT OF CYTOPLASMIC SIDE OF PLASMA MEMBRANE	84	0.013597209	0.879593567
GAMMA TUBULIN COMPLEX	17	-0.16581536	0.380622058
POLYSOME	41	0.061501407	0.634845114
PRESYNAPTIC ACTIVE ZONE	26	0.016787295	0.917022898
EXOCYST	14	0.041273473	0.851490559
DNA DIRECTED RNA POLYMERASE III COMPLEX	18	-0.041346699	0.828672129
PODOSOME	22	0.019131378	0.913070027
CILIARY BASE	20	-0.20289756	0.236436292
AUTOPHAGOSOME MEMBRANE	25	-0.037478601	0.817442288
INTRINSIC COMPONENT OF MITOCHONDRIAL INNER MEMBRANE	22	-0.034127181	0.843713837
DYNEIN COMPLEX	32	0.250092303	0.086760719
ORGANELLAR LARGE RIBOSOMAL SUBUNIT	29	-0.240493537	0.086245762
CARBOXY TERMINAL DOMAIN PROTEIN KINASE COMPLEX	21	-0.188183091	0.264389393
DESMOSOME	21	-0.08507943	0.627085778
RECYCLING ENDOSOME MEMBRANE	37	-0.000785088	0.995347051
ORGANELLE ENVELOPE LUMEN	77	-0.134985318	0.134042678
SPINDLE MICROTUBULE	54	-0.157518839	0.140634502
GOLGI TRANSPORT COMPLEX	12	-0.020349372	0.931034374
OUTER MEMBRANE	168	-0.078619131	0.206846958
RIBOSOMAL SUBUNIT	155	-0.234619926	0.000136864
CIS GOLGI NETWORK	38	0.068201884	0.612413967
TRANS GOLGI NETWORK TRANSPORT VESICLE	26	0.090231328	0.581157335
INHIBITORY SYNAPSE	10	0.083233843	0.75091803
MICROTUBULE END	22	0.26705714	0.133577835
SUPRAMOLECULAR FIBER	505	-0.009897969	0.788799597
FIBRIL	12	0.054390193	0.819596821
NONMOTILE PRIMARY CILIUM	93	-0.07573961	0.364066952
NUCLEAR MATRIX	89	-0.143510481	0.086192352
AXOLEMMA	14	0.188586337	0.402559506
MICROTUBULE ASSOCIATED COMPLEX	118	-0.033222149	0.657943487
APICAL PLASMA MEMBRANE	236	-0.030767288	0.563811343
PHAGOCYTIC VESICLE MEMBRANE	51	-0.076611094	0.496453428
PHOTORECEPTOR INNER SEGMENT	23	0.314502052	0.063275921
EUCHROMATIN	28	0.176249346	0.263550341
CELL CORTEX	211	-0.07851988	0.158039343
DNA DIRECTED RNA POLYMERASE II HOLOENZYME	94	-0.129477541	0.112788044
ENDOPLASMIC RETICULUM QUALITY CONTROL COMPARTMENT	12	-0.598404705	0.002079579
INTRINSIC COMPONENT OF MITOCHONDRIAL MEMBRANE	49	-0.054317516	0.638077563
LATERAL ELEMENT	9	0.131303551	0.63589268
CYTOPLASMIC VESICLE PART	514	0.042425106	0.251675858
INTERMEDIATE FILAMENT CYTOSKELETON	138	0.145060484	0.042069986
ENDOSOMAL PART	388	0.030847874	0.46613695
ACROSOMAL VESICLE	64	0.149479576	0.152397911
PSEUDOPODIUM	14	-0.115053083	0.589028786
T TUBULE	39	0.047764434	0.718196733
ENDOPLASMIC RETICULUM GOLGI INTERMEDIATE COMPARTMENT	95	-0.189478972	0.017594163
PLASMA MEMBRANE REGION	744	-0.011667062	0.703794849
NEURON PROJECTION MEMBRANE	30	-0.084813986	0.562427292
INTEGRATOR COMPLEX	13	-0.12720061	0.561185818
SARCOLEMMA	106	-0.051160273	0.516055674
IKAPPAB KINASE COMPLEX	9	-0.00611302	0.982095308
CELL BODY	416	0.04161665	0.310020756
LYTIC VACUOLE	453	-0.106130075	0.00530212
CUL4 RING E3 UBIQUITIN LIGASE COMPLEX	24	-0.430473653	0.002672077
CONDENSED CHROMOSOME	181	-0.137151606	0.02003004
ESC E Z COMPLEX	16	-0.412159595	0.023076223
NUCLEAR PERIPHERY	112	-0.13231682	0.07733754
CULLIN RING UBIQUITIN LIGASE COMPLEX	134	-0.014223407	0.840698395
MAIN AXON	50	0.088572896	0.451535186
LAMELLAR BODY	17	0.393294303	0.055769347
CHROMATOID BODY	13	-0.159051403	0.4648362
CHROMOSOME TELOMERIC REGION	154	-0.035118127	0.593149004

TOR COMPLEX	13	-0.143060871	0.510558355
WNT SIGNALOSOME	10	-0.027050923	0.916220146
ACTIN BASED CELL PROJECTION	148	-0.001958649	0.976866532
CELL PROJECTION	1487	0.011624154	0.603598652
REPLISOME	26	0.308371437	0.058623251
CILIARY MEMBRANE	52	0.182602839	0.113449665
COATED VESICLE	209	-0.108194996	0.051313398
INTERCELLULAR BRIDGE	40	-0.004707577	0.970975035
CELL PROJECTION PART	791	-0.014008904	0.638243555
MHC CLASS II PROTEIN COMPLEX	12	-0.022995829	0.9220373
KERATIN FILAMENT	41	0.221445326	0.086786046
RNAI EFFECTOR COMPLEX	11	-0.25218708	0.2651787
PRESYNAPSE	241	0.096094777	0.075045869
A BAND	27	0.126390073	0.429934954
NEUROMUSCULAR JUNCTION	46	-0.012025701	0.92048873
SOMATODENDRITIC COMPARTMENT	552	-0.006289138	0.859095209
PRE AUTOPHAGOSOMAL STRUCTURE MEMBRANE	16	0.351902272	0.090525421
EXTERNAL SIDE OF PLASMA MEMBRANE	164	0.052989773	0.414118909
LYTIC VACUOLE MEMBRANE	251	-0.100736683	0.04740496
CUL3 RING UBIQUITIN LIGASE COMPLEX	54	0.291996792	0.011669346
ENVELOPE	985	-0.133864243	3.39E-07
PRIMARY CILIUM	153	-0.010619736	0.87277461
RUFFLE	142	-0.112149303	0.09425783
WASH COMPLEX	10	0.142041304	0.589442428
DENDRITE CYTOPLASM	14	0.108958651	0.623481261
CORNIFIED ENVELOPE	20	0.23036385	0.210665062
PERIKARYON	85	0.108206999	0.231423041
MICROBODY PART	79	0.038548449	0.678385485
GOLGI APPARATUS	1271	-0.01438463	0.547498124
NUCLEOLUS	793	-0.08434752	0.004033555
PERICENTRIOLAR MATERIAL	16	-0.26505006	0.153658407
TELOMERE CAP COMPLEX	12	-0.399071627	0.048011117
NUCLEOTIDE EXCISION REPAIR COMPLEX	13	0.081922935	0.721994531
NUCLEAR PORE	74	-0.257334199	0.003978839
AP TYPE MEMBRANE COAT ADAPTOR COMPLEX	39	-0.070104715	0.586512201
BAF TYPE COMPLEX	21	-0.116918196	0.502194748
MYOSIN II COMPLEX	19	0.162158521	0.394859864
MICROTUBULE ORGANIZING CENTER PART	131	-0.100319719	0.151608919
NUCLEAR MEMBRANE	255	-0.141972075	0.004435219
SWI SNF COMPLEX	13	-0.049877944	0.824208472
CYTOPLASMIC MICROTUBULE	49	-0.087603977	0.443780065
CLATHRIN COAT OF ENDOCYTIC VESICLE	11	0.497814556	0.03176074
HOST	11	0.000410755	0.998671832
PIGMENT GRANULE	94	0.008979656	0.91566627
EXTRINSIC COMPONENT OF MEMBRANE	217	0.016673457	0.766413158
SPINDLE	267	-0.079380615	0.109286903
TRANSPORT VESICLE	297	0.028916741	0.548710555
TRANSCRIPTION ELONGATION FACTOR COMPLEX	42	0.039630578	0.755794857
ACTIN FILAMENT	65	-0.057817179	0.564303196
INTRINSIC COMPONENT OF EXTERNAL SIDE OF PLASMA MEMBRANE	14	-0.017680051	0.93532279
CILIARY PLASM	62	0.031209154	0.765738511
SPERM FLAGELLUM	38	0.173520684	0.206004528
GOLGI LUMEN	68	0.042601418	0.670777299
ESCRT III COMPLEX	11	0.144920092	0.566404415
COHESIN COMPLEX	9	-0.347071434	0.144907811
MITOCHONDRIAL ENVELOPE	622	-0.126389814	0.000101572
RIBONUCLEOPROTEIN COMPLEX	678	-0.148293464	1.88E-06
CELL LEADING EDGE	324	-0.0308562	0.4989315
CLATHRIN COATED ENDOCYTIC VESICLE MEMBRANE	38	0.15842212	0.244020857
ENDOPLASMIC RETICULUM	1384	-0.043746356	0.056014822
CALCIUM CHANNEL COMPLEX	51	0.066779315	0.565174126
TRANSLATION PREINITIATION COMPLEX	15	-0.431678899	0.027645231
MITOCHONDRIAL PROTEIN COMPLEX	125	-0.168352888	0.015899403
MICROTUBULE ORGANIZING CENTER	566	-0.045135789	0.19349617
PROTON TRANSPORTING V TYPE ATPASE COMPLEX	21	0.057440677	0.750299777
PROTEIN LIPID COMPLEX	27	0.352013888	0.025075096

MEMBRANE REGION	928	-0.006951136	0.801726018
NBAF COMPLEX	13	0.277492981	0.236113793
MEDIATOR COMPLEX	33	-0.05464367	0.697761946
CILIUM	353	-0.006841484	0.876419956
EXTRINSIC COMPONENT OF PLASMA MEMBRANE	116	0.021685023	0.777066059
AXON PART	193	0.086406214	0.151000472
BRUSH BORDER MEMBRANE	44	-0.002756078	0.982185316
TERMINAL BOUTON	56	0.049916161	0.651719135
ANCHORED COMPONENT OF PLASMA MEMBRANE	23	-0.105753316	0.524568278
AXONEMAL DYNEIN COMPLEX	7	0.154085294	0.623725863
MAST CELL GRANULE	17	-0.311069979	0.085206202
RECYCLING ENDOSOME	114	0.051534265	0.507160931
COATED MEMBRANE	84	0.022840272	0.799506882
SYNAPSE PART	516	-0.006117351	0.867249806
EARLY ENDOSOME	268	0.047790055	0.347684873
PROTEASOME ACCESSORY COMPLEX	22	-0.314347452	0.042182377
CYTOSOLIC RIBOSOME	107	-0.207149294	0.00556146
PHOTORECEPTOR DISC MEMBRANE	9	0.201400417	0.466546556
PROTEIN PHOSPHATASE TYPE 2A COMPLEX	19	-0.088015354	0.632049833
CILIARY BASAL BODY	71	-0.000734411	0.993977453
TRANS GOLGI NETWORK MEMBRANE	74	0.18907639	0.052355981
ENDOSOME	714	-0.00588412	0.851014482
TRANSPORT VESICLE MEMBRANE	130	-0.021795175	0.761178632
NEURONAL CELL BODY MEMBRANE	15	0.281358033	0.1916741
HISTONE DEACETYLASE COMPLEX	58	-0.072994419	0.489969198
PHOTORECEPTOR OUTER SEGMENT	42	0.087630468	0.494291318
U12 TYPE SPLICEOSOMAL COMPLEX	25	-0.165261518	0.286194261
BASAL PART OF CELL	44	0.016034464	0.897042696
SODIUM POTASSIUM EXCHANGING ATPASE COMPLEX	8	0.05881124	0.840686907
SITE OF DOUBLE STRAND BREAK	29	0.316010711	0.048560407
PLASMA MEMBRANE RAFT	74	-0.167333281	0.066392265
MICROBODY LUMEN	37	0.101208861	0.45851204
FILAMENTOUS ACTIN	19	-0.146471256	0.416472236
METHYLOSOME	12	0.027920731	0.906427744
GOLGI CISTERNA MEMBRANE	66	-0.159944522	0.09800258
PROTON TRANSPORTING ATP SYNTHASE COMPLEX	20	-0.380634816	0.016005684
PLASMA MEMBRANE PROTEIN COMPLEX	402	0.0340371	0.413556186
CENTRIOLAR SATELLITE	21	-0.285693892	0.075034632
CLATHRIN ADAPTOR COMPLEX	26	-0.150563162	0.328468021
GAP JUNCTION	22	0.00616682	0.971850655
DERLIN 1 RETROTRANSLOCATION COMPLEX	10	-0.049961229	0.845366744
PLATELET DENSE GRANULE	16	0.011806084	0.954101284
DENSE CORE GRANULE	13	-0.207568563	0.32796828
SYNAPTIC MEMBRANE	211	-0.08206618	0.139743062
EXOCYTIC VESICLE MEMBRANE	49	0.228050956	0.056755557
ACETYLTRANSFERASE COMPLEX	95	-0.002391347	0.977329008
ANCHORED COMPONENT OF MEMBRANE	95	0.084127018	0.324377489
MHC CLASS I PROTEIN COMPLEX	10	-0.532035478	0.011564034
DENDRITE	385	-0.018840635	0.654223227
NUCLEAR UBIQUITIN LIGASE COMPLEX	40	0.020347394	0.875675608
ORGANELLE SUBCOMPARTMENT	287	-0.015528434	0.749291653
CLATHRIN COAT OF TRANS GOLGI NETWORK VESICLE	10	0.140767113	0.594711231
CHYLOMICRON	9	0.259026599	0.341843029
PRIMARY LYSOSOME	12	-0.07119145	0.758968852
TRIGLYCERIDE RICH LIPOPROTEIN PARTICLE	15	0.263084851	0.215766829
ENDOPLASMIC RETICULUM SUBCOMPARTMENT	15	0.149723512	0.488656239
COLLAGEN TRIMER	61	0.072189808	0.497242173
SMALL NUCLEAR RIBONUCLEOPROTEIN COMPLEX	60	-0.232410306	0.018087423
CYCLIN DEPENDENT PROTEIN KINASE HOLOENZYME COMPLEX	30	-0.408516434	0.001965714
VACUOLAR MEMBRANE	534	-0.007241856	0.840524638

Table S4. miRNA GO molecular function.

GO Term	Genes Tested	Model Coefficient	p-value
CYCLIC NUCLEOTIDE PHOSPHODIESTERASE ACTIVITY	22	0.14930253	0.400724189
PROTEIN SERINE THREONINE TYROSINE KINASE ACTIVITY	37	0.053682046	0.693220938
HYDROLASE ACTIVITY ACTING ON CARBON NITROGEN BUT NOT PEPTIDE BONDS IN CYCLIC AMIDINES	28	0.168171412	0.288030746
5 3 EXONUCLEASE ACTIVITY	12	-0.328097674	0.11059667
POLYUBIQUITIN BINDING	39	0.020535641	0.87612819
COFACTOR TRANSPORTER ACTIVITY	20	0.112019616	0.546996654
BETA CATENIN BINDING	77	0.055750675	0.554922861
UDP GALACTOSYLTRANSFERASE ACTIVITY	25	-0.227619444	0.138586213
MITOGEN ACTIVATED PROTEIN KINASE BINDING	22	-0.237460672	0.141500332
PHOSPHATE TRANSMEMBRANE TRANSPORTER ACTIVITY	27	-0.263664378	0.06848877
PEPTIDASE ACTIVITY	461	-0.054600958	0.153198267
IMMUNOGLOBULIN BINDING	12	0.135274282	0.572084354
PROTEIN KINASE A REGULATORY SUBUNIT BINDING	14	-0.41903431	0.026162421
SIGNAL TRANSDUCER ACTIVITY	1040	0.086422515	0.001195852
MHC PROTEIN COMPLEX BINDING	17	0.144758793	0.475874409
IONOTROPIC GLUTAMATE RECEPTOR BINDING	22	-0.076677369	0.653951989
UBIQUITIN LIKE PROTEIN TRANSFERASE ACTIVITY	383	0.002428849	0.954276588
TRANSMEMBRANE RECEPTOR PROTEIN KINASE ACTIVITY	66	0.094698525	0.355538118
SMAD BINDING	69	0.165135487	0.101486793
SYNTAXIN 1 BINDING	17	-0.014028698	0.94348673
LONG CHAIN FATTY ACID BINDING	11	-0.094097542	0.695522227
ACETYLGALACTOSAMINYLTRANSFERASE ACTIVITY	29	0.050339754	0.742891961
OXIDOREDUCTASE ACTIVITY ACTING ON THE ALDEHYDE OR OXO GROUP OF DONORS NAD OR NADP AS ACCEPTOR	32	0.013491212	0.925957421
ACTIVE ION TRANSMEMBRANE TRANSPORTER ACTIVITY	133	-0.032896803	0.641756145
CYTOKINE BINDING	78	0.134525675	0.155809427
NEUROTRANSMITTER BINDING	13	0.00511808	0.982029853
OLFACTORY RECEPTOR ACTIVITY	48	0.266792699	0.023700366
SERINE TYPE EXOPEPTIDASE ACTIVITY	14	-0.247577642	0.217114061
3 5 CYCLIC AMP PHOSPHODIESTERASE ACTIVITY	15	0.124035197	0.56416505
VOLTAGE GATED ION CHANNEL ACTIVITY	136	0.256638848	0.000323999
PHOSPHATIDYLINOSITOL 3 5 BISPHOSPHATE BINDING	19	-0.339987294	0.038718628
PROTEIN KINASE C BINDING	44	-0.104738273	0.383971381
BETA 2 MICROGLOBULIN BINDING	8	-0.300314909	0.251625602
PROTEIN COMPLEX SCAFFOLD	62	0.047437969	0.651624021
HEAT SHOCK PROTEIN BINDING	84	-0.082925999	0.344052786
PHOSPHOPROTEIN BINDING	53	-0.155717606	0.14922201
GTPASE ACTIVITY	217	0.071249571	0.208936291
AMINO ACID BINDING	86	-0.130027994	0.12753779
OXIDOREDUCTASE ACTIVITY ACTING ON THE CH CH GROUP OF DONORS	50	-0.020648538	0.857912012
OXIDOREDUCTASE ACTIVITY ACTING ON A HEME GROUP OF DONORS	21	-0.272948267	0.08984041
QUINONE BINDING	16	-0.040159225	0.842904067
G PROTEIN ALPHA SUBUNIT BINDING	19	-0.069954522	0.70470369
MITOGEN ACTIVATED PROTEIN KINASE KINASE BINDING	16	0.083076427	0.689302012
PEPTIDASE REGULATOR ACTIVITY	157	0.047205747	0.475808667
CYTOSKELETAL PROTEIN BINDING	713	-0.04885655	0.115845859
VITAMIN TRANSPORTER ACTIVITY	22	-0.179519951	0.278095295
TRANSFERASE ACTIVITY TRANSFERRING SULFUR CONTAINING GROUPS	56	-0.001250556	0.990886834
CYCLASE ACTIVITY	15	0.265484536	0.20752608
PROTEIN SELF ASSOCIATION	38	-0.201160801	0.107209619
CYSTEINE TYPE PEPTIDASE ACTIVITY	141	-0.079174059	0.243670145
LIPID TRANSPORTER ACTIVITY	87	0.36117929	3.14E-05
ENDODEOXYRIBONUCLEASE ACTIVITY PRODUCING 5 PHOSPHOMONOESTERS	10	-0.318052086	0.163253276
SULFUR COMPOUND TRANSMEMBRANE TRANSPORTER ACTIVITY	20	-0.22071786	0.193893402
DNA POLYMERASE ACTIVITY	32	-0.137732595	0.322198128
HISTONE BINDING	161	0.021541065	0.740729804
UBIQUITIN LIKE PROTEIN BINDING	116	0.039064709	0.611216932
DNA BINDING BENDING	19	0.113696348	0.551760395
RNA METHYLTRANSFERASE ACTIVITY	42	-0.053962758	0.66532731
CYCLIC NUCLEOTIDE GATED ION CHANNEL ACTIVITY	7	0.086745894	0.781844017
NARROW PORE CHANNEL ACTIVITY	10	0.342492849	0.176768446

STEROID DEHYDROGENASE ACTIVITY	21	0.237740183	0.186951974
BHLH TRANSCRIPTION FACTOR BINDING	25	0.131299936	0.431326992
G PROTEIN COUPLED NEUROTRANSMITTER RECEPTOR ACTIVITY	9	0.29231595	0.275425704
METHYLATED HISTONE BINDING	45	-0.128883145	0.274113225
ATP DEPENDENT DNA HELICASE ACTIVITY	30	-0.048246119	0.74420797
PURINE NTP DEPENDENT HELICASE ACTIVITY	89	-0.059482847	0.487878213
PROTEIN BINDING INVOLVED IN CELL ADHESION	15	-0.162058898	0.421609718
FIBROBLAST GROWTH FACTOR RECEPTOR BINDING	22	0.098786424	0.57674861
VOLTAGE GATED ANION CHANNEL ACTIVITY	12	0.578537998	0.005101267
RNA POLYMERASE II TRANSCRIPTION COREPRESSOR ACTIVITY	23	0.211293308	0.22892623
PHOSPHATASE INHIBITOR ACTIVITY	29	-0.154639407	0.28704779
MAGNESIUM ION BINDING	179	0.114956121	0.066813794
DNA SECONDARY STRUCTURE BINDING	22	-0.204219063	0.212933129
SULFUR COMPOUND BINDING	193	0.084352116	0.160382299
SIGNAL SEQUENCE BINDING	36	-0.247217945	0.051649819
OXIDOREDUCTASE ACTIVITY ACTING ON THE CH OH GROUP OF DONORS<br>NAD OR NADP AS ACCEPTOR	90	0.120806368	0.169441637
SYNTAXIN BINDING	79	0.012651499	0.891236685
HEPARAN SULFATE SULFOTRANSFERASE ACTIVITY	12	-0.122723795	0.5904955
PHOSPHATIDYLINOSITOL PHOSPHATE PHOSPHATASE ACTIVITY	27	-0.047267364	0.761564453
CARBOHYDRATE BINDING	202	0.125167971	0.033763551
TAU PROTEIN BINDING	9	0.083960181	0.762360208
ACYLGLYCEROL O ACYLTRANSFERASE ACTIVITY	25	-0.084163887	0.5995595
COMPLEMENT BINDING	12	-0.065329912	0.778503156
HORMONE RECEPTOR BINDING	143	-0.145344119	0.028317891
DIVALENT INORGANIC CATION TRANSMEMBRANE TRANSPORTER<br>ACTIVITY	138	0.06792258	0.337583103
PROTEIN HETERODIMERIZATION ACTIVITY	398	0.060697862	0.148577844
PROTEIN PHOSPHATASE BINDING	110	0.116322264	0.145513648
MONOVALENT INORGANIC CATION TRANSMEMBRANE TRANSPORTER<br>ACTIVITY	265	0.032056212	0.52975677
3 5 CYCLIC GMP PHOSPHODIESTERASE ACTIVITY	11	0.122005315	0.626069495
VOLTAGE GATED CATION CHANNEL ACTIVITY	96	0.251709982	0.003025301
PROTEIN TYROSINE KINASE ACTIVITY	146	0.022766924	0.739059146
TRANSMITTER GATED CHANNEL ACTIVITY	19	-0.039657425	0.831124896
PROTEIN DEACETYLASE ACTIVITY	41	-0.100180124	0.421630092
ENDONUCLEASE ACTIVITY ACTIVE WITH EITHER RIBO OR<br>DEOXYRIBONUCLEIC ACIDS AND PRODUCING 3 PHOSPHOMONOESTERS	15	-0.021785715	0.917474256
SODIUM AMINO ACID SYMPORTER ACTIVITY	9	0.087296701	0.752909464
FATTY ACYL COA BINDING	26	0.161765321	0.321917562
PHOSPHATIDYLINOSITOL 3 5 BISPHOSPHATE 3 PHOSPHATASE ACTIVITY	10	-0.084174642	0.739446015
TRANSCRIPTIONAL ACTIVATOR ACTIVITY RNA POLYMERASE II DISTAL<br>ENHANCER SEQUENCE SPECIFIC BINDING	21	-0.460685365	0.003694263
CYCLIN BINDING	18	-0.102715045	0.584878812
ACID AMINO ACID LIGASE ACTIVITY	18	-0.096751414	0.606854357
OXIDOREDUCTASE ACTIVITY ACTING ON NAD P H	81	-0.1411875	0.106734402
CORE PROMOTER BINDING	140	-0.058483496	0.39339549
NEUROPEPTIDE RECEPTOR BINDING	22	0.160726975	0.363808731
RETINOIC ACID BINDING	13	0.294515964	0.202838522
DIACYLGLYCEROL KINASE ACTIVITY	10	0.463418586	0.053935016
STEROID BINDING	74	0.269336327	0.005061859
LEUCINE ZIPPER DOMAIN BINDING	10	0.206295518	0.436584206
PHOSPHATIDYLINOSITOL 5 PHOSPHATE BINDING	11	-0.300621058	0.17285474
I SMAD BINDING	11	0.316396145	0.207489369
ENHANCER BINDING	80	-0.261698765	0.002006029
DEMETHYLASE ACTIVITY	32	0.050990156	0.727456598
RAC GUANYL NUCLEOTIDE EXCHANGE FACTOR ACTIVITY	12	-0.298769841	0.161317283
PROTEIN BINDING INVOLVED IN PROTEIN FOLDING	11	-0.147924147	0.530330158
KINASE INHIBITOR ACTIVITY	80	-0.115976963	0.192052833
PHOTORECEPTOR ACTIVITY	8	-0.1592575	0.561921138
CYTOSKELETAL ADAPTOR ACTIVITY	14	-0.094997987	0.656107842
TRANSCRIPTION FACTOR ACTIVITY DIRECT LIGAND REGULATED<br>SEQUENCE SPECIFIC DNA BINDING	42	0.110252312	0.391664911
RACEMASE AND EPIMERASE ACTIVITY	15	0.014651382	0.944892143
PROTEIN LIPID COMPLEX BINDING	20	0.333430956	0.076552674
CARBOXY LYASE ACTIVITY	31	0.049589075	0.738265071

MIRNA BINDING	15	-0.387010889	0.034962349
ESTROGEN RECEPTOR BINDING	37	-0.003517162	0.97914486
TRANSCRIPTIONAL REPRESSOR ACTIVITY RNA POLYMERASE II TRANSCRIPTION REGULATORY REGION SEQUENCE SPECIFIC BINDING	141	0.056378748	0.420361764
UBIQUITIN UBIQUITIN LIGASE ACTIVITY	13	-0.135180263	0.536034142
FMN BINDING	12	-0.018529489	0.937215202
ADENYLYLTRANSFERASE ACTIVITY	22	0.152378382	0.395419134
ATPASE ACTIVITY COUPLED	273	-0.04976357	0.313813367
S ACYLTRANSFERASE ACTIVITY	27	-0.167380947	0.263150085
ANKYRIN BINDING	18	0.187469327	0.339112573
PROTEASOME BINDING	16	0.043046858	0.834826701
DNA N GLYCOSYLASE ACTIVITY	14	0.193056399	0.385732592
PHOSPHATIDYLINOSITOL MONOPHOSPHATE PHOSPHATASE ACTIVITY	13	-0.109733355	0.617972626
PROTEIN DIMERIZATION ACTIVITY	959	0.024470663	0.372168045
TRANSFERASE ACTIVITY TRANSFERRING ALKYL OR ARYL OTHER THAN METHYL GROUPS	53	0.011605794	0.918077704
UNFOLDED PROTEIN BINDING	84	-0.107793114	0.215545759
POLY A BINDING	13	-0.092762276	0.677746146
ATPASE ACTIVATOR ACTIVITY	16	-0.361725714	0.046346572
CARBOHYDRATE PHOSPHATASE ACTIVITY	8	-0.229755791	0.391727009
VIRUS RECEPTOR ACTIVITY	64	0.019702657	0.848137074
NUCLEOSOME BINDING	44	-0.002209729	0.985718062
UBIQUITIN LIKE PROTEIN CONJUGATING ENZYME ACTIVITY	29	-0.334870995	0.01357101
ATP DEPENDENT MICROTUBULE MOTOR ACTIVITY	14	-0.239672333	0.237318669
C2H2 ZINC FINGER DOMAIN BINDING	13	0.271082888	0.236554316
CALCIUM ACTIVATED POTASSIUM CHANNEL ACTIVITY	12	0.178687198	0.455756903
ACTIN BINDING	346	-0.034183631	0.438865047
PHOSPHATIDYLINOSITOL 4 5 BISPHOSPHATE BINDING	44	-0.208128932	0.071591701
PRENYLTRANSFERASE ACTIVITY	14	0.157623126	0.48074488
ANION CATION SYMPORTER ACTIVITY	41	0.16262579	0.211364312
LYSINE ACETYLATED HISTONE BINDING	17	-0.071663774	0.71307142
PEPTIDE HORMONE RECEPTOR BINDING	12	-0.155373578	0.488996996
INORGANIC ANION TRANSMEMBRANE TRANSPORTER ACTIVITY	97	0.035407425	0.673017822
PROTEIN KINASE A BINDING	37	-0.092279808	0.483403024
MANNOSIDASE ACTIVITY	14	0.109341837	0.622324949
KINASE BINDING	561	-0.10861999	0.001567593
METAL ION TRANSMEMBRANE TRANSPORTER ACTIVITY	315	0.101357032	0.032285186
COA HYDROLASE ACTIVITY	13	0.123254294	0.594118371
ADRENERGIC RECEPTOR ACTIVITY	8	0.139994962	0.634037515
GATED CHANNEL ACTIVITY	231	0.156572389	0.004621326
RNA DNA HYBRID RIBONUCLEASE ACTIVITY	7	-0.354967234	0.189472531
HISTONE DEACETYLASE BINDING	95	-0.094217916	0.25247062
ACTIN FILAMENT BINDING	111	-0.037836811	0.624284543
HYDROLASE ACTIVITY ACTING ON CARBON NITROGEN BUT NOT PEPTIDE BONDS IN LINEAR AMIDINES	7	-0.133072102	0.653850612
PROTEIN KINASE ACTIVITY	562	0.04450385	0.20956196
HEPARIN BINDING	124	0.152646144	0.042140822
EPIDERMAL GROWTH FACTOR RECEPTOR BINDING	28	0.03838416	0.805573724
LOW DENSITY LIPOPROTEIN PARTICLE BINDING	12	0.193068511	0.42922996
SERINE HYDROLASE ACTIVITY	138	-0.005091173	0.941919696
TRANSMEMBRANE RECEPTOR PROTEIN TYROSINE KINASE ACTIVITY	50	0.158280435	0.180227191
WNT PROTEIN BINDING	24	-0.139171516	0.384837849
TRANSFERASE ACTIVITY TRANSFERRING HEXOSYL GROUPS	163	-0.047163099	0.45923434
ARYL SULFOTRANSFERASE ACTIVITY	8	0.426436776	0.10940488
PROTEIN SERINE THREONINE PHOSPHATASE INHIBITOR ACTIVITY	8	-0.091156687	0.747123717
HYDRO LYASE ACTIVITY	45	-0.028015409	0.817389591
TRANSLATION INITIATION FACTOR ACTIVITY	48	-0.206731077	0.064416535
LIPOPROTEIN PARTICLE RECEPTOR BINDING	20	0.0178836	0.922455349
LIGAND GATED CHANNEL ACTIVITY	98	0.054305009	0.516547694
RHO GTPASE BINDING	72	0.009595577	0.921062098
PROTEIN HOMODIMERIZATION ACTIVITY	605	0.016004936	0.638332448
POLY PYRIMIDINE TRACT BINDING	17	0.214821332	0.296638406
PROTEIN PHOSPHORYLATED AMINO ACID BINDING	20	0.055493731	0.764267638
COLLAGEN BINDING	52	0.051372593	0.654499483
REGULATORY REGION NUCLEIC ACID BINDING	715	-0.048339792	0.119369674
TRIGLYCERIDE LIPASE ACTIVITY	12	0.4639488	0.030658904

N METHYLTRANSFERASE ACTIVITY	75	0.027503727	0.772728039
TRANSCRIPTIONAL ACTIVATOR ACTIVITY RNA POLYMERASE II TRANSCRIPTION REGULATORY REGION SEQUENCE SPECIFIC BINDING	276	0.059652151	0.235361595
L AMINO ACID TRANSMEMBRANE TRANSPORTER ACTIVITY	45	0.000326199	0.997869014
ACETYLCHOLINE RECEPTOR ACTIVITY	14	0.033693854	0.878353441
CALCIUM ACTIVATED CATION CHANNEL ACTIVITY	19	0.15787707	0.407427875
KINASE REGULATOR ACTIVITY	167	-0.000942142	0.988188579
ENDONUCLEASE ACTIVITY ACTIVE WITH EITHER RIBO OR DEOXYRIBONUCLEIC ACIDS AND PRODUCING 5 PHOSPHOMONOESTERS	33	-0.11774157	0.393476837
CARBOXYPEPTIDASE ACTIVITY	31	0.082107062	0.58156024
ATPASE REGULATOR ACTIVITY	29	-0.310966728	0.022719615
PHOSPHATIDYLINOSITOL PHOSPHATE BINDING	102	-0.121892175	0.121202638
GTPASE INHIBITOR ACTIVITY	14	-0.101523781	0.63281335
PHOSPHATIDYLINOSITOL PHOSPHATE 5 PHOSPHATASE ACTIVITY	13	0.145919543	0.527816943
ATPASE ACTIVITY COUPLED TO TRANSMEMBRANE MOVEMENT OF IONS PHOSPHORYLATIVE MECHANISM	28	0.104793369	0.50600211
GLUCOSIDASE ACTIVITY	11	0.163323884	0.516053047
ORGANIC HYDROXY COMPOUND TRANSMEMBRANE TRANSPORTER ACTIVITY	38	-0.056320899	0.667337961
EXTRACELLULAR MATRIX STRUCTURAL CONSTITUENT	60	0.007384426	0.944451806
SOLUTE PROTON ANTIPORTER ACTIVITY	12	0.033479046	0.888028698
ACIDIC AMINO ACID TRANSMEMBRANE TRANSPORTER ACTIVITY	14	0.211160331	0.343204776
ENDORIBONUCLEASE ACTIVITY PRODUCING 5 PHOSPHOMONOESTERS	26	-0.089452901	0.568392088
LYSOPHOSPHOLIPASE ACTIVITY	15	0.560737229	0.001985162
ADRENERGIC RECEPTOR BINDING	19	0.040859888	0.829112406
NUCLEOSIDE DIPHOSPHATASE ACTIVITY	12	-0.084575072	0.714259572
ACETYLTRANSFERASE ACTIVITY	94	-0.054181641	0.516582798
PASSIVE TRANSMEMBRANE TRANSPORTER ACTIVITY	335	0.100121886	0.029234595
RNA POLYMERASE II CORE BINDING	17	-0.295239133	0.099481435
MRNA BINDING	150	-0.136905145	0.035072342
MHC CLASS II RECEPTOR ACTIVITY	9	-0.07369339	0.782835295
NEUROPEPTIDE RECEPTOR ACTIVITY	23	0.254811815	0.135472444
EXTRACELLULAR MATRIX BINDING	46	0.255607874	0.038745967
SEROTONIN RECEPTOR ACTIVITY	9	0.294766768	0.278817106
METALLOEXOPEPTIDASE ACTIVITY	38	0.030884115	0.817283232
TRNA BINDING	39	-0.370819293	0.001162886
CALCIUM ION BINDING	531	0.0399641	0.272377794
NITRIC OXIDE SYNTHASE BINDING	18	-0.185972262	0.310150129
PROTEIN C TERMINUS BINDING	173	-0.068255093	0.26751657
GLYCINE BINDING	9	-0.031634957	0.90696567
THIOLESTER HYDROLASE ACTIVITY	25	0.18923385	0.257002322
INSULIN RECEPTOR BINDING	28	-0.212981245	0.139348274
R SMAD BINDING	22	0.494345987	0.003234065
CIS TRANS ISOMERASE ACTIVITY	38	-0.146432935	0.250260924
METALLOCARBOXYPEPTIDASE ACTIVITY	17	0.107857686	0.5925372
ALDEHYDE DEHYDROGENASE NAD ACTIVITY	18	0.270260182	0.165730684
OXIDOREDUCTASE ACTIVITY ACTING ON PAIRED DONORS WITH INCORPORATION OR REDUCTION OF MOLECULAR OXYGEN 2 OXOGLUTARATE AS ONE DONOR AND INCORPORATION OF ONE ATOM EACH OF OXYGEN INTO BOTH DONORS	39	0.220798589	0.102833659
PHOSPHATE ION BINDING	9	-0.158993758	0.541613759
NUCLEOSIDE DIPHOSPHATE KINASE ACTIVITY	13	-0.067308245	0.762891916
FAD BINDING	10	-0.012781929	0.960490159
CLATHRIN BINDING	54	0.124861732	0.271763136
CATION CATION ANTIPORTER ACTIVITY	17	0.021211559	0.9152695
PROTEASE BINDING	92	0.094845265	0.275578089
RAN GTPASE BINDING	30	-0.295534502	0.028492175
ANTIPORTER ACTIVITY	53	-0.019811979	0.859660516
AMMONIUM TRANSMEMBRANE TRANSPORTER ACTIVITY	18	0.091716129	0.639335654
PHOSPHATASE ACTIVATOR ACTIVITY	11	-0.347962948	0.100073215
FATTY ACID BINDING	23	-0.085184419	0.609697153
AMINOPEPTIDASE ACTIVITY	34	-0.053714459	0.698313124
NADPH BINDING	14	-0.113247568	0.592215391
SINGLE STRANDED RNA BINDING	66	-0.192579208	0.045829286
CATECHOLAMINE BINDING	12	0.136091514	0.570402544
CALCIUM DEPENDENT PROTEIN BINDING	51	0.010674956	0.926041761

NUCLEOCYTOPLASMIC TRANSPORTER ACTIVITY	23	-0.146129862	0.379933355
GLUTATHIONE PEROXIDASE ACTIVITY	17	-0.066922133	0.731577766
PSEUDOURIDINE SYNTHASE ACTIVITY	12	0.02942078	0.901454759
G PROTEIN COUPLED RECEPTOR ACTIVITY	327	0.137041319	0.003217104
MAP KINASE ACTIVITY	13	0.129962363	0.572829099
NEUROPEPTIDE HORMONE ACTIVITY	18	0.218084716	0.260720066
CATION TRANSMEMBRANE TRANSPORTER ACTIVITY	468	0.043977418	0.256167018
CHANNEL REGULATOR ACTIVITY	107	0.160373936	0.047525693
INOSITOL TETRAKISPHOSPHATE PHOSPHATASE ACTIVITY	11	0.112654859	0.653585106
PHOSPHOLIPASE BINDING	16	-0.063404841	0.752782885
STRUCTURAL CONSTITUENT OF EYE LENS	12	0.240178346	0.309298928
DISULFIDE OXIDOREDUCTASE ACTIVITY	27	-0.220063603	0.134818844
GDP BINDING	49	0.213569588	0.078355253
PHOSPHORIC DIESTER HYDROLASE ACTIVITY	78	0.010850613	0.907194626
S100 PROTEIN BINDING	10	-0.477897818	0.017630944
KINASE ACTIVITY	737	0.029634011	0.340207129
TRANSCRIPTION FACTOR ACTIVITY RNA POLYMERASE II DISTAL ENHANCER SEQUENCE SPECIFIC BINDING	72	-0.115339669	0.219982535
CARBOHYDRATE DERIVATIVE TRANSPORTER ACTIVITY	33	-0.018623116	0.895629952
SERINE TYPE CARBOXYPEPTIDASE ACTIVITY	11	-0.07268916	0.763825267
HORMONE ACTIVITY	70	0.194478567	0.051312355
STEROID HORMONE RECEPTOR ACTIVITY	52	0.292057329	0.011767593
ANDROGEN RECEPTOR BINDING	36	-0.23343875	0.074699471
AMIDE TRANSMEMBRANE TRANSPORTER ACTIVITY	11	-0.216297589	0.343627005
MANGANESE ION BINDING	45	-0.095603911	0.422825153
RNA CAP BINDING	12	-0.182158239	0.415101782
SCAVENGER RECEPTOR ACTIVITY	32	0.218898286	0.135538438
CYCLIN DEPENDENT PROTEIN SERINE THREONINE KINASE INHIBITOR ACTIVITY	11	-0.434255898	0.039692596
ICOSANOID RECEPTOR ACTIVITY	12	0.380822151	0.091602144
METALLOENDOPEPTIDASE ACTIVITY	86	-0.133912293	0.116431208
PHOSPHATIDYLCHOLINE BINDING	13	0.128532067	0.577380362
TRANSLATION ELONGATION FACTOR ACTIVITY	16	-0.288106371	0.120614648
MONOSACCHARIDE TRANSMEMBRANE TRANSPORTER ACTIVITY	14	-0.044740991	0.836144732
HYDROLASE ACTIVITY ACTING ON ESTER BONDS	632	-0.0403163	0.221302747
CAMP BINDING	22	-0.083939681	0.622719227
GLYCOSAMINOGLYCAN BINDING	160	0.115747402	0.079865845
VITAMIN D RECEPTOR BINDING	17	-0.071853347	0.712283512
DAMAGED DNA BINDING	62	-0.066116652	0.518901467
PROTEIN SERINE THREONINE KINASE ACTIVATOR ACTIVITY	18	-0.187761828	0.304844525
PHOSPHOLIPID TRANSLOCATING ATPASE ACTIVITY	13	0.628706358	0.001648205
ALCOHOL BINDING	79	0.150287065	0.109017277
L ASCORBIC ACID BINDING	19	0.153184875	0.422153214
DIPEPTIDASE ACTIVITY	11	0.040727555	0.869902851
GLUTAMATE RECEPTOR ACTIVITY	19	0.044701762	0.81335107
GAMMA TUBULIN BINDING	22	-0.337869985	0.028515493
PEPTIDASE ACTIVATOR ACTIVITY	35	-0.143193564	0.283133654
POLY PURINE TRACT BINDING	19	-0.087114681	0.63703538
INTRONIC TRANSCRIPTION REGULATORY REGION DNA BINDING	11	0.382979517	0.10961783
HYDROLASE ACTIVITY ACTING ON ACID ANHYDRIDES	707	0.016804299	0.595045039
STEROL BINDING	37	0.286014411	0.033448452
RNA POLYMERASE II TRANSCRIPTION FACTOR ACTIVITY SEQUENCE SPECIFIC DNA BINDING	534	0.058993916	0.105535304
PHOSPHATIDYLINOSITOL BINDING	175	-0.023537514	0.703647559
TRANSFORMING GROWTH FACTOR BETA BINDING	15	0.185150185	0.3910828
SH3 DOMAIN BINDING	100	0.062740501	0.450322079
LIGASE ACTIVITY FORMING CARBON SULFUR BONDS	32	0.106348487	0.469514387
MANNOSE BINDING	13	0.352524538	0.114855639
RNA POLYMERASE II REPRESSING TRANSCRIPTION FACTOR BINDING	26	0.072777835	0.654656161
CATION SUGAR SYMPORTER ACTIVITY	13	-0.169069173	0.431757742
PROTEIN SERINE THREONINE PHOSPHATASE ACTIVITY	61	-0.103288779	0.311707598
TRANSCRIPTION FACTOR ACTIVITY RNA POLYMERASE II TRANSCRIPTION FACTOR BINDING	123	0.218125871	0.004258125
AMINOACYL TRNA EDITING ACTIVITY	10	-0.455632667	0.040659472
FOLIC ACID BINDING	7	0.168405606	0.592156424
E BOX BINDING	26	0.077268334	0.635587144

GLUCOSE BINDING	12	-0.0950467	0.679928373
NEUROTRANSMITTER SODIUM SYMPORTER ACTIVITY	12	0.047710407	0.84132538
NUCLEASE ACTIVITY	181	-0.040803696	0.50070993
DIPEPTIDYL PEPTIDASE ACTIVITY	10	0.314744108	0.221793552
TOXIC SUBSTANCE BINDING	9	-0.022927432	0.932648714
ESTRADIOL 17 BETA DEHYDROGENASE ACTIVITY	10	0.391615525	0.114072457
GDP DISSOCIATION INHIBITOR ACTIVITY	9	-0.174530803	0.503693106
POLYSACCHARIDE BINDING	13	0.41451285	0.05897684
SNAP RECEPTOR ACTIVITY	35	-0.092149969	0.495050131
MICROTUBULE SEVERING ATPASE ACTIVITY	9	-0.463999521	0.059366136
RRNA METHYLTRANSFERASE ACTIVITY	12	0.124255763	0.605794069
NON MEMBRANE SPANNING PROTEIN TYROSINE KINASE ACTIVITY	40	-0.262210446	0.029157615
H4 HISTONE ACETYLTRANSFERASE ACTIVITY	16	0.014346688	0.944266735
HISTONE DEMETHYLASE ACTIVITY	24	-0.031175714	0.851025639
EXONUCLEASE ACTIVITY	74	-0.059003003	0.53005855
MACROLIDE BINDING	16	-0.01466267	0.942684182
INSULIN LIKE GROWTH FACTOR BINDING	23	0.135335981	0.436194561
O METHYLTRANSFERASE ACTIVITY	17	-0.04141148	0.833121987
TROPOMYOSIN BINDING	10	0.191723494	0.464117254
AMINO ACID TRANSMEMBRANE TRANSPORTER ACTIVITY	63	0.037362641	0.719489138
LYSOZYME ACTIVITY	2	0.219468308	0.704933627
ANION CHANNEL ACTIVITY	72	0.152728857	0.121100166
STRUCTURAL CONSTITUENT OF NUCLEAR PORE	14	-0.245036257	0.231097928
NUCLEOTIDASE ACTIVITY	14	-0.06266953	0.771148634
ION ANTIPORTER ACTIVITY	32	-0.107828409	0.442161176
PHOSPHORIC ESTER HYDROLASE ACTIVITY	330	-0.072935273	0.103350901
SIGNALING RECEPTOR ACTIVITY	729	0.128023413	5.23E-05
SUGAR TRANSMEMBRANE TRANSPORTER ACTIVITY	25	-0.177491513	0.251094466
INTERLEUKIN 1 RECEPTOR BINDING	13	0.227144042	0.323039559
PROTEIN TRANSPORTER ACTIVITY	91	-0.063004037	0.45704848
IMMUNOGLOBULIN RECEPTOR BINDING	6	0.082860451	0.806526472
RECEPTOR ACTIVATOR ACTIVITY	26	0.294893007	0.073286141
ALCOHOL DEHYDROGENASE NADP ACTIVITY	15	0.062319971	0.770452756
NEUROTRANSMITTER RECEPTOR ACTIVITY	41	0.028422261	0.825135865
DIACYLGLYCEROL BINDING	10	-0.228008693	0.342558171
PHOSPHOLIPASE INHIBITOR ACTIVITY	11	-0.170699867	0.463172971
ACTININ BINDING	23	-0.087351456	0.601314382
PROTEIN SERINE THREONINE KINASE INHIBITOR ACTIVITY	28	-0.381914285	0.004717832
OXIDOREDUCTASE ACTIVITY ACTING ON A SULFUR GROUP OF DONORS DISULFIDE AS ACCEPTOR	11	0.171201416	0.497764918
TRANSCRIPTION COFACTOR BINDING	22	-1.08E-05	0.999950842
HSP90 PROTEIN BINDING	25	0.088369936	0.595038718
AMINE BINDING	9	0.571711174	0.012938449
DIOXYGENASE ACTIVITY	74	0.090765261	0.349042535
PHOSPHOTRANSFERASE ACTIVITY FOR OTHER SUBSTITUTED PHOSPHATE GROUPS	19	0.067821114	0.721194165
PALMITOYLTRANSFERASE ACTIVITY	34	-0.069335136	0.615238222
NUCLEAR LOCALIZATION SEQUENCE BINDING	20	-0.289427619	0.079884438
RECEPTOR AGONIST ACTIVITY	12	0.087821594	0.714204872
PHOSPHATASE BINDING	149	0.120289712	0.080187939
RNA DIRECTED DNA POLYMERASE ACTIVITY	7	-0.579847385	0.010075417
RAS GUANYL NUCLEOTIDE EXCHANGE FACTOR ACTIVITY	193	0.067275343	0.261996474
PROTEIN METHYLTRANSFERASE ACTIVITY	72	-0.049508533	0.604326051
LYSOPHOSPHOLIPID ACYLTRANSFERASE ACTIVITY	19	-0.134795887	0.45676964
STRUCTURAL CONSTITUENT OF MUSCLE	34	-0.018653805	0.893894802
ENZYME ACTIVATOR ACTIVITY	399	0.013532346	0.745060638
SH2 DOMAIN BINDING	26	-0.035753838	0.822344705
CARBON CARBON LYASE ACTIVITY	43	0.077962815	0.537703994
PROTEIN COMPLEX BINDING	825	-0.033982102	0.242822617
TRANSFERASE ACTIVITY TRANSFERRING ACYL GROUPS	213	-0.036814791	0.510722004
FATTY ACID TRANSPORTER ACTIVITY	10	0.361791117	0.144113941
PROTEIN TYROSINE SERINE THREONINE PHOSPHATASE ACTIVITY	40	-0.188210563	0.129825289
NUCLEOSIDE KINASE ACTIVITY	11	-0.114582309	0.63121169
NUCLEIC ACID BINDING TRANSCRIPTION FACTOR ACTIVITY	1025	-0.001093091	0.967046979
FIBROBLAST GROWTH FACTOR BINDING	21	0.238772152	0.190228659
ADP BINDING	33	-0.179093572	0.189594659

BETA 1 3 GALACTOSYLTRANSFERASE ACTIVITY	14	0.098510156	0.657344038
CATION TRANSPORTING ATPASE ACTIVITY	51	-0.038106685	0.737579908
ATPASE COUPLED ION TRANSMEMBRANE TRANSPORTER ACTIVITY	60	-0.118739199	0.24687686
STEROL TRANSPORTER ACTIVITY	13	0.544979655	0.00784146
RNA POLYMERASE BINDING	34	-0.135979553	0.314648127
CARBOHYDRATE KINASE ACTIVITY	18	-0.112291117	0.547965691
BINDING BRIDGING	146	0.009387657	0.890459685
THREONINE TYPE PEPTIDASE ACTIVITY	19	-0.223534968	0.20082541
MONOOXYGENASE ACTIVITY	61	0.092450164	0.38488784
ADENOSINE DEAMINASE ACTIVITY	9	0.422740034	0.128315541
STRUCTURAL CONSTITUENT OF RIBOSOME	196	-0.204087927	0.00022887
14 3 3 PROTEIN BINDING	19	0.037780701	0.841688899
COPPER ION BINDING	45	0.115394984	0.352321218
MISMATCHED DNA BINDING	12	-0.033274875	0.887161265
HYDROLASE ACTIVITY ACTING ON GLYCOSYL BONDS	93	0.121402514	0.160270546
RAB GTPASE BINDING	107	0.007018759	0.92963235
NAD ADP RIBOSYLTRANSFERASE ACTIVITY	24	-0.303880336	0.046637523
GUANYL NUCLEOTIDE EXCHANGE FACTOR ACTIVITY	261	0.026563858	0.604925186
TELOMERASE RNA BINDING	13	-0.363285344	0.062801554
BETA TUBULIN BINDING	33	-0.407879935	0.001419372
INTRAMOLECULAR TRANSFERASE ACTIVITY	25	0.070760526	0.669521609
SERINE TYPE ENDOPEPTIDASE INHIBITOR ACTIVITY	66	0.149509895	0.144592365
HELICASE ACTIVITY	140	-0.054834453	0.423889572
RNA POLYMERASE CORE ENZYME BINDING	21	-0.191821626	0.254334134
VOLTAGE GATED CHLORIDE CHANNEL ACTIVITY	8	0.623444187	0.01125361
PYRIDOXAL PHOSPHATE BINDING	42	0.037451437	0.768639739
LIPASE INHIBITOR ACTIVITY	15	-0.019677317	0.9254734
CHROMATIN DNA BINDING	76	-0.007815056	0.933678384
ATPASE BINDING	69	-0.228321972	0.012933635
LAMIN BINDING	17	-0.383573072	0.037251655
PHOSPHATIDYLINOSITOL KINASE ACTIVITY	44	0.057736556	0.643671854
CYCLIC NUCLEOTIDE BINDING	30	0.038944839	0.795955962
CYSTEINE TYPE ENDOPEPTIDASE ACTIVITY	73	-0.106271898	0.254950931
OLIGOPEPTIDE BINDING	11	-0.194488113	0.399660156
CO SMAD BINDING	11	0.201560116	0.425080924
GROWTH FACTOR ACTIVITY	120	0.179547924	0.018718382
INTRACELLULAR CALCIUM ACTIVATED CHLORIDE CHANNEL ACTIVITY	13	0.127072293	0.584749699
G PROTEIN COUPLED PHOTORECEPTOR ACTIVITY	5	-0.275472241	0.402829043
ALDO KETO REDUCTASE NADP ACTIVITY	23	0.071671201	0.678137704
TRANSFERASE ACTIVITY TRANSFERRING ACYL GROUPS OTHER THAN AMINO ACYL GROUPS	188	-0.042568527	0.473899412
MOLECULAR FUNCTION REGULATOR	1126	0.024950839	0.327009239
CYSTEINE TYPE ENDOPEPTIDASE INHIBITOR ACTIVITY INVOLVED IN APOPTOTIC PROCESS	20	-0.084082111	0.63838723
FIBRONECTIN BINDING	21	0.022685814	0.899409486
POTASSIUM TRANSPORTING ATPASE ACTIVITY	8	0.141043263	0.633388837
MICROTUBULE MOTOR ACTIVITY	58	-0.055334402	0.602222668
OXIDOREDUCTASE ACTIVITY OXIDIZING METAL IONS	15	0.021819709	0.918120396
ALPHA TUBULIN BINDING	19	-0.362020635	0.043650542
EXCITATORY EXTRACELLULAR LIGAND GATED ION CHANNEL ACTIVITY	31	-0.053615774	0.711718135
OXIDOREDUCTASE ACTIVITY OXIDIZING METAL IONS NAD OR NADP AS ACCEPTOR	10	0.182438132	0.486248898
ATPASE ACTIVITY COUPLED TO MOVEMENT OF SUBSTANCES	101	0.10176903	0.220643221
TRANSITION METAL ION TRANSMEMBRANE TRANSPORTER ACTIVITY	34	-0.050513191	0.715805604
DOUBLE STRANDED DNA BINDING	673	-0.039726926	0.214410127
TRANSITION METAL ION BINDING	1178	0.005146399	0.835899847
TELOMERIC DNA BINDING	28	-0.078651394	0.60479883
MODIFIED AMINO ACID TRANSMEMBRANE TRANSPORTER ACTIVITY	12	-0.119945749	0.599623539
SCAFFOLD PROTEIN BINDING	40	-0.060519297	0.635297083
TRANSCRIPTIONAL REPRESSOR ACTIVITY RNA POLYMERASE II ACTIVATING TRANSCRIPTION FACTOR BINDING	50	0.442699521	8.52E-05
LOW DENSITY LIPOPROTEIN PARTICLE RECEPTOR BINDING	15	-0.041227686	0.843842903
PHOSPHATIDYLSERINE BINDING	29	-0.200462776	0.161402267
GLYCOLIPID BINDING	15	-0.149295966	0.461212396
RECEPTOR SIGNALING PROTEIN SERINE THREONINE KINASE ACTIVITY	89	0.05033532	0.566539325
CALMODULIN BINDING	154	0.016465602	0.804390704

RNA POLYMERASE II CORE PROMOTER SEQUENCE SPECIFIC DNA BINDING	47	-0.083118594	0.477688583
MAP KINASE KINASE KINASE ACTIVITY	21	0.083371114	0.646035771
MHC CLASS II PROTEIN COMPLEX BINDING	16	0.034599225	0.866640717
PHOSPHOTYROSINE BINDING	11	0.253329811	0.329673236
NUCLEOSIDE TRIPHOSPHATASE REGULATOR ACTIVITY	284	-0.022982846	0.637382471
LIGAND GATED CALCIUM CHANNEL ACTIVITY	16	-0.25737712	0.165627332
RIBOSOMAL SMALL SUBUNIT BINDING	12	-0.061655306	0.791174548
OMEGA PEPTIDASE ACTIVITY	13	0.368819978	0.097717703
PHOSPHATIDYLCHOLINE 1 ACYLHYDROLASE ACTIVITY	10	0.362728795	0.150025091
ELECTRON CARRIER ACTIVITY	95	-0.105996558	0.195192024
HMG BOX DOMAIN BINDING	15	0.237503187	0.26637023
CHANNEL INHIBITOR ACTIVITY	32	0.112188492	0.445753917
SUMO BINDING	13	-0.009525491	0.966454897
PALMITOYL COA HYDROLASE ACTIVITY	8	0.326218074	0.253992639
MANNOSYLTRANSFERASE ACTIVITY	21	0.009642549	0.957039013
RECEPTOR SERINE THREONINE KINASE BINDING	14	0.160228784	0.476266522
RETINAL BINDING	6	0.383903957	0.225308426
TRANSFERASE ACTIVITY TRANSFERRING GLYCOSYL GROUPS	232	-0.095578455	0.07062076
TRANSFORMING GROWTH FACTOR BETA RECEPTOR BINDING	38	0.247918564	0.066344378
WW DOMAIN BINDING	26	0.044277792	0.784621994
PEPTIDE N ACETYLTRANSFERASE ACTIVITY	63	0.02454867	0.813031618
GLUCOCORTICOID RECEPTOR BINDING	13	-0.190062832	0.377133745
LIGASE REGULATOR ACTIVITY	13	-0.155966387	0.473553085
ACTIVE TRANSMEMBRANE TRANSPORTER ACTIVITY	271	-0.018207812	0.71544732
CAMP RESPONSE ELEMENT BINDING	13	-0.419412619	0.032556701
PROTEIN DISULFIDE OXIDOREDUCTASE ACTIVITY	21	-0.241212065	0.144931001
PHOSPHATIDYLINOSITOL BISPHOSPHATE BINDING	62	-0.150978767	0.129535863
CALCIUM ION TRANSMEMBRANE TRANSPORTER ACTIVITY	103	0.027966299	0.730971623
INTERMEDIATE FILAMENT BINDING	11	-0.007373732	0.976119537
AMIDE BINDING	208	-0.083295084	0.1364711
LIPOPOLYSACCHARIDE BINDING	13	0.201338025	0.382704567
LIGAND DEPENDENT NUCLEAR RECEPTOR BINDING	20	0.214608273	0.256762084
POLYPEPTIDE N ACETYLGALACTOSAMINYLTRANSFERASE ACTIVITY	15	0.043892069	0.836830945
MODIFIED AMINO ACID BINDING	54	-0.147627188	0.166998135
ION CHANNEL BINDING	98	-0.183685672	0.019641257
FILAMIN BINDING	10	0.31475433	0.215275188
PROTEOGLYCAN BINDING	25	0.200422631	0.230586589
SUMO TRANSFERASE ACTIVITY	15	-0.364175697	0.046662162
MYOSIN HEAVY CHAIN BINDING	9	0.002955828	0.991359128
LRR DOMAIN BINDING	15	0.509714868	0.017057117
TRANSFERASE ACTIVITY TRANSFERRING AMINO ACYL GROUPS	14	0.232464096	0.288540053
INTEGRIN BINDING	98	-0.117125197	0.145451441
ALCOHOL TRANSMEMBRANE TRANSPORTER ACTIVITY	14	0.161247075	0.468500533
CALMODULIN DEPENDENT PROTEIN KINASE ACTIVITY	26	-0.130386879	0.40030189
MICROFILAMENT MOTOR ACTIVITY	17	0.174605478	0.388034082
THIOL DEPENDENT UBIQUITIN SPECIFIC PROTEASE ACTIVITY	67	-0.122119238	0.209982861
FERROUS IRON BINDING	20	0.076002935	0.681623144
ACETYLCHOLINE BINDING	9	-0.058700972	0.827041766
TRANSCRIPTION FACTOR ACTIVITY RNA POLYMERASE II CORE PROMOTER PROXIMAL REGION SEQUENCE SPECIFIC BINDING	289	0.050980944	0.298905541
INORGANIC CATION TRANSMEMBRANE TRANSPORTER ACTIVITY	403	0.050748518	0.223525998
NAD BINDING	49	0.071886209	0.544392034
ANION TRANSMEMBRANE TRANSPORTER ACTIVITY	231	-0.004505219	0.933738336
CHAPERONE BINDING	72	-0.139778993	0.132025039
PROTEIN TYROSINE PHOSPHATASE ACTIVITY	95	-0.143242894	0.077179842
IDENTICAL PROTEIN BINDING	1032	-0.001150757	0.965199697
GALACTOSYLTRANSFERASE ACTIVITY	28	-0.200576134	0.17023501
PHOSPHOTRANSFERASE ACTIVITY PHOSPHATE GROUP AS ACCEPTOR	35	0.079558913	0.570774445
ALKALI METAL ION BINDING	14	-0.102295251	0.630309057
RIBONUCLEOPROTEIN COMPLEX BINDING	90	-0.154605528	0.062266473
DNA APURINIC OR APYRIMIDINIC SITE LYASE ACTIVITY	14	0.400647261	0.058656806
NEUROPILIN BINDING	14	0.077166058	0.727771753
PEPTIDE ANTIGEN BINDING	22	-0.300310559	0.056040432
DYNEIN BINDING	23	0.256183043	0.154711375
TRANSCRIPTIONAL REPRESSOR ACTIVITY RNA POLYMERASE II TRANSCRIPTION FACTOR BINDING	78	0.3563342	0.000139145

RAGE RECEPTOR BINDING	10	-0.220868982	0.357004246
TRANSLATION FACTOR ACTIVITY RNA BINDING	82	-0.272706574	0.001096524
LOW DENSITY LIPOPROTEIN RECEPTOR ACTIVITY	13	0.499851119	0.029759605
CGMP BINDING	10	0.120695335	0.645642117
DRUG BINDING	93	-0.066395084	0.427551279
SPECTRIN BINDING	20	-0.113213547	0.523927373
O ACYLTRANSFERASE ACTIVITY	41	-0.066563678	0.596744822
TRANSFERASE ACTIVITY TRANSFERRING NITROGENOUS GROUPS	21	0.000792882	0.996459126
TRANSLATION INITIATION FACTOR BINDING	26	-0.137146609	0.376821787
BILE ACID TRANSMEMBRANE TRANSPORTER ACTIVITY	9	0.175051397	0.526608482
PEPTIDASE ACTIVATOR ACTIVITY INVOLVED IN APOPTOTIC PROCESS	19	-0.220647922	0.207797643
METAL CLUSTER BINDING	54	0.014794319	0.894783692
ENZYME INHIBITOR ACTIVITY	293	-0.00421387	0.93035898
OXYGEN BINDING	30	0.119723538	0.430224078
ENDODEOXYRIBONUCLEASE ACTIVITY	46	0.001083198	0.992847009
CARBON NITROGEN LIGASE ACTIVITY WITH GLUTAMINE AS AMIDO N DONOR	11	-0.203912246	0.379321617
OXIDOREDUCTASE ACTIVITY ACTING ON THE CH NH GROUP OF DONORS NAD OR NADP AS ACCEPTOR	16	-0.033216106	0.870068083
ORGANIC ACID TRANSMEMBRANE TRANSPORTER ACTIVITY	110	-0.056326817	0.46570816
LONG CHAIN FATTY ACID COA LIGASE ACTIVITY	10	0.030819595	0.905767126
DEATH RECEPTOR BINDING	18	-0.065641461	0.729655956
SUPEROXIDE GENERATING NADPH OXIDASE ACTIVITY	6	0.363420858	0.268489454
INTRAMOLECULAR OXIDOREDUCTASE ACTIVITY TRANSPOSING S S BONDS	20	-0.218338467	0.208008923
RHO GUANYL NUCLEOTIDE EXCHANGE FACTOR ACTIVITY	65	-0.083109577	0.403662817
BICARBONATE TRANSMEMBRANE TRANSPORTER ACTIVITY	11	-0.279363268	0.205411541
HORMONE BINDING	42	0.081836693	0.523266218
INOSITOL PHOSPHATE PHOSPHATASE ACTIVITY	20	0.208523762	0.261950865
3 5 DNA HELICASE ACTIVITY	11	0.234585108	0.34577854
FOUR WAY JUNCTION DNA BINDING	15	-0.250694766	0.19615502
ISOPRENOID BINDING	20	0.313914744	0.087760945
PROTEIN KINASE A CATALYTIC SUBUNIT BINDING	14	-0.104610859	0.623534321
INOSITOL TRISPHOSPHATE KINASE ACTIVITY	10	0.547268788	0.03968407
MHC CLASS I PROTEIN BINDING	14	-0.02361748	0.913577526
ORGANIC ACID BINDING	163	-0.044001068	0.490138023
GABA RECEPTOR BINDING	14	-0.196786161	0.344756433
CARBOXYLIC ESTER HYDROLASE ACTIVITY	96	0.067417977	0.426454424
CARBON SULFUR LYASE ACTIVITY	10	-0.061841575	0.808181526
CARRECEPTOR ACTIVITY	50	0.245057288	0.037374442
LIPASE ACTIVATOR ACTIVITY	10	-0.139773362	0.575566846
SOLUTE CATION ANTIPORTER ACTIVITY	20	0.018485548	0.919857684
GLUCURONOSYLTRANSFERASE ACTIVITY	21	0.057093772	0.751742603
ANNEALING ACTIVITY	12	0.37195088	0.110208698
ION GATED CHANNEL ACTIVITY	31	0.153806597	0.305798418
HYDROGEN EXPORTING ATPASE ACTIVITY	25	0.009646201	0.953118452
MAP KINASE PHOSPHATASE ACTIVITY	14	-0.347190079	0.089658308
HEPARAN SULFATE PROTEOGLYCAN BINDING	15	0.263246623	0.217217028
ORGANIC ANION TRANSMEMBRANE TRANSPORTER ACTIVITY	137	-0.076628256	0.265850032
WIDE PORE CHANNEL ACTIVITY	19	0.007191956	0.969496256
DEOXYRIBONUCLEASE ACTIVITY	62	-0.122801289	0.222710496
RRNA BINDING	56	-0.128588047	0.225035478
OXIDOREDUCTASE ACTIVITY ACTING ON SINGLE DONORS WITH INCORPORATION OF MOLECULAR OXYGEN	21	0.091355003	0.614041233
ORGANIC ACID SODIUM SYMPORTER ACTIVITY	26	0.153079369	0.349691607
CARBOHYDRATE TRANSPORTER ACTIVITY	30	-0.148303077	0.299076966
VITAMIN BINDING	61	0.141226519	0.185435217
RAL GTPASE BINDING	13	0.391991055	0.077870325
CHEMOKINE ACTIVITY	33	0.230152298	0.108766635
UDP GLYCOSYLTRANSFERASE ACTIVITY	112	-0.018261071	0.813183663
GLUTATHIONE TRANSFERASE ACTIVITY	27	-0.034022417	0.827675716
OXIDOREDUCTASE ACTIVITY ACTING ON NAD P H OXYGEN AS ACCEPTOR	10	0.275671511	0.288075814
SULFOTRANSFERASE ACTIVITY	42	0.028342983	0.823569185
WNT ACTIVATED RECEPTOR ACTIVITY	19	-0.101242394	0.578992611
PHOSPHATIDYLINOSITOL 3 PHOSPHATE BINDING	29	-0.162324492	0.261089014
LYASE ACTIVITY	153	0.046623384	0.486822707
CHOLESTEROL TRANSPORTER ACTIVITY	8	0.711446444	0.000886414

TRANSCRIPTIONAL ACTIVATOR ACTIVITY RNA POLYMERASE II CORE PROMOTER PROXIMAL REGION SEQUENCE SPECIFIC BINDING	201	0.092033257	0.119232225
SINGLE STRANDED DNA DEPENDENT ATPASE ACTIVITY	12	-0.287425892	0.190582018
N ACYLTRANSFERASE ACTIVITY	94	-0.031948685	0.703726943
PROTEIN PHOSPHATASE 1 BINDING	16	0.016044744	0.937709358
CHEMOATTRACTANT ACTIVITY	24	-0.140724317	0.378135749
CORECEPTOR ACTIVITY	31	0.015135115	0.918293329
QUATERNARY AMMONIUM GROUP BINDING	25	-0.029263693	0.857187591
VOLTAGE GATED POTASSIUM CHANNEL ACTIVITY	62	0.262525028	0.012521694
CYCLIN DEPENDENT PROTEIN KINASE ACTIVITY	28	0.014359297	0.926290051
TRANSMEMBRANE TRANSPORTER ACTIVITY	767	0.012285494	0.686007513
CHEMOKINE BINDING	18	0.073663231	0.706030531
PEPTIDE HORMONE BINDING	20	0.062350154	0.736210157
KINESIN BINDING	30	-0.193550312	0.171056612
PHOSPHATIDYLINOSITOL 3 KINASE ACTIVITY	64	0.21932068	0.035216582
UBIQUITIN LIKE PROTEIN CONJUGATING ENZYME BINDING	32	0.325953665	0.025625241
STEROID HORMONE RECEPTOR BINDING	74	-0.100673736	0.279632739
BITTER TASTE RECEPTOR ACTIVITY	7	0.208971136	0.504415858
PROTEIN KINASE C ACTIVITY	16	0.027823026	0.892434982
AU RICH ELEMENT BINDING	22	-0.097643162	0.565948759
UBIQUITIN LIKE PROTEIN LIGASE BINDING	244	-0.093350174	0.070810859
LIPOPROTEIN PARTICLE RECEPTOR ACTIVITY	15	0.438066599	0.041228721
RNA HELICASE ACTIVITY	62	-0.152839082	0.126213252
CYCLIN DEPENDENT PROTEIN SERINE THREONINE KINASE REGULATOR ACTIVITY	26	-0.391904388	0.005291019
CCR CHEMOKINE RECEPTOR BINDING	22	0.256313113	0.141681388
GABA RECEPTOR ACTIVITY	17	0.101258589	0.614847989
DNA DEPENDENT ATPASE ACTIVITY	71	-0.138553127	0.139537425
METALLOPEPTIDASE ACTIVITY	144	-0.071060716	0.291044447
UBIQUITIN LIKE PROTEIN LIGASE ACTIVITY	184	-0.040023385	0.505678509
OXIDOREDUCTASE ACTIVITY ACTING ON THE CH CH GROUP OF DONORS NAD OR NADP AS ACCEPTOR	22	-0.103924358	0.539940452
GUANYL NUCLEOTIDE BINDING	337	0.048147046	0.289837854
PHOSPHATIDYLINOSITOL 3 KINASE BINDING	28	-0.019334392	0.900127848
ENDONUCLEASE ACTIVITY	108	0.022371163	0.77801503
TRANSCRIPTIONAL REPRESSOR ACTIVITY RNA POLYMERASE II CORE PROMOTER PROXIMAL REGION SEQUENCE SPECIFIC BINDING	92	0.02355458	0.784088174
ANION ANION ANTIPORTER ACTIVITY	15	-0.242155253	0.207393702
TASTE RECEPTOR ACTIVITY	8	0.224908376	0.441027879
ORGANIC CATION TRANSMEMBRANE TRANSPORTER ACTIVITY	12	0.348411427	0.131016598
WATER TRANSMEMBRANE TRANSPORTER ACTIVITY	8	0.084440271	0.773112604
ACTIVIN BINDING	12	0.3964035	0.089763006
OXIDOREDUCTASE ACTIVITY ACTING ON THE CH NH2 GROUP OF DONORS	17	0.217509743	0.278525504
BETA AMYLOID BINDING	27	0.273714118	0.086059759
HYDROLASE ACTIVITY HYDROLYZING N GLYCOSYL COMPOUNDS	20	0.277327233	0.130944835
PROTEIN HORMONE RECEPTOR ACTIVITY	6	-0.238588405	0.436794343
CHEMOKINE RECEPTOR BINDING	42	0.278443524	0.027293551
PROLINE RICH REGION BINDING	18	0.213398544	0.282766134
VOLTAGE GATED SODIUM CHANNEL ACTIVITY	17	-0.032949104	0.867145052
OXIDOREDUCTASE ACTIVITY ACTING ON PAIRED DONORS WITH INCORPORATION OR REDUCTION OF MOLECULAR OXYGEN	113	0.091528449	0.243245794
TRANSCRIPTION FACTOR ACTIVITY PROTEIN BINDING	529	0.076667124	0.03683906
INTRAMOLECULAR TRANSFERASE ACTIVITY PHOSPHOTRANSFERASES	10	-0.087624186	0.729111615
SINGLE STRANDED DNA BINDING	81	-0.039865477	0.658934519
TYPE I INTERFERON RECEPTOR BINDING	6	0.276014143	0.407631258
RIBONUCLEOTIDE BINDING	1604	-0.020189556	0.348402449
TRANSCRIPTION COACTIVATOR BINDING	10	-0.05788734	0.820526751
RETINOIC ACID RECEPTOR BINDING	33	-0.113627685	0.412753275
PHOSPHATIDYLINOSITOL PHOSPHATE KINASE ACTIVITY	14	0.169510267	0.445789302
ACTIVATING TRANSCRIPTION FACTOR BINDING	53	0.044587413	0.694598209
NEUREXIN FAMILY PROTEIN BINDING	12	-0.089745735	0.697039634
MICROTUBULE BINDING	173	-0.127817221	0.034627083
TUBULIN BINDING	232	-0.122967326	0.019129191
ORGANOPHOSPHATE ESTER TRANSMEMBRANE TRANSPORTER ACTIVITY	21	-0.333931377	0.037274672
TRANSMEMBRANE RECEPTOR PROTEIN PHOSPHATASE ACTIVITY	17	0.087403067	0.664497537
NADP BINDING	38	-0.125342665	0.3289406

S ADENOSYLMETHIONINE DEPENDENT METHYLTRANSFERASE ACTIVITY	122	-0.0035591	0.96179712
KINASE ACTIVATOR ACTIVITY	53	0.156806879	0.173250638
HISTONE LYSINE N METHYLTRANSFERASE ACTIVITY	40	-0.076388082	0.548121236
INSULIN LIKE GROWTH FACTOR RECEPTOR BINDING	12	-0.456691272	0.020598388
MOTOR ACTIVITY	100	0.019289707	0.814905966
TRANSCRIPTION FACTOR BINDING	475	-0.048332053	0.200382548
TRNA SPECIFIC RIBONUCLEASE ACTIVITY	15	0.154988458	0.470329839
SOLUTE CATION SYMPORTER ACTIVITY	72	0.005640611	0.953510004
UBIQUITIN SPECIFIC PROTEASE BINDING	12	0.422372806	0.090493825
PHOSPHOLIPID TRANSPORTER ACTIVITY	42	0.398498124	0.001306528
RETINOID X RECEPTOR BINDING	16	-0.204310999	0.300255496
MONOCARBOXYLIC ACID TRANSMEMBRANE TRANSPORTER ACTIVITY	33	-0.127900626	0.352062845
ANION TRANSMEMBRANE TRANSPORTING ATPASE ACTIVITY	8	-0.46374603	0.047977336
POTASSIUM ION BINDING	9	-0.196664548	0.441329916
BASAL TRANSCRIPTION MACHINERY BINDING	25	-0.295018821	0.045600183
INTRACELLULAR LIGAND GATED ION CHANNEL ACTIVITY	23	-0.160340887	0.32259728
LIGASE ACTIVITY FORMING CARBON NITROGEN BONDS	52	-0.171047269	0.113563587
1 PHOSPHATIDYLINOSITOL BINDING	17	0.25076131	0.217384738
OXIDOREDUCTASE ACTIVITY ACTING ON PEROXIDE AS ACCEPTOR	34	-0.06439535	0.641106297
GROWTH FACTOR RECEPTOR BINDING	113	-0.040666387	0.595190343
PHOSPHOLIPASE A2 ACTIVITY	20	0.190012215	0.305276107
TITIN BINDING	13	0.030567888	0.89349579
NUCLEOBASE CONTAINING COMPOUND TRANSMEMBRANE TRANSPORTER ACTIVITY	30	-0.265153625	0.055245936
GTPASE ACTIVATING PROTEIN BINDING	13	-0.125481179	0.568099507
OXIDOREDUCTASE ACTIVITY ACTING ON THE CH NH2 GROUP OF DONORS OXYGEN AS ACCEPTOR	14	0.155087814	0.485219453
LIGAND DEPENDENT NUCLEAR RECEPTOR TRANSCRIPTION COACTIVATOR ACTIVITY	49	0.001321529	0.990994521
HSP70 PROTEIN BINDING	29	-0.051614751	0.731028296
SNRNA BINDING	34	-0.478514454	2.84E-05
ENZYME REGULATOR ACTIVITY	799	0.005528176	0.852676892
ARF GUANYL NUCLEOTIDE EXCHANGE FACTOR ACTIVITY	23	-0.159218856	0.331434959
CARBONATE DEHYDRATASE ACTIVITY	11	0.18251849	0.466937636
RECEPTOR SIGNALING COMPLEX SCAFFOLD ACTIVITY	18	0.362517741	0.066899004
GLYCOPROTEIN BINDING	92	0.036091301	0.675320267
MYOSIN V BINDING	17	0.245052577	0.228031055
EXOPEPTIDASE ACTIVITY	82	0.005012591	0.955916635
NUCLEOTIDE TRANSMEMBRANE TRANSPORTER ACTIVITY	18	-0.376428365	0.028550169
FUCOSYLTRANSFERASE ACTIVITY	9	-0.275477092	0.263037116
SOLUTE PROTON SYMPORTER ACTIVITY	19	-0.305093644	0.070030367
PLATELET DERIVED GROWTH FACTOR BINDING	10	-0.219173142	0.36227811
PHOSPHOTRANSFERASE ACTIVITY NITROGENOUS GROUP AS ACCEPTOR	9	-0.15316805	0.555973203
SNARE BINDING	107	0.013321915	0.867059272
GTP RHO BINDING	15	0.013899627	0.947705875
DELAYED RECTIFIER POTASSIUM CHANNEL ACTIVITY	23	0.226324961	0.19116508
NAD DEPENDENT PROTEIN DEACETYLASE ACTIVITY	15	-0.117061096	0.566725078
PHOSPHATIDIC ACID BINDING	13	-0.188290871	0.380912267
DICARBOXYLIC ACID TRANSMEMBRANE TRANSPORTER ACTIVITY	29	-0.052655191	0.725708438
TRANSLATION REGULATOR ACTIVITY	31	0.263269537	0.086327112
ACETYLGLUCOSAMINYLTRANSFERASE ACTIVITY	43	0.070368668	0.578391073
INTRAMOLECULAR OXIDOREDUCTASE ACTIVITY TRANSPOSING C C BONDS	9	-0.391748323	0.095548849
NF KAPPAB BINDING	29	-0.087933536	0.554164082
SODIUM INDEPENDENT ORGANIC ANION TRANSMEMBRANE TRANSPORTER ACTIVITY	13	-0.116991743	0.593681668
BIOACTIVE LIPID RECEPTOR ACTIVITY	10	-0.015358037	0.952507948
STORE OPERATED CALCIUM CHANNEL ACTIVITY	11	-0.238803358	0.29627034
CALCIUM DEPENDENT PROTEIN KINASE ACTIVITY	12	-0.11110863	0.627863876
MACROMOLECULE TRANSMEMBRANE TRANSPORTER ACTIVITY	19	-0.283643991	0.101334475
PEPTIDASE INHIBITOR ACTIVITY	123	0.094191736	0.209710024
RAC GTPASE BINDING	37	0.099027997	0.469024951
SULFURIC ESTER HYDROLASE ACTIVITY	16	-0.011114972	0.956571639
SECONDARY ACTIVE TRANSMEMBRANE TRANSPORTER ACTIVITY	170	-0.060586819	0.329854179
PEPTIDOGLYCAN BINDING	5	0.035394091	0.923442481
DRUG TRANSPORTER ACTIVITY	19	-0.09101627	0.619724094
HYALURONIC ACID BINDING	17	0.059205238	0.767839931

ENZYME BINDING	1585	-0.052155686	0.015428411
TRANSLATION REPRESSOR ACTIVITY	18	0.418069026	0.034959767
DNA DIRECTED DNA POLYMERASE ACTIVITY	25	0.018688292	0.90947939
COFACTOR BINDING	223	0.015333253	0.781803301
RNA POLYMERASE II ACTIVATING TRANSCRIPTION FACTOR BINDING	33	0.045338018	0.752707485
RNA POLYMERASE II TRANSCRIPTION COACTIVATOR ACTIVITY	35	0.537204518	1.51E-05
PEROXISOME PROLIFERATOR ACTIVATED RECEPTOR BINDING	13	-0.206972546	0.337209353
K63 LINKED POLYUBIQUITIN BINDING	17	-0.438340971	0.010796913
OXIDOREDUCTASE ACTIVITY ACTING ON CH OH GROUP OF DONORS	109	0.118617154	0.137766619
CULLIN FAMILY PROTEIN BINDING	12	0.219428054	0.356000389
PROTEIN TYROSINE KINASE BINDING	49	-0.147712931	0.187737395
INSULIN RECEPTOR SUBSTRATE BINDING	10	0.008893118	0.972636828
MONOVALENT CATION PROTON ANTIPORTER ACTIVITY	9	0.043684813	0.873795817
ZINC ION BINDING	992	-0.004442408	0.868574012
TETRAPYRROLE BINDING	94	0.045100153	0.597239167
3 5 EXONUCLEASE ACTIVITY	46	0.02717171	0.822983575
MITOGEN ACTIVATED PROTEIN KINASE KINASE KINASE BINDING	18	0.029920279	0.877466903
PDZ DOMAIN BINDING	84	0.061380485	0.497873827
POLY A RNA BINDING	1140	-0.151250874	7.12E-10
POTASSIUM CHANNEL REGULATOR ACTIVITY	35	0.180041622	0.203576225
EXTRACELLULAR LIGAND GATED ION CHANNEL ACTIVITY	45	-0.006830818	0.955328591
G PROTEIN COUPLED AMINE RECEPTOR ACTIVITY	26	0.195864491	0.228178823
CXCR CHEMOKINE RECEPTOR BINDING	13	0.302063814	0.183720415
IRON ION BINDING	114	0.123446247	0.114253094
STEROID HYDROXYLASE ACTIVITY	18	0.071645415	0.713597918
SIGNALING ADAPTOR ACTIVITY	61	0.055726577	0.599277879
G PROTEIN BETA GAMMA SUBUNIT COMPLEX BINDING	18	0.026991376	0.889348531
DOPAMINE BINDING	7	0.036574741	0.906501011
SH3 SH2 ADAPTOR ACTIVITY	41	0.120259354	0.356527405
RETINOL BINDING	6	0.130663515	0.69951615
INORGANIC ANION EXCHANGER ACTIVITY	15	0.012365628	0.953456997
RETINOL DEHYDROGENASE ACTIVITY	12	0.094384296	0.693458293
PHOSPHOLIPASE C ACTIVITY	28	0.15128119	0.337985434
NUCLEOSOMAL DNA BINDING	29	-0.205000627	0.153394074
LIPASE ACTIVITY	91	0.159482933	0.06928698
PROTEIN BINDING INVOLVED IN CELL CELL ADHESION	10	-0.087925045	0.728311256
PHOSPHATIDYLINOSITOL 3 4 BISPHOSPHATE BINDING	16	-0.016020862	0.937365955
ACTIN DEPENDENT ATPASE ACTIVITY	11	0.24511766	0.329274783
PROTEIN SERINE THREONINE KINASE ACTIVITY	404	0.045849951	0.270628713
METHYL CPG BINDING	18	0.126713439	0.520681896
TRANSLATION REGULATOR ACTIVITY NUCLEIC ACID BINDING	15	0.4342101	0.067929237
RECEPTOR ACTIVITY	932	0.124977331	9.36E-06
ACTIN MONOMER BINDING	22	-0.083525175	0.625037783
REPRESSING TRANSCRIPTION FACTOR BINDING	53	-0.032995491	0.767729709
POLYOL TRANSMEMBRANE TRANSPORTER ACTIVITY	8	0.048956072	0.866768392
CHROMATIN BINDING	400	0.059054169	0.159023165
MYOSIN BINDING	53	-0.063198511	0.568477111
HYDROLASE ACTIVITY ACTING ON CARBON NITROGEN BUT NOT PEPTIDE BONDS IN LINEAR AMIDES	72	-0.035503872	0.711032442
CHEMOREPELLENT ACTIVITY	25	0.293824486	0.079187828
MAP KINASE KINASE ACTIVITY	12	0.293644437	0.21626853
ANTIGEN BINDING	64	-0.127005675	0.199454361
PROTEIN DOMAIN SPECIFIC BINDING	564	0.043912844	0.214848705
SODIUM CHANNEL REGULATOR ACTIVITY	28	0.308506681	0.045384476
POLY A SPECIFIC RIBONUCLEASE ACTIVITY	12	0.012600591	0.957574835
OXIDOREDUCTASE ACTIVITY ACTING ON THE ALDEHYDE OR OXO GROUP OF DONORS	39	-0.026609919	0.838260254
DOUBLE STRANDED RNA BINDING	63	-0.228354466	0.018330299
MHC PROTEIN BINDING	22	0.097757749	0.582631052
POTASSIUM CHANNEL ACTIVITY	80	0.21048927	0.023852805
FATTY ACID LIGASE ACTIVITY	11	0.101275065	0.686032119
APOLIPOPROTEIN BINDING	13	0.507540156	0.014933197
LIPID BINDING	527	0.060009354	0.101736635
HISTONE ACETYLTRANSFERASE BINDING	26	0.218333638	0.187871603
STRUCTURAL CONSTITUENT OF CYTOSKELETON	82	-0.231916521	0.005990268
IGG BINDING	5	0.235833388	0.518674694

2 IRON 2 SULFUR CLUSTER BINDING	18	-0.166029001	0.36481986
ALPHA ACTININ BINDING	17	-0.11440694	0.55379148
OXIDOREDUCTASE ACTIVITY ACTING ON A SULFUR GROUP OF DONORS	44	-0.299740452	0.007229384
DEACETYLASE ACTIVITY	51	-0.084237677	0.453205569
TUMOR NECROSIS FACTOR RECEPTOR BINDING	28	-0.057157775	0.707953847
FLAVIN ADENINE DINUCLEOTIDE BINDING	61	0.019499369	0.853184408
TRANSFERASE ACTIVITY TRANSFERRING ONE CARBON GROUPS	192	-0.079287686	0.173701871
PROTEIN N TERMINUS BINDING	94	-0.007288868	0.931262999
ANGIOTENSIN RECEPTOR BINDING	12	0.396035141	0.096109901
INWARD RECTIFIER POTASSIUM CHANNEL ACTIVITY	17	0.359949025	0.073398271
GLUCOSYLTRANSFERASE ACTIVITY	16	-0.138366001	0.480794347
RIBONUCLEASE P ACTIVITY	10	0.166068184	0.527287153
P53 BINDING	64	-0.058565459	0.562145246
PROTON TRANSPORTING ATP SYNTHASE ACTIVITY ROTATIONAL MECHANISM	10	-0.257822881	0.277398994
PEPTIDE RECEPTOR ACTIVITY	87	0.101631549	0.254750026
SYMPORTER ACTIVITY	104	-0.041609847	0.601675238
FRIZZLED BINDING	29	0.047114604	0.75873755
RNA POLYMERASE II TRANSCRIPTION COFACTOR ACTIVITY	86	0.178399648	0.048915897
RNA POLYMERASE II CARBOXY TERMINAL DOMAIN KINASE ACTIVITY	16	-0.006226587	0.975690433
ENDORIBONUCLEASE ACTIVITY	45	0.01411906	0.908230473
NEUROTROPHIN RECEPTOR BINDING	12	0.156692437	0.51945025
UBIQUITIN LIKE PROTEIN SPECIFIC PROTEASE ACTIVITY	86	-0.089033924	0.30416146
PHOSPHOLIPID BINDING	305	0.043993447	0.356563753
C ACYLTRANSFERASE ACTIVITY	13	0.144419883	0.531503292
INTRAMOLECULAR OXIDOREDUCTASE ACTIVITY	45	-0.115567661	0.330357476
G PROTEIN COUPLED CHEMOATTRACTANT RECEPTOR ACTIVITY	20	0.046114815	0.802705101
SEQUENCE SPECIFIC DNA BINDING	873	-0.012943145	0.649165153
GTP DEPENDENT PROTEIN BINDING	15	0.253874525	0.240295039
TRANSCRIPTION COREPRESSOR ACTIVITY	197	0.128142331	0.03231469
DEATH RECEPTOR ACTIVITY	19	0.178223497	0.349719185
RIBONUCLEASE ACTIVITY	85	-0.015277767	0.863211098
MISFOLDED PROTEIN BINDING	11	-0.008387596	0.972831278
LAMININ BINDING	25	0.132178096	0.430690107
GLUTAMATE RECEPTOR BINDING	34	-0.167425981	0.209285735
CYTOKINE RECEPTOR ACTIVITY	71	0.119238885	0.229091272
ACID PHOSPHATASE ACTIVITY	8	0.214558662	0.465073859
ENDOPEPTIDASE ACTIVITY	304	-0.104392905	0.023768938
OXIDOREDUCTASE ACTIVITY ACTING ON PAIRED DONORS WITH INCORPORATION OR REDUCTION OF MOLECULAR OXYGEN REDUCED FLAVIN OR FLAVOPROTEIN AS ONE DONOR AND INCORPORATION OF ONE ATOM OF OXYGEN	14	0.105069306	0.635831521
ARYLSULFATASE ACTIVITY	11	0.113583542	0.650728335
NOTCH BINDING	18	-0.079415117	0.674914128
CATION AMINO ACID SYMPORTER ACTIVITY	10	0.166248344	0.531255345
DEAMINASE ACTIVITY	26	0.217508628	0.188018802
THYROID HORMONE RECEPTOR BINDING	30	-0.190756188	0.184896155
RECEPTOR SIGNALING PROTEIN ACTIVITY	162	-0.019740319	0.758978887
METALLOAMINOPEPTIDASE ACTIVITY	14	-0.142305984	0.497316071
GAP JUNCTION CHANNEL ACTIVITY	14	-0.045805864	0.83226034
DOPAMINE RECEPTOR BINDING	16	0.2182791	0.297329139
SIGNALING PATTERN RECOGNITION RECEPTOR ACTIVITY	14	0.144237816	0.516198724
NEUROPEPTIDE BINDING	11	0.17685911	0.48048213
HIGH VOLTAGE GATED CALCIUM CHANNEL ACTIVITY	8	0.294488155	0.301963972
GAMMA CATENIN BINDING	10	-0.286869362	0.214502566
CALCIUM DEPENDENT CYSTEINE TYPE ENDOPEPTIDASE ACTIVITY	18	0.19189827	0.324437973
TBP CLASS PROTEIN BINDING	18	-0.258854863	0.143555981
OXIDOREDUCTASE ACTIVITY ACTING ON NAD P H QUINONE OR SIMILAR COMPOUND AS ACCEPTOR	49	-0.094465483	0.407582948
TAU PROTEIN KINASE ACTIVITY	11	0.173947603	0.490622948
TRANSCRIPTION FACTOR ACTIVITY RNA POLYMERASE II CORE PROMOTER SEQUENCE SPECIFIC	13	0.130238665	0.575000791
RIBOSOME BINDING	45	-0.203294675	0.078260471
TRANSCRIPTION COACTIVATOR ACTIVITY	273	0.057521552	0.254873001
RECEPTOR REGULATOR ACTIVITY	34	0.225651584	0.117477368
LYSINE N METHYLTRANSFERASE ACTIVITY	51	0.055109058	0.634796584

SODIUM ION BINDING	10	-0.086918868	0.73101783
C C CHEMOKINE RECEPTOR ACTIVITY	11	0.28542254	0.24133183
OXIDOREDUCTASE ACTIVITY	585	-0.021869483	0.52421382
MONOSACCHARIDE BINDING	62	0.095470035	0.366164354
CORE PROMOTER PROXIMAL REGION DNA BINDING	323	-0.051310729	0.259416372
EPHRIN RECEPTOR ACTIVITY	14	0.233459787	0.287699384
ATPASE ACTIVITY	365	-0.037228995	0.38656073
GTPASE BINDING	271	0.026708325	0.596240892
PRE MRNA BINDING	24	-0.404603734	0.006297284
TRANSMEMBRANE RECEPTOR PROTEIN SERINE THREONINE KINASE ACTIVITY	16	-0.096322769	0.629498575
G PROTEIN COUPLED RECEPTOR BINDING	203	0.102949062	0.079557832
LIGASE ACTIVITY FORMING CARBON OXYGEN BONDS	42	-0.2744342	0.020097127
CARBON OXYGEN LYASE ACTIVITY	65	0.015831772	0.876671553
ADENYL NUCLEOTIDE BINDING	1307	-0.037238526	0.113321823
EXTRACELLULAR GLUTAMATE GATED ION CHANNEL ACTIVITY	15	0.115732056	0.589461085
SOLUTE SODIUM SYMPORTER ACTIVITY	38	0.135789437	0.315399969
PHOSPHATIDATE PHOSPHATASE ACTIVITY	9	0.058164498	0.833053784
TRANSFERASE ACTIVITY TRANSFERRING PENTOSYL GROUPS	52	-0.231092563	0.029642964
NUCLEOTIDE KINASE ACTIVITY	22	0.144581863	0.415675725
NEUROTRANSMITTER TRANSPORTER ACTIVITY	13	0.174617781	0.44986619
TRNA METHYLTRANSFERASE ACTIVITY	19	-0.204528843	0.245055497
HISTONE METHYLTRANSFERASE ACTIVITY H3 K4 SPECIFIC	16	0.106824572	0.607722386
CELL ADHESION MOLECULE BINDING	169	-0.16546606	0.006209467
RECEPTOR BINDING	1195	0.010072531	0.68348137
RECEPTOR INHIBITOR ACTIVITY	7	0.067348948	0.829677863
CADHERIN BINDING	27	-0.245006297	0.089392744
SEMAPHORIN RECEPTOR BINDING	21	0.148160638	0.417081008
METALLOENDOPEPTIDASE INHIBITOR ACTIVITY	12	-0.069941984	0.762899967
EPHRIN RECEPTOR BINDING	24	0.017606807	0.916403579
CHLORIDE CHANNEL REGULATOR ACTIVITY	13	0.312990973	0.174817145
OPSONIN BINDING	7	0.208316567	0.504653091
DNA HELICASE ACTIVITY	47	-0.035837594	0.762391747
AMMONIUM ION BINDING	40	0.037667491	0.772769851
RNA POLYMERASE II TRANSCRIPTION FACTOR BINDING	97	0.091277081	0.281094764
EXONUCLEASE ACTIVITY ACTIVE WITH EITHER RIBO OR DEOXYRIBONUCLEIC ACIDS AND PRODUCING 5 PHOSPHOMONOESTERS	44	-0.167986616	0.152603247
ASPARTIC TYPE PEPTIDASE ACTIVITY	17	-0.280249682	0.120470848
RNA POLYMERASE ACTIVITY	44	-0.021346406	0.862120312
PROTEIN PHOSPHATASE 2A BINDING	26	-0.089351587	0.569704871
MONOCARBOXYLIC ACID BINDING	45	0.01799101	0.883313289
ISOMERASE ACTIVITY	135	-0.083117024	0.230314983
HYDROLASE ACTIVITY ACTING ON CARBON NITROGEN BUT NOT PEPTIDE BONDS	120	0.006858558	0.927216654
PLATELET DERIVED GROWTH FACTOR RECEPTOR BINDING	15	-0.128958782	0.528064817
DNA POLYMERASE BINDING	12	0.239917699	0.314636261
NUCLEOBASE CONTAINING COMPOUND KINASE ACTIVITY	42	-0.032885044	0.793183907
TRANSPORTER ACTIVITY	990	0.035392072	0.191052021
VOLTAGE GATED CALCIUM CHANNEL ACTIVITY	34	0.229589386	0.107851266
CYTOKINE ACTIVITY	151	0.087300616	0.19816679
EXODEOXYRIBONUCLEASE ACTIVITY	16	-0.4274732	0.012465939
4 IRON 4 SULFUR CLUSTER BINDING	35	-0.010977274	0.936642372
OXIDOREDUCTASE ACTIVITY ACTING ON PAIRED DONORS WITH INCORPORATION OR REDUCTION OF MOLECULAR OXYGEN NAD P H AS ONE DONOR AND INCORPORATION OF ONE ATOM OF OXYGEN	29	-0.003456616	0.981849247
PHOSPHOLIPASE ACTIVITY	75	0.140770425	0.144632076
ARACHIDONIC ACID MONOOXYGENASE ACTIVITY	8	0.198782155	0.497267727
N ACETYLTRANSFERASE ACTIVITY	80	-0.034939319	0.700877521
HYDROLASE ACTIVITY HYDROLYZING O GLYCOSYL COMPOUNDS	70	0.078338193	0.42991896
MACROMOLECULAR COMPLEX BINDING	1249	-0.017638926	0.464048716
INTRAMOLECULAR OXIDOREDUCTASE ACTIVITY INTERCONVERTING ALDOSES AND KETOSES	11	0.543861667	0.014226949
ACID THIOL LIGASE ACTIVITY	15	-0.01851908	0.9298716
STRUCTURAL MOLECULE ACTIVITY	591	-0.098864533	0.003224999
HISTONE DEACETYLASE ACTIVITY H3 K14 SPECIFIC	10	-0.08931984	0.723802484
RNA BINDING	1502	-0.166073843	1.97E-14

ODORANT BINDING	8	0.550181238	0.026961139
CYSTEINE TYPE ENDOPEPTIDASE INHIBITOR ACTIVITY	42	0.023670029	0.852144106
PHOSPHATIDYLINOSITOL 3 4 5 TRISPHOSPHATE BINDING	31	-0.199643765	0.150132565
RNA POLYMERASE II DISTAL ENHANCER SEQUENCE SPECIFIC DNA BINDING	54	-0.344071865	0.000602086
CYTOKINE RECEPTOR BINDING	213	0.142664168	0.013344464
PHOSPHATASE REGULATOR ACTIVITY	77	-0.094618459	0.299672856
OXIDOREDUCTASE ACTIVITY ACTING ON THE CH NH GROUP OF DONORS	22	-0.006485259	0.970316749
TUMOR NECROSIS FACTOR RECEPTOR SUPERFAMILY BINDING	45	-0.039047546	0.746955603
PROTEIN CHANNEL ACTIVITY	10	-0.081450037	0.748004867
CORE PROMOTER SEQUENCE SPECIFIC DNA BINDING	93	-0.041672839	0.620936492
HISTONE KINASE ACTIVITY	18	-0.659990104	4.48E-06
ARMADILLO REPEAT DOMAIN BINDING	13	0.111805702	0.627921479
CATION CHANNEL ACTIVITY	208	0.122936836	0.034465525
RAB GUANYL NUCLEOTIDE EXCHANGE FACTOR ACTIVITY	29	-0.149992964	0.304379647
OXIDIZED NAD BINDING	12	0.141544908	0.55490126
GROWTH FACTOR BINDING	113	-0.026374009	0.731246257
CALCIUM CHANNEL REGULATOR ACTIVITY	32	0.142705842	0.332218569
THIOESTERASE BINDING	14	-0.266445053	0.181864229
INOSITOL TRISPHOSPHATE PHOSPHATASE ACTIVITY	11	0.116385421	0.642194534
TRANSFERASE ACTIVITY TRANSFERRING PHOSPHORUS CONTAINING GROUPS	876	0.014227287	0.618300569
HYDROLASE ACTIVITY ACTING ON ETHER BONDS	9	0.096650126	0.726972725
ACYL COA DEHYDROGENASE ACTIVITY	14	0.158480952	0.476175138
LIGASE ACTIVITY	377	-0.006166866	0.884930093
HEXOSAMINIDASE ACTIVITY	14	0.23683376	0.280914798
MRNA 3 UTR BINDING	47	-0.078818293	0.501885639
PHOSPHATIDYLINOSITOL 4 PHOSPHATE BINDING	19	0.136857044	0.474762959
CYSTEINE TYPE ENDOPEPTIDASE ACTIVITY INVOLVED IN APOPTOTIC PROCESS	12	-0.240912978	0.273434101
SODIUM CHANNEL ACTIVITY	27	0.061223078	0.700701294
STRUCTURE SPECIFIC DNA BINDING	111	-0.01369209	0.860117687
COENZYME BINDING	152	0.014630819	0.82685716
SNORNA BINDING	25	-0.286244207	0.053030523
SODIUM ION TRANSMEMBRANE TRANSPORTER ACTIVITY	98	0.080130794	0.340177061
NUCLEOTIDYLTRANSFERASE ACTIVITY	120	-0.071664266	0.330682524
CALCIUM DEPENDENT PHOSPHOLIPID BINDING	43	0.039666348	0.752672456
CYSTEINE TYPE ENDOPEPTIDASE REGULATOR ACTIVITY INVOLVED IN APOPTOTIC PROCESS	37	-0.144630102	0.262471256
HISTONE METHYLTRANSFERASE ACTIVITY	50	-0.09941254	0.379210924
PROTEIN TYROSINE KINASE ACTIVATOR ACTIVITY	10	0.668707226	0.004930331

Table S5. miRNA KEGG.

GO Term	Genes Tested	Model Coefficient	p-value
GLYCOLYSIS GLUCONEOGENESIS	51	0.086839985	0.455411028
CITRATE CYCLE TCA CYCLE	24	-0.265289165	0.083377498
PENTOSE PHOSPHATE PATHWAY	24	-0.019674303	0.905861207
PENTOSE AND GLUCURONATE INTERCONVERSIONS	14	0.148959932	0.50234514
FRUCTOSE AND MANNOSE METABOLISM	31	0.090390152	0.54459536
GALACTOSE METABOLISM	23	-0.252585385	0.108567343
ASCORBATE AND ALDARATE METABOLISM	14	0.425204856	0.040108777
FATTY ACID METABOLISM	36	0.172475471	0.214289114
STEROID BIOSYNTHESIS	17	0.027800582	0.889256788
PRIMARY BILE ACID BIOSYNTHESIS	10	-0.065788934	0.795829563
STEROID HORMONE BIOSYNTHESIS	31	0.080971131	0.586752658
OXIDATIVE PHOSPHORYLATION	117	-0.189274084	0.008315894
PURINE METABOLISM	140	-0.029314177	0.670943505
PYRIMIDINE METABOLISM	94	-0.193629857	0.015636249
ALANINE ASPARTATE AND GLUTAMATE METABOLISM	29	0.179648713	0.248632551
GLYCINE SERINE AND THREONINE METABOLISM	25	-0.025955463	0.873301241
CYSTEINE AND METHIONINE METABOLISM	28	-0.148902781	0.314587712
VALINE LEUCINE AND ISOLEUCINE DEGRADATION	40	0.132735735	0.313751259
VALINE LEUCINE AND ISOLEUCINE BIOSYNTHESIS	10	-0.536615378	0.013491591
LYSINE DEGRADATION	41	0.143340515	0.273694265
ARGININE AND PROLINE METABOLISM	47	-0.102166864	0.379565542
HISTIDINE METABOLISM	26	0.185573404	0.255608147

TYROSINE METABOLISM	34	0.048999051	0.729496099
PHENYLALANINE METABOLISM	17	0.033968996	0.865006001
TRYPTOPHAN METABOLISM	37	0.371465014	0.004311241
BETA ALANINE METABOLISM	20	0.238859404	0.199652948
TAURINE AND HYPOTAURINE METABOLISM	7	-0.389910262	0.139718185
SELENOAMINO ACID METABOLISM	22	-0.125539523	0.45595029
GLUTATHIONE METABOLISM	42	-0.252266837	0.029677269
STARCH AND SUCROSE METABOLISM	32	0.145092182	0.324982609
N GLYCAN BIOSYNTHESIS	46	-0.33193439	0.002082826
OTHER GLYCAN DEGRADATION	14	-0.011049438	0.959610969
O GLYCAN BIOSYNTHESIS	25	0.043688017	0.791351789
AMINO SUGAR AND NUCLEOTIDE SUGAR METABOLISM	41	0.249512777	0.054330266
GLYCOSAMINOGLYCAN DEGRADATION	20	0.019603398	0.915045686
GLYCOSAMINOGLYCAN BIOSYNTHESIS CHONDROITIN SULFATE	20	-0.041011614	0.821069681
GLYCOSAMINOGLYCAN BIOSYNTHESIS KERATAN SULFATE	15	-0.573068168	0.000803476
GLYCOSAMINOGLYCAN BIOSYNTHESIS HEPARAN SULFATE	21	0.092389623	0.610581314
GLYCEROLIPID METABOLISM	42	0.313917426	0.013405506
INOSITOL PHOSPHATE METABOLISM	50	-0.035881797	0.754872678
GLYCOSYLPHOSPHATIDYLINOSITOL GPI ANCHOR BIOSYNTHESIS	24	-0.206396706	0.188111274
GLYCEROPHOSPHOLIPID METABOLISM	64	0.058682789	0.571117537
ETHER LIPID METABOLISM	24	0.16427567	0.334607044
ARACHIDONIC ACID METABOLISM	39	0.061984024	0.640075637
LINOLEIC ACID METABOLISM	16	0.24381306	0.234783868
ALPHA LINOLENIC ACID METABOLISM	12	0.28226344	0.227499904
SPHINGOLIPID METABOLISM	34	-0.152548295	0.25593084
GLYCOSPHINGOLIPID BIOSYNTHESIS LACTO AND NEOLACTO SERIES	20	-0.458427715	0.003130447
GLYCOSPHINGOLIPID BIOSYNTHESIS GLOBO SERIES	11	-0.153196482	0.514669399
GLYCOSPHINGOLIPID BIOSYNTHESIS GANGLIO SERIES	14	-0.159898158	0.441877818
PYRUVATE METABOLISM	33	0.06450252	0.65463585
GLYOXYLATE AND DICARBOXYLATE METABOLISM	15	0.10864743	0.613397898
PROPANOATE METABOLISM	28	0.161000876	0.306782463
BUTANOATE METABOLISM	26	0.255951368	0.117952882
ONE CARBON POOL BY FOLATE	15	-0.104974854	0.608489799
RIBOFLAVIN METABOLISM	12	-0.22583088	0.305129812
NICOTINATE AND NICOTINAMIDE METABOLISM	21	-0.350188248	0.030001492
PANTOTHENATE AND COA BIOSYNTHESIS	13	0.119531644	0.603729758
FOLATE BIOSYNTHESIS	10	-0.035480172	0.889994649
RETINOL METABOLISM	37	0.130530243	0.33944954
PORPHYRIN AND CHLOROPHYLL METABOLISM	26	-0.061830546	0.696091965
TERPENOID BACKBONE BIOSYNTHESIS	13	0.083718812	0.71589081
LIMONENE AND PINENE DEGRADATION	9	0.226657612	0.420306537
NITROGEN METABOLISM	20	0.05760745	0.755399287
SULFUR METABOLISM	10	-0.010868982	0.966414932
AMINOACYL TRNA BIOSYNTHESIS	39	-0.278349764	0.022953317
METABOLISM OF XENOBIOTICS BY CYTOCHROME P450	44	0.011808123	0.924016275
DRUG METABOLISM CYTOCHROME P450	45	0.039761885	0.74657885
DRUG METABOLISM OTHER ENZYMES	35	0.052612623	0.706530336
BIOSYNTHESIS OF UNSATURATED FATTY ACIDS	21	0.117346917	0.519237599
ABC TRANSPORTERS	34	0.145544771	0.309503963
RIBOSOME	87	-0.188369664	0.023638851
RNA DEGRADATION	55	-0.062313742	0.566913584
RNA POLYMERASE	29	-0.019671572	0.896606675
BASAL TRANSCRIPTION FACTORS	33	-0.115112719	0.404607685
DNA REPLICATION	35	-0.06750072	0.619975072
SPLICEOSOME	122	-0.23692353	0.000652637
PROTEASOME	42	-0.263346106	0.021784354
PROTEIN EXPORT	22	0.089024663	0.615111853
PPAR SIGNALING PATHWAY	55	0.117593203	0.295213197
BASE EXCISION REPAIR	32	-0.018339067	0.898773128
NUCLEOTIDE EXCISION REPAIR	40	0.182883565	0.167744933
MISMATCH REPAIR	22	-0.120146478	0.480262255
HOMOLOGOUS RECOMBINATION	25	0.02686363	0.870393858
NON HOMOLOGOUS END JOINING	12	0.130210544	0.592262538
MAPK SIGNALING PATHWAY	239	0.019156852	0.720524388
ERBB SIGNALING PATHWAY	84	0.044065199	0.625638717
CALCIUM SIGNALING PATHWAY	147	0.046854469	0.493353681

CYTOKINE CYTOKINE RECEPTOR INTERACTION	197	0.114133857	0.055816811
CHEMOKINE SIGNALING PATHWAY	157	0.074599269	0.262064248
PHOSPHATIDYLINOSITOL SIGNALING SYSTEM	71	-0.017883992	0.853608295
NEUROACTIVE LIGAND RECEPTOR INTERACTION	176	0.114455685	0.069248059
CELL CYCLE	121	-0.243360441	0.000458837
OOCYTE MEIOSIS	99	-0.156602926	0.047094014
P53 SIGNALING PATHWAY	67	-0.327351759	0.000263952
UBIQUITIN MEDIATED PROTEOLYSIS	126	-0.013995674	0.847873264
SNARE INTERACTIONS IN VESICULAR TRANSPORT	35	-0.109239706	0.416062398
REGULATION OF AUTOPHAGY	24	0.271925017	0.108318003
LYSOSOME	112	-0.141638892	0.058027072
ENDOCYTOSIS	168	0.081006298	0.208567918
PEROXISOME	68	-0.082503013	0.396446512
MTOR SIGNALING PATHWAY	50	0.136772155	0.246736972
APOPTOSIS	81	-0.071539329	0.424265257
CARDIAC MUSCLE CONTRACTION	64	-0.042838652	0.672836994
VASCULAR SMOOTH MUSCLE CONTRACTION	93	0.111262424	0.198425407
WNT SIGNALING PATHWAY	135	-0.01812849	0.796894705
DORSO VENTRAL AXIS FORMATION	23	0.354126129	0.045193107
NOTCH SIGNALING PATHWAY	44	-0.059837574	0.623102624
HEDGEHOG SIGNALING PATHWAY	47	0.389877301	0.001122923
TGF BETA SIGNALING PATHWAY	74	-0.060282274	0.52113682
AXON GUIDANCE	120	0.198384807	0.009770492
VEGF SIGNALING PATHWAY	66	0.055768445	0.584481413
FOCAL ADHESION	190	-0.023027736	0.698354113
ECM RECEPTOR INTERACTION	77	0.103142188	0.277781019
CELL ADHESION MOLECULES CAMS	112	-0.147046917	0.048219664
ADHERENS JUNCTION	70	-0.187333193	0.044477508
TIGHT JUNCTION	117	-0.091880439	0.215581484
GAP JUNCTION	75	-0.107065783	0.245192047
COMPLEMENT AND COAGULATION CASCADES	53	0.178137584	0.119091123
ANTIGEN PROCESSING AND PRESENTATION	62	-0.067525289	0.509532107
RENIN ANGIOTENSIN SYSTEM	13	0.049530042	0.828793856
TOLL LIKE RECEPTOR SIGNALING PATHWAY	88	0.121832337	0.17083461
NOD LIKE RECEPTOR SIGNALING PATHWAY	52	-0.029106357	0.796519449
RIG I LIKE RECEPTOR SIGNALING PATHWAY	58	-0.072328502	0.493712139
CYTOSOLIC DNA SENSING PATHWAY	42	-0.030829541	0.805944877
JAK STAT SIGNALING PATHWAY	122	-0.07186557	0.325589879
HEMATOPOIETIC CELL LINEAGE	63	0.004190156	0.967666214
NATURAL KILLER CELL MEDIATED CYTOTOXICITY	100	-0.037634439	0.643735047
T CELL RECEPTOR SIGNALING PATHWAY	92	0.108896895	0.211231083
B CELL RECEPTOR SIGNALING PATHWAY	65	0.08630121	0.403484465
FC EPSILON RI SIGNALING PATHWAY	66	0.013277609	0.89561148
FC GAMMA R MEDIATED PHAGOCYTOSIS	82	-0.093354097	0.291626452
LEUKOCYTE TRANSENDOTHELIAL MIGRATION	104	-0.210228671	0.005547248
INTESTINAL IMMUNE NETWORK FOR IGA PRODUCTION	38	-0.069411851	0.594803569
CIRCADIAN RHYTHM MAMMAL	13	0.678853705	0.000503987
LONG TERM POTENTIATION	66	-0.078636531	0.426710361
NEUROTROPHIN SIGNALING PATHWAY	124	-0.057048626	0.433172455
LONG TERM DEPRESSION	57	-0.094207046	0.374287321
OLFACTORY TRANSDUCTION	71	0.220839306	0.024510902
TASTE TRANSDUCTION	26	0.247973006	0.127611462
REGULATION OF ACTIN CYTOSKELETON	189	0.020740985	0.730153809
INSULIN SIGNALING PATHWAY	127	-0.086190527	0.227056477
GNRH SIGNALING PATHWAY	85	0.041827117	0.641029893
PROGESTERONE MEDIATED OOCYTE MATURATION	78	0.040843123	0.662602211
MELANOGENESIS	90	-0.034343336	0.688958402
ADIPOCYTOKINE SIGNALING PATHWAY	60	-0.095856812	0.352711327
TYPE II DIABETES MELLITUS	43	0.069849865	0.580565064
TYPE I DIABETES MELLITUS	34	-0.298089065	0.018836224
MATURITY ONSET DIABETES OF THE YOUNG	18	0.477260725	0.006825543
ALDOSTERONE REGULATED SODIUM REABSORPTION	36	0.114051027	0.412648701
VASOPRESSIN REGULATED WATER REABSORPTION	42	-0.02272262	0.856607943
PROXIMAL TUBULE BICARBONATE RECLAMATION	19	0.140470432	0.462558813
ALZHEIMERS DISEASE	146	-0.258184297	4.02E-05
PARKINSONS DISEASE	113	-0.212621177	0.00343061

AMYOTROPHIC LATERAL SCLEROSIS ALS	46	-0.051571762	0.665660099
HUNTINGTONS DISEASE	158	-0.173594822	0.00538128
PRION DISEASES	31	0.009092079	0.950800365
VIBRIO CHOLERAE INFECTION	49	-0.224078789	0.041796344
EPITHELIAL CELL SIGNALING IN HELICOBACTER PYLORI INFECTION	63	-0.184096035	0.062692212
PATHOGENIC ESCHERICHIA COLI INFECTION	49	-0.170528156	0.128093682
LEISHMANIA INFECTION	61	0.035114871	0.739660063
PATHWAYS IN CANCER	303	-0.067838326	0.146682412
COLORECTAL CANCER	58	-0.052384957	0.622259112
RENAL CELL CARCINOMA	69	0.118793421	0.238998532
PANCREATIC CANCER	67	0.085575732	0.400220285
ENDOMETRIAL CANCER	49	0.097691169	0.411938414
GLIOMA	64	-0.007426058	0.942134567
PROSTATE CANCER	82	-0.039235229	0.662100444
THYROID CANCER	26	0.11822023	0.470540566
BASAL CELL CARCINOMA	48	0.01233699	0.917128245
MELANOMA	65	0.121683281	0.240461496
BLADDER CANCER	41	-0.224463067	0.062140541
CHRONIC MYELOID LEUKEMIA	70	-0.074096902	0.441763346
ACUTE MYELOID LEUKEMIA	55	0.084671243	0.450381446
SMALL CELL LUNG CANCER	81	-0.237245423	0.005153162
NON SMALL CELL LUNG CANCER	51	0.020001099	0.86219568
ASTHMA	17	-0.029470685	0.881151206
AUTOIMMUNE THYROID DISEASE	32	-0.250158796	0.060042432
SYSTEMIC LUPUS ERYTHEMATOSUS	110	0.022747516	0.772372971
ALLOGRAFT REJECTION	28	-0.341992324	0.011993536
GRAFT VERSUS HOST DISEASE	30	-0.292831104	0.031294545
PRIMARY IMMUNODEFICIENCY	27	0.120385409	0.451860038
HYPERTROPHIC CARDIOMYOPATHY HCM	73	-0.058859852	0.534035123
ARRHYTHMOGENIC RIGHT VENTRICULAR CARDIOMYOPATHY ARVC	67	-0.082319622	0.401894231
DILATED CARDIOMYOPATHY	78	0.024037332	0.796737622
VIRAL MYOCARDITIS	60	-0.182870217	0.070994807
